# Intramolecular Friedel–Crafts alkylation with a silylium-ion-activated cyclopropyl group: formation of tricyclic ring systems from benzyl-substituted vinylcyclopropanes and hydrosilanes†

**DOI:** 10.1039/d0sc05553k

**Published:** 2020-10-29

**Authors:** Tao He, Guoqiang Wang, Peng-Wei Long, Sebastian Kemper, Elisabeth Irran, Hendrik F. T. Klare, Martin Oestreich

**Affiliations:** Institut für Chemie, Technische Universität Berlin Strasse des 17. Juni 115 10623 Berlin Germany hendrik.klare@tu-berlin.de martin.oestreich@tu-berlin.de

## Abstract

A trityl-cation-initiated annulation of benzyl-substituted vinylcyclopropanes (VCPs) with hydrosilanes is reported. Two Si–C(sp^3^) bonds and one C(sp^2^)–C(sp^3^) bond are formed in this process where an intramolecular 6-*endo-tet* Friedel–Crafts alkylation of a silylium-ion-activated cyclopropane ring is the rate-determining key step. The reaction mechanism is proposed based on computations and is in agreement with experimental observations. The new reaction leads to an unprecedented silicon-containing 6/6/5-fused ring system. A phenethyl-substituted VCP derivative yields another unknown tricycle having 6/6/6 ring fusion by reacting in a related but different way involving a 6-*exo-tet* ring closure.

## Abstract 2

Downstream to alkene hydrosilylation, the opening of the cyclopropane ring in benzyl-substituted VCPs is interlinked with an S_E_Ar of the aryl group.

## Graphical abstract

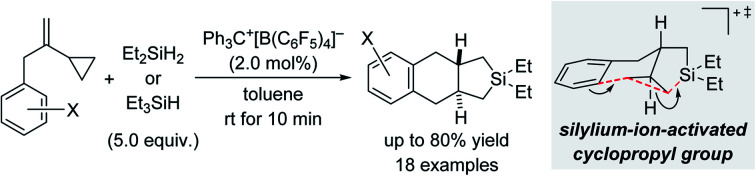

## Introduction

**Scheme 1:**
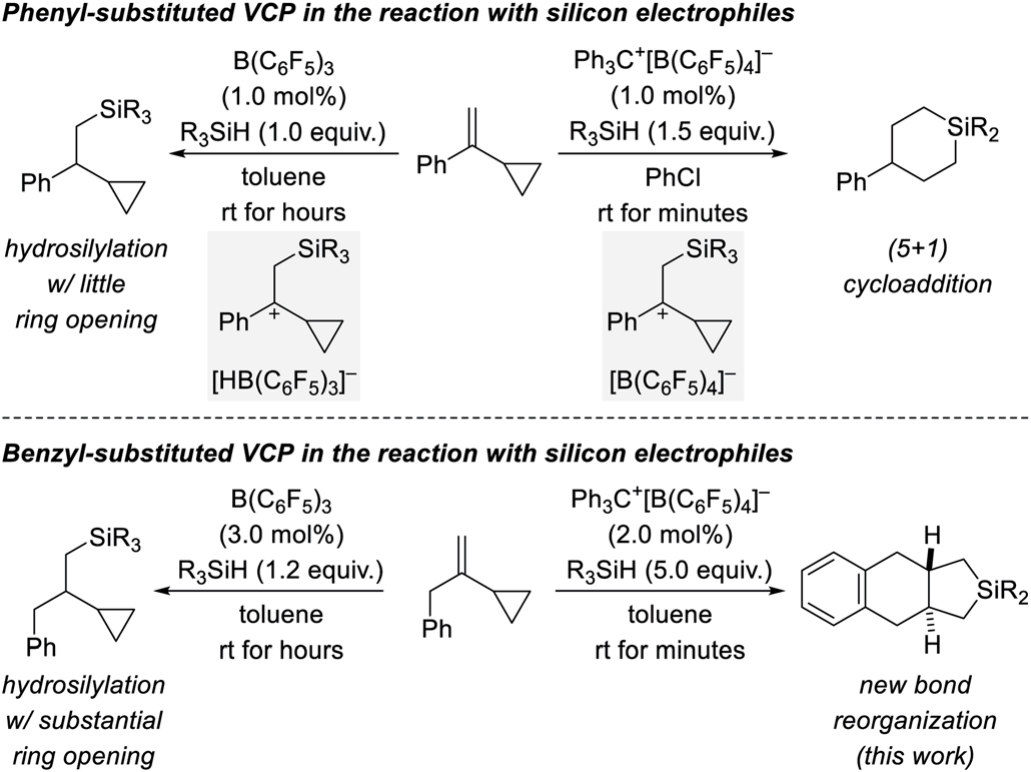
Diverse outcomes from the reaction of phenyl- and benzyl-substituted VCPs and hydrosilanes (R_3_SiH = 3°, 2°, and 1° hydrosilanes with R = alkyl and/or aryl).

We recently became interested in the reactivity of catalytically generated silicon electrophiles towards cyclopropane derivatives.^1–3^ Our investigations typically comprise B(C_6_F_5_)_3_/hydrosilane combinations^4^ and silylium-ion-like reactants^5^ emerging from hydrosilanes and the trityl salt Ph_3_C^+^[B(C_6_F_5_)_4_]^−^ as an initiator. VCPs as substrates specifically caught our attention because these versatile building blocks can react in diverse ways.^6^ This is also true for their reactions with silicon electrophiles (Scheme 1, top).^2,3^ These reactions involve the intermediacy of β-silicon-stabilized^7^ cyclopropylcarbinyl cations^8^ with different counteranions (gray boxes). Depending on the hydride source, the outcomes could not be more different. For aryl-substituted VCPs, the B(C_6_F_5_)_3_-catalyzed hydrosilylation proceeds generally with little ring opening (top left).^2^ In contrast, treatment of these VCPs with Ph_3_C^+^[B(C_6_F_5_)_4_]^−^ in the presence of various hydrosilanes affords silicon-containing six-membered rings as a result of a formal (5 + 1) cycloaddition accompanied by an aryl migration (top right).^3^ The situation changes again when replacing the aryl substituent by a benzyl group (Scheme 1, bottom). The additional methylene group makes a huge difference. The hydrosilylation under B(C_6_F_5_)_3_ catalysis is now plagued with ring opening, likely due to poorer stabilization of the carbocation intermediate (bottom left).^2^ Strikingly, the silylium-ion-promoted setup gives rise to yet another product where the aryl group becomes part of the product's ring system (bottom right). We report here a new trityl-cation-initiated cycloaddition of benzyl-substituted VCPs and hydrosilanes involving an intramolecular Friedel–Crafts alkylation. Our study includes a full experimental and computational mechanistic analysis.

## Results and discussion

Using 2 mol% of Ph_3_C^+^[B(C_6_F_5_)_4_]^−^ as an initiator, we began investigating the reaction of VCP 1a and excess Et_2_SiH_2_ (2a) in various arene solvents at ambient temperature (Table 1). In benzene as solvent, 2,2-diethyl-2,3,3*a*,4,9,9*a*-hexahydro-1*H*-naphtho[2,3-*c*]silole (3aa) was found as the major product along with ring-opened 4-methyl-5-phenylpentyl-substituted silane 4ab and six-membered ring system 5aa (entry 1). As ring-opening hydrosilylation would yield an unsaturated compound,^1^ we speculated that the formation of saturated 4ab (through initially formed 4aa)^9,10^ involves an additional alkene hydrogenation by protonation of the VCP to form a cyclopropylcarbinyl cation followed by hydride transfer from the hydrosilane. The proton could be released from a Wheland intermediate in the course of an assumed Friedel–Crafts reaction. Addition of norbornene as a proton scavenger had no effect though (entry 2). In turn, the silicon-containing ring system 5aa could be the result of the aforementioned silylium-ion-promoted hydrosilylation of cyclopropanes^1^ coupled with an *endo* cyclization of an allylbenzene intermediate (see discussion of the mechanism).

**Table 1:** Optimization of the trityl-cation-initiated cycloaddition^a^

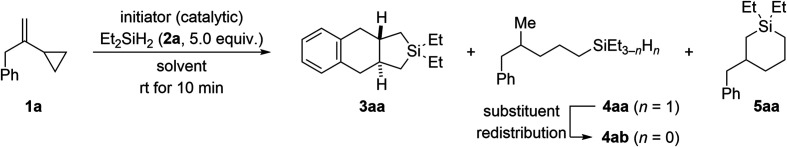					
Entry	Initiator (mol%)	Solvent	Yield^b^ (%)		
			3aa	4aa/4ab	5aa
1	Ph_3_C^+^[B(C_6_F_5_)_4_]^−^ (2.0 mol%)	Benzene	51	34	9
2^c^	Ph_3_C^+^[B(C_6_F_5_)_4_]^−^ (2.0 mol%)	Toluene-*d*_8_	61	26	9
3^d^	Ph_3_C^+^[B(C_6_F_5_)_4_]^−^ (2.0 mol%)	Benzene	41	47	8
4^e^	Ph_3_C^+^[B(C_6_F_5_)_4_]^−^ (1.0 mol%)	Benzene	28	28	<5
5	Ph_3_C^+^[B(C_6_F_5_)_4_]^−^ (5.0 mol%)	Benzene	55	30	13
6^f^	Ph_3_C^+^[B(C_6_F_5_)_4_]^−^ (2.0 mol%)	Benzene	52	33	11
7	Ph_3_C^+^[B(C_6_F_5_)_4_]^−^ (2.0 mol%)	Chlorobenzene	56	20	14
8	Ph_3_C^+^[B(C_6_F_5_)_4_]^−^ (2.0 mol%)	1,2-C_6_H_4_Cl_2_	61	19	9
9	Ph_3_C^+^[B(C_6_F_5_)_4_]^−^ (2.0 mol%)	Toluene-*d*_8_	65	21	13
10	Et_3_Si^+^[CHB_11_H_5_Br_6_]^−^ (2.0 mol%)	Toluene-*d*_8_	50	23	25
11	[(C_6_H_6_)·H]^+^[CHB_11_H_5_Br_6_]^−^ (2.0 mol%)	Toluene-*d*_8_	48	26	23

a All reactions were performed with VCP 1a (0.10 mmol) and the indicated amounts of the initiator and Et_2_SiH_2_ (2a) under argon atmosphere in the indicated arene solvent (0.5 mL, 0.2 M) at room temperature. Unless otherwise noted, conversion was greater than 95% for each entry as estimated by ^1^H NMR spectroscopy using CH_2_Br_2_ as an internal standard.

b Yields were estimated by ^1^H NMR spectroscopy using CH_2_Br_2_ as an internal standard and tend to be too high because of the long relaxation time of CH_2_Br_2_.

c 1.0 equiv. of norbornene used.

d 2.0 equiv. of Et_2_SiH_2_ (2a) used.

e 39% of VCP 1a recovered.

f Performed at 0.1 M.

A diminished yield was observed at a lower loading of the dihydrosilane (2.0 instead of 5.0 equiv.; entry 3). Poor conversion was seen with less initiator; the product distribution was also affected unfavorably at 1.0 mol% catalyst loading (entry 4). More of Ph_3_C^+^[B(C_6_F_5_)_4_]^−^ was without effect (entry 5). Likewise, the reaction outcome did not change at lower concentration (0.1 M instead of 0.2 M; entry 6). The solvent had a minor effect on the ratio of 3aa, 4ab, and 5aa (entries 7–9), and we eventually proceeded with toluene for the highest overall yield. In accordance with our mechanistic picture, both the counteranion-stabilized silylium ion Et_3_Si^+^[CHB_11_H_5_Br_6_]^−^ (ref. 11) and the benzenium ion [(C_6_H_6_)·H]^+^[CHB_11_H_5_Br_6_]^−^ (ref. 12) could also be used to initiate this cycloaddition, yet with more pronounced formation of undesired cyclic 5aa (entries 10 and 11).

**Scheme 2:**
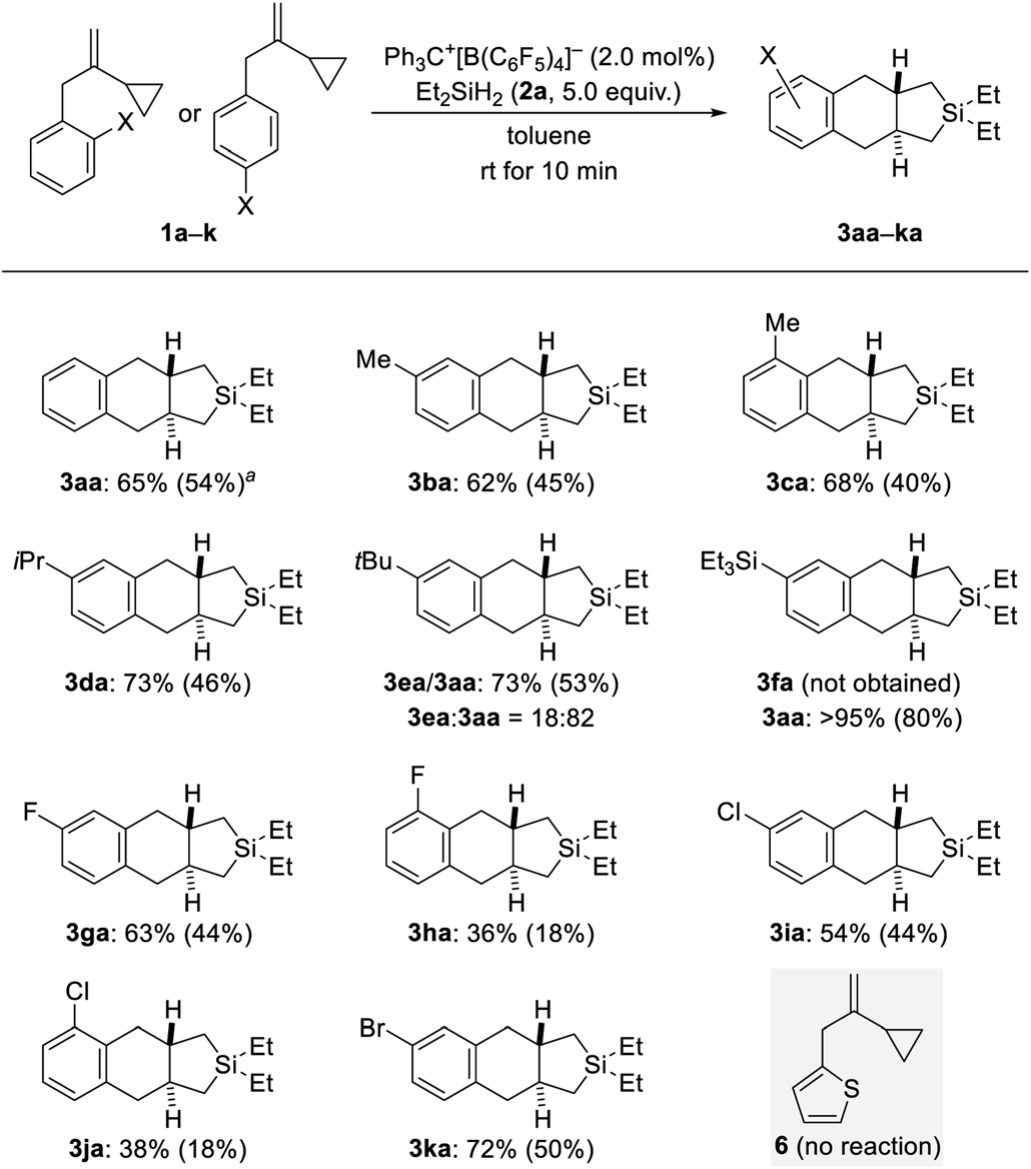
Scope I: variation of the *ortho*- and *para*-substituent of the benzyl group in VCPs 1. All reactions were performed on 0.30 mmol scale unless noted otherwise. Conversion was generally greater than 95% as estimated by ^1^H NMR spectroscopy using CH_2_Br_2_ as an internal standard. Yields were estimated by ^1^H NMR spectroscopy using CH_2_Br_2_ as an internal standard and tend to be too high because of the long relaxation time of CH_2_Br_2_; isolated yields in parentheses are of analytically pure material after flash chromatography on silica gel. ^*a*^65% (48%) on a 1.2 mmol scale.

**Scheme 3:**
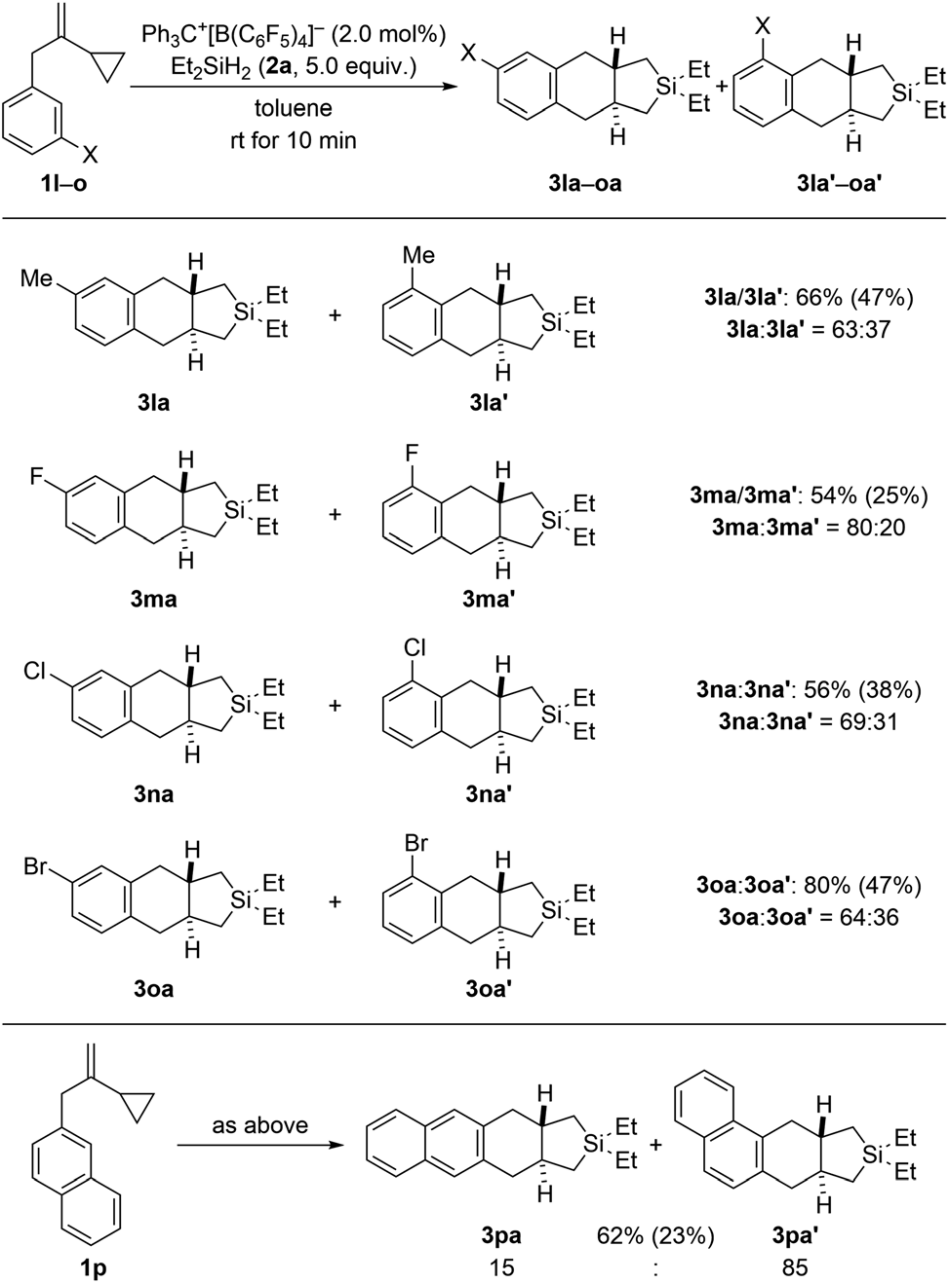
Scope II: variation of the *meta*-substituent of the benzyl group in VCPs 1. See caption of Scheme 2 for further details. Regio-isomeric ratios determined by ^1^H NMR spectroscopy after purification.

**Scheme 4:**
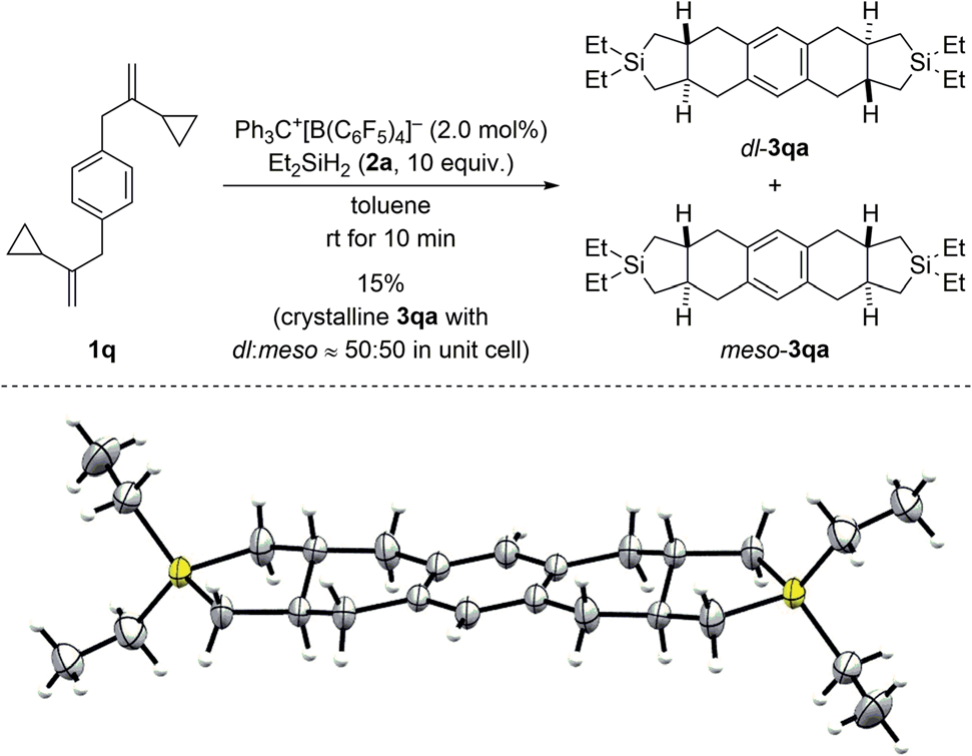
Scope III: reaction of a bis(VCP) system (top; see caption of Scheme 2 for further details) and molecular structure of the bis-*trans*-isomer *meso*-3qa (bottom; thermal ellipsoids are shown at the 50% probability level).

With the optimized protocol in hand (Table 1, entry 9), we probed the substrate scope (Schemes 2–4). Electronic and steric effects of the substituent on the aryl group were examined with VCPs 1a–k (*ortho* and *para*, Scheme 2) and 1l–o (*meta*, Scheme 3). Parent VCP 1a afforded the cycloadduct 3aa in 54% isolated yield. Yields were moderate for VCPs with aryl rings bearing an electron-donating methyl or isopropyl group; the position of the substituent made no difference (1b–d → 3ba–da). VCP 1e decorated with a *tert*-butyl group did participate equally well but underwent predominant de-*tert*-butylation^13^ to mainly afford 3aa rather than 3ea. Similarly, Et_3_Si-substituted 1f suffered complete desilylation^14^ with none of 3fa being formed. Interestingly, the isolated yield of 80% for 3aa was significantly higher than that obtained for the parent system (54% for 1a → 3aa). We explain this with loss of a silylium ion instead of a proton, thus eliminating the aforementioned formation of the saturated byproducts 4aa and 4ab. This finding not only provides a solution of how to bypass unwanted alkene protonation but is also evidence for the intermediacy of Wheland complexes and as such a Friedel–Crafts-type mechanism. Halogen atoms were tolerated in this reaction but there was a clear difference between *ortho*- and *para*-substituted VCP derivatives (1g–k → 3ga–ka). Yields were moderate for the *para*- and low for the *ortho*-halogenated substrates (the preparation of the *ortho*-bromine-substituted VCP was unsuccessful). Both electronic and steric effects could be responsible for that. Besides, we also subjected VCP 6 with a thien-2-yl instead of the phenyl group to the procedure but did not obtain the cycloadduct; 6 was almost completely recovered (gray box, Scheme 2).

Regioisomeric mixtures of the cycloadducts were generated when starting from representative VCPs with *meta*-substituted aryl groups (1l–o → 3la–oa/3la′–oa′, Scheme 3, top). Bond formation occurred preferentially in the less hindered *ortho*-position with regioisomeric ratios ranging from 63 : 37 to 80 : 20. Conversely, VCP 1p with a naphth-2-yl group yielded the kinetically favored, more hindered cycloadduct 3pa′ with good regiocontrol (Scheme 3, bottom). The cycloaddition of a bis(vinylcyclopropane) system was also tested (1q → 3qa/3qa′, Scheme 4, top); *para*-substituted 1q would first convert into a *meta*,*para*-disubstituted intermediate (not shown) which, in turn, would then give rise to a mixture of the regioisomers 3qa and 3qa′ (not shown) in the second cycloaddition event. A complex reaction mixture was experimentally found, and the separation by various chromatography methods failed. Part of the problem is that 3qa is formed as a mixture of racemic *dl* and *meso* diastereomers in approximately 50 : 50 ratio; the same likely applies to regioisomeric 3qa′ which we were not able to isolate (not shown). Slow evaporation of a solution of that regio- and diastereomeric mixture (four compounds; assuming only *trans* and no *cis* annulation) in ethyl acetate and cyclohexane (1 : 1) eventually led to the co-crystallization of bis-*trans*-isomers *dl*-3qa and *meso*-3qa. The *meso* compound is depicted in Scheme 4 (bottom; see the ESI† for details).^15^ The relative configuration of all annulation products was deduced from this molecular structure and assigned as *trans*, thereby confirming the result obtained from recorded and simulated ^1^H NMR spectra.

**Scheme 5:**
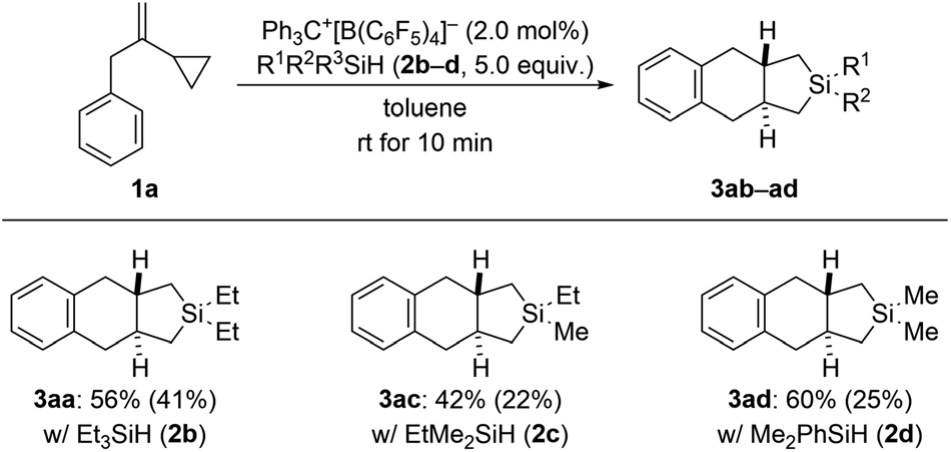
Scope IV: variation of the hydrosilane in the cycloaddition of VCP 1a. See caption of Scheme 2 for further details.

In our previous study (see Scheme 1, top right),^3^ we had already shown that, apart from dihydrosilanes, monohydrosilanes can be used with similar success. The reason for this is the ability of silylium ions to cleave Si–C(sp^3^) as well as Si–C(sp^2^) bonds,^16,17^ corresponding to an exchange of alkyl or aryl group between two silicon centers.^9^ Hence, when there is no Si–H bond available (as in a quaternary silane) an alkyl or aryl group can be abstracted by another silylium ion (*cf.* the ESI† of ref. 3). We therefore tested a few tertiary hydrosilanes (2b–d) in the cycloaddition of model VCP 1a (Scheme 5). As expected, Et_3_SiH (2b) afforded the same product 3aa as obtained with Et_2_SiH_2_ (2a) albeit in lower yield. Demethylation was again preferred over cleavage of an ethyl group with EtMe_2_SiH (2c). Dearylation occurred when employing Me_2_PhSiH (2d). These observations are in line with those made earlier.^3,16,17^

**Scheme 6:**
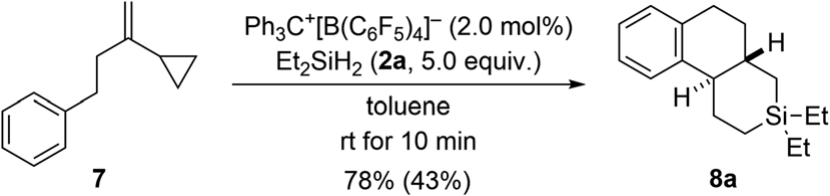
Scope V: going from benzyl to phenethyl in the VCP. See caption of Scheme 2 for further details.

The results with the phenyl-substituted VCPs^3^ and those with a benzyl substituent (see Scheme 1, bottom) underscore the structural richness that becomes accessible with this chemistry. We had already reported that the corresponding VCP with a cyclohexyl group led to a complex reaction mixture.^3^ However, going from a benzyl to a phenethyl group was successful, yielding yet another unknown silicon-containing ring system (7 → 8a, Scheme 6). The relative configuration was deduced from the ^3^*J*_H,H_ coupling (10.6 Hz) in the ^1^H NMR spectrum of the highlighted H nuclei.

**Scheme 7:**
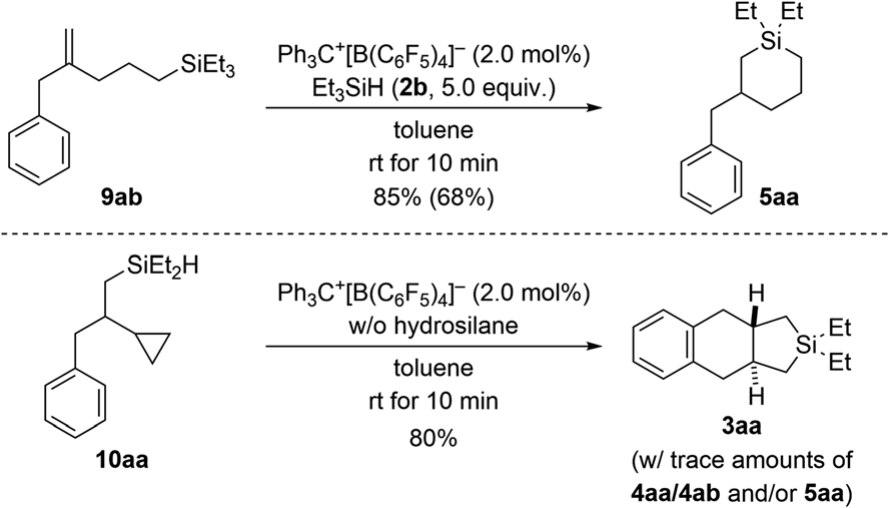
Control experiments I: verification of ring opening of the cyclopropyl group in VCPs prior to engagement of the alkene unit. See caption of Scheme 2 for further details.

To gain insight into the reaction mechanism, we designed control experiments to distinguish between reaction pathways with the ring opening of the cyclopropyl group happening before or after manipulation of the alkene (Scheme 7). We showed before that silylium-ion-promoted ring-opening hydrosilylation of cyclopropanes is feasible^1^ but had excluded this possibility for phenyl-substituted VCPs^3^ in the (5 + 1) cycloaddition (see Scheme 1, top right). If hydrosilylation of the cyclopropyl group in VCPs 1 is the initial step, 2-substituted allylbenzene derivatives 9 are likely intermediates. When subjecting independently prepared 9ab to the standard procedure using Et_3_SiH (2b), an *endo* cyclization furnished product 5aa in high yield (top). With 5aa being formed as minor byproduct in the annulation reaction (*cf.*Table 1), we can state that ring opening preceding the functionalization of the alkene in the VCP is a competing pathway. In turn, potential intermediate 10aa^2^ arising from alkene hydrosilylation of VCP 1a and Et_2_SiH_2_ (2a) transformed cleanly into the cycloadduct 3aa upon treatment with trityl borate Ph_3_C^+^[B(C_6_F_5_)_4_]^−^ in the absence of external hydrosilane (bottom). Hence, the opening of the cyclopropyl ring is interlinked with the intramolecular Friedel–Crafts-type bond formation at the aryl group^18^ and is downstream to the alkene hydrosilylation.

**Scheme 8:**
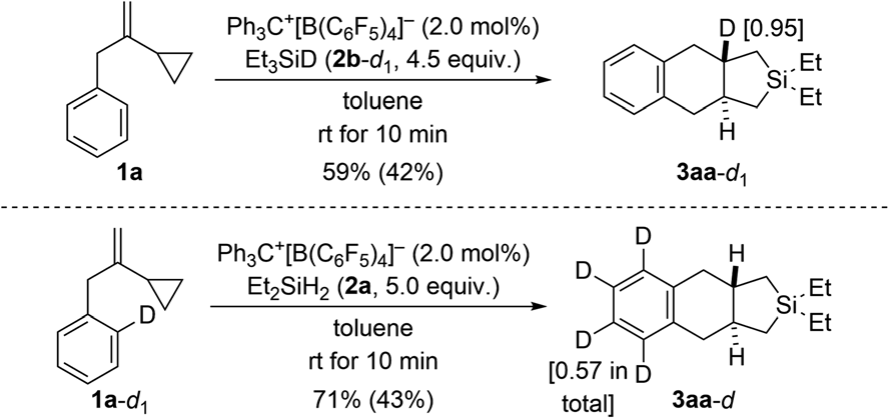
Control experiments II: deuterium-labeling of the hydrosilane or the VCP. See caption of Scheme 2 for further details. The deuteration grades were estimated by NMR spectroscopy.

In another set of control experiments, we employed deuterium-labeled hydrosilane Et_3_SiD (2b-*d*_1_) and model VCP 1a-*d*_1_ with an *ortho*-C–D bond (Scheme 8). When 1a was reacted with 2b-*d*_1_, the deuterium label was exclusively incorporated into one of the angular positions of cycloadduct 3aa-*d*_1_ (top). To verify whether cleavage of the *ortho*-C–H bond is involved in the rate-determining step, 1a-*d*_1_ was subjected to the standard setup (bottom). However, H/D scrambling had occurred at all positions of the phenylene group in 3aa-*d*.^19^

**Scheme 9:**
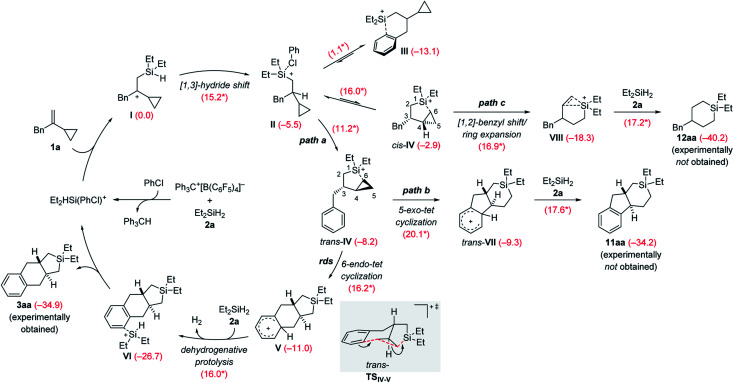
Catalytic cycle of the silylium-ion-promoted cycloaddition of VCP 1a and Et_2_SiH_2_ (2a). For each reaction step, the Gibbs free reaction energies and barriers (labeled with an asterisk) in kcal mol^−1^ were computed with the M06-2X functional (see the ESI† for details). rds = rate-determining step.

The rough mechanistic picture was refined by density functional theory (DFT) calculations at the M062X/cc-PVTZ//M062X/6-31G(d,p) level.^20^ Computations were performed on the model reaction of VCP 1a and Et_2_SiH_2_ (2a) with Ph_3_C^+^[B(C_6_F_5_)_4_]^−^ as the initiator (Scheme 9; see the ESI for details and Fig. S85 and S86† for the free-energy profiles and the optimized structures). The solvent effect was taken into consideration using a polarizable continuum model (PCM)^21^ for both geometry optimizations and single-point energy calculations. Chlorobenzene was chosen to avoid the complexity of possible proton exchange between Wheland intermediates and toluene. The cycloadduct 3aa is obtained in moderate yield in chlorobenzene (Table 1, entry 7). We have previously shown that β-silicon-stabilized cyclopropylcarbinyl cation I, generated from the association of VCP 1a and the hydrogen-substituted silylium ion [Et_2_HSi(PhCl)]^+^, is much more stable than other donor-stabilized silylium ions such as the corresponding chlorobenzene-, hydrosilane-, or cyclopropane-stabilized systems.^3^ Unless otherwise noted, adduct I is considered the reference minimum for the estimation of the relative energy of intermediates or transition states calculated here.

The β-silylcarbenium ion I then undergoes an intramolecular [1,3]-hydride shift from the silicon atom to the benzylic carbon atom to arrive at the chlorobenzene-stabilized silylium ion II over a barrier of 15.2 kcal mol^−1^. We also calculated the potential intermolecular hydride transfer from Et_2_SiH_2_ (2a) to I but this pathway can be excluded because of an energetically higher transition state (19.0 *versus* 15.2 kcal mol^−1^, Fig. S84 in the ESI†). Subsequent reorganization can lead to several intramolecularly donor-stabilized silylium ions III (arene stabilization; see ref. 16*b* for a crystallographically characterized derivative) and *cis*-IV/*trans*-IV (cyclopropane stabilization with *cis*- or *trans*-configuration). In the case of benzyl-substituted VCPs, III is much more stable than either of the two cyclopropane-stabilized silylium ions IV. Conversion of III into *cis*-IV or *trans*-IV requires an activation barrier of 16.0 and 11.2 kcal mol^−1^, respectively. The *trans*-isomer is kinetically and thermodynamically more accessible than the *cis*-isomer. It is therefore *trans*-IV which engages in a subsequent intramolecular Friedel–Crafts alkylation reaction of a silylium-ion-activated cyclopropane ring.^18^

A 6-*endo-tet* ring closure by nucleophilic attack of the phenyl group at C5 with concomitant cleavage of the distal C5–C6 bond and formation of a bond between C6 and the silicon atom leads to tricyclic Wheland intermediate V (path a). This process through transition state *trans*-TS_IV–V_ (gray box) is exergonic by −2.8 kcal mol^−1^ with an activation barrier of 16.2 kcal mol^−1^. It is also the rate-determining step, consistent with the rapid reaction rate at room temperature. The Brønsted acid V reacts with hydrosilane 2a by dehydrogenative protolysis^17,22^ to form the experimentally obtained product 3aa*via*VI along with dihydrogen and the chlorobenzene-stabilized silylium-ion catalyst (Δ*G*^‡^ = 16.0 kcal mol^−1^). The overall Gibbs free energy change Δ*G* is −34.9 kcal mol^−1^ (calculated from the Gibbs energy difference between 3aa and 1a/2a). Based on transition state *trans*-TS_IV–V_, the predicted relative configuration of 3aa is *trans*, and is supported by ^1^H NMR spectroscopy (^3^*J*_H,H_ = 11.7 Hz) and was eventually confirmed by X-ray diffraction (Scheme 4, bottom).

We considered other kinetically less favorable pathways such as a the 5-*exo-tet* cyclization (path b, cleavage of the proximal C4–C6 bond leading to 11aa) or [1,2]-benzyl shift/ring expansion (path c, leading to 12aa). Neither 11aa nor 12aa were observed experimentally. The route to 12aa corresponds to the previously reported (5 + 1) cycloaddition of phenyl-substituted VCPs^3^ (see Fig. S87–S90 in the ESI†). However, the annulation of a phenethyl-substituted VCP 7 to afford the 6/6/6-fused ring system 8a can be explained by a related 6-*exo-tet* cyclization corresponding to path b (see Schemes 6 and S12 in the ESI† for the computed pathway).

**Scheme 10:**
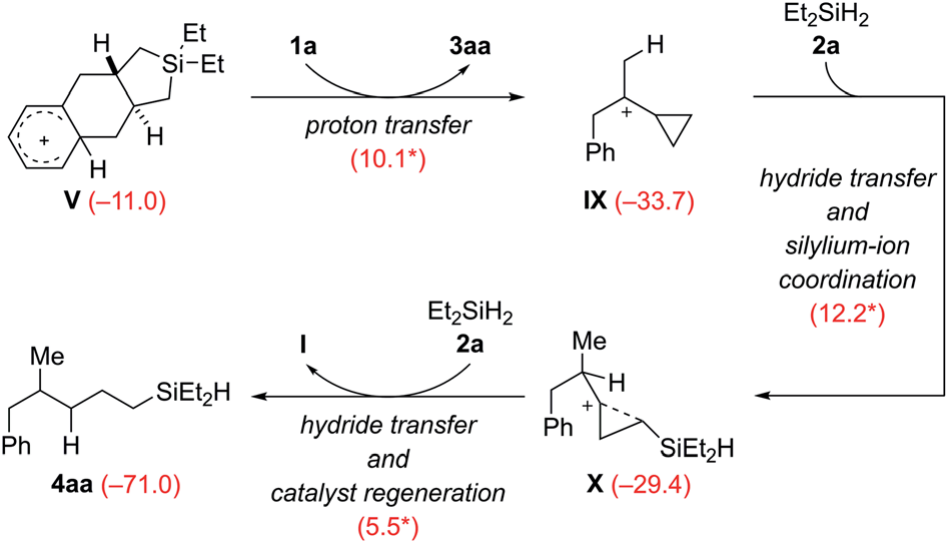
Computed pathway of the formation of byproduct 4aa, precursor of 4ab (see discussion of Table 1).

The Wheland intermediate V is the proton source in this reaction that can also be the starting point of side reactions. Instead of the above dehydrogenative protolysis of the hydrosilane over a barrier of 16.0 kcal mol^−1^ (V → VI, Scheme 9), this Brønsted acid can also transfer a proton to the C=C double bond of VCP 1a, releasing the product 3aa and the cyclopropylcarbinyl cation^8^IX (Scheme 10). The barrier of this protonation step is only 10.1 kcal mol^−1^ and is as such kinetically competitive. Subsequent hydride transfer from Et_2_SiH_2_ (2a) to IX gives a γ-silicon-stabilized carbenium ion X. This undergoes hydride abstraction from another molecule of 2a to yield open-chain product 4aa; the silylium ion released at the same time forms the β-silicon-stabilized cyclopropylcarbinyl cation I with VCP 1a. This ring-opening hydrosilylation of a cyclopropane promoted by a silylium ion is a known reaction and was recently studied by us in detail.^1^ With the low barriers involved, the formation of 4aa and eventually 4ab after rapid substituent redistribution^9,10^ cannot be avoided.

## Conclusion

The present work showcases that reactions of VCPs with different electrophilic silicon reagents do lead to drastically different outcomes (*cf.*Scheme 1). The substituent on the VCP and the choice of the silicon electrophile together with the counteranion of the β-silicon-stabilized cyclopropyl carbocation intermediates decide their fate. With silylium-ion-like reagents, benzyl- and phenethyl-substituted VCPs lead to silicon-containing 6/6/5- and 6/6/6-fused ring systems, respectively, both of which previously unknown motifs. The corresponding phenyl-substituted VCPs engage in a (5 + 1) cycloaddition under otherwise identical reaction conditions.^3^ The annulation sequence is initiated by the trityl cation and then maintained by self-regeneration of the silylium-ion reagent.^5*a*^ Two Si–C(sp^3^) bonds and one C(sp^2^)–C(sp^3^) bond are formed with an intramolecular Friedel–Crafts alkylation of a silylium-ion-activated cyclopropane ring as the key step (6-*endo-tet* for 6/6/5 system starting from benzyl-substituted VCP and 6-*exo-tet* for 6/6/6 system starting from phenethyl-substituted VCP). Given the high number of possible reaction pathways, the chemoselectivity and preference for one product with any of the substrates is remarkable.

## Conflicts of interest

There are no conflicts to declare.

## Supplementary Material

SC-012-D0SC05553K-s001

SC-012-D0SC05553K-s002
