# Supplementary material for: Intramolecular Friedel–Crafts alkylation with a silylium-ion-activated cyclopropyl group: formation of tricyclic ring systems from benzyl-substituted vinylcyclopropanes and hydrosilanes
Source: Chem Sci. 2020 Oct 29;12(2):569–75. doi: 10.1039/d0sc05553k (PMC8178999; doi:10.1039/d0sc05553k)
Supplement: SC-012-D0SC05553K-s001 [file SC-012-D0SC05553K-s001.pdf]

Electronic Supplementary Material (ESI) for Chemical Science.
This journal is © The Royal Society of Chemistry 2020

Intramolecular Friedel–Crafts alkylation with a silylium-ion-activated cyclopropyl group: formation of tricyclic ring systems from benzyl-substituted vinylcyclopropanes and hydrosilanes

Tao He, Guoqiang Wang, Peng-Wei Long, Sebastian Kemper, Elisabeth Irran, Hendrik F. T. Klare* and Martin Oestreich*

Institut für Chemie, Technische Universität Berlin
Straße des 17. Juni 115, 10623 Berlin, Germany
hendrik.klare@tu-berlin.de
martin.oestreich@tu-berlin.de

Electronic Supplementary Information

Table of Contents

**1**	**General Information**	**S3**
**2**	**Experimental Details for the Synthesis of $Et_3Si^+[CHB_{11}H_5Br_6]^-$**	**S4**
**3**	**Experimental Details for the Synthesis of Benzyl-Substituted Vinylcyclopropanes (GP 1–3)**	**S5**
3.1	Method A for the Synthesis of Benzyl-Substituted Vinylcyclopropanes **1** (**GP 1**)	S5
3.2	Method B for the Synthesis of Benzyl-Substituted Vinylcyclopropanes **1** (**GP 2**)	S7
3.3	Method C for the Synthesis of Benzyl-Substituted Vinylcyclopropanes **1** (**GP 3**)	S8
3.4	Characterization Data of Vinylcyclopropanes **1a–q** and **6**	S9
**4**	**Experimental Details for the Trityl-Cation-Initiated Reaction of Benzyl-Substituted Vinylcyclopropanes with Hydrosilanes**	**S18**
4.1	General Procedure for the Trityl-Cation-Initiated Reaction of Benzyl-Substituted Vinylcyclopropanes with Hydrosilanes (**GP 4**)	S18
4.2	Characterization Data of Products **3aa–ka**, **3pa'**, **3qa**, **3ac** and **3ad**	S18
4.3	Characterization Data of Byproduct Triethyl(4-methyl-5-phenylpentyl)silane (**4ab**)	S28
4.4	^{1}H NMR Spectra of the Trityl-Cation-Initiated Reaction of Benzyl-Substituted Vinylcyclopropane **1a** with Et_2SiH_2 (**2a**): Assignment of all Compounds	S28
**5**	**Experimental Details of the Mechanistic Control Experiments**	**S30**
5.1	Synthesis of Vinylcyclopropane **7**, Cyclization Precursors **9ab**, **10aa** and Byproduct **4aa**	S30
5.2	Control Experiments with VCP **7**, Precursors **9ab**, **10aa** and Byproduct **4aa**	S36
5.3	Mechanistic Control Experiments with Deuterium-Labeled Et_3SiD (**2b**-d_1) and Vinylcyclopropane **1a**-d_1	S39
**6**	**NMR Spectra**	**S43**
**7**	**Crystallographic Data**	**S123**
**8**	**Computational Data**	**S125**
8.1	Results of the DFT Calculations	S125
8.2	Cartesian Coordinates of the Optimized Stationary Points	S134
**9**	**References**	**S168**

1 General Information

All reactions were performed in flame-dried glassware using an *MBraun* glovebox or conventional Schlenk techniques under a static pressure of argon (glovebox) or nitrogen (fume hood) unless otherwise stated. Standard solvents and reagents were obtained from commercial suppliers and used as received unless otherwise stated. Technical grade solvents for extraction and chromatography were distilled prior to use. Dichloromethane (CH_2Cl_2) and tetrahydrofurane (THF) were dried over calcium hydride and sodium, respectively, and freshly distilled prior to use. Dry benzene (C_6H_6) and *n*-pentane were obtained from an *MBraun* solvent purification system (SPS-800), degassed by three freeze-pump-thaw cycles, and stored in a glovebox over thermally activated 4 Å molecular sieves. Chlorobenzene (C_6H_5Cl) and all hydrosilanes were dried over CaH_2, distilled, degassed by three freeze-pump-thaw cycles, and stored in a glovebox over thermally activated 4 Å molecular sieves. $Ph_3C^+[B(C_6F_5)_4]^-$,[S1] $Ph_3C^+[CHB_{11}H_5Br_6]^-$[S2] and $[(C_6H_6)\cdot H]^+[CHB_{11}H_5Br_6]^-$[S2] were synthesized according to a reported procedures. Analytical thin-layer chromatography (TLC) was performed on silica gel 60 F254 glass plates. Flash column chromatography was performed on silica gel 60 (40–63 µm, 230–400 mesh ASTM) using the indicated solvents. ^1H, ^{13}C, ^{19}F, and ^{29}Si NMR spectra were recorded in C_6D_6, C_6D_5Cl, $CDCl_3$ or CD_2Cl_2 on a *Bruker* AV500 instrument. C_6D_6 and C_6D_5Cl were degassed by three freeze-pump-thaw cycles and stored in a glovebox over thermally activated 4 Å molecular sieves. Chemical shifts are reported in parts per million (ppm) and are referenced to the residual solvent resonance as the internal standard (C_6D_5H: δ = 7.16 ppm for ^1H NMR and C_6D_6: δ = 128.06 ppm for ^{13}C NMR; C_6D_4HCl: δ 6.96, 6.99, and 7.14 ppm for ^1H NMR and C_6D_5Cl: δ 125.96, 128.25, 129.26, and 134.19 ppm for ^{13}C NMR; $CHCl_3$: δ = 7.26 ppm for ^1H NMR and $CDCl_3$: δ = 77.16 ppm for ^{13}C NMR; $CDHCl_2$: δ = 5.32 ppm for ^1H NMR and CD_2Cl_2: δ = 53.84 ppm for ^{13}C NMR). ^{19}F and ^{29}Si NMR spectra are referenced in compliance with the unified scale for NMR chemical shifts as recommended by the IUPAC stating the chemical shift relative to CCl_3F and TMS, respectively.[S3] Data are reported as follows: chemical shift, multiplicity (s = singlet, d = doublet, t = triplet, q = quartet, hept = heptet, m = multiplet, br = broad signal), coupling constants (Hz), and integration. Infrared (IR) spectra were recorded on an *Agilent Technologies* Cary 630 FT-IR spectrometer equipped with an ATR unit or a *Jasco* FT/IR-4100 spectrometer, and the signals are reported in wavenumbers (cm^{-1}). Melting points (m.p.) were determined with a *Stuart Scientific* SMP20 instrument and were not corrected. High resolution mass spectra (HRMS) were obtained from the *Laboratory of Mass Spectrometry* at the *Institut für Chemie*, *Technische Universität Berlin*. The single-crystal diffraction data were collected with an *Agilent* SuperNova diffractometer.

2 Experimental Details for the Synthesis of $Et_3Si^+[CHB_{11}H_5Br_6]^-$

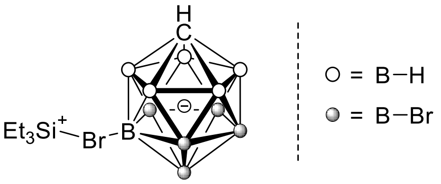

$C_7H_{21}B_{11}Br_6Si$
M = 731.63 g/mol

According to a reported procedure,[S2,S4] $Ph_3C^+[CHB_{11}H_5Br_6]^-$ (50.0 mg, 0.058 mmol, 1.0 equiv) was suspended in toluene (~10 drops) and treated with triethylsilane (27.3 mg, 0.235 mmol, 4.0 equiv). After stirring the reaction mixture at room temperature for 24 h, *n*-pentane (0.5 mL) was added to the resulting white suspension. The precipitate was collected by filtration, washed with *n*-pentane (3 × ~10 drops), and briefly dried under vacuum to afford silylium carborate $Et_3Si^+[CHB_{11}H_5Br_6]^-$ (38.0 mg, 90%) as a white solid. The silylium salt can be stored for several weeks in the glove box at –30 °C without any decomposition and loss of reactivity.

^{1}H NMR (500 MHz, C_6D_5Cl, 298 K): δ = 0.80 (t, *J* = 8.0 Hz, 9H), 1.00 (q, *J* = 8.0 Hz, 6H), ~1.9–3.2 (br m, 6H) ppm. **^{11}B NMR** (161 MHz, C_6D_5Cl, 298 K): δ = –19.8 (d, *J* = 167.8 Hz), –9.1 (s), –1.0 (s) ppm. **^{13}C{^{1}H} NMR** (126 MHz, C_6D_5Cl, 298 K): δ = 6.2, 7.9 ppm. **^{1}H/^{29}Si HMQC NMR** (500/99 MHz, C_6D_5Cl, 298 K, optimized for *J* = 7 Hz): δ = 0.80/98.6, 1.00/98.6 ppm.

3 Experimental Details for the Synthesis of Benzyl-Substituted Vinylcyclopropanes (GP 1–3)

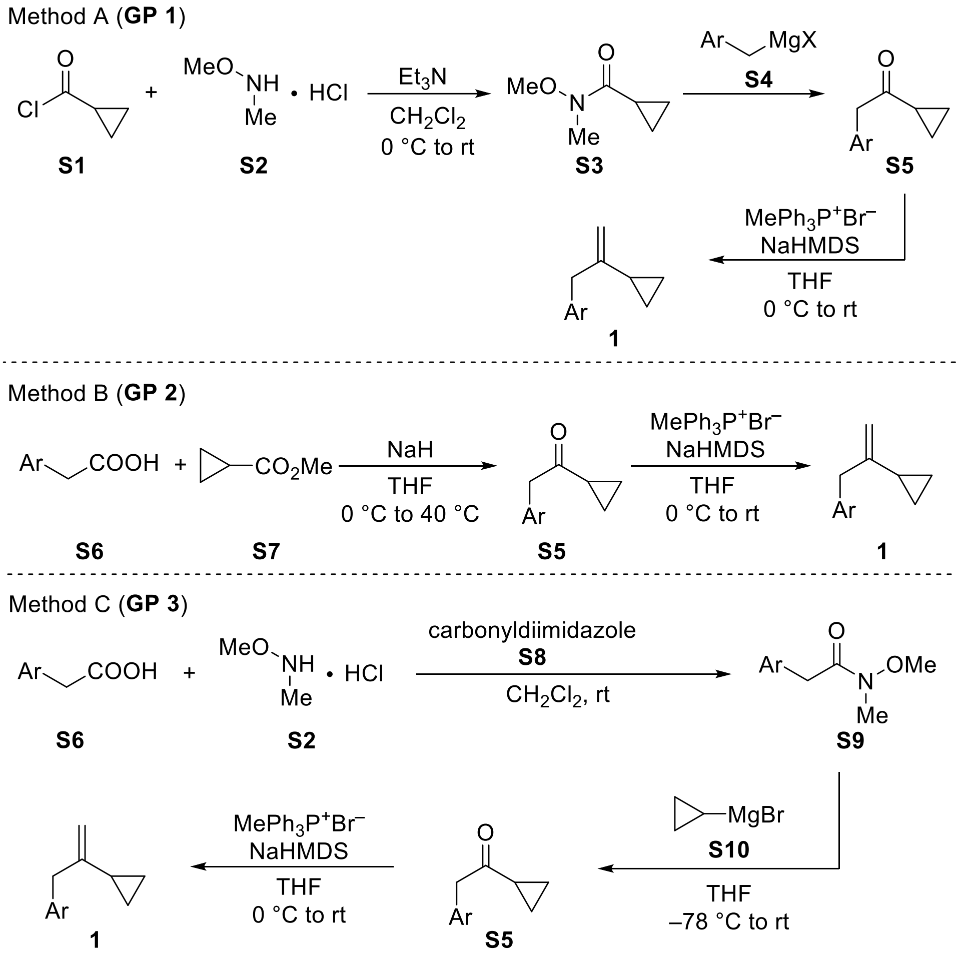

Scheme S1. Synthetic Routes to Benzyl-Substituted Vinylcyclopropanes.

3.1 Method A for the Synthesis of Benzyl-Substituted Vinylcyclopropanes 1 (GP 1)

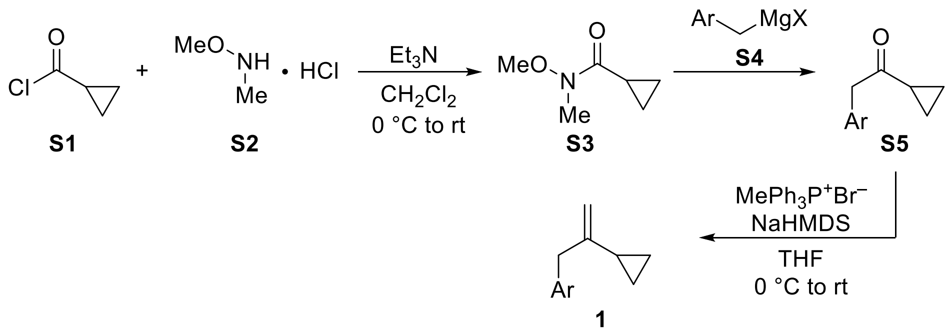

Scheme S2. Synthesis of Benzyl-Substituted Vinylcyclopropanes via **GP1**.

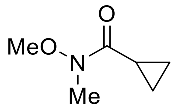

S3

$C_6H_{11}NO_2$

M = 129.08 g/mol

According to a reported procedure,[S5] triethylamine (28.0 mL, 200 mmol, 2.0 equiv) was added dropwise to a solution of cyclopropanecarbonyl chloride (**S1**, 10.5 g, 100 mmol, 1.0 equiv) and *N*,*O*-dimethylhydroxylammonium chloride (**S2**, 9.8 g, 100 mmol, 1.0 equiv) in anhydrous CH_2Cl_2 (150 mL) at 0 °C. After complete addition, the reaction mixture was stirred at ambient temperature for 1 h and quenched by the addition of saturated aqueous $NaHCO_3$ solution (100 mL). The organic phase was separated, and the aqueous layer was extracted with CH_2Cl_2 (3 × 50 mL). The combined organic phases were washed with brine (50 mL) and dried over $MgSO_4$. After removal of the solvent, the Weinreb amide **S3** was obtained as a pale yellow oil (11.6 g, 90% yield), which was used directly in the next step without further purification. **^{1}H NMR** (500 MHz, $CDCl_3$, 298 K): δ = 0.74–0.87 (m, 2H), 0.89–1.02 (m, 2H), 2.06–2.19 (m, 1H), 3.20 (s, 3H), 3.75 (s, 3H) ppm. **^{13}C{^{1}H} NMR** (126 MHz, $CDCl_3$, 298 K): δ = 8.0, 9.9, 32.7, 61.6, 174.8 ppm. The NMR spectroscopic data are in accordance with those reported.[S5]

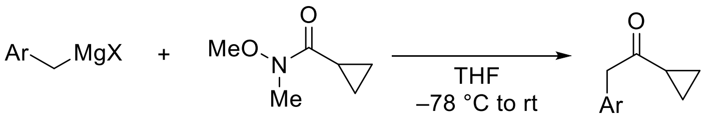

S4 **S3** **S5**

According to a reported procedure,[S6] commercially available or freshly prepared[S7] Grignard reagent **S4** (20 mmol, 1.0 equiv) is added dropwise to a solution of the Weinreb amide **S3** (22 mmol, 1.1 equiv) in anhydrous THF (20 mL) at –78 °C. After complete addition, the mixture is allowed to warm to room temperature and stirred at this temperature for additional 2 h. The reaction mixture is quenched by the addition of water (10 mL) and saturated aqueous NH_4Cl solution (20 mL). The organic phase is separated, and the aqueous layer is extracted with EtOAc (3 × 20 mL). The combined organic phases are dried over $MgSO_4$ and concentrated under reduced pressure. The residue is purified by flash column chromatography on silica gel using cyclohexane/EtOAc mixtures as eluent to afford the corresponding ketone **S5** in analytically pure form.

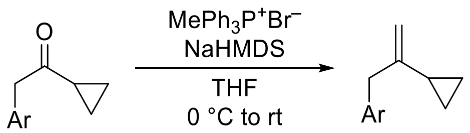

S5 **1**

According to a reported procedure,[S5] sodium bis(trimethylsilyl)amide (NaHMDS, 3.6 mL of a 2.0 M solution in THF, 7.2 mmol, 1.2 equiv) is added dropwise to a solution of methyltriphenyl-phosphonium bromide (2.6 g, 7.2 mmol, 1.2 equiv) in anhydrous THF (10 mL) at 0 °C. After

complete addition, the reaction mixture is stirred at 0 °C for additional 30 min, followed by dropwise addition of a solution of the corresponding ketone **S5** (6 mmol, 1.0 equiv) in THF (10 mL) at 0 °C. The reaction mixture is allowed to warm to room temperature, stirred overnight and quenched by the addition of water (10 mL) and saturated aqueous NH_4Cl solution (10 mL). The organic phase is separated, and the aqueous layer is extracted with EtOAc (3 × 20 mL). The combined organic phases are dried over $MgSO_4$ and concentrated under reduced pressure. The residue is purified by flash column chromatography on silica gel using cyclohexane or cyclohexane/EtOAc mixtures as eluent to afford the corresponding benzyl-substituted vinylcyclopropanes **1** in analytically pure form.

3.2 Method B for the Synthesis of Benzyl-Substituted Vinylcyclopropanes 1 (GP 2)

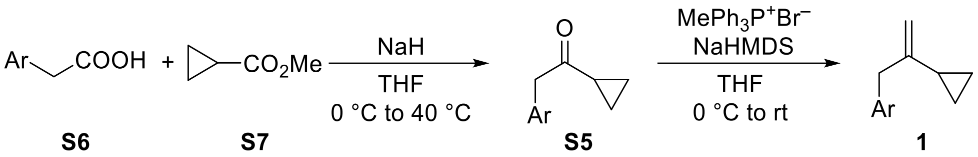

Scheme S3. Synthesis of Benzyl-Substituted Vinylcyclopropanes via **GP 2**.

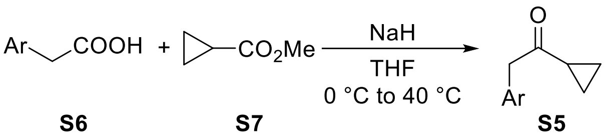

According to a reported procedure,[S8] a solution of sodium hydride (1.2 g of a 60% dispersion in mineral oil, 30 mmol, 1.5 equiv) in anhydrous THF (60 mL) is added dropwise to a solution of arylacetic acid **S6** (20 mmol, 1.0 equiv) in anhydrous THF (20 mL) at 0 °C. After complete addition, the reaction mixture is stirred at 40 °C for 1 h, followed by the addition of a solution of methyl cyclopropanecarboxylate (**S7**, 2.2 g, 22 mmol, 1.1 equiv) in anhydrous THF (10 mL). The reaction mixture is stirred at 40 °C overnight and then quenched by the addition of saturated aqueous NH_4Cl solution (60 mL). The organic phase is separated, and the aqueous layer is extracted with EtOAc (3 × 50 mL). The combined organic phases are washed with brine (50 mL) and dried over $MgSO_4$. After removal of the solvent under reduced pressure, the residue is purified by flash column chromatography on silica gel using cyclohexane/EtOAc mixtures as eluent to afford the corresponding ketone **S5** in analytically pure form.

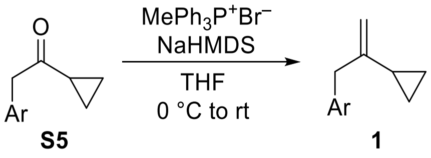

For the reaction procedure of the Wittig reaction to afford the benzyl-substituted vinylcyclopropanes **1** from the corresponding ketone **S5**, see section 3.1.

3.3 Method C for the Synthesis of Benzyl-Substituted Vinylcyclopropanes 1 (GP 3)

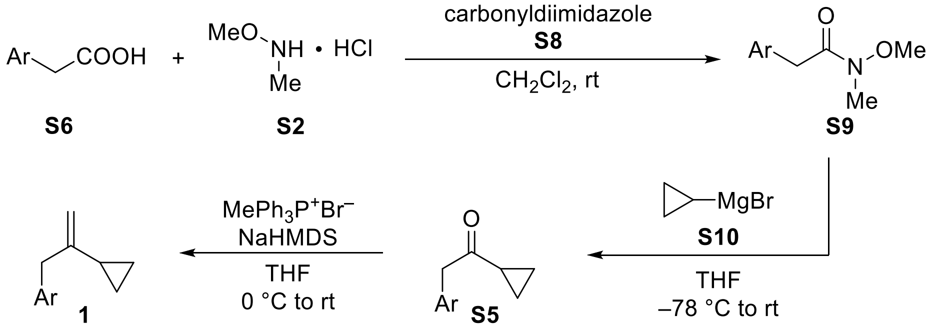

Scheme S4. Synthesis of Benzyl-Substituted Vinylcyclopropanes via **GP 3**.

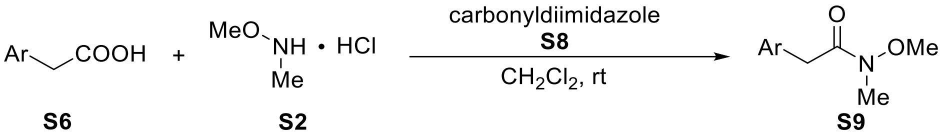

According to a reported procedure,[S9] carbonyldiimidazole (**S8**, 4.2 g, 26 mmol, 1.3 equiv) is added to a solution of arylacetic acid **S6** (20 mmol, 1.0 equiv) in anhydrous CH_2Cl_2 (50 mL) at ambient temperature. After stirring for 1 h, *N*,*O*-dimethylhydroxylammonium chloride (**S2**, 3.9 g, 40 mmol, 2.0 equiv) is added, and the reaction mixture is stirred overnight at ambient temperature. The reaction mixture is quenched by the addition of water (20 mL) and saturated aqueous NH_4Cl solution (20 mL). The organic phase is separated, and the aqueous layer is extracted with CH_2Cl_2 (2 × 30 mL). The combined organic phases are washed with brine (30 mL), dried over $MgSO_4$ and concentrated under reduced pressure. Purification of the residue by flash column chromatography on silica gel using cyclohexane/EtOAc mixtures as eluent affords the corresponding Weinreb amide **S9** in analytically pure form.

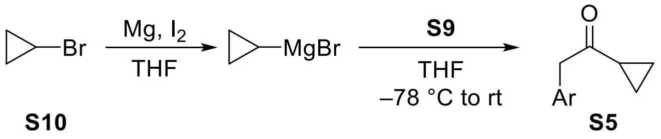

According to a reported procedure,[S10] a solution of cyclopropyl bromide (**S10**, 2.5 g, 21 mmol, 1.05 equiv) in anhydrous THF (20 mL) is slowly added to a suspension of magnesium turnings (760 mg, 32 mmol, 1.6 equiv) and iodine (catalytic amounts) in anhydrous THF (5 mL) at room temperature. After complete addition, the mixture is heated to reflux for 2 h. The thus obtained solution of cyclopropylmagnesium bromide (**S10**) in THF is slowly added to a solution of Weinreb amide **S9** (20 mmol, 1.0 equiv) in anhydrous THF (20 mL) at –78 °C. After complete addition, the mixture is allowed to warm to room temperature and stirred at this temperature for additional 2 h. The reaction mixture is then quenched by the addition of water (10 mL) and saturated aqueous NH_4Cl solution (20 mL). The organic phase is separated, and the aqueous layer is extracted with EtOAc (3 × 20 mL). The combined organic phases are dried over $MgSO_4$

and concentrated under reduced pressure. Purification of the residue by flash column chromatography on silica gel using cyclohexane/EtOAc mixtures as eluent affords the corresponding ketone **S5** in analytically pure form.

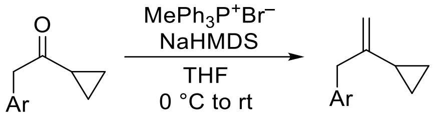

S5 **1**

For the reaction procedure of the Wittig reaction to afford the benzyl-substituted vinylcyclopropanes **1** from the corresponding ketone **S5**, see section 3.1.

3.4 Characterization Data of Vinylcyclopropanes 1a–q and 6

3.4.1 (2-Cyclopropylallyl)benzene (**1a**)

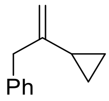

1a

$C_{12}H_{14}$
M = 158.11 g/mol

Prepared from phenylacetic acid (2.7 g, 20.0 mmol) according to **GP 1**. Flash column chromatography on silica gel using cyclohexane afforded VCP **1a** as a colorless oil (1.6 g, 51% yield). **R*f*** = 0.71 (cyclohexane). **^{1}H NMR** (500 MHz, $CDCl_3$, 298 K): δ = 0.42–0.46 (m, 2H), 0.58–0.62 (m, 2H), 1.28 (dddd, *J* = 13.7, 8.3, 5.3, 1.0 Hz, 1H), 3.39 (s, 2H), 4.62–4.63 (m, 1H), 4.73–4.74 (m, 1H), 7.19–7.23 (m, 3H), 7.28–7.31 (m, 2H) ppm. **^{13}C{^{1}H} NMR** (126 MHz, $CDCl_3$, 298 K): δ = 6.4, 16.1, 43.0, 108.5, 126.1, 128.4, 129.2, 140.0, 150.5 ppm. The NMR spectroscopic data are in accordance with those reported.[S11]

3.4.2 (2-Cyclopropylallyl)-4-methylbenzene (**1b**)

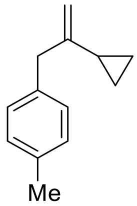

1b

$C_{13}H_{16}$
M = 172.13g/mol

Prepared from 4-methylphenylacetic acid (3.0 g, 20.0 mmol) according to **GP 1**. Flash column chromatography on silica gel using cyclohexane afforded VCP **1b** as a colorless oil (0.9 g, 27% yield). **R*f*** = 0.74 (cyclohexane). **IR** (ATR): ṽ = 3079, 3044, 3002, 2918, 2858, 1640, 1511, 1430, 1378, 1104, 1046, 1019, 958, 882, 846, 807, 764 cm^{-1}. **^{1}H NMR** (500 MHz, $CDCl_3$, 298 K): δ = 0.44–0.47 (m, 2H), 0.59–0.62 (m, 2H), 1.25–1.33 (m, 1H), 2.34 (s, 3H), 3.35 (s, 2H), 4.62–

4.63 (m, 1H), 4.72–4.73 (m, 1H), 7.10–7.14 (m, 4H) ppm. **^{13}C{^{1}H} NMR** (101 MHz, $CDCl_3$, 298 K): δ = 6.4, 16.1, 21.2, 42.5, 108.3, 129.1 (2C), 135.6, 136.9, 150.7 ppm. **HRMS** (APCI): calculated for $C_{13}H_{17}^{\bullet+}$ $[M+H]^{\bullet+}$: 173.1330; found 173.1327.

3.4.3 (2-Cyclopropylallyl)-2-methylbenzene (**1c**)

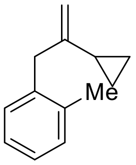

1c
$C_{13}H_{16}$
M = 172.13g/mol

Prepared from 2-methylphenylacetic acid (3.0 g, 20.0 mmol) according to **GP 3**. Flash column chromatography on silica gel using cyclohexane afforded VCP **1c** as a colorless oil (380 mg, 11% yield). **R*f*** = 0.60 (cyclohexane). **IR** (ATR): ṽ = 3078, 3010, 2910, 1640, 1490, 1458, 1429, 1378, 1048, 1018, 884, 819, 776, 739 cm^{-1}. **^{1}H NMR** (500 MHz, $CDCl_3$, 298 K): δ = 0.47–0.51 (m, 2H), 0.62–0.65 (m, 2H), 1.34 (tt, *J* = 8.5, 5.3 Hz, 1H), 2.27 (s, 3H), 3.34 (s, 2H), 4.38–4.39 (m, 1H), 4.71–4.72 (m, 1H), 7.12–7.16 (m, 4H) ppm. **^{13}C{^{1}H} NMR** (126 MHz, $CDCl_3$, 298 K): δ = 6.2, 16.6, 19.4, 39.9, 108.1, 125.9, 126.4, 130.2, 130.2, 136.9, 138.0, 149.5 ppm. **HRMS** (APCI): calculated for $C_{13}H_{17}^{\bullet+}$ $[M+H]^{\bullet+}$: 173.1330; found 173.1327.

3.4.4 1-(2-Cyclopropylallyl)-4-isopropylbenzene (**1d**)

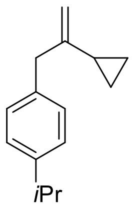

1d
$C_{15}H_{20}$
M = 200.16 g/mol

Prepared from 4-isopropylphenylacetic acid (3.6 g, 20.0 mmol) according to **GP 3**. Flash column chromatography on silica gel using cyclohexane afforded VCP **1d** as a colorless oil (1.2 g, 30% yield). **R*f*** = 0.80 (cyclohexane). **IR** (ATR): ṽ = 3080, 3006, 2958, 2925, 2900, 2869, 1641, 1511, 1460, 1421, 1382, 1098, 1052, 1018, 885, 846, 815 cm^{-1}. **^{1}H NMR** (500 MHz, $CDCl_3$, 298 K): δ = 0.41–0.48 (m, 2H), 0.57–0.63 (m, 2H), 1.25 (d, *J* = 6.9 Hz, 6H), 1.27–1.32 (m, 1H), 2.89 (hept, *J* = 6.8 Hz, 1H), 3.35 (s, 2H), 4.61–4.63 (m, 1H), 4.72 (s, 1H), 7.12–7.17 (m, 4H) ppm. **^{13}C{^{1}H} NMR** (126 MHz, $CDCl_3$, 298 K): δ = 6.4, 16.1, 24.2, 33.8, 42.5, 108.3, 126.4, 129.1, 137.3, 146.6, 150.7 ppm. **HRMS** (APCI): calculated for $C_{15}H_{21}^{\bullet+}$ $[M+H]^{\bullet+}$: 201.1643; found 201.1638.

3.4.5 (2-Cyclopropylallyl)-4-(*tert*-Butyl)benzene (**1e**)

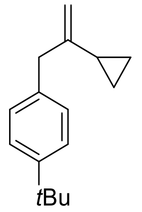

1e
$C_{16}H_{22}$
M = 214.17 g/mol

Prepared from 4-(*tert*-butyl)phenylacetic acid (3.8 g, 20.0 mmol) according to **GP 3**. Flash column chromatography on silica gel using cyclohexane afforded VCP **1e** as a colorless oil (985 mg, 23% yield). **R*f*** = 0.78 (cyclohexane). **IR** (ATR): ṽ = 3081, 3003, 2960, 2904, 2867, 1642, 1511, 1461, 1363, 1268, 1110, 1019, 883, 836, 813 cm^{-1}. **^{1}H NMR** (500 MHz, $CDCl_3$, 298 K): δ = 0.43–0.47 (m, 2H), 0.58–0.62 (m, 2H), 1.29–1.32 (m, 10H), 3.35 (s, 2H), 4.62–4.63 (m, 1H), 4.62–4.73 (m, 1H), 7.13–7.17 (m, 2H), 7.30–7.32 (m, 2H) ppm. **^{13}C{^{1}H} NMR** (126 MHz, $CDCl_3$, 298 K): δ = 6.4, 16.1, 31.6, 34.5, 42.4, 108.3, 125.3, 128.8, 136.9, 148.9, 150.6 ppm. **HRMS** (APCI): calculated for $C_{16}H_{23}^{\bullet+}$ $[M+H]^{\bullet+}$: 215.1800; found 215.1794.

3.4.6 (4-(2-Cyclopropylallyl)phenyl)triethylsilane (**1f**)

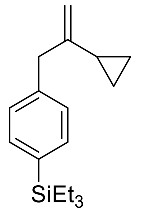

1f
$C_{18}H_{28}Si$
M = 272.20 g/mol

To a solution of (2-cyclopropylallyl)-4-bromobenzene (**1k**, 947 mg, 4.0 mmol, 1.0 equiv, see section 3.4.11 for its preparation) in anhydrous THF (20 mL), *n*-BuLi (4.4 mmol, 1.8 mL of a 2.5 M solution in *n*-hexane, 1.1 equiv) was added dropwise at –78 °C. After stirring for additional 30 min at –78 °C, chlorotriethylsilane (723 mg, 4.8 mmol, 1.2 equiv) was added. The mixture was allowed to warm to room temperature and stirred for additional 2.5 h. The mixture was quenched with water (10 mL) and saturated aqueous NH_4Cl solution (15 mL). The organic phase was separated, and the aqueous layer was extracted with EtOAc (3 × 20 mL). The combined organic phases were dried over $MgSO_4$ and concentrated under reduced pressure. Purification of the residue by flash column chromatography on silica gel using cyclohexane afforded VCP **1f** as a colorless oil (880 mg, 81% yield). **R*f*** = 0.85 (cyclohexane). **IR** (ATR): ṽ = 3010, 2952, 2909, 2874, 1599, 1458, 1415, 1394, 1237, 1104, 1016, 890, 719, 670 cm^{-1}. **^{1}H NMR** (500 MHz, $CDCl_3$, 298 K): δ = 0.46–0.42 (m, 2H), 0.57–0.63 (m, 2H), 0.79 (q, *J* = 7.9 Hz, 6H), 0.97 (t, *J* = 7.8 Hz, 9H), 1.25–1.33 (m, 1H), 3.38 (s, 2H), 4.62–4.65 (m, 1H), 4.72–4.75 (m, 1H), 7.19–7.23 (m, 2H), 7.40–7.44 (m, 2H) ppm. **^{13}C{^{1}H} NMR** (126 MHz, $CDCl_3$, 298 K):

δ = 3.6, 6.4, 7.6, 16.2, 43.0, 108.5, 128.6, 134.3, 134.7, 140.5, 150.4 ppm. **^{1}H/^{29}Si HMQC NMR** (500/99 MHz, $CDCl_3$, 298 K, optimized for J = 7 Hz): δ 0.79/1.3, 0.97/1.3, 7.42/1.3 ppm. **HRMS** (APCI): calculated for $C_{18}H_{29}Si^{\bullet+}$ $[M+H]^{\bullet+}$: 273.2039; found 273.2032.

3.4.7 (2-Cyclopropylallyl)-4-fluorobenzene (**1g**)

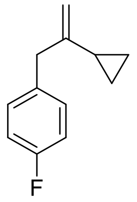

1g
$C_{12}H_{13}F$
M = 176.10 g/mol

Prepared from 4-fluorophenylacetic acid (3.1 g, 20.0 mmol) according to **GP 2**. Flash column chromatography on silica gel using cyclohexane afforded VCP **1g** as a colorless oil (282 mg, 8% yield). **R*f*** = 0.72 (cyclohexane). **IR** (ATR): ṽ = 2962, 2938, 2920, 1701, 1507, 1222, 1156, 824, 752 cm^{-1}. **^{1}H NMR** (500 MHz, $CDCl_3$, 298 K): δ = 0.39–0.46 (m, 2H), 0.56–0.64 (m, 2H), 1.21–1.30 (m, 1H), 3.35 (s, 2H), 4.61 (s, 1H), 4.73 (s, 1H), 6.94–7.02 (m, 2H), 7.13–7.22 (m, 2H) ppm. **^{13}C{^{1}H} NMR** (126 MHz, $CDCl_3$, 298 K): δ = 6.4, 16.0, 42.2, 108.6, 115.1 (d, $J_{C,F}$ = 21.2 Hz), 130.5 (d, $J_{C,F}$ = 8.0 Hz), 135.6 (d, $J_{C,F}$ = 2.8 Hz), 150.4, 161.6 (d, $J_{C,F}$ = 244.1 Hz) ppm. **^{19}F NMR** (471 MHz, $CDCl_3$, 298 K): δ = –117.6 ppm. **HRMS** (APCI): calculated for $C_{12}H_{14}F^{\bullet+}$ $[M+H]^{\bullet+}$: 177.1080; found 177.1072.

3.4.8 (2-Cyclopropylallyl)-2-fluorobenzene (**1h**)

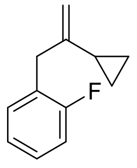

1h
$C_{12}H_{13}F$
M = 176.10 g/mol

Prepared from 4-fluorophenylacetic acid (3.1 g, 20.0 mmol) according to **GP 2**. Flash column chromatography on silica gel using cyclohexane afforded VCP **1h** as a colorless oil (300 mg, 9% yield). **R*f*** = 0.66 (cyclohexane). **^{1}H NMR** (500 MHz, $CDCl_3$, 298 K): δ = 0.42–0.48 (m, 2H), 0.59–0.65 (m, 2H), 1.29–1.37 (m, 1H), 3.40 (s, 2H), 4.58 (s, 1H), 4.74 (s, 1H), 6.98–7.05 (m, 1H), 7.05–7.10 (m, 1H), 7.16–7.25 (m, 2H) ppm. **^{13}C{^{1}H} NMR** (126 MHz, $CDCl_3$, 298 K): δ = 6.3, 16.2, 35.3 (d, $J_{C,F}$ = 3.1 Hz), 108.5, 115.3 (d, $J_{C,F}$ = 22.3 Hz), 124.0 (d, $J_{C,F}$ = 3.5 Hz), 127.0 (d, $J_{C,F}$ = 16.0 Hz), 127.9 (d, $J_{C,F}$ = 8.1 Hz), 131.4 (d, $J_{C,F}$ = 4.8 Hz), 149.2, 161.4 (d, $J_{C,F}$ = 245.7 Hz) ppm. **^{19}F NMR** (471 MHz, $CDCl_3$, 298 K): δ = –118.3 ppm. The NMR spectroscopic data are accordance with those reported.[S12]

3.4.9 (2-Cyclopropylallyl)-4-chlorobenzene (**1i**)

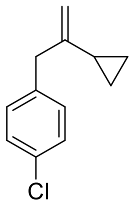

1i
$C_{12}H_{13}Cl$
M = 192.07 g/mol

Prepared from 4-chlorophenylacetic acid (3.4 g, 20.0 mmol) according to **GP 2**. Flash column chromatography on silica gel using cyclohexane afforded VCP **1i** as a colorless oil (806 mg, 21% yield). **R*f*** = 0.76 (cyclohexane). **IR** (ATR): ṽ = 3081, 3004, 2922, 2852, 1642, 1489, 1458, 1432, 1405, 1377, 1091, 1016, 891, 844, 805 cm^{-1}. **^{1}H NMR** (500 MHz, $CDCl_3$, 298 K): δ = 0.38–0.45 (m, 2H), 0.56–0.63 (m, 2H), 1.21–1.26 (m, 1H), 3.34 (s, 2H), 4.61–4.62 (m, 1H), 4.70–4.76 (m, 1H), 7.12–7.17 (m, 2H), 7.23–7.27 (m, 2H) ppm. **^{13}C{^{1}H} NMR** (126 MHz, $CDCl_3$, 298 K): δ = 6.5, 16.0, 42.4, 108.8, 128.5, 130.5, 131.9, 138.5, 150.0 ppm. **HRMS** (APCI): calculated for $C_{12}H_{14}Cl^{•+}$ $[M+H]^{•+}$: 193.0784; found 193.0784.

3.4.10 (2-Cyclopropylallyl)-2-chlorobenzene (**1j**)

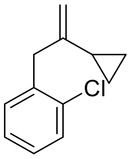

1j
$C_{12}H_{13}Cl$
M = 192.07 g/mol

Prepared from 2-chlorophenylacetic acid (3.4 g, 20.0 mmol) according to **GP 2**. Flash column chromatography on silica gel using cyclohexane afforded VCP **1j** as a colorless oil (320 mg, 8% yield). **R*f*** = 0.76 (cyclohexane). **IR** (ATR): ṽ = 3080, 3004, 2906, 1642, 1570, 1472, 1441, 1124, 1051, 1019, 891, 819, 746, 681 cm^{-1}. **^{1}H NMR** (500 MHz, $CDCl_3$, 298 K): δ = 0.47–0.50 (m, 2H), 0.63 (ddd, *J* = 8.2, 6.1, 4.0 Hz, 2H), 1.36 (tt, *J* = 8.5, 5.0 Hz, 1H), 3.49 (s, 2H), 4.49 (d, *J* = 1.7 Hz, 1H), 4.78 (s, 1H), 7.14–7.22 (m, 2H), 7.26 (dd, *J* = 7.4, 2.2 Hz, 1H), 7.36 (dd, *J* = 7.7, 1.7 Hz, 1H) ppm. **^{13}C{^{1}H} NMR** (126 MHz, $CDCl_3$, 298 K): δ = 6.3, 16.5, 39.7, 108.9, 126.7, 127.7, 129.5, 131.3, 134.6, 137.6, 148.8 ppm. **HRMS** (APCI): calculated for $C_{12}H_{14}Cl^{•+}$ $[M+H]^{•+}$: 193.0784; found 193.0779.

3.4.11 (2-Cyclopropylallyl)-4-bromobenzene (**1k**)

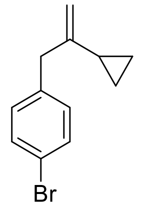

1k
$C_{12}H_{13}Br$
M = 236.02 g/mol

Prepared from 4-bromophenylacetic acid (4.3 g, 20.0 mmol) according to **GP 2**. Flash column chromatography on silica gel using cyclohexane afforded VCP **1k** as a colorless oil (975 mg, 21% yield). **R*f*** = 0.76 (cyclohexane). **IR** (ATR): ṽ = 3078, 3002, 2905, 1640, 1588, 1458, 1428, 1402, 1098, 1070, 1010, 881, 842, 791 cm^{-1}. **^{1}H NMR** (500 MHz, $CDCl_3$, 298 K): δ = 0.29–0.48 (m, 2H), 0.52–0.69 (m, 2H), 1.24 (tt, *J* = 8.3, 4.0 Hz, 1H), 3.33 (s, 2H), 4.61–4.62 (m, 1H), 4.74 (s, 1H), 6.97–7.16 (m, 2H), 7.33–7.50 (m, 2H) ppm. **^{13}C{^{1}H} NMR** (126 MHz, $CDCl_3$, 298 K): δ = 6.5, 16.0, 42.4, 108.9, 120.0, 131.0, 131.4, 139.0, 149.9 ppm. **HRMS** (APCI): calculated for $C_{12}H_{14}Br^{\bullet+}$ $[M+H]^{\bullet+}$: 237.2079, 239.0258; found 237.0275, 239.0254.

3.4.12 (2-Cyclopropylallyl)-3-methylbenzene (**1l**)

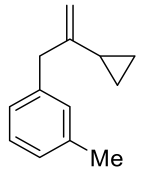

1l
$C_{13}H_{16}$
M = 172.13g/mol

Prepared from 3-methylphenylacetic acid (3.0 g, 20.0 mmol) according to **GP 1**. Flash column chromatography on silica gel using cyclohexane afforded VCP **1l** as a colorless oil (0.9 g, 27% yield). **R*f*** = 0.74 (cyclohexane). **IR** (ATR): ṽ = 3078, 3007, 2918, 2857, 1640, 1606, 1486, 1456, 1435, 1377, 1169, 1092, 1046, 1018, 879, 818, 783, 752, 696 cm^{-1}. **^{1}H NMR** (500 MHz, $CDCl_3$, 298 K): δ = 0.44–0.47 (m, 2H), 0.59–0.63 (m, 2H), 1.26–1.32 (m, 1H), 2.34 (s, 3H), 3.35 (s, 2H), 4.63 (s, 1H), 4.74 (s, 1H), 7.02–7.04 (m, 3H), 7.17–7.20 (m, 1H) ppm. **^{13}C{^{1}H} NMR** (126 MHz, $CDCl_3$, 298 K): δ = 6.4, 16.1, 21.5, 42.9, 108.4, 126.2, 126.9, 128.2, 130.0, 137.9, 139.9, 150.5 ppm. **HRMS** (APCI): calculated for $C_{13}H_{17}^{\bullet+}$ $[M+H]^{\bullet+}$: 173.1330; found 173.1327.

3.4.13 (2-Cyclopropylallyl)-3-fluorobenzene (**1m**)

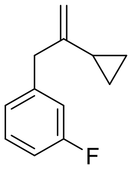

1m
$C_{12}H_{13}F$
M = 176.10 g/mol

Prepared from 4-fluorophenylacetic acid (3.1 g, 20.0 mmol) according to **GP 2**. Flash column chromatography on silica gel using cyclohexane afforded VCP **1m** as a colorless oil (600 mg, 17% yield). **R*f*** = 0.70 (cyclohexane). **IR** (ATR): ṽ = 2953, 2922, 2852, 1614, 1590, 1486, 1457, 1376, 1251, 1137, 1019, 887, 865, 784, 761, 688 cm^{-1}. **^{1}H NMR** (500 MHz, $CDCl_3$, 298 K): δ = 0.41–0.45 (m, 2H), 0.58–0.63 (m, 2H), 1.19–1.30 (m, 1H), 3.37 (s, 2H), 4.62–4.67 (m, 1H), 4.75 (s, 1H), 6.85–6.96 (m, 2H), 6.99 (d, *J* = 7.5 Hz, 1H), 7.21–7.28 (m, 1H) ppm. **^{13}C{^{1}H} NMR** (101 MHz, $CDCl_3$, 298 K): δ = 6.4, 16.0, 42.7 (d, $J_{C,F}$ = 1.3 Hz), 109.0, 113.0 (d, $J_{C,F}$ = 21.1 Hz), 116.0 (d, $J_{C,F}$ = 21.1 Hz), 124.8 (d, $J_{C,F}$ = 2.4 Hz), 129.7 (d, $J_{C,F}$ = 8.3 Hz), 142.7 (d, $J_{C,F}$ = 7.3 Hz), 149.7, 163.0 (d, $J_{C,F}$ = 246.0 Hz) ppm. **^{19}F NMR** (471 MHz, $CDCl_3$, 298 K): δ = –114.0 ppm. **HRMS** (APCI): calculated for $C_{12}H_{14}F^{\bullet+}$ $[M+H]^{\bullet+}$: 177.1080; found 177.1075.

3.4.14 (2-Cyclopropylallyl)-3-chlorobenzene (**1n**)

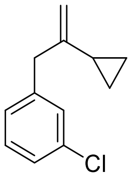

1n
$C_{12}H_{13}Cl$
M = 192.07 g/mol

Prepared from 3-chlorophenylacetic acid (3.4 g, 20.0 mmol) according to **GP 2**. Flash column chromatography on silica gel using cyclohexane afforded VCP **1n** as a colorless oil (750 mg, 20% yield). **R*f*** = 0.76 (cyclohexane). **IR** (ATR): ṽ = 3189, 3080, 3003, 2906, 2837, 1642, 1595, 1572, 1473, 1427, 1197, 1093, 1077, 1048, 1019, 885, 858, 780, 738, 718, 682 cm^{-1}. **^{1}H NMR** (500 MHz, $CDCl_3$, 298 K): δ = 0.37–0.47 (m, 2H), 0.56–0.65 (m, 2H), 1.25 (tt, *J* = 8.2, 4.0 Hz, 1H), 3.35 (s, 2H), 4.63–4.64 (m, 1H), 4.75 (s, 1H), 7.07–7.13 (m, 1H), 7.15–7.24 (m, 3H) ppm. **^{13}C{^{1}H} NMR** (126 MHz, $CDCl_3$, 298 K): δ = 6.4, 16.0, 42.7, 109.1, 126.4, 127.4, 129.3, 129.6, 134.2, 142.1, 149.7 ppm. **HRMS** (APCI): calculated for $C_{12}H_{14}Cl^{\bullet+}$ $[M+H]^{\bullet+}$: 193.0784; found 193.0782.

3.4.15 (2-Cyclopropylallyl)-3-bromobenzene (**1o**)

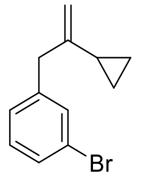

1o
$C_{12}H_{13}Br$
M = 236.02 g/mol

Prepared from 4-bromophenylacetic acid (4.3 g, 20.0 mmol) according to **GP 2**. Flash column chromatography on silica gel using cyclohexane afforded VCP **1o** as a colorless oil (488 mg, 10%). **R*f*** = 0.76 (cyclohexane). **IR** (ATR): ṽ = 3079, 3003, 2919, 2851, 1641, 1591, 1566, 1472, 1424, 1070, 1018, 996, 883, 846, 777, 689, 669 cm^{-1}. **^{1}H NMR** (500 MHz, $CDCl_3$, 298 K): δ = 0.40–0.45 (m, 2H), 0.57–0.64 (m, 2H), 1.20–1.28 (m, 1H), 3.34 (s, 2H), 4.59–4.67 (m, 1H), 4.75 (s, 1H), 7.13–7.18 (m, 2H), 7.31–7.36 (m, 1H), 7.37 (s, 1H) ppm. **^{13}C{^{1}H} NMR** (126 MHz, $CDCl_3$, 298 K): δ = 6.5, 16.0, 42.7, 109.2, 122.5, 127.9, 129.3, 129.9, 132.2, 142.5, 149.7 ppm. **HRMS** (APCI): calculated for $C_{12}H_{14}Br^{\bullet+}$ $[M+H]^{\bullet+}$: 237.0279, 239.0258; found 237.0276, 239.0254.

3.4.16 2-(2-Cyclopropylallyl)naphthalene (**1p**)

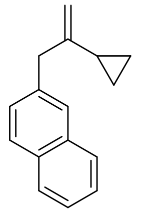

1p
$C_{16}H_{16}$
M = 208.13 g/mol

Prepared from 2-naphthylacetic acid (3.7 g, 20.0 mmol) according to **GP 3**. Flash column chromatography on silica gel using cyclohexane afforded VCP **1p** as a colorless oil (430 mg, 10% yield). **R*f*** = 0.40 (cyclohexane). **IR** (ATR): ṽ = 3077, 3049, 3004, 2921, 2853, 1632, 1598, 1506, 1428, 1363, 1268, 1047, 1017, 954, 887, 853, 813, 774, 741 cm^{-1}. **^{1}H NMR** (500 MHz, $CDCl_3$, 298 K): δ = 0.47 (ddd, *J* = 6.4, 5.3, 4.1 Hz, 2H), 0.58–0.64 (m, 2H), 1.32 (tt, *J* = 8.4, 5.3 Hz, 1H), 3.55 (s, 2H), 4.68–4.69 (m, 1H), 4.78 (dt, *J* = 1.8, 0.9 Hz, 1H), 7.37 (dd, *J* = 8.4, 1.8 Hz, 1H), 7.40–7.48 (m, 2H), 7.65–7.69 (m, 1H), 7.76–7.84 (m, 3H) ppm. **^{13}C{^{1}H} NMR** (126 MHz, $CDCl_3$, 298 K): δ = 6.5, 16.2, 43.2, 108.8, 125.4, 126.0, 127.4, 127.7, 127.8, 127.9, 127.9, 132.3, 133.8, 137.6, 150.4 ppm. **HRMS** (APCI): calculated for $C_{16}H_{17}^{\bullet+}$ $[M+H]^{\bullet+}$: 209.1330; found 209.1326.

3.4.17 1,4-Bis(2-cyclopropylallyl)benzene (**1q**)

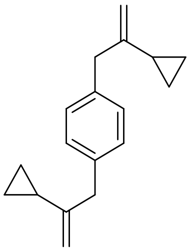

1q
$C_{18}H_{22}$
M = 238.17 g/mol

Prepared from 2-naphthylacetic acid (3.9 g, 20.0 mmol) according to **GP 3** using twice the amount of carbonyldiimidazole (**S8**) and *N*,*O*-dimethylhydroxylammonium chloride (**S2**). Flash column chromatography on silica gel using cyclohexane afforded VCP **1q** as a colorless oil (455 mg, 10% yield). **R*f*** = 0.45 (cyclohexane). **IR** (ATR): ṽ = 3079, 3004, 2902, 2831, 1641, 1510, 1429, 1238, 1100, 1047, 1018, 958, 881, 851, 818, 773 cm^{-1}. **^{1}H NMR** (500 MHz, $CDCl_3$, 298 K): δ = 0.36–0.47 (m, 4H), 0.53–0.65 (m, 4H), 1.20–1.32 (m, 2H), 3.35 (s, 4H), 4.61–4.62 (m, 2H), 4.72 (m, 2H), 7.14 (s, 4H) ppm. **^{13}C{^{1}H} NMR** (126 MHz, $CDCl_3$, 298 K): δ = 6.4, 16.1, 42.6, 108.3, 129.1, 137.6, 150.7 ppm. **HRMS** (APCI): calculated for $C_{18}H_{23}^{\bullet+}$ $[M+H]^{\bullet+}$: 239.1800; found 239.1796.

3.4.18 2-(2-Cyclopropylallyl)thiophene (**6**)

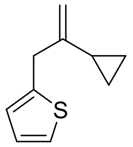

5
$C_{10}H_{12}S$
M = 164.07 g/mol

Prepared from 2-(thiophen-2-yl)acetic acid (2.8 g, 20.0 mmol) according to **GP 3**. Flash column chromatography on silica gel using cyclohexane afforded VCP **6** as a colorless oil (340 mg, 10% yield). **R*f*** = 0.50 (cyclohexane). IR (ATR): ṽ = 3079, 3003, 2901, 1643, 1433, 1230, 1038, 1018, 891, 850, 821, 692 cm^{-1}. **^{1}H NMR** (500 MHz, $CDCl_3$, 298 K): δ = 0.44–0.50 (m, 2H), 0.61–0.67 (m, 2H), 1.34 (ddd, *J* = 13.7, 8.4, 5.2 Hz, 1H), 3.58 (s, 2H), 4.75 (s, 1H), 4.76 (s, 1H), 6.85 (d, *J* = 3.2 Hz, 1H), 6.94 (dd, *J* = 5.2, 3.4 Hz, 1H), 7.15 (dd, *J* = 5.2, 1.1 Hz, 1H) ppm. **^{13}C{^{1}H} NMR** (126 MHz, $CDCl_3$, 298 K): δ = 6.6, 15.8, 37.0, 108.6, 123.8, 125.5, 126.8, 143.1, 149.9 ppm. **HRMS** (APCI): calculated for $C_{10}H_{13}S^{\bullet+}$ $[M+H]^{\bullet+}$: 165.0738; found 165.0733.

4 Experimental Details for the Trityl-Cation-Initiated Reaction of Benzyl-Substituted Vinylcyclopropanes with Hydrosilanes

4.1 General Procedure for the Trityl-Cation-Initiated Reaction of Benzyl-Substituted Vinylcyclopropanes with Hydrosilanes (GP 4)

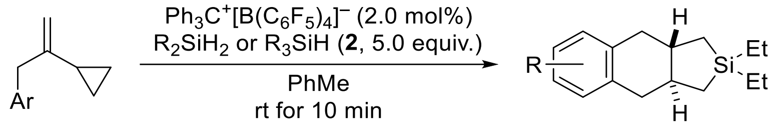

1 **3**

In a glovebox, trityl borate $Ph_3C^+[B(C_6F_5)_4]^-$ (5.5 mg, 6.0 µmol, 2.0 mol%) is suspended in toluene (0.3 mL), and the corresponding hydrosilane **2** (1.5 mmol, 5.0 equiv) is added at ambient temperature. Then, a solution of the corresponding vinylcyclopropane **1** (0.30 mmol, 1.0 equiv) in toluene (1.2 mL) is added dropwise over a period of 5 min. After complete addition, CH_2Br_2 (21.0 µL, 0.30 mmol, 1.0 equiv) is added as an internal standard to determine the yield by NMR spectroscopy. Purification by flash column chromatography on silica gel using *n*-pentane as eluent affords the corresponding hexahydrosilole **3** in analytically pure form.

4.2 Characterization Data of Products 3aa–ka, 3pa', 3qa, 3ac and 3ad

4.2.1 2,2-Diethyl-2,3,3a,4,9,9a-hexahydro-1*H*-naphtho[2,3-*c*]silole (**3aa**)

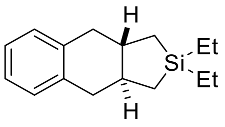

3aa
$C_{16}H_{24}Si$
M = 244.16 g/mol

Prepared from VCP **1a** (47.5 mg, 0.30 mmol, 1.0 equiv) and Et_2SiH_2 (**2a**, 132.3 mg, 1.5 mmol, 5.0 equiv) according to **GP 4** (65% NMR yield). Purification by flash column chromatography on silica gel using *n*-pentane afforded **3aa** as a colorless oil (39.5 mg, 54% yield). **R*f*** = 0.66 (cyclohexane). **IR** (ATR): ṽ = 2949, 2927, 2906, 2875, 1455, 1431, 1405, 1159, 1075, 1033, 1009, 795, 737, 708, 671 cm^{-1}. **^{1}H NMR** (500 MHz, $CDCl_3$, 298 K): δ = 0.29 (dd, *J* = 14.5, 11.4 Hz, 2H), 0.63 (q, *J* = 7.7 Hz, 4H), 1.00 (t, *J* = 7.9 Hz, 6H), 1.07–1.16 (m, 2H), 1.46–1.59 (m, 2H), 2.47–2.62 (m, 2H), 3.04–3.15 (m, 2H), 7.00–7.12 (m, 4H). **^{13}C{^{1}H} NMR** (126 MHz, $CDCl_3$, 298 K): δ = 5.4, 7.9, 18.2, 40.9, 42.0, 125.5, 128.9, 137.5. **^{1}H/^{29}Si HMQC NMR** (500/99 MHz, $CDCl_3$, 298 K, optimized for *J* = 7 Hz): δ 0.29/19.2, 0.63/19.2, 1.00/19.2, 1.12/19.2 ppm. **HRMS** (APCI): calculated for $C_{14}H_{19}Si^{\bullet+}$ $[M–CH_2CH_3]^{\bullet+}$: 215.1256; found 215.1251.

The *trans* conformation of **3aa** can be deducted from the three different couplings bigger than 10 Hz. Simulation for the compounds **3ba**, **3ga**, **3ia** and **3ka** are giving similar coupling constants, confirming the *trans* configuration in these compounds (Figure S1).

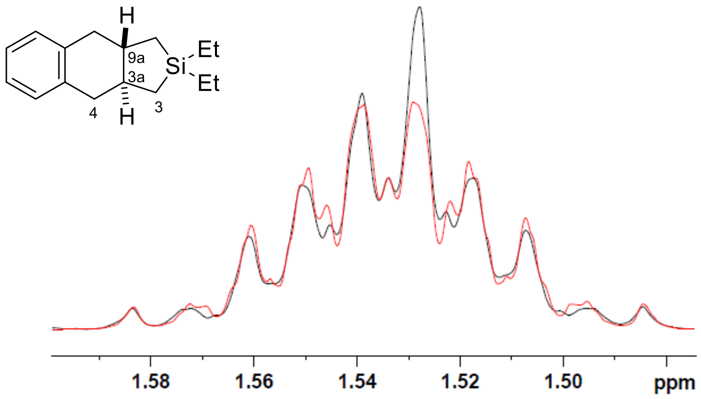

Figure S1. Experimental (black) and simulated (red) multiplet of the protons 3a/9a of the compound **3aa**. The simulated spectrum was calculated using DAISY and Topspin 3.5. Following coupling constants were used in the calculation: $^{3}J_{4,3a}$ = 5.46 Hz, $^{3}J_{4,3a}$ = 11.17 Hz, $^{3}J_{3,3a}$ = 11.75 Hz, $^{3}J_{3,3a}$ = 6.67 Hz, $^{3}J_{3a,9a}$ =11.69 Hz.

4.2.2 2,2-Diethyl-6-methyl-2,3,3a,4,9,9a-hexahydro-1*H*-naphtho[2,3-*c*]silole (**3ba**)

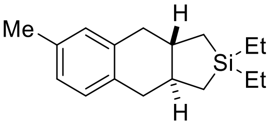

3ba
$C_{17}H_{26}Si$
M = 258.18 g/mol

Prepared from VCP **1b** (51.7 mg, 0.30 mmol, 1.0 equiv) and Et_2SiH_2 (**2a**, 132.3 mg, 1.5 mmol, 5.0 equiv) according to **GP 4** (62% NMR yield). Purification by flash column chromatography on silica gel using *n*-pentane afforded **3ba** as a colorless oil (35.0 mg, 45% yield). **R$_f$** = 0.70 (cyclohexane). **IR** (ATR): ṽ = 2949, 2925, 2906, 2875, 1501, 1458, 1431, 1405, 1160, 1071, 1010, 906, 789, 756, 733, 761 cm^{-1}. **^{1}H NMR** (500 MHz, $CDCl_3$, 298 K): δ = 0.19–0.35 (m, 2H), 0.63 (q, *J* = 7.8 Hz, 4H), 0.99 (t, *J* = 7.9 Hz, 6H), 1.09 (dd, *J* = 14.5, 5.5 Hz, 2H), 1.43–1.57 (m, 2H), 2.29 (s, 3H), 2.50 (ddd, *J* = 17.1, 10.5, 7.7 Hz, 2H), 3.05 (dt, *J* = 16.8, 4.3 Hz, 2H), 6.88 (s, 1H), 6.91 (d, *J* = 8.0 Hz, 1H), 6.96 (d, *J* = 7.7 Hz, 1H) ppm. **^{13}C{^{1}H} NMR** (126 MHz, $CDCl_3$, 298 K): δ = 5.4, 7.9, 18.2, 18.2, 21.1, 40.5, 40.9, 42.1, 42.2, 126.4, 128.7, 129.4, 134.4, 134.9, 137.4 ppm. **^{1}H/^{29}Si HMQC NMR** (500/99 MHz, $CDCl_3$, 298 K, optimized for *J* = 7 Hz): δ = 0.27/19.2, 0.63/19.2, 0.99/19.2, 1.09/19.2. **HRMS** (APCI): calculated for $C_{15}H_{21}Si^{•+}$ [M–CH_2CH_3]$^{•+}$: 229.1413; found 229.1407.

4.2.3 2,2-Diethyl-5-methyl-2,3,3a,4,9,9a-hexahydro-1*H*-naphtho[2,3-*c*]silole (**3ca**)

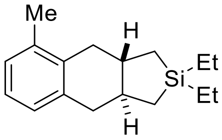

3ca
$C_{17}H_{26}Si$
M = 258.18 g/mol

Prepared from VCP **1c** (51.7 mg, 0.30 mmol, 1.0 equiv) and Et_2SiH_2 (**2a**, 132.3 mg, 1.5 mmol, 5.0 equiv) according to **GP 4** (68% NMR yield). Purification by flash column chromatography on silica gel using *n*-pentane afforded **3ca** as a colorless oil (31.0 mg, 40% yield). **R*f*** = 0.70 (cyclohexane). **IR** (ATR): ṽ = 2949, 2926, 2907, 2874, 1459, 1430, 1405, 1163, 1066, 1009, 796, 746, 705, 690, 671 cm^{-1}. **^{1}H NMR** (500 MHz, $CDCl_3$, 298 K): δ = 0.32 (ddd, *J* = 25.5, 14.5, 11.3 Hz, 2H), 0.65 (qd, *J* = 7.5, 1.3 Hz, 4H), 1.01 (td, *J* = 7.9, 2.1 Hz, 6H), 1.13 (ddd, *J* = 20.0, 14.4, 5.7 Hz, 2H), 1.45–1.59 (m, 2H), 2.24 (s, 3H), 2.29 (dd, *J* = 17.6, 9.8 Hz, 1H), 2.57 (dd, *J* = 16.7, 9.8 Hz, 1H), 2.98–3.15 (m, 2H), 6.94 (d, *J* = 7.4 Hz, 1H), 6.98 (d, *J* = 7.1 Hz, 1H), 7.00–7.05 (m, 1H) ppm. **^{13}C{^{1}H} NMR** (101 MHz, $CDCl_3$, 298 K): δ = 5.4, 5.5, 7.9, 18.0, 18.5, 19.8, 38.3, 41.4, 41.5, 42.2, 125.3, 126.7, 127.1, 136.1, 136.3, 137.5 ppm. **^{1}H/^{29}Si HMQC NMR** (500/99 MHz, $CDCl_3$, 298 K, optimized for *J* = 7 Hz): δ = 0.32/19.3, 0.65/19.3, 1.01/19.3, 1.13/19.3 ppm. **HRMS** (APCI): calculated for $C_{15}H_{21}Si^{\bullet+}$ $[M–CH_2CH_3]^{\bullet+}$: 229.1413; found 229.1407.

4.2.4 2,2-Diethyl-6-isopropyl-2,3,3a,4,9,9a-hexahydro-1*H*-naphtho[2,3-*c*]silole (**3da**)

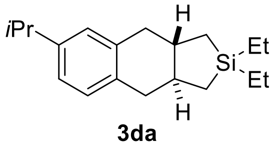

3da
$C_{19}H_{30}Si$
M = 286.21 g/mol

Prepared from VCP **1d** (60.1 mg, 0.30 mmol, 1.0 equiv) and Et_2SiH_2 (**2a**, 132.3 mg, 1.5 mmol, 5.0 equiv) according to **GP 4** (73% NMR yield). Purification by flash column chromatography on silica gel using *n*-pentane afforded **3da** as a colorless oil (39.4 mg, 46% yield). **R*f*** = 0.71 (cyclohexane). **IR** (ATR): ṽ = 2952, 2926, 2906, 2875, 1500, 1459, 1432, 1405, 1159, 1073, 1009, 907, 816, 796, 739, 716, 660 cm^{-1}. **^{1}H NMR** (500 MHz, $CDCl_3$, 298 K): δ = 0.29 (ddd, *J* = 14.6, 11.4, 1.7 Hz, 2H), 0.64 (q, *J* = 7.7 Hz, 4H), 1.00 (t, *J* = 8.0 Hz, 6H), 1.07–1.14 (m, 2H), 1.25 (d, *J* = 6.9 Hz, 6H), 1.48–1.58 (m, 2H), 2.44–2.63 (m, 2H), 2.85 (h, *J* = 7.1 Hz, 1H), 3.08 (dt, *J* = 16.8, 4.2 Hz, 2H), 6.94 (s, 1H), 6.96–7.04 (m, 2H) ppm. **^{13}C{^{1}H} NMR** (126 MHz, $CDCl_3$, 298 K): δ = 5.4, 7.9, 18.2, 24.3, 24.3, 33.9, 40.6, 41.0, 42.1, 42.1, 123.7, 126.8, 128.8, 135.0, 137.4, 146.1 ppm. **^{1}H/^{29}Si HMQC NMR** (500/99 MHz, $CDCl_3$, 298 K, optimized for *J* = 7 Hz): δ = 0.29/19.2, 0.64/19.2, 1.00/19.2, 1.10/19.2 ppm. **HRMS** (APCI): calculated for $C_{17}H_{25}Si^{\bullet+}$ $[M–CH_2CH_3]^{\bullet+}$: 257.1726; found 257.1717.

4.2.5 6-(*tert*-Butyl)-2,2-diethyl-2,3,3a,4,9,9a-hexahydro-1*H*-naphtho[2,3-*c*]silole (**3ea**)

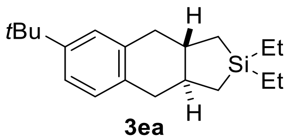

3ea
$C_{20}H_{32}Si$
M = 300.23 g/mol

Prepared from VCP **1e** (64.3 mg, 0.30 mmol, 1.0 equiv) and Et_2SiH_2 (**2a**, 132.3 mg, 1.5 mmol, 5.0 equiv) according to **GP 4**. VCP **1e** did participate in the reaction but underwent predominant de-*tert*-butylation. Purification by flash column chromatography on silica gel using *n*-pentane afforded a mixture of **3ea** and **3aa** as a colorless oil (40.1 mg, 53% yield, **3ea**:**3aa** = 18:82). The ratio was determined by ^{1}H NMR spectroscopy after purification. Attempts to separate both compounds failed. Selected NMR spectroscopic data for **3ea**: **^{1}H NMR** (500 MHz, $CDCl_3$, 298 K): δ = 1.32 (s, 9H), 7.02 (d, *J* = 8.0 Hz, 1H), 7.15 (dd, *J* = 8.0, 1.7 Hz, 1H) ppm. **^{13}C{^{1}H} NMR** (126 MHz, $CDCl_3$, 298 K): δ = 18.2, 31.6, 34.4, 40.5, 41.2, 42.1, 122.7, 125.6, 128.6, 134.6, 137.0, 148.4 ppm. **HRMS** (APCI) of **3ea**: calculated for $C_{18}H_{27}Si^{\bullet+}$ $[M–CH_2CH_3]^{\bullet+}$: 271.1882; found 271.1875. For the full characterization data of **3aa**, see section 4.2.1.

4.2.6 2,2-Diethyl-6-fluoro-2,3,3a,4,9,9a-hexahydro-1*H*-naphtho[2,3-*c*]silole (**3ga**)

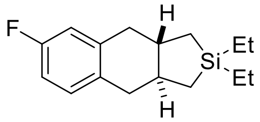

3ga
$C_{16}H_{23}FSi$
M = 262.16 g/mol

Prepared from VCP **1g** (52.9 mg, 0.30 mmol, 1.0 equiv) and Et_2SiH_2 (**2a**, 132.3 mg, 1.5 mmol, 5.0 equiv) according to **GP 4** (63% NMR yield). Purification by flash column chromatography on silica gel using *n*-pentane afforded **3ga** as a colorless oil (35.0 mg, 44% yield). **R_f** = 0.76 (cyclohexane). **IR** (ATR): ṽ = 2950, 2928, 2908, 2877, 1611, 1494, 1459, 1432, 1406, 1242, 1226, 1161, 1138, 1072, 1009, 950, 903, 857, 811, 789, 737, 710, 662 cm^{-1}. **^{1}H NMR** (500 MHz, $CDCl_3$, 298 K): δ = 0.22–0.33 (m, 2H), 0.62 (q, *J* = 7.7 Hz, 4H), 0.98 (t, *J* = 7.9 Hz, 6H), 1.09 (dd, *J* = 14.6, 5.4 Hz, 2H), 1.43–1.57 (m, 2H), 2.49 (ddd, *J* = 25.5, 16.9, 9.7 Hz, 2H), 3.05 (dt, *J* = 16.6, 4.1 Hz, 2H), 6.70–6.81 (m, 2H), 6.98 (dd, *J* = 8.3, 5.9 Hz, 1H) ppm. **^{13}C{^{1}H} NMR** (126 MHz, $CDCl_3$, 298 K): δ = 5.4, 7.9, 18.0, 18.0, 40.2, 40.9, 41.7, 42.1, 112.5 (d, $J_{C,F}$ = 21.1 Hz), 114.9 (d, $J_{C,F}$ = 20.0 Hz), 129.9 (d, $J_{C,F}$ = 7.7 Hz), 133.0 (d, $J_{C,F}$ = 2.5 Hz), 139.5 (d, $J_{C,F}$ = 6.9 Hz), 161.0 (d, $J_{C,F}$ = 243.3 Hz) ppm. **^{1}H/^{29}Si HMQC NMR** (500/99 MHz, $CDCl_3$, 298 K, optimized for *J* = 7 Hz): δ = 0.27/19.3, 0.62/19.3, 0.98/19.3, 1.09/19.3 ppm. **^{19}F NMR** (471 MHz, $CDCl_3$, 298 K): δ = –118.7 ppm. **HRMS** (APCI): calculated for $C_{14}H_{18}FSi^{\bullet+}$ $[M–CH_2CH_3]^{\bullet+}$: 233.1162; found 233.1158.

4.2.7 2,2-Diethyl-5-fluoro-2,3,3a,4,9,9a-hexahydro-1*H*-naphtho[2,3-*c*]silole (**3ha**)

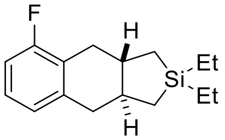

3ha
$C_{16}H_{23}FSi$
M = 262.16 g/mol

Prepared from VCP **1h** (52.9 mg, 0.30 mmol, 1.0 equiv) and Et_2SiH_2 (**2a**, 132.3 mg, 1.5 mmol, 5.0 equiv) according to **GP 4** (36% NMR yield). Purification by flash column chromatography on silica gel using *n*-pentane afforded **3ha** as a colorless oil (14.2 mg, 18% yield). **R*f*** = 0.72 (cyclohexane). **IR** (ATR): ṽ = 2950, 2927, 2909, 2876, 1577, 1459, 1432, 1238, 1166, 1077, 1060, 1037, 1012, 956, 794, 767, 746, 699, 671 cm^{-1}. **^{1}H NMR** (500 MHz, $CDCl_3$, 298 K): δ = 0.30 (ddd, *J* = 22.5, 14.5, 11.2 Hz, 2H), 0.63 (q, *J* = 7.9 Hz, 4H), 0.99 (t, *J* = 7.9 Hz, 6H), 1.12 (ddd, *J* = 20.8, 14.7, 6.0 Hz, 2H), 1.41–1.56 (m, 2H), 2.30 (dd, *J* = 17.2, 10.0 Hz, 1H), 2.52 (dd, *J* = 16.5, 10.1 Hz, 1H), 3.09 (dd, *J* = 16.6, 4.2 Hz, 1H), 3.24 (ddd, *J* = 17.1, 4.8, 1.9 Hz, 1H), 6.76–6.82 (m, 1H), 6.84 (d, *J* = 7.6 Hz, 1H), 7.01–7.07 (m, 1H) ppm. **^{13}C{^{1}H} NMR** (126 MHz, $CDCl_3$, 298 K): δ = 5.4, 5.4, 7.9, 18.0, 18.2, 33.2 (d, $J_{C,F}$ = 2.9 Hz), 40.6 (d, $J_{C,F}$ = 2.6 Hz), 41.2, 41.5, 111.8 (d, $J_{C,F}$ = 22.2 Hz), 124.1 (d, $J_{C,F}$ = 3.0 Hz), 125.0 (d, $J_{C,F}$ = 17.7 Hz), 126.3 (d, $J_{C,F}$ = 8.8 Hz), 140.3 (d, $J_{C,F}$ = 4.7 Hz), 160.9 (d, $J_{C,F}$ = 243.9 Hz) ppm. **^{1}H/^{29}Si HMQC NMR** (500/99 MHz, $CDCl_3$, 298 K, optimized for *J* = 7 Hz): δ = 0.30/19.1, 0.63/19.1, 0.99/19.1, 1.12/19.1 ppm. **^{19}F NMR** (471 MHz, $CDCl_3$, 298 K): δ = –118.5 ppm. **HRMS** (APCI): calculated for $C_{14}H_{18}FSi^{\bullet+}$ [M–CH_2CH_3]$^{\bullet+}$: 233.1162; found 233.1156.

4.2.8 6-Chloro-2,2-diethyl-2,3,3a,4,9,9a-hexahydro-1*H*-naphtho[2,3-*c*]silole (**3ia**)

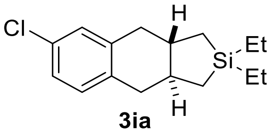

3ia
$C_{16}H_{23}ClSi$
M = 278.13 g/mol

Prepared from VCP **1i** (57.8 mg, 0.30 mmol, 1.0 equiv) and Et_2SiH_2 (**2a**, 132.3 mg, 1.5 mmol, 5.0 equiv) according to **GP 4** (54% NMR yield). Purification by flash column chromatography on silica gel using *n*-pentane afforded **3ia** as a colorless oil (36.9 mg, 44% yield). **R*f*** = 0.77 (cyclohexane). **IR** (ATR): ṽ = 2950, 2928, 2907, 2875, 1481, 1458, 1430, 1406, 1159, 1092, 1009, 892, 797, 737, 695, 655 cm^{-1}. **^{1}H NMR** (500 MHz, $CDCl_3$, 298 K): δ = 0.24–0.30 (m, 2H), 0.62 (q, *J* = 7.8 Hz, 4H), 0.98 (t, *J* = 7.9 Hz, 6H), 1.09 (dd, *J* = 14.6, 5.4 Hz, 2H), 1.42–1.54 (m, 2H), 2.44–2.53 (m, 2H), 3.05 (dd, *J* = 16.8, 3.9 Hz, 2H), 6.96 (d, *J* = 8.7 Hz, 1H), 7.03–7.05 (m, 2H) ppm. **^{13}C{^{1}H} NMR** (126 MHz, $CDCl_3$, 298 K): δ = 5.4, 7.8, 18.0, 18.0, 40.3, 40.6, 41.7, 41.9, 125.6, 128.5, 130.1, 130.9, 135.9, 139.4 ppm. **^{1}H/^{29}Si HMQC NMR** (500/99 MHz, $CDCl_3$, 298 K, optimized for *J* = 7 Hz): δ = 0.26/19.3, 0.62/19.3, 0.98/19.3, 1.09/19.3 ppm. **HRMS** (APCI): calculated for $C_{14}H_{18}ClSi^{\bullet+}$ [M–CH_2CH_3]$^{\bullet+}$: 249.0866; found 249.0860.

4.2.9 5-Chloro-2,2-diethyl-2,3,3a,4,9,9a-hexahydro-1*H*-naphtho[2,3-*c*]silole (**3ja**)

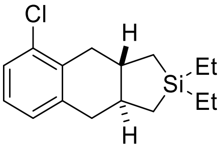

3ja
$C_{16}H_{23}ClSi$
M = 278.13 g/mol

Prepared from VCP **1j** (57.8 mg, 0.30 mmol, 1.0 equiv) and Et_2SiH_2 (**2a**, 132.3 mg, 1.5 mmol, 5.0 equiv) according to **GP 4** (38% NMR yield). Purification by flash column chromatography on silica gel using *n*-pentane afforded **3ja** as a colorless oil (15.0 mg, 18% yield). **R*f*** = 0.73 (cyclohexane). **IR** (ATR): ṽ = 2949, 2927, 2908, 2874, 1563, 1458, 1440, 1406, 1161, 1081, 1062, 1038, 1008, 794, 765, 743, 697, 667 cm^{-1}. **^{1}H NMR** (500 MHz, $CDCl_3$, 298 K): δ = 0.23–0.39 (m, 2H), 0.63 (q, *J* = 8.0 Hz, 4H), 0.98 (t, *J* = 7.9 Hz, 3H), 0.99 (t, *J* = 7.9 Hz, 3H), 1.12 (ddd, *J* = 28.7, 14.5, 5.5 Hz, 2H), 1.45–1.53 (m, 2H), 2.34 (dd, *J* = 17.7, 9.3 Hz, 1H), 2.54 (dd, *J* = 17.0, 9.7 Hz, 1H), 3.07 (dd, *J* = 16.8, 3.8 Hz, 1H), 3.30 (dd, *J* = 17.8, 4.4 Hz, 1H), 6.96 (d, *J* = 7.6 Hz, 1H), 7.00–7.04 (m, 1H), 7.16 (d, *J* = 7.7 Hz, 1H) ppm. **^{13}C{^{1}H} NMR** (126 MHz, $CDCl_3$, 298 K): δ = 5.4, 5.4, 7.9, 17.8, 18.2, 38.5, 41.2, 41.3, 41.9, 126.4, 126.5, 127.3, 134.4, 135.4, 140.0 ppm. **^{1}H/^{29}Si HMQC NMR** (500/99 MHz, $CDCl_3$, 298 K, optimized for *J* = 7 Hz): δ = 0.31/19.3, 0.63/19.3, 0.98/19.3, 0.99/19.3, 1.12/19.3 ppm. **HRMS** (APCI): calculated for $C_{16}H_{24}ClSi^{•+}$ $[M+H]^{•+}$: 279.1336; found 279.1329.

4.2.10 6-Bromo-2,2-diethyl-2,3,3a,4,9,9a-hexahydro-1*H*-naphtho[2,3-*c*]silole (**3ka**)

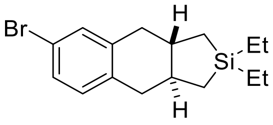

3ka
$C_{16}H_{23}BrSi$
M = 322.08 g/mol

Prepared from VCP **1k** (71.1 mg, 0.30 mmol, 1.0 equiv) and Et_2SiH_2 (**2a**, 132.3 mg, 1.5 mmol, 5.0 equiv) according to **GP 4** (72% NMR yield). Purification by flash column chromatography on silica gel using *n*-pentane afforded **3ka** as a colorless oil (48.0 mg, 50% yield). **R*f*** = 0.79 (cyclohexane). **IR** (ATR): ṽ = 2949, 2927, 2907, 2875, 1740, 1588, 1477, 1459, 1429, 1405, 1291, 1234, 1177, 1159, 1088, 1009, 905, 879, 797, 765, 711, 684 cm^{-1}. **^{1}H NMR** (500 MHz, $CDCl_3$, 298 K): δ = 0.24–0.29 (m, 2H), 0.62 (q, *J* = 7.9 Hz, 4H), 0.98 (t, *J* = 7.9 Hz, 6H), 1.09 (dd, *J* = 14.6, 5.5 Hz, 2H), 1.46–1.51 (m, 2H), 2.42–2.53 (m, 2H), 3.01–3.06 (m, 2H), 6.91 (d, *J* = 7.9 Hz, 1H), 7.16–7.20 (m, 2H) ppm. **^{13}C{^{1}H} NMR** (101 MHz, $CDCl_3$, 298 K): δ = 5.3, 7.8, 18.0, 18.0, 40.3, 40.5, 41.6, 41.8, 119.0, 128.5, 130.4, 131.5, 136.4, 139.8 ppm. **^{1}H/^{29}Si HMQC NMR** (500/99 MHz, $CDCl_3$, 298 K, optimized for *J* = 7 Hz): δ = 0.27/19.3, 0.62/19.3, 0.98/19.3, 1.09/19.3 ppm. **HRMS** (LIFDI): calculated for $C_{16}H_{23}BrSi^{•+}$ $[M]^{•+}$: 322.0752, 324.0732; found 322.0756, 324.0734.

4.2.11 2,2-Diethyl-6-methyl-2,3,3a,4,9,9a-hexahydro-1*H*-naphtho[2,3-*c*]silole (**3la**) and 2,2-Diethyl-5-methyl-2,3,3a,4,9,9a-hexahydro-1*H*-naphtho[2,3-*c*]silole (**3la'**)

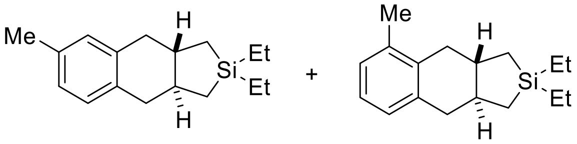

3la $C_{17}H_{26}Si$ M = 258.18 g/mol

3la' $C_{17}H_{26}Si$ M = 258.18 g/mol

Prepared from VCP **1l** (51.6 mg, 0.30 mmol, 1.0 equiv) and Et_2SiH_2 (**2a**, 132.3 mg, 1.5 mmol, 5.0 equiv) according to **GP 4** (66% NMR yield). Purification by flash column chromatography on silica gel using *n*-pentane afforded a regioisomeric mixture of **3la** and **3la'** as a colorless oil (36.4 mg, 47% yield, **3la**:**3la'** = 63:37). The regioisomeric ratio was determined by ^1H NMR spectroscopy after purification. For the full characterization data of **3la**, see section 4.2.2; for the full characterization data of **3la'**, see section 4.2.3.

4.2.12 2,2-Diethyl-6-fluoro-2,3,3a,4,9,9a-hexahydro-1*H*-naphtho[2,3-*c*]silole (**3ma**) and 2,2-Diethyl-5-fluoro-2,3,3a,4,9,9a-hexahydro-1*H*-naphtho[2,3-*c*]silole (**3ma'**)

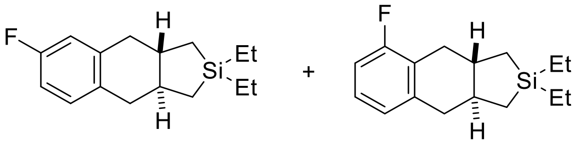

3ma $C_{16}H_{23}FSi$ M = 262.16 g/mol

3ma' $C_{16}H_{23}FSi$ M = 262.16 g/mol

Prepared from VCP **1m** (52.9 mg, 0.30 mmol, 1.0 equiv) and Et_2SiH_2 (**2a**, 132.3 mg, 1.5 mmol, 5.0 equiv) according to **GP 4** (54% NMR yield). Purification by flash column chromatography on silica gel using *n*-pentane afforded a regioisomeric mixture of **3ma** and **3ma'** as a colorless oil (20.0 mg, 25% yield, **3ma**:**3ma'** = 80:20). The regioisomeric ratio was determined by ^1H NMR spectroscopy after purification. For the full characterization data of **3ma**, see section 4.2.6; for the full characterization data of **3ma'**, see section 4.2.7.

4.2.13 6-Chloro-2,2-diethyl-2,3,3a,4,9,9a-hexahydro-1*H*-naphtho[2,3-*c*]silole (**3na**) and 5-Chloro-2,2-diethyl-2,3,3a,4,9,9a-hexahydro-1*H*-naphtho[2,3-*c*]silole (**3na'**)

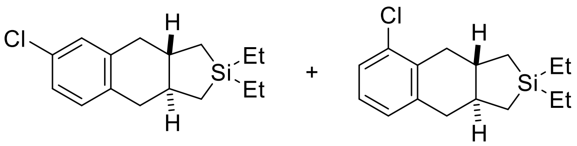

3na $C_{16}H_{23}ClSi$ M = 278.13 g/mol

3na' $C_{16}H_{23}ClSi$ M = 278.13 g/mol

Prepared from VCP **1n** (57.8 mg, 0.30 mmol, 1.0 equiv) and Et_2SiH_2 (**2a**, 132.3 mg, 1.5 mmol, 5.0 equiv) according to **GP 4** (56% NMR yield). Purification by flash column chromatography

on silica gel using *n*-pentane afforded a regioisomeric mixture of **3na** and **3na'** as a colorless oil (32.0 mg, 38% yield, **3na**:**3na'** = 69:31). The regioisomeric ratio was determined by ^{1}H NMR spectroscopy after purification. For the full characterization data of **3na**, see section 4.2.8; for the full characterization data of **3na'**, see section 4.2.9.

4.2.14 6-Bromo-2,2-diethyl-2,3,3a,4,9,9a-hexahydro-1*H*-naphtho[2,3-*c*]silole (**3oa**) and 5-Bromo-2,2-diethyl-2,3,3a,4,9,9a-hexahydro-1*H*-naphtho[2,3-*c*]silole (**3oa'**)

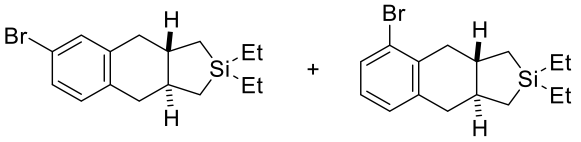

3oa $C_{16}H_{23}BrSi$ M = 322.08 g/mol

3oa' $C_{16}H_{23}BrSi$ M = 322.08 g/mol

Prepared from VCP **1o** (71.1 mg, 0.30 mmol, 1.0 equiv) and Et_2SiH_2 (**2a**, 132.3 mg, 1.5 mmol, 5.0 equiv) according to **GP 4** (80% NMR yield). Purification by flash column chromatography on silica gel using *n*-pentane afforded a regioisomeric mixture of **3oa** and **3oa'** as a colorless oil (45.0 mg, 47% yield, **3oa**:**3oa'** = 64:36). The regioisomeric ratio was determined by ^{1}H NMR spectroscopy after purification. For the full characterization data of **3oa**, see section 4.2.10.

4.2.15 2,2-Diethyl-2,3,3a,4,11,11a-hexahydro-1*H*-anthra[2,3-*c*]silole (**3pa**) and 9,9-Diethyl-7a,8,9,10,10a,11-hexahydro-7*H*-phenanthro[2,3-*c*]silole (**3pa'**)

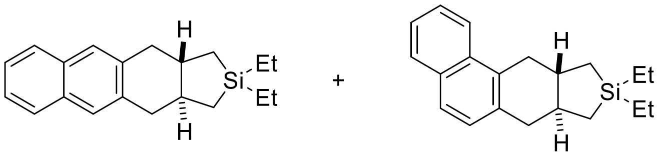

3pa $C_{20}H_{26}Si$ M = 294.18 g/mol

3pa' $C_{20}H_{26}Si$ M = 294.18 g/mol

Prepared from VCP **1p** (62.4 mg, 0.30 mmol, 1.0 equiv) and Et_2SiH_2 (**2a**, 132.3 mg, 1.5 mmol, 5.0 equiv) according to **GP 4** (62% NMR yield). Purification by flash column chromatography on silica gel using *n*-pentane afforded a regioisomeric mixture of **3pa** and **3pa'** as a colorless oil (20.0 mg, 23% yield, **3pa**:**3pa'** = 15:85). The regioisomeric ratio was determined by ^{1}H NMR spectroscopy after purification. **R*f*** = 0.58 (cyclohexane). **IR** (ATR): ṽ = 3046, 2948, 2926, 2906, 2872, 1509, 1458, 1429, 1406, 1390, 1167, 1077, 1037, 1009, 794, 762, 739, 694, 668 cm^{-1}. NMR spectroscopic data of **3pa'**: **^{1}H NMR** (500 MHz, $CDCl_3$, 298 K): δ = 0.36 (dd, *J* = 14.5, 11.2 Hz, 1H), 0.44 (dd, *J* = 14.4, 11.3 Hz, 1H), 0.66 (q, *J* = 7.9 Hz, 4H), 1.01 (t, *J* = 7.9 Hz, 3H), 1.02 (t, *J* = 7.9 Hz, 3H), 1.16 (dd, *J* = 14.4, 5.9 Hz, 1H), 1.24 (dd, *J* = 14.4, 5.8 Hz, 1H), 1.55–1.71 (m, 2H), 2.63–2.79 (m, 2H), 3.19 (dd, *J* = 16.6, 4.0 Hz, 1H), 3.63 (dd, *J* = 16.7, 4.4 Hz, 1H), 7.20 (d, *J* = 8.4 Hz, 1H), 7.39–7.45 (m, 1H), 7.49 (ddd, *J* = 8.4, 6.7, 1.4 Hz, 1H), 7.61 (d, *J* = 8.4 Hz, 1H), 7.79 (dd, *J* = 8.1, 1.3 Hz, 1H), 8.00 (d, *J* = 8.4 Hz, 1H) ppm. **^{13}C{^{1}H} NMR** (101

MHz, $CDCl_3$, 298 K): δ = 5.5, 5.5, 7.9, 18.0, 18.6, 37.3, 41.6, 41.8, 42.1, 123.2, 124.8, 125.8 (2C), 128.1, 128.5, 132.0, 132.2, 132.4, 134.8 ppm. **$^{1}H/^{29}Si$ HMQC NMR** (500/99 MHz, $CDCl_3$, 298 K, optimized for *J* = 7 Hz): δ = 0.36/19.3, 0.44/19.3, 0.66/19.3, 1.01/19.3, 1.02/19.3, 1.16/19.3, 1.24/19.3 ppm. **HRMS** (APCI): calculated for $C_{18}H_{21}Si^{•+}$ $[M–CH_2CH_3]^{•+}$: 265.1413; found 265.1404.

4.2.16 2,2,8,8-Tetraethyl-1,2,3,3a,4,6,6a,7,8,9,9a,10,12,12a-tetradecahydroanthra[2,3-*c*:6,7-*c*']bis(silole) (**3qa**)

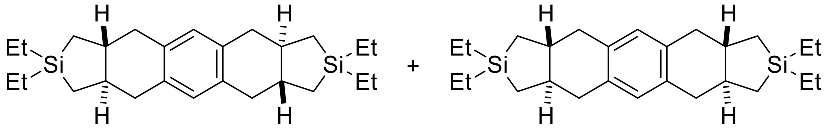

dl-**3qa**
$C_{26}H_{42}Si_2$
M = 410.79 g/mol

meso-**3qa**
$C_{26}H_{42}Si_2$
M = 410.79 g/mol

Prepared from VCP **1q** (71.5 mg, 0.30 mmol, 1.0 equiv) and Et_2SiH_2 (**2a**, 264.6 mg, 3.0 mmol, 10.0 equiv) according to **GP 4**. Purification by flash column chromatography on silica gel using *n*-pentane, followed by crystallization from a mixture of ethyl acetate and cyclohexane (1:1) afforded **3qa** as a crystalline solid (18.5 mg, 15% yield, *dl*:*meso* ≈ 50:50). **R_f** = 0.68 (cyclohexane). **M.p.** = 142.3–143.5 °C. **IR** (ATR): ṽ = 2952, 2924, 2909, 2880, 1458, 1433, 1159, 1075, 1009, 946, 797, 745, 703 cm^{-1}. **^{1}H NMR** (500 MHz, $CDCl_3$, 298 K): δ = 0.18–0.32 (m, 4H), 0.61 (q, *J* = 8.1 Hz, 8H), 0.97 (t, *J* = 7.9 Hz, 12H), 1.07 (dd, *J* = 14.6, 5.8 Hz, 4H), 1.41–1.53 (m, 4H), 2.46 (ddd, *J* = 15.5, 10.4, 4.1 Hz, 4H), 3.02 (dd, *J* = 16.3, 3.9 Hz, 4H), 6.76 (d, *J* = 1.3 Hz, 2H) ppm. **$^{13}C\{^{1}H\}$ NMR** (126 MHz, $CDCl_3$, 298 K): δ = 5.4, 7.9, 18.2, 40.56/ 40.59, 42.2, 128.79/128.82, 134.7 ppm. **$^{1}H/^{29}Si$ HMQC NMR** (500/99 MHz, $CDCl_3$, 298 K, optimized for *J* = 7 Hz): δ = 0.25/19.2, 0.61/19.2, 0.97/19.2, 1.07/19.2 ppm. **HRMS** (LIFDI): calculated for $C_{26}H_{42}Si_2^{•+}$ $[M]^{•+}$: 410.2825; found 410.2813.

Single crystals of **3qa** suitable for X-ray diffraction analysis were obtained by slow evaporation of a solution in ethyl acetate and cyclohexane (1:1). The crystal consists of a mixture of the two diastomers with a ratio of 53:47. CCDC 2034954 contains the supplementary crystallographic data (see section 7). These data are provided free of charge by The Cambridge Crystallographic Data Centre.

4.2.17 2-Ethyl-2-methyl-2,3,3a,4,9,9a-hexahydro-1*H*-naphtho[2,3-*c*]silole (**3ac**)

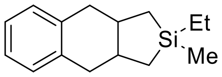

3ac

$C_{15}H_{22}Si$

M = 230.15 g/mol

Prepared from VCP **1a** (47.5 mg, 0.30 mmol, 1.0 equiv) and $EtMe_2SiH$ (**2c**, 132.3 mg, 1.5 mmol, 5.0 equiv) according to **GP 4** (42% NMR yield). Purification by flash column chromatography on silica gel using *n*-pentane afforded **3ac** as a colorless oil (15.0 mg, 22% yield). $\mathbf{R_f}$ = 0.75 (cyclohexane). **IR** (ATR): ṽ = 2949, 2926, 2906, 2880, 1486, 1452, 1431, 1402, 1248, 1159, 1074, 1033, 1010, 825, 805, 773, 739, 682 cm^{-1}. **^{1}H NMR** (500 MHz, $CDCl_3$, 298 K): δ = 0.13 (s, 2.4H), 0.16 (s, 0.6H), 0.24 (dd, *J* = 14.5, 11.2 Hz, 1H), 0.33 (dd, *J* = 14.4, 11.2 Hz, 1H), 0.62 (q, *J* = 8.0 Hz, 2H), 0.99 (t, *J* = 7.9 Hz, 3H), 1.05 (dd, *J* = 14.4, 5.9 Hz, 1H), 1.17 (dd, *J* = 14.5, 6.0 Hz, 1H), 1.48–1.61 (m, 2H), 2.55 (ddd, *J* = 15.3, 9.9, 3.4 Hz, 2H), 3.09 (ddd, *J* = 16.6, 4.5, 2.1 Hz, 2H), 7.04–7.09 (m, 4H) ppm. **^{13}C{^{1}H} NMR** (101 MHz, $CDCl_3$, 298 K): δ = –3.1, 7.3, 7.9, 20.0, 20.3, 40.8, 40.9, 41.6, 42.1, 125.5, 128.9, 137.5 ppm. **^{1}H/^{29}Si HMQC NMR** (500/99 MHz, $CDCl_3$, 298 K, optimized for *J* = 7 Hz): δ = 0.13/16.1, 0.16/16.1, 0.24/16.1, 0.33/16.1, 0.62/16.1, 0.99/16.1, 10.5/16.1, 1.17/16.1 ppm. **HRMS** (APCI): calculated for $C_{13}H_{17}Si^{\bullet+}$ $[M–CH_2CH_3]^{\bullet+}$: 201.1100; found 201.1093.

4.2.18 2,2-Dimethyl-2,3,3a,4,9,9a-hexahydro-1*H*-naphtho[2,3-*c*]silole (**3ad**)

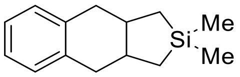

3ad

$C_{14}H_{20}Si$

M = 216.13 g/mol

Prepared from VCP **1a** (47.5 mg, 0.30 mmol, 1.0 equiv) and $PhMe_2SiH$ (**2d**, 204.4 mg, 1.5 mmol, 5.0 equiv) according to **GP 4** (60% NMR yield). Purification by flash column chromatography on silica gel using *n*-pentane afforded **3ad** as a colorless oil (16.0 mg, 25% yield). $\mathbf{R_f}$ = 0.70 (cyclohexane). **IR** (ATR): ṽ = 2951, 2925, 2885, 2854, 1450, 1431, 1246, 1158, 1073, 1031, 837, 809, 739, 721, 699, 674 cm^{-1}. **^{1}H NMR** (500 MHz, $CDCl_3$, 298 K): δ = 0.16 (s, 6H), 0.23–0.37 (m, 2H), 1.12 (dd, *J* = 14.5, 5.7 Hz, 2H), 1.51–1.61 (m, 2H), 2.43–2.64 (m, 2H), 3.08 (dd, *J* = 16.4, 4.0 Hz, 2H), 7.04–7.10 (m, 4H) ppm. **^{13}C{^{1}H} NMR** (101 MHz, $CDCl_3$, 298 K): δ = –0.9, 22.0, 40.8, 41.8, 125.5, 128.9, 137.5 ppm. **^{1}H/^{29}Si HMQC NMR** (500/99 MHz, $CDCl_3$, 298 K, optimized for *J* = 7 Hz): δ 0.16/12.9, 0.30/12.9, 1.12/12.9 ppm. **HRMS** (APCI): calculated for $C_{13}H_{17}Si^{\bullet+}$ $[M–CH_3]^{\bullet+}$: 201.1100; found 201.1094.

4.3 Characterization Data of Byproduct Triethyl(4-methyl-5-phenylpentyl)silane (4ab)

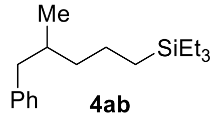

4ab

$C_{18}H_{32}Si$

M = 276.23 g/mol

Prepared from VCP **1a** (79.1 mg, 0.5 mmol, 1.0 equiv) and Et_2SiH_2 (**2a**, 220.6 mg, 5.0 equiv) using $Ph_3C^+[B(C_6F_5)_4]^-$ (9.2 mg, 0.01 mmol, 2.0 mol%) in benzene (2.5 mL) according to **GP4** (9% NMR yield, entry 1 in Table 1). Purification by flash column chromatography on silica gel using *n*-pentane afforded **4ab** as a colorless oil in low yield (5.5 mg, 4% yield). $\mathbf{R_f}$ = 0.69 (cyclohexane). **IR** (ATR): ṽ = 3027, 2952, 2911, 2874, 2101, 1494, 1455, 1073, 1012, 812, 738, 698 cm^{-1}. **^{1}H NMR** (500 MHz, $CDCl_3$, 298 K): δ = 0.42–0.53 (m, 2H), 0.50 (q, *J* = 8.0 Hz, 6H), 0.84 (d, *J* = 6.6 Hz, 3H), 0.92 (t, *J* = 7.9 Hz, 9H), 1.14–1.22 (m, 1H), 1.22–1.29 (m, 1H), 1.34–1.43 (m, 2H), 1.67–1.80 (m, 1H), 2.35 (dd, *J* = 13.4, 8.2 Hz, 1H), 2.63 (dd, *J* = 13.4, 6.0 Hz, 1H), 7.10–7.21 (m, 3H), 7.24–7.29 (m, 2H) ppm. **^{13}C{^{1}H} NMR** (126 MHz, $CDCl_3$, 298 K): δ = 3.5, 7.6, 11.6, 19.5, 21.4, 34.8, 41.2, 43.9, 125.7, 128.2, 129.3, 141.8 ppm. **^{1}H/^{29}Si HMQC NMR** (500/99 MHz, $CDCl_3$, 298 K, optimized for *J* = 7 Hz): δ = 0.47/6.4, 0.50/6.4, 0.92/6.4 ppm. **HRMS** (APCI): calculated for $C_{16}H_{27}Si^{•+}$ $[M–CH_2CH_3]^{•+}$: 247.1882; found 247.1875.

4.4 ^{1}H NMR Spectra of the Trityl-Cation-Initiated Reaction of Benzyl-Substituted Vinylcyclopropane 1a with Et_2SiH_2 (2a): Assignment of all Compounds

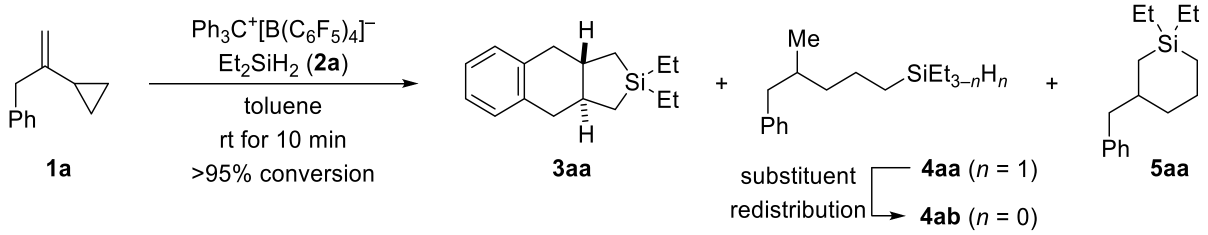

The reaction was performed according to **GP 4** with VCP **1a** (47.5 mg, 0.30 mmol, 1.0 equiv) and Et_2SiH_2 (**2a**, 132.3 mg, 1.50 mmol, 5.0 equiv) using $Ph_3C^+[B(C_6F_5)_4]^-$ (5.5 mg, 6.0 μmmol, 2.0 mol%) in toluene (1.5 mL). A sample of the reaction mixture was dissolved in C_6D_6 and directly subjected to NMR spectroscopy using CH_2Br_2 (21.0 μL, 0.30 mmol, 1.0 equiv) as an internal standard. Complete conversion of VCP **1a** was observed (Figure S2, a). After a quick fitration over silica gel, the formation of **3aa** (54%) as the main product along with **4aa** (30%)**,** **4ab** (9%) and **5aa** (7%%) was detected (Figure S2, b). Purification of the crude reaction mixture by flash column chromatography on silica gel using *n*-pentane afforded **3aa** and **4ab** in analytically pure form (Figure S2, c and e). The samples of pure **4aa** (Figure S2, d) and **5aa** (Figure S2, f) were obtained from independent experiments (see section 5.1.4 for the preparation of **4aa** and section 5.2.2 for the preparation of **5aa**).

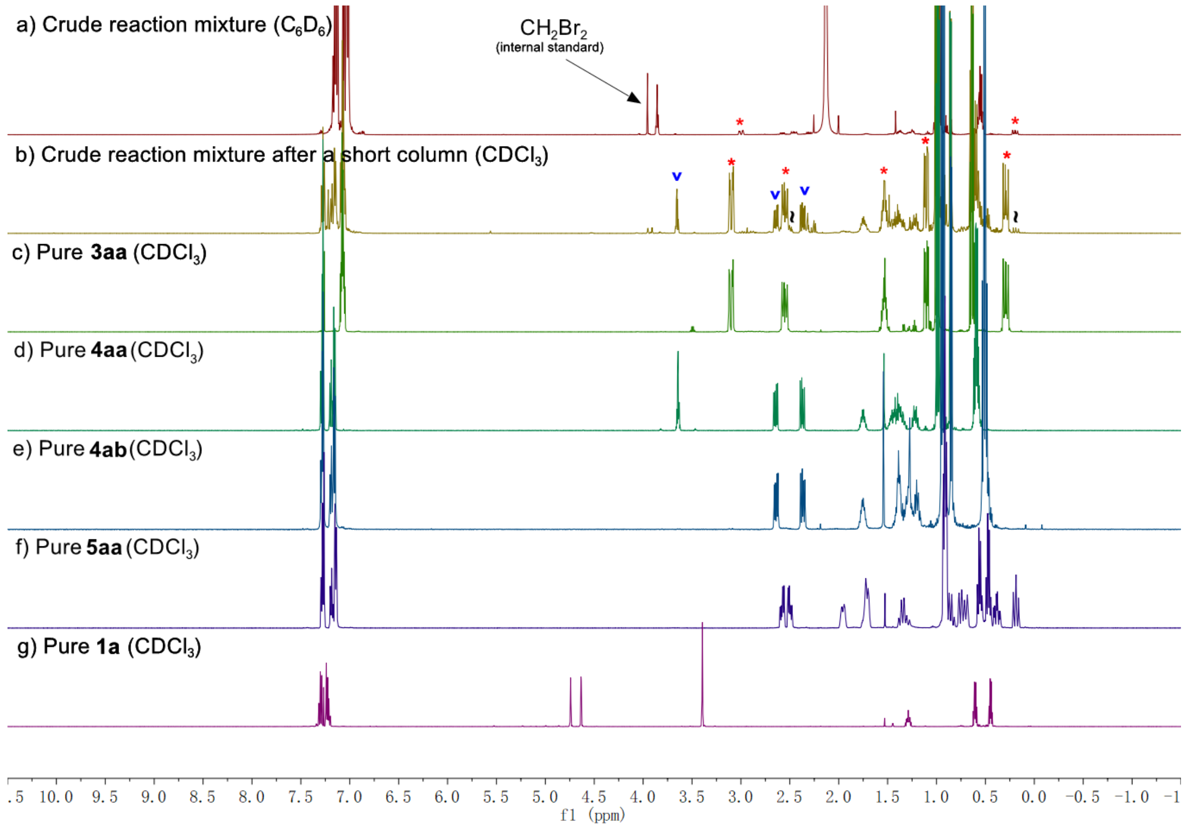

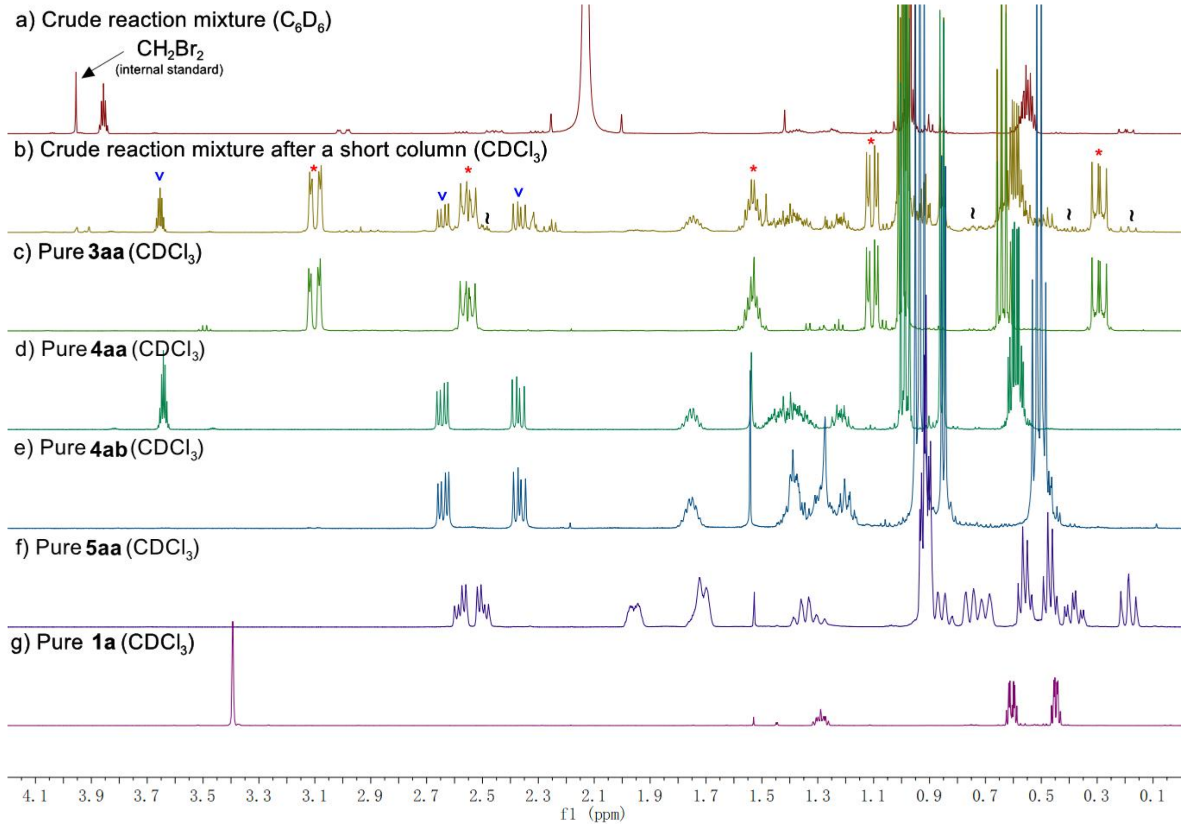

Figure S2. ^{1}H NMR spectra (500 MHz, 298 K) of the trityl-cation-initiated reaction of VCP **1a** with Et_2SiH_2 (**2a**) (top) and enlarged section of the characteristic signals in the high-field region (bottom). * for **3aa**, v for **4ab** and ≀ for **5aa**.

5 Experimental Details of the Mechanistic Control Experiments

5.1 Synthesis of Vinylcclopropane 7, Cyclization Precursors 9ab, 10aa and Byproduct 4aa

5.1.1 Synthesis of (3-Cyclopropylbut-3-en-1-yl)benzene (**7**)

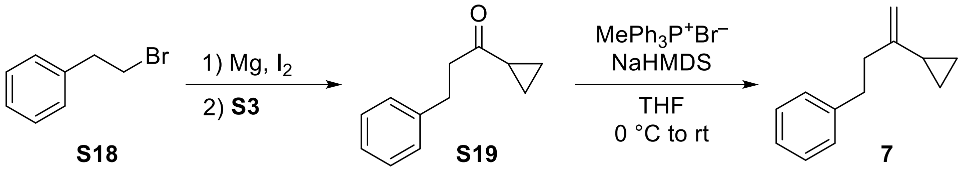

According to a reported procedure,[S13] a solution of (2-bromoethyl)benzene (**S18**, 3.7 g, 20 mmol, 1.0 equiv) in anhydrous THF (20 mL) was slowly added to a suspension of magnesium turnings (720 mg, 30 mmol, 1.5 equiv) and iodine (catalytic amounts) in anhydrous THF (5 mL) at room temperature. After complete addition, the mixture was heated to reflux for 2 h. The thus obtained solution of phenethylmagnesium bromide in THF was slowly added to a solution of Weinreb amide **S3** (2.6 g, 20 mmol, 1.0 equiv) in anhydrous THF (20 mL) at –78 °C. After complete addition, the mixture was allowed to warm to room temperature and stirred at this temperature for additional 2 h. The reaction mixture was quenched by the addition of water (10 mL) and saturated aqueous NH_4Cl solution (20 mL). The organic phase was separated, and the aqueous layer was extracted with EtOAc (3 × 20 mL). The combined organic phases were dried over $MgSO_4$ and concentrated under reduced pressure. Purification of the residue by flash column chromatography on silica gel using cyclohexane/EtOAc (20:1) afforded ketone **S19**, which was transformed into VCP **7** by a Wittig reaction according to the described procedure in section 3.1. Purification by flash column chromatography on silicon gel using cyclohexane afforded VCP **7** as a colorless oil (2.6 g, 76% yield).

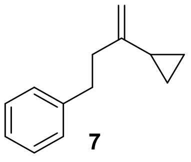

7
$C_{13}H_{16}$
M = 172.13 g/mol

R*f* = 0.68 (cyclohexane). **IR** (ATR): ṽ = 3080, 3024, 3002, 2931, 2857, 1640, 1602, 1494, 1453, 1429, 1018, 878, 818, 746, 697 cm^{-1}. **^{1}H NMR** (500 MHz, $CDCl_3$, 298 K): δ = 0.41–0.50 (m, 2H), 0.62–0.70 (m, 2H), 1.31–1.39 (m, 1H), 2.28–2.40 (m, 2H), 2.75–2.86 (m, 2H), 4.66–4.67 (m, 1H), 4.67–4.68 (m, 1H), 7.16–7.23 (m, 3H), 7.26–7.31 (m, 2H) ppm. **^{13}C{^{1}H} NMR** (126 MHz, $CDCl_3$, 298 K): δ = 6.2, 16.3, 34.9, 37.9, 106.6, 125.9, 128.4, 128.5, 142.5, 150.7 ppm. **HRMS** (APCI): calculated for $C_{13}H_{17}^{\bullet+}$ [M+H]$^{\bullet+}$: 173.1330; found 173.1324.

5.1.2 Synthesis of Cyclization Precursor Triethyl(4-phenylpent-4-en-1-yl)silane (**9ab**)

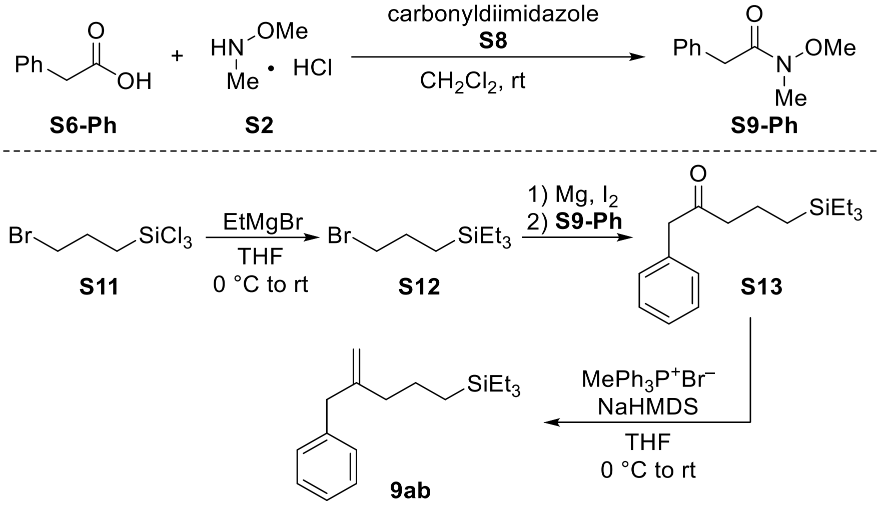

Scheme S5. Synthesis of Cyclization Precursor **9ab**.

N-Methoxy-*N*-methyl-2-phenylacetamide (**S9-Ph**)

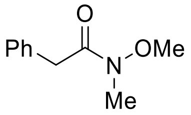

S9-Ph
$C_{10}H_{13}NO_2$
M = 179.09 g/mol

According to **GP 3**, carbonyldiimidazole (**S8**, 4.2 g, 26 mmol, 1.3 equiv) was added to a solution of phenylacetic acid (**S6-Ph**, 2.7 g, 20 mmol, 1.0 equiv) in anhydrous CH_2Cl_2 (50 mL) at ambient temperature. After stirring for 1 h, *N*,*O*-dimethylhydroxylammonium chloride (**S2**, 3.9 g, 40 mmol, 2.0 equiv) was added, and the reaction mixture was stirred overnight at ambient temperature. Water (20 mL) and saturated aqueous NH_4Cl solution (20 mL) was added to the reaction mixture, and the organic phase was separated. The aqueous layer was extracted with CH_2Cl_2 (2 × 30 mL), and the combined organic phases were washed with brine (30 mL), dried over $MgSO_4$ and concentrated under reduced pressure. Purification of the residue by flash column chromatography on silicon gel using cyclohexane/EtOAc (1:1) afforded the Weinreb amide **S9-Ph** as a pale yellow oil (3.5 g, 97% yield). **^{1}H NMR** (500 MHz, $CDCl_3$, 298 K): δ = 3.19 (s, 3H), 3.60 (s, 3H), 3.78 (s, 2H), 7.21–7.33 (m, 5H) ppm. The NMR spectroscopic data are in accordance with those reported.[S14]

(3-Bromopropyl)triethylsilane (**S12**)

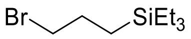

S12
$C_9H_{21}BrSi$
M = 237.26 g/mol

To a solution of (3-bromopropyl)trichlorosilane (**S11**, 5.1 g, 20 mmol, 1.0 equiv) in anhydrous THF (20 mL), ethylmagnesium bromide (22 mL of a 3 M solution in Et_2O, 66 mmol, 3.3 equiv) was added dropwise at 0 °C. The reaction mixture was stirred at room temperature for 2 h and then carefully quenched with water (20 mL) and aqueous HCl solution (1 N, 20 mL) at 0 °C. The organic phase was separated, and the aqueous layer was extracted with Et_2O (3 × 30 mL). The combined organic phases were dried over $MgSO_4$ and concentrated under reduced pressure to afford silane **S12** as a colorless oil (4.3 g, 90% yield), which was directly used in the next step without further purification. **^{1}H NMR** (500 MHz, $CDCl_3$, 298 K): δ = 0.52 (q, J = 8.0 Hz, 6H), 0.60–0.65 (m, 2H), 0.93 (t, J = 8.0 Hz, 9H), 1.80–1.88 (m, 2H), 3.38 (t, J = 7.1 Hz, 2H) ppm. **^{13}C{^{1}H} NMR** (126 MHz, $CDCl_3$, 298 K): δ = 3.4, 7.5, 10.9, 28.1, 37.5 ppm. **^{1}H/^{29}Si HMQC NMR** (500/99 MHz, $CDCl_3$, 298 K, optimized for J = 7 Hz): δ = 0.52/6.5, 0.63/6.5, 0.93/6.5 ppm. The NMR spectroscopic data are in accordance with those reported.[S5]

1-Phenyl-5-(triethylsilyl)pentan-2-one (**S13**)

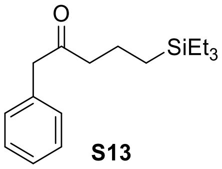

S13
$C_{17}H_{28}OSi$
M = 276.19 g/mol

To a suspension of magnesium turnings (648 mg, 27.0 mmol, 1.5 equiv) and iodine (catalytic amounts) in anhydrous THF (5 mL), a solution of (3-bromopropyl)triethylsilane (**S12**, 4.3 g, 18.0 mmol, 1.0 equiv) in anhydrous THF (25 mL) was slowly added at room temperature. After complete addition, the mixture was heated to reflux for 2 h. The thus obtained Grignard reagent was added dropwise to a solution of Weinreb amide **S9-Ph** (3.2g, 18 mmol, 1.0 equiv) in anhydrous THF (15 mL) at –78 °C. After complete addition, the mixture was allowed to slowly warm to ambient temperature and stirred at this temperature for additional 2 h. The reaction mixture was then quenched with water (10 mL) and saturated aqueous NH_4Cl solution (10 mL). The organic phase was separated, and the aqueous layer was extracted with EtOAc (3 × 30 mL). The combined organic phases were dried over $MgSO_4$ and concentrated under reduced pressure. Purification of the residue by flash column chromatography on silica gel using cyclohexane/EtOAc (20:1) afforded ketone **S13** as a pale yellow oil (1.42 g, 26% yield). **R_f** = 0.60 (cyclohexane/EtOAc 10:1). **^{1}H NMR** (500 MHz, $CDCl_3$, 298 K): δ = 0.42–0.45 (m, 2H), 0.48 (q, J = 8.0 Hz, 6H), 0.90 (t, J = 7.9 Hz, 9H), 1.52–1.58 (m, 2H), 2.47 (t, J = 7.2 Hz, 2H), 3.67 (s, 2H), 7.19–7.22 (m, 2H), 7.24–7.28 (m, 1H), 7.31–7.35 (m, 2H) ppm. **^{13}C{^{1}H} NMR** (126 MHz, $CDCl_3$, 298 K): δ = 3.3, 7.5, 11.4, 27.1, 46.1, 50.4, 127.1, 128.8, 129.6, 134.5, 208.7 ppm. **^{1}H/^{29}Si HMQC NMR** (500/99 MHz, $CDCl_3$, 298 K, optimized for J = 7 Hz): δ = 0.43/6.4, 0.48/6.4, 0.90/6.4 ppm.

(4-Benzylpent-4-en-1-yl)triethylsilane (**9ab**)

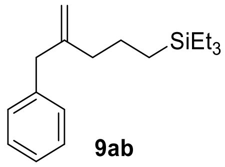

9ab
$C_{18}H_{30}Si$
M = 274.21 g/mol

For the reaction procedure of the Wittig reaction to afford alkene **9ab** from ketone **S13**, see section 3.1. Purification by flash column chromatography on silicon gel using cyclohexane as eluent afforded **9ab** as a colorless oil (820 mg, 58% yield). **R*f*** = 0.75 (cyclohexane). **IR** (ATR): ṽ = 3063, 3026, 2950, 2908, 2873, 1493, 1454, 1415, 1237, 1013, 891, 753, 723, 698 cm^{-1}. **^{1}H NMR** (500 MHz, $CDCl_3$): δ = 0.45–0.50 (m, 2H), 0.48 (q, *J* = 7.9 Hz, 6H), 0.92 (t, *J* = 8.0 Hz, 9H), 1.39–1.47 (m, 2H), 1.99 (t, *J* = 7.5 Hz, 2H), 3.32 (s, 2H), 4.74 (s, 1H), 4.81 (s, 1H), 7.16–7.22 (m, 3H), 7.26–7.31 (m, 2H) ppm. **^{13}C{^{1}H} NMR** (126 MHz, $CDCl_3$, 298 K): δ = 3.5, 7.6, 11.2, 22.1, 39.8, 43.1, 111.3, 126.1, 128.4, 129.2, 140.1, 149.2 ppm. **^{1}H/^{29}Si HMQC NMR** (500/99 MHz, $CDCl_3$, 298 K, optimized for *J* = 7 Hz): δ = 0.47/6.4, 0.48/6.4, 0.92/6.4 ppm. **HRMS** (LIFDI): calculated for $C_{18}H_{30}Si^{•+}$ $[M]^{•+}$: 274.2117; found 274.2117.

5.1.3 Synthesis of (2-Cyclopropyl-3-phenylpropyl)diethylsilane (**10aa**)

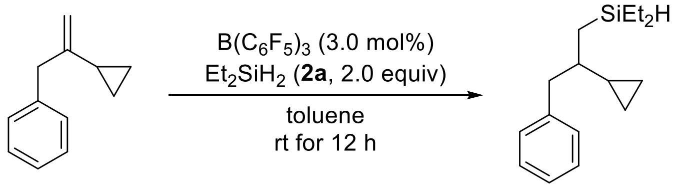

Scheme S6. Synthesis of Cyclization Precursor **10aa**.

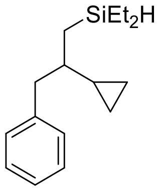

10aa
$C_{16}H_{26}Si$
M = 246.18 g/mol

According to a reported procedure,[S11] diethylsilane (**2a**, 212 mg, 2.4 mmol, 1.2 equiv) and benzyl-substituted VCP **1a** (316.5 mg, 2.0 mmol, 1.0 equiv) were added to a solution of $B(C_6F_5)_3$ (30.7 mg, 0.06 mmol, 3 mol%) in anhydrous toluene (10 mL). The reaction mixture was stirred at room temperature for 12 h. Purification by flash column chromatography on silica gel using *n*-pentane as eluent afforded hydrosilane **10aa** as a colorless oil (118 mg, 24% yield). **^{1}H NMR** (500 MHz, $CDCl_3$, 298 K): δ = –0.11 – –0.03 (m, 1H), 0.05–0.13 (m, 1H), 0.33–0.45 (m, 2H), 0.54–0.63 (m, 5H), 0.72 (qdd, *J* = 14.8, 6.8, 3.5 Hz, 2H), 0.93 (t, *J* = 7.8 Hz, 3H), 0.96 (t, *J* = 8.0 Hz, 3H), 1.06 (dp, *J* = 9.2, 6.7 Hz, 1H), 2.69 (dd, *J* = 13.2, 6.9 Hz, 1H), 2.74 (dd, *J* =

13.2, 6.4 Hz, 1H), 3.80 (quint, J = 3.2 Hz, 1H), 7.15–7.20 (m, 3H), 7.23–7.28 (m, 2H) ppm. **^{13}C{^{1}H} NMR** (126 NHz, $CDCl_3$, 298 K): δ = 3.4, 3.5, 4.8, 5.0, 8.2, 8.3, 16.4, 18.4, 42.8, 44.1, 125.6, 127.9, 129.5 141.2 ppm. **^{1}H/^{29}Si HMQC NMR** (500/99 MHz, $CDCl_3$, 298 K, optimized for J = 7 Hz): δ = 0.59/–4.5, 0.72/–4.5, 0.93/–4.5, 0.96/–4.5, 1.06/–4.5 ppm. The NMR spectroscopic data are in accordance with those reported.[S11]

5.1.4 Synthesis of Byproduct Diethyl(4-methyl-5-phenylpentyl)silane (**4aa**)

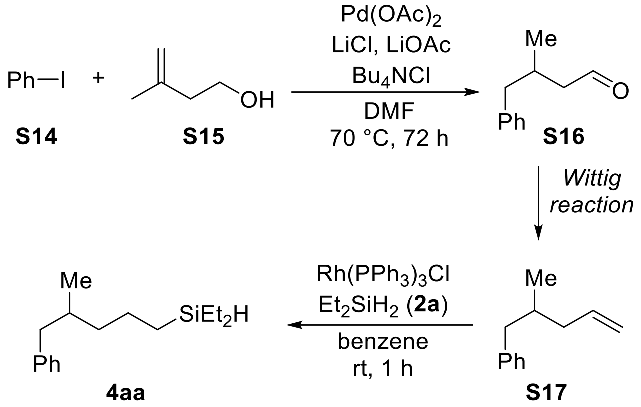

Scheme S7. Synthesis of Byproduct **4aa**.

3-Methyl-4-phenylbutanal (**S16**)

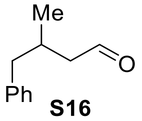

S16

$C_{11}H_{14}O$

M = 162.10 g/mol

According to a reported procedure,[S15] iodobenzene (**S14**, 2.0 g, 10 mmol, 1.0 equiv) and 3-methyl-3-buten-1-ol (**S15**, 861 mg, 10 mmol, 1.0 equiv) were added to a suspension of $Pd(OAc)_2$ (67 mg, 0.3 mmol, 3.0 mol%), Bu_4NCl (5.5 g, 20 mmol, 2.0 equiv), LiCl (424 mg, 10 mmol, 1.0 equiv) and LiOAc (1.7 g, 25 mmol, 2.5 equiv) in dry DMF (20 mL). The reaction mixture was stirred for 72 h at 70 °C. The reaction was then quenched with saturated aqueous NH_4Cl solution (15 mL) and extracted with EtOAc (3 × 20 mL). The combined organic phases were dried over $MgSO_4$ and concentrated under reduced pressure. Purification of the residue by flash column chromatography on silica gel using *n*-pentane to *n*-pentane/EtOAc (10:1) afforded aldehyde **S16** as a pale yellow oil (410 mg, 25% yield). **^{1}H NMR** (500 MHz, $CDCl_3$, 298 K): δ = 0.99 (d, J = 6.7 Hz, 3H), 2.25 (ddd, J = 15.7, 7.5, 2.5 Hz, 1H), 2.32–2.47 (m, 2H), 2.52–2.64 (m, 2H), 7.12–7.18 (m, 2H), 7.18–7.24 (m, 1H), 7.27–7.33 (m, 2H), 9.71 (dd, J = 2.5, 1.6 Hz, 1H) ppm. The NMR spectroscopic data are in accordance with those reported.[S16]

(2-Methylpent-4-en-1-yl)benzene (**S17**)

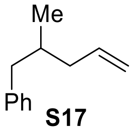

S17

$C_{12}H_{16}$

M = 160.13 g/mol

For the reaction procedure of the Wittig reaction to afford alkene **S17** from aldehyde **S16**, see section 3.1. Purification by flash column chromatography on silicon gel using *n*-pentane as eluent afforded **S17** as a colorless oil (200 mg, 50% yield). **^{1}H NMR** (500 MHz, $CDCl_3$, 298 K): δ = 0.87 (d, *J* = 6.6 Hz, 3H), 1.77–1.88 (m, 1H), 1.93 (dtt, *J* = 13.7, 7.5, 1.2 Hz, 1H), 2.12 (dddt, *J* = 13.7, 6.9, 5.5, 1.4 Hz, 1H), 2.39 (dd, *J* = 13.4, 8.1 Hz, 1H), 2.67 (dd, *J* = 13.4, 6.1 Hz, 1H), 4.97–5.10 (m, 2H), 5.76–5.87 (m, 1H), 7.13–7.23 (m, 3H), 7.24–7.31 (m, 2H) ppm. **^{13}C{^{1}H} NMR** (126 MHz, $CDCl_3$, 298 K): δ = 19.3, 35.1, 41.1, 43.2, 116.1, 125.8, 128.3, 129.3, 137.5, 141.5 ppm. The NMR spectroscopic data are in accordance with those reported.[S17]

Diethyl(4-methyl-5-phenylpentyl)silane (**4aa**)

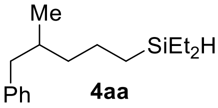

4aa

$C_{16}H_{28}Si$

M = 248.20 g/mol

According to a reported procedure,[S18] Wilkinson's catalyst $Rh(PPh_3)_3Cl$ (11.6 mg, 12.5 µmol, 1.0 mol%) was suspended in benzene (2.0 mL), and (2-methylpent-4-en-1-yl)benzene (**S17**, 200 mg, 1.25 mmol, 1.0 equiv) was added at ambient temperature. Then, diethylsilane (**2a**, 220.6 mg, 2.5 mmol, 2.0 equiv) was added, and the reaction mixture was stirred at ambient temperature for additional 1 h. The solvent was removed under reduced pressure, and the residue was purified by flash column chromatography on silica gel using *n*-pentane as eluent, to afford hydrosilane **4aa** as a colorless oil (247 mg, 80% yield). **R_f** = 0.68 (cyclohexane). **IR** (ATR): ṽ = 3062, 3026, 2952, 2912, 2872, 2095, 1454, 1013, 968, 810, 737, 698 cm^{-1}. **^{1}H NMR** (500 MHz, $CDCl_3$, 298 K): δ = 0.52–0.63 (m, 6H), 0.84 (d, *J* = 6.7 Hz, 3H), 0.97 (t, *J* = 7.9 Hz, 6H), 1.15–1.25 (m, 1H), 1.28–1.50 (m, 3H), 1.67–1.82 (m, 1H), 2.36 (dd, *J* = 13.4, 8.2 Hz, 1H), 2.63 (dd, *J* = 13.3, 6.0 Hz, 1H), 3.63 (h, *J* = 3.2 Hz, 1H), 7.11–7.21 (m, 3H), 7.24–7.30 (m, 2H) ppm. **^{13}C{^{1}H} NMR** (126 MHz, $CDCl_3$, 298 K): δ = 3.0, 8.4, 10.9, 19.5, 22.2, 34.9, 40.6, 43.9, 125.7, 128.2, 129.3, 141.8 ppm. **^{1}H/^{29}Si HMQC NMR** (500/99 MHz, $CDCl_3$, 298 K, optimized for *J* = 7 Hz): δ = 0.57/–2.2, 0.97/–2.2, 1.39/–2.2 ppm. **^{1}H/^{29}Si HMQC NMR** (500/99 MHz, $CDCl_3$, 298 K, optimized for *J* = 200 Hz): δ = 3.63/–2.2 ppm. **HRMS** (APCI): calculated for $C_{14}H_{23}Si^{•+}$ $[M–CH_2CH_3]^{•+}$: 219.1569; found 219.1569.

5.2 Control Experiments with VCP 7, Precursors 9ab, 10aa and Byproduct 4aa

5.2.1 Control Experiment with VCP **7** and Et_2SiH_2 (**2a**)

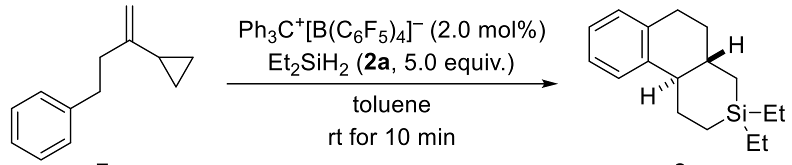

7 **8a**

Trityl borate $Ph_3C^+[B(C_6F_5)_4]^-$ (5.5 mg, 6.0 µmol, 2.0 mol%) was suspended in toluene (0.3 mL), and Et_2SiH_2 (**2a**, 132.3 mg, 1.5 mmol, 5.0 equiv) was added at ambient temperature. Then, a solution of VCP **7** (51.6 mg, 0.30 mmol, 1.0 equiv) in toluene (1.2 mL) was added dropwise over a period of 5 min. After complete addition, CH_2Br_2 (21.0 µL, 0.30 mmol, 1.0 equiv) was added as an internal standard to determine the yield by NMR spectroscopy (78% NMR yield). Purification by flash column chromatography on silica gel using *n*-pentane as eluent afforded **8a** as a colorless oil (50.0 mg, 43% yield).

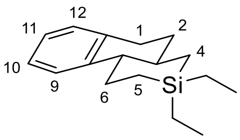

8a
$C_{17}H_{26}Si$
M =258.18 g/mol

R$_f$ = 0.70 (cyclohexane). **IR** (ATR): ṽ = 3058, 3015, 2948, 2907, 2871, 1487, 1451, 1411, 1231, 1153, 1068, 1007, 959, 921, 891, 858, 842, 785, 767, 734, 696 cm^{-1}. **^{1}H NMR** (500 MHz, $CDCl_3$, 298 K): δ = 0.53 (q, ^3J = 8.0 Hz, 2H, $SiCH_2CH_3$), 0.54 (dd, $^2J_{4ax,4eq}$ = 14.2 Hz, $^3J_{4ax,3ax}$ = 12.7 Hz, 1H, H-4ax), 0.64 (q, ^3J = 8.0 Hz, 2H, $SiCH_2CH_3$), 0.75 (ddd, $^2J_{5ax,5eq}$ = 14.2 Hz, $^3J_{5ax,6ax}$ = 14.2 Hz, $^3J_{5ax,6eq}$ = 5.5 Hz, 1H, H-5ax), 0.88 (ddd, $^2J_{4eq,4ax}$ = 14.2 Hz, $^3J_{4eq,3ax}$ = 2.1 Hz, $^4J_{4eq,5eq}$ = 2.1 Hz, 1H, H-4eq), 0.97 (dddd, $^2J_{5eq,5ax}$ = 14.4 Hz, $^3J_{5eq,6eq}$ = 3.8 Hz, $^3J_{5eq,6ax}$ = 2.8 Hz, $^4J_{5eq,4eq}$ = 1.5 Hz, 1H, H-5eq), 0.97 (t, ^3J = 8.0 Hz, 3H, $SiCH_2CH_3$), 1.00 (t, ^3J = 8.0 Hz, 3H, $SiCH_2CH_3$), 1.44 (dddd, $^2J_{6ax,6eq}$ = 14.0 Hz, $^3J_{6ax,7ax}$ = 11.5 Hz, $^3J_{6ax,5ax}$ = 14.0 Hz, $^3J_{6ax,5eq}$ = 3.2 Hz, 1H, H-6ax), 1.62–1.48 (m, 2H, H-2a, H-3ax), 1.77 (dddd, $^2J_{2eq,2ax}$ = 12.4 Hz, $^3J_{2eq,1ax}$ = 4.9 Hz, $^3J_{2eq,1eq}$ = 2.2 Hz, $^3J_{2eq,3ax}$ = 2.2 Hz, 1H, H-2eq), 2.25 (dd, $^3J_{7ax,3ax}$ = 10.6 Hz, $^3J_{7ax,6ax}$ = 10.6 Hz, 1H, H-7ax), 2.64 (dddd, $^2J_{6eq,6ax}$ = 14.0 Hz, $^3J_{6eq,5ax}$ = 5.5 Hz, $^3J_{6eq,5eq}$ = 3.9 Hz, $^3J_{6eq,7ax}$ = 2.5 Hz, 1H, H-6eq), 2.72 (ddd, $^2J_{1eq,1ax}$ = 16.3 Hz, $^3J_{1eq,2ax}$ = 4.2 Hz, $^3J_{1eq,2eq}$ = 2.5 Hz , 1H, H-1eq), 2.87 (ddd, $^2J_{1ax,1eq}$ = 16.3 Hz, $^3J_{1ax,2eq}$ = 12.8 Hz, $^3J_{1ax,2eq}$ = 4.9 Hz, 1H, H-1ax), 7.04–7.10 (m, 2H, H-11, H-12), 7.14 (t, ^3J = 8.0 Hz, 1H, H-10), 7.33 (d, $^3J_{9,10}$ = 7.9 Hz, 1H, H-9) ppm. **^{13}C{^{1}H} NMR** (126 MHz, $CDCl_3$, 298 K): δ = 3.1, 5.0, 7.4, 7.7, 11.1, 19.9, 31.3, 32.3, 35.9, 39.9, 47.3, 125.3, 125.8, 127.8, 129.1, 137.9, 141.0 ppm. **^{1}H/^{29}Si HMQC NMR** (500/99 MHz, $CDCl_3$, 298 K, optimized for J = 7 Hz): δ = 0.54/0.5, 0.64/0.5, 0.75/0.5, 0.88/0.5, 0.94/0.5, 0.97/0.5, 1.00/0.5 ppm. **HRMS** (APCI): calculated for $C_{15}H_{21}Si^{\bullet+}$ $[M–CH_2CH_3]^{\bullet+}$: 229.1413; found 229.1406.

5.2.2 Control Experiment with Cyclization Precursor **9ab** and Et_3SiH (**2b**)

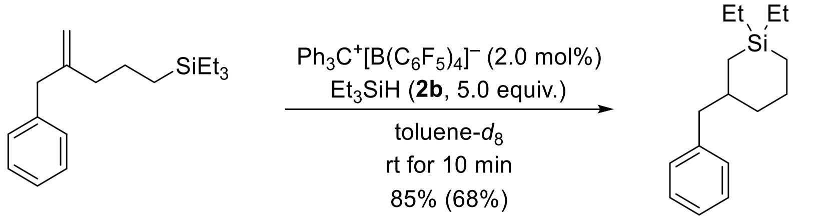

9ab **5aa**

Trityl borate $Ph_3C^+[B(C_6F_5)_4]^-$ (5.5 mg, 6.0 µmol, 2.0 mol%) was suspended in toluene-d_8 (0.3 mL), and Et_3SiH (**2b**, 174.4 mg, 1.5 mol, 5.0 equiv) was added at ambient temperature. Then, a solution of precursor **9ab** (82.4 mg, 0.30 mmol, 1.0 equiv) in toluene-d_8 (1.2 mL) was added dropwise over a period of 5 min. After complete addition, CH_2Br_2 (21.0 µL, 0.30 mmol, 1.0 equiv) was added as an internal standard, and the reaction mixture was directly subjected to NMR spectroscopic analysis, revealing the formation of 3-benzyl-1,1-diethylsilinane (**5aa**, 85% NMR yield). No formation of **3aa** was observed. Purification by flash column chromatography on silica gel using *n*-pentane afforded silinane **5aa** as a colorless oil (50.1 mg, 68% yield).

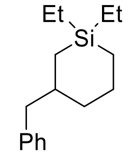

5aa

$C_{16}H_{26}Si$

M = 246.18 g/mol

R$_f$ = 0.70 (cyclohexane). **IR** (ATR): ṽ = 3025, 2950, 2905, 2872, 1493, 1453, 1412, 1232, 1192, 1071, 1008, 778, 761, 727, 696 cm^{-1}. **^{1}H NMR** (500 MHz, $CDCl_3$, 298 K): δ = 0.18 (ddd, *J* = 14.4, 12.6, 1.7 Hz, 1H), 0.38 (tdd, *J* = 13.9, 5.5, 1.7 Hz, 1H), 0.43–0.51 (m, 2H), 0.51–0.62 (m, 2H), 0.65–0.72 (m, 1H), 0.72–0.79 (m, 1H), 0.80–0.89 (m, 1H), 0.88–0.95 (m, 6H), 1.28–1.41 (m, 1H), 1.65–1.78 (m, 2H), 1.90–2.00 (m, 1H), 2.49 (dd, *J* = 13.3, 6.8 Hz, 1H), 2.58 (dd, *J* = 13.3, 6.8 Hz, 1H), 7.10–7.16 (m, 2H), 7.16–7.21 (m, 1H), 7.25–7.31 (m, 2H) ppm. **^{13}C{^{1}H} NMR** (126 MHz, $CDCl_3$, 298 K): δ = 3.0, 5.1, 7.4, 7.6, 9.8, 17.4, 23.9, 36.3, 38.5, 48.3, 125.7, 128.1, 129.4, 141.5 ppm. **^{1}H/^{29}Si HMQC NMR** (500/99 MHz, $CDCl_3$, 298 K, optimized for *J* = 7 Hz): δ = 0.18/1.1, 0.38/1.1, 0.47/1.1, 0.57/1.1, 0.68/1.1, 0.76/1.1, 0.85/1.1 0.92/1.1ppm. **HRMS** (APCI): calculated for $C_{14}H_{21}Si^{\bullet+}$ $[M–CH_2CH_3]^{\bullet+}$: 217.1413; found 217.1403.

5.2.3 Control Experiment with Cyclization Precursor **10aa** without Hydrosilane

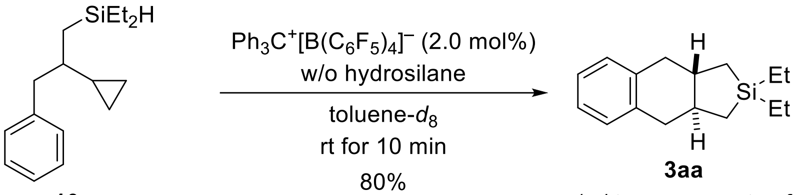

10aa **3aa**

(w/ trace amounts of **4aa/4ab** and/or **5aa**)

Trityl borate $Ph_3C^+[B(C_6F_5)_4]^-$ (1.8 mg, 2.0 µmol, 2.0 mol%) was suspended in toluene-*d*$_8$ (0.10 mL). Then, a solution of hydrosilane **10aa** (24.7 mg, 0.10 mmol, 1.0 equiv) in toluene-*d*$_8$ (0.4 mL) was added dropwise over a period of 5 min. After complete addition, CH_2Br_2 (7.0 µL, 0.30 mmol, 1.0 equiv) was added as an internal standard, and the reaction mixture was directly subjected to NMR spectroscopic analysis, revealing the formation of **3aa** (80% NMR yield) along with trace amounts of byproducts **4aa**/**4ab** and/or **5aa**. For full characterization data of **3aa**, see section 3.2.1.

5.2.4 Control Experiments with Byproduct **4aa**

5.2.4.1 Control Experiment with **4aa** without Hydrosilane

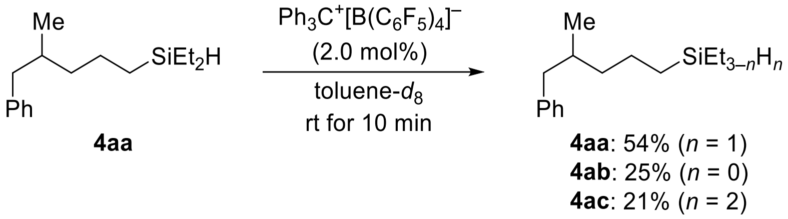

In a glovebox, trityl borate $Ph_3C^+[B(C_6F_5)_4]^-$ (1.8 mg, 2.0 µmol, 2.0 mol%) was suspended in toluene-*d*$_8$ (0.10 mL). Then, a solution of **4aa** (24.8 mg, 0.10 mmol, 1.0 equiv) in toluene-*d*$_8$ (0.40 mL) was added dropwise over a period of 5 min. After complete addition, CH_2Br_2 (7.0 µL, 0.10 mmol, 1.0 equiv) was added as an internal standard, and the reaction mixture was directly subjected to NMR spectroscopic analysis. Filtration over a short column using *n*-pentane afforded a mixture of **4aa**, **4ab** and **4ac** (20.8 mg). The ratio was determined by ^{1}H NMR specscopy. For full characterization data of **4aa**, see section 4.1.4; for full characterization data of **4ab**, see section 3.3. The presence of **4ac** was determined by GC-MS and ^{1}H NMR spectroscopy (see Figure S68) as well as ^{1}H,^{29}Si-HMQC analysis (see Figures S69 and S70).

5.2.4.2 Control Experiment with **4aa** with Hydrosilane

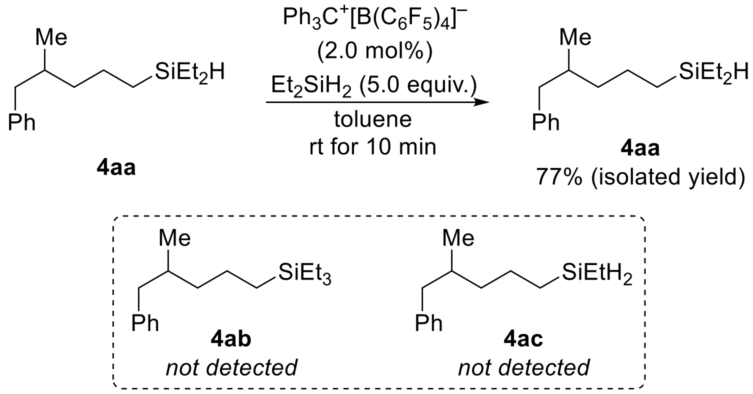

In a glovebox, trityl borate $Ph_3C^+[B(C_6F_5)_4]^-$ (1.8 mg, 2.0 µmol, 2.0 mol%) was suspended in toluene-*d*$_8$ (0.10 mL), and Et_2SiH_2 (**2b**, 44.1 mg, 0.50 mol, 5.0 equiv) was added at ambient temperature. Then, a solution of **4aa** (24.8 mg, 0.10 mmol, 1.0 equiv) in toluene-*d*$_8$ (0.40 mL)

was added dropwise over a period of 5 min. After complete addition, CH_2Br_2 (7.0 µL, 0.10 mmol, 1.0 equiv) was added as an internal standard, and the reaction mixture was directly subjected to NMR spectroscopic analysis. Filtration over a short column using *n*-pentane afforded **4aa** (19.2 mg, 77% yield). No formation of **4ab** and **4ac** was detected.

5.3 Mechanistic Control Experiments with Deuterium-Labeled Et_3SiD (2b-d_1) and Vinylcyclopropane 1a-d_1

5.3.1 Mechanistic Control Experiment with VCP **1a** and Deuterium-Labeled Et_3SiD (**2b**-d_1)

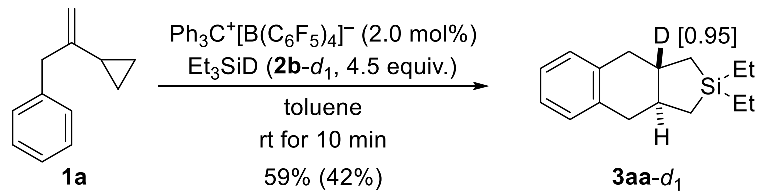

In a glovebox, trityl borate $Ph_3C^+[B(C_6F_5)_4]^-$ (5.5 mg, 6.0 µmol, 2.0 mol%) was suspended in toluene (0.30 mL), and Et_3SiD[S5] (**2b**-d_1, 175 mg, 1.5 mmol, 5.0 equiv) was added at ambient temperature. Then, a solution of VCP **1a** (47.5 mg, 0.30 mmol, 1.0 equiv) in toluene (1.2 mL) was added dropwise over a period of 5 min. After complete addition, CH_2Br_2 (21.0 µL, 0.30 mmol, 1.0 equiv) was added as an internal standard, and the reaction mixture was directly subjected to NMR spectroscopic analysis, revealing the formation of deuterated **3aa**-d_1 (59% NMR yield). Purification by flash column chromatography on silica gel using *n*-pentane as eluent afforded **3aa**-d_1 as a colorless oil (31.2 mg, 42% yield). The deuterium incorporation in the C3 position was determined by ^1H and ^2H NMR spectroscopy. For full characterization data of non-deuterated **3aa**, see section 3.2.1.

5.3.2 Mechanistic Control Experiment with VCP **1a**-d_1 and Et_2SiH_2 (**2a**)

5.3.2.1 Synthesis of Deuterium-Labeled Benzyl-Substituted VCP **1a**-d_1

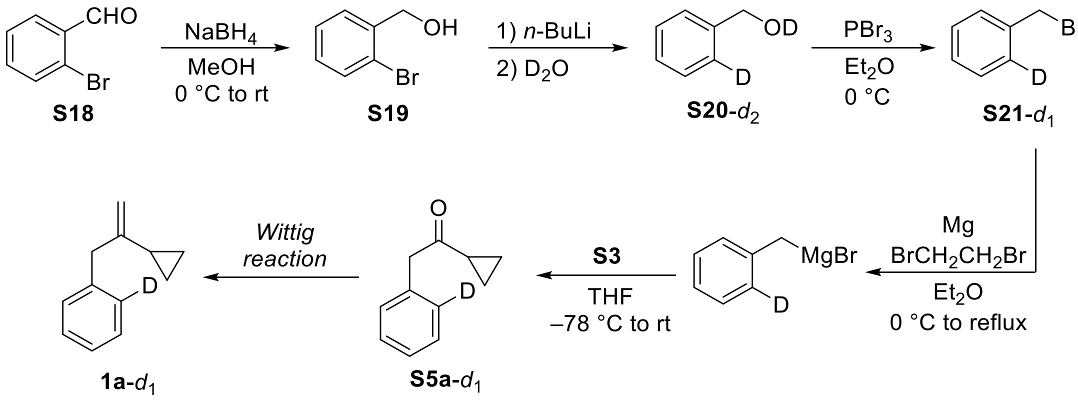

Scheme S8. Synthesis of Deuterium-Labeled Benzyl-Substituted VCP **1a**-d_1.

2-Bromobenzyl alcohol (**S19**)

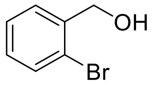

S19
C_7H_7BrO
M = 185.97 g/mol

According to a reported procedure,[S19] sodium borohydride (0.91 g, 24 mmol, 1.2 equiv) was added portionwise to a solution of 2-bromobenzaldehyde (**S18**, 3.7 g, 20 mmol, 1.0 equiv) in methanol (20 mL) at 0 °C. The reaction mixture was stirred at 0 °C for 1 h and then allowed to warm to room temperature overnight. The reaction mixture was quenched with saturated aqueous NH_4Cl solution (30 mL) and aqueous HCl (1 N, 20 mL) and then extracted with Et_2O (3 × 30 mL). The combined organic phases were dried over $MgSO_4$ and concentrated under reduced pressure to afford 2-bromobenzyl alcohol (**S19**) as a pale yellow solid (3.5 g, 95% yield), which was directly used in the next step without further purification.

Deuterium-labeled benzyl alcohol (**S20**-*d*$_2$)

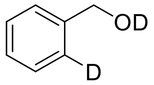

S20-*d*$_2$
$C_7H_6D_2O$
M = 110.07 g/mol

According to a reported procedure,[S20] *n*-BuLi (30.4 mL of a 2.5 M solution in *n*-hexane, 76 mmol, 4.0 equiv) was added dropwise to a solution of 2-bromobenzyl alcohol (**S19**, 3.5 g, 19 mmol, 1.0 equiv) in anhydrous THF (30 mL) at –78 °C. The mixture was stirred at this temperature for additional 1 h and then carefully quenched by the dropwise addition of deuterium oxide (10 mL). The reaction mixture was stirred at –78 °C for additional 30 min and then allowed to warm to room temperature. The mixture was extracted with Et_2O (3 × 30 mL), dried over $MgSO_4$ and concentrated under reduced pressure. The residue was purified by flash chromatography on silica gel using cyclohexane/Et_2O (10:1) as eluent to afford **S20**-*d*$_2$ as a colorless oil (1.8 g, 86% yield).

Deuterium-labeled benzyl bromide (**S21**-*d*$_1$)

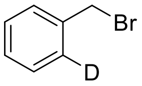

S21-*d*$_1$
C_7H_6DBr
M = 170.98 g/mol

Caution: *Benzyl bromide is a volatile liquid and a strong lachrymator, also intensely irritating to skin*. According to a reported procedure,[S21] a solution of phosphorus tribromide (1.5 mL, 16.4 mmol, 1.0 equiv) in anhydrous Et_2O (30 mL) was stirred at 0 °C for 10 min. Then, a

solution of deuterium-labeled benzyl alcohol (**S20**-*d*$_2$, 1.8 g, 16.4 mmol) was added dropwise at 0 °C. The reaction mixture was stirred for additional 1 h at 0 °C and then carefully quenched by the addition of saturated aqueous $NaHCO_3$ solution (30 mL). The mixture was extracted with Et_2O (3 × 20 mL), dried over $MgSO_4$ and concentrated under reduced pressure. The residue was purified by flash column chromatography using *n*-pentane as eluent to afford deuterium-labeled benzyl bromide **S21**-*d*$_1$ as a pale yellow oil (2.3 g, 82% yield).

1-Cyclopropyl-2-(phenyl-2-*d*)ethan-1-one (**S5a**-*d*$_1$)

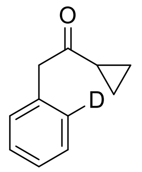

S5a-*d*$_1$
$C_{11}H_{11}DO$
M = 161.10 g/mol

According to a reported procedure,[S7] a solution of deuterated benzyl bromide (**S21**-*d*$_1$, 2.3 g, 13.5 mmol, 1.0 equiv) in anhydrous Et_2O (40 mL) was added dropwise to a suspension of magnesium turnings (486 mg, 20.3 mmol, 1.5 equiv) and 1,2-dibromoethane (30 µL) in anhydrous Et_2O (10 mL) at 0 °C. After complete addition, the mixture was heated at 50 °C for 2 h. The thus obtained Grignard reagent was added dropwise to a solution of Weinreb amide **S3** (1.8 g, 21.6 mmol, 1.0 equiv) in anhydrous THF (20 mL) at –78 °C. After complete addition, the mixture was allowed to warm to room temperature and stirred for additional 2 h. The reaction mixture was then quenched with water (20 mL) and saturated aqueous NH_4Cl solution (20 mL). The organic phase was separated, and the aqueous layer was extracted with EtOAc (3 × 30 mL). The combined organic phases were dried over $MgSO_4$ and concentrated under reduced pressure. Purification of the residue by flash column chromatography on silica gel using cyclohexane/EtOAc (20:1) as eluent afforded deuterium-labeled ketone **S5a**-*d*$_1$ as a pale yellow oil (1.6 g, 74% yield). **R*f*** = 0.60 (cyclohexane/EtOAc 10:1).

Deuterium-labeled benzyl-substituted VCP **1a**-*d*$_1$

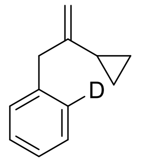

1a-*d*$_1$
$C_{12}H_{13}D$
M = 159.12 g/mol

For the reaction procedure of the Wittig reaction to afford VCP **1a**-*d*$_1$ from ketone **S5a**-*d*$_1$, see section 3.1. Purification by flash column chromatography on silicon gel using cyclohexane as eluent afforded VCP **1a**-*d*$_1$ as a colorless oil (990 mg, 70% yield). **R*f*** = 0.71 (cyclohexane). **^{1}H**

NMR (500 MHz, $CDCl_3$, 298 K): δ = 0.42–0.46 (m, 2H), 0.58–0.62 (m, 2H), 1.28 (dddd, *J* = 13.7, 8.3, 5.3, 1.0 Hz, 1H), 3.39 (s, 2H), 4.62–4.63 (m, 1H), 4.73–4.74 (m, 1H), 7.19–7.23 (m, 2H), 7.28–7.31 (m, 2H) ppm. **^{13}C{^{1}H} NMR** (126 MHz, $CDCl_3$, 298 K): δ = 6.4, 16.1, 43.0, 108.5, 126.1, 128.3, 128.4, 129.2, 140.0, 150.5 ppm. For full characterization data of non-deuterated VCP **1a**, see section 3.4.1.

5.3.2.2 Mechanistic Control Experiment with Deuterium-Labeled VCP **1a**-d_1

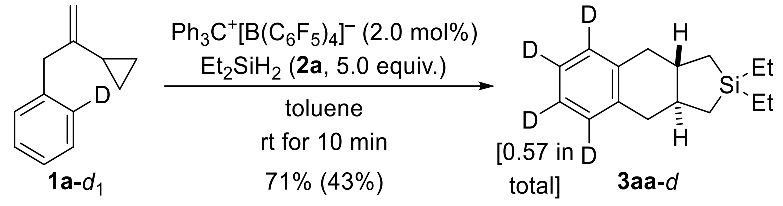

In a glovebox, trityl borate $Ph_3C^+[B(C_6F_5)_4]^-$ (5.5 mg, 6.0 μmol, 2.0 mol%) was suspended in toluene (0.3 mL), and Et_2SiH_2 (**2a**, 132.3 mg, 1.5 mmol, 5.0 equiv) was added at ambient temperature. Then, a solution of deuterium-labeled VCP **1a**-d_1 (47.8 mg, 0.30 mmol, 1.0 equiv) in toluene (1.2 mL) was added dropwise over a period of 5 min. After complete addition, CH_2Br_2 (21.0 μL, 0.30 mmol, 1.0 equiv) was added as an internal standard, and the reaction mixture was directly subjected to NMR spectroscopic analysis, revealing the formation of deuterated **3aa**-*d* (71% NMR yield). Purification by flash column chromatography on silica gel using *n*-pentane as eluent afforded **3aa**-*d* as a colorless oil (31.2 mg, 43% yield). The deuterium incorporation was determined by ^{1}H NMR, ^{2}H NMR and ^{13}C{^{1}H} NMR spectroscopy. For full characterization data of non-deuterated **3aa**, see section 4.2.1.

6 NMR Spectra

Figure S3. **^{1}H NMR** spectrum (500 MHz, C_6D_5Cl, 298 K) of $Et_3Si^+[CHB_{11}H_5Br_6]^-$ from the reaction of Et_3SiH with $Ph_3C^+[CHB_{11}H_5Br_6]^-$.

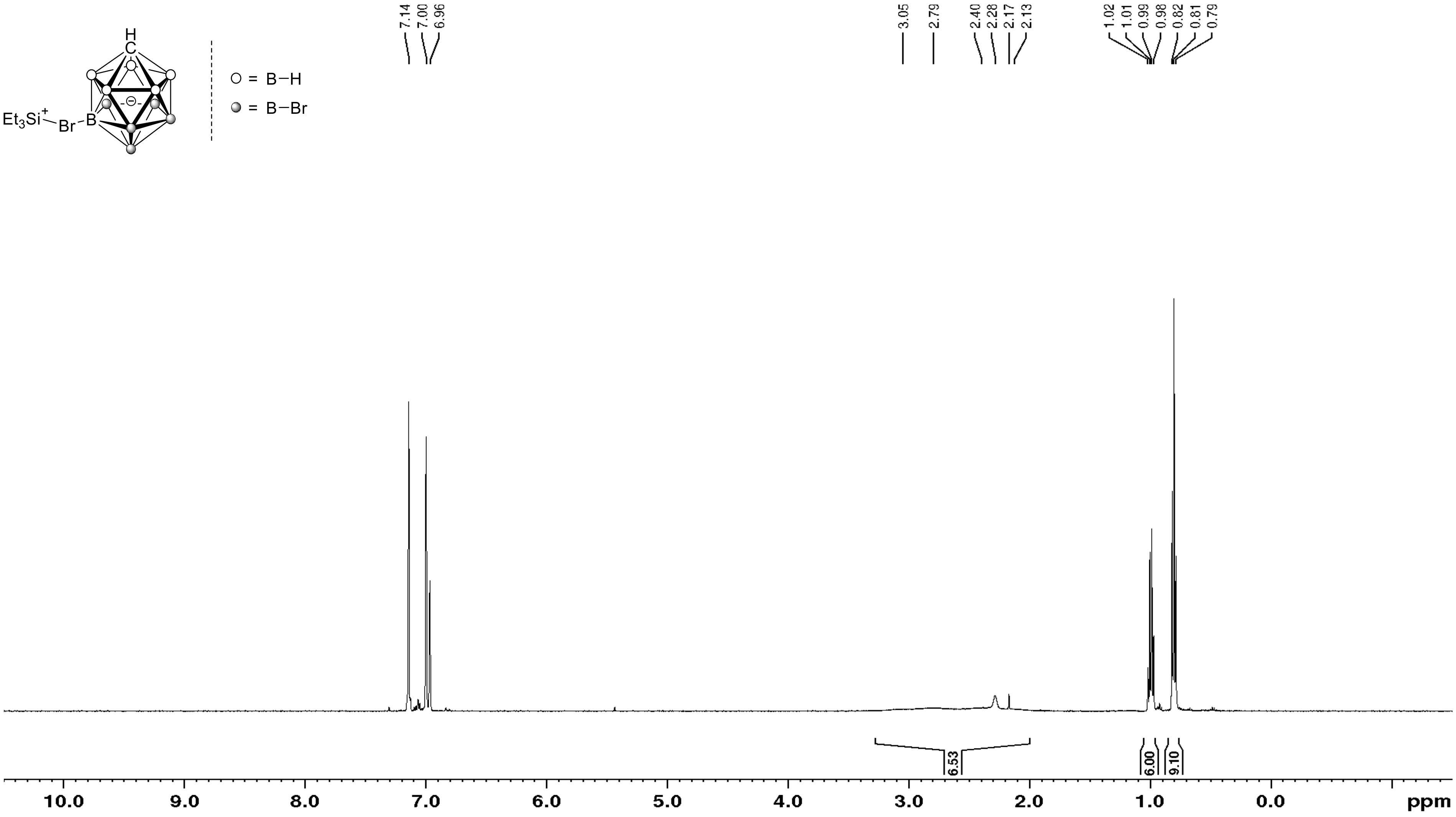

Figure S4. **^{11}B NMR** spectrum (161 MHz, C_6D_5Cl, 298 K) of $Et_3Si^+[CHB_{11}H_5Br_6]^-$ from the reaction of Et_3SiH with $Ph_3C^+[CHB_{11}H_5Br_6]^-$.

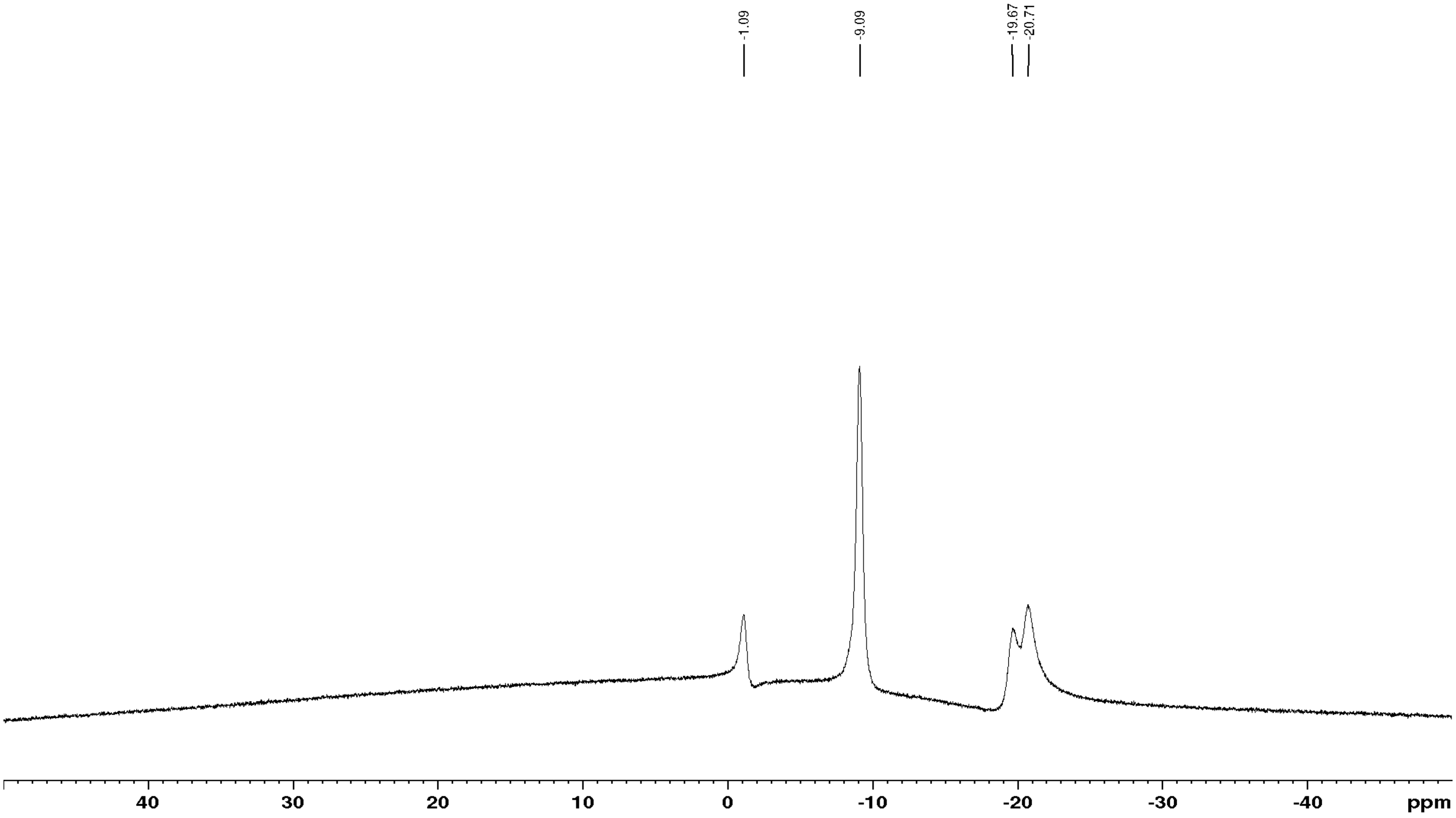

Figure S5. **$^{13}C\{^{1}H\}$ NMR** spectrum (126 MHz, C_6D_5Cl, 298 K) of $Et_3Si^+[CHB_{11}H_5Br_6]^-$ from the reaction of Et_3SiH with $Ph_3C^+[CHB_{11}H_5Br_6]^-$.

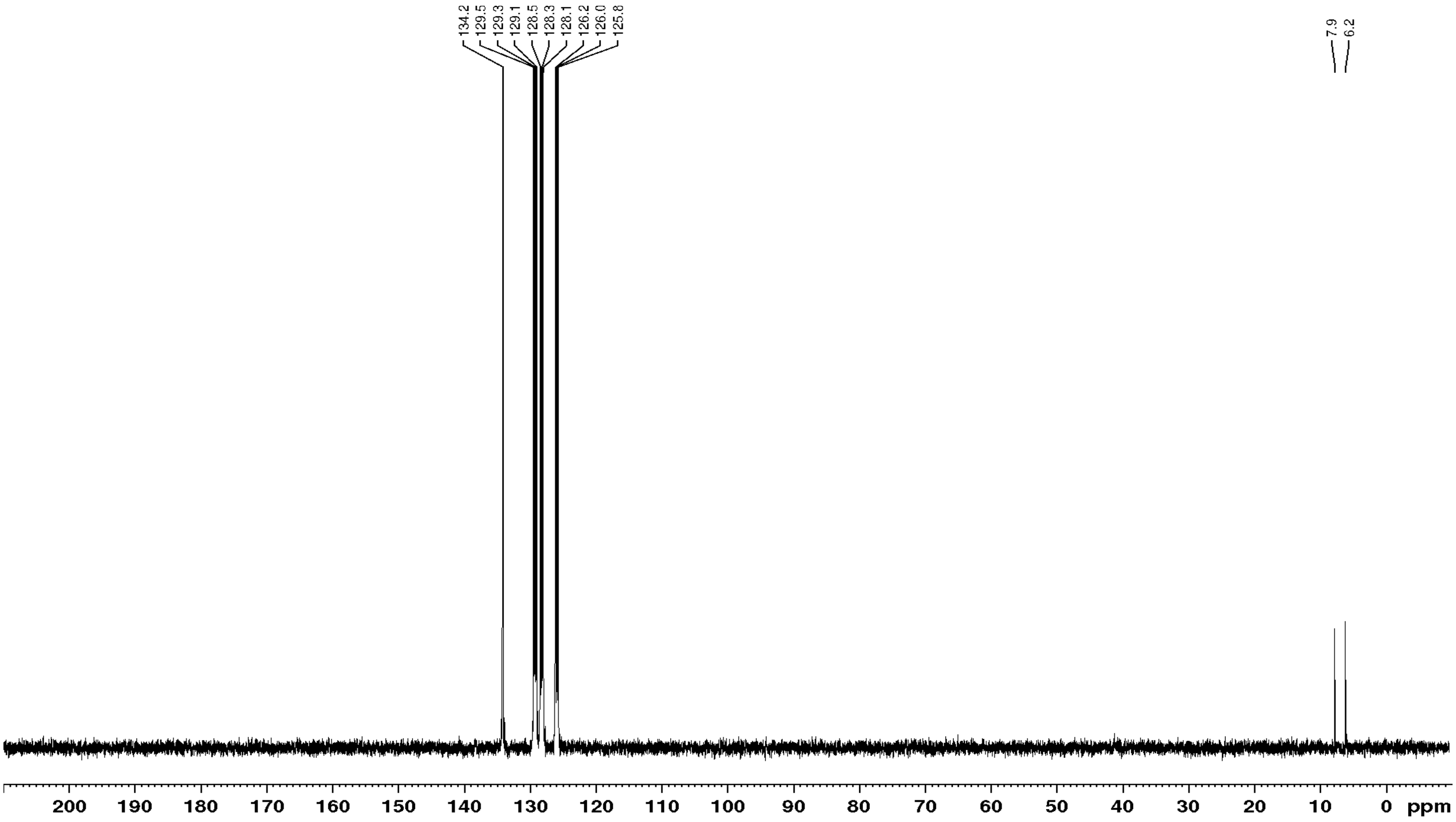

Figure S6 **$^{1}H/^{29}Si$ HMQC NMR** spectrum (500/99 MHz, CD_2Cl_2, 298 K, optimized for *J* = 7 Hz) of $Et_3Si^+[CHB_{11}H_5Br_6]^-$ from the reaction of Et_3SiH with $Ph_3C^+[CHB_{11}H_5Br_6]^-$.

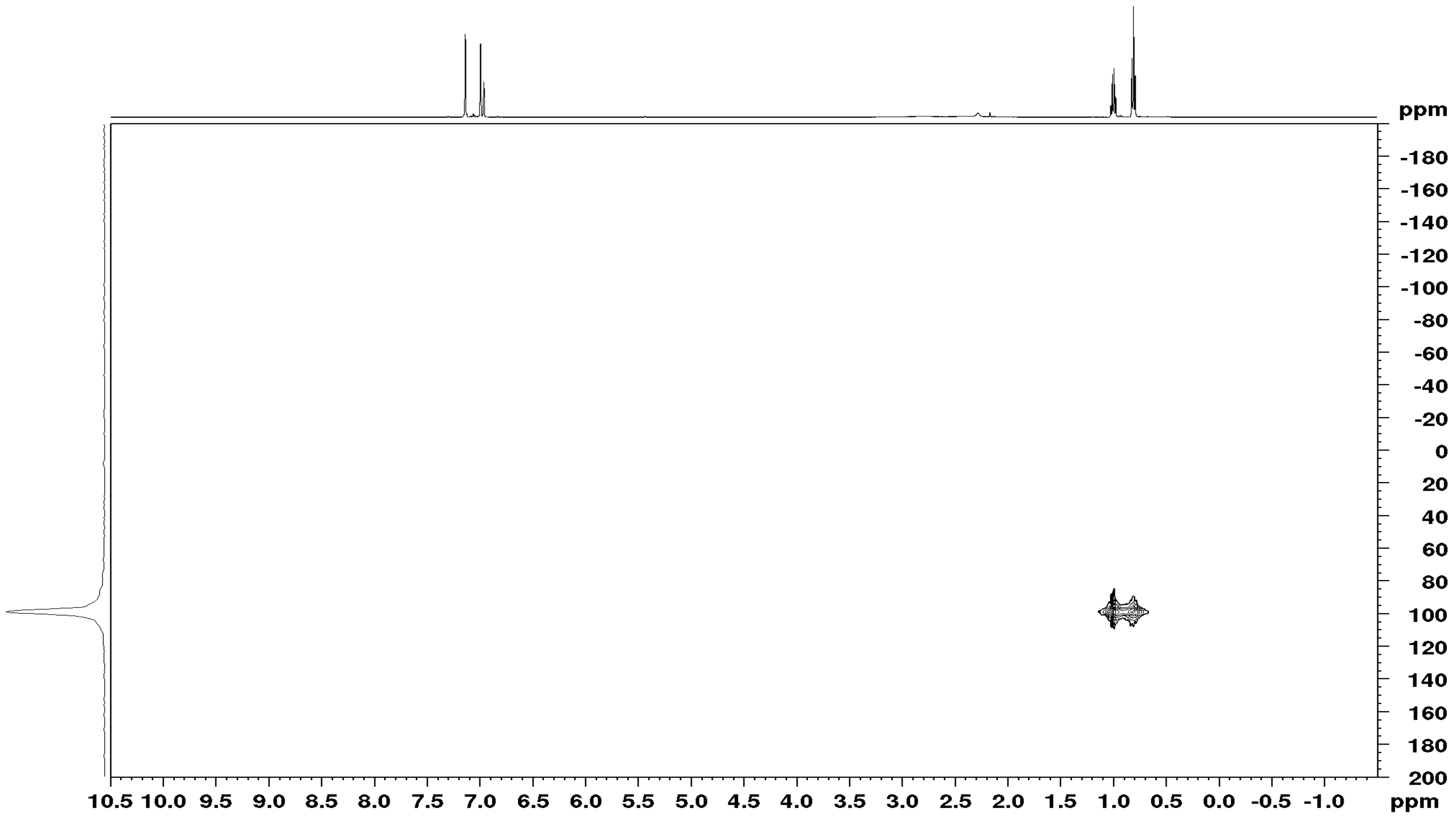

Figure S7. **^{1}H NMR** spectrum (500 MHz, $CDCl_3$, 298 K) of **3aa** from the reaction of VCP **1a** and Et_2SiH_2 (**2a**) using $Ph_3C^+[B(C_6F_5)_4]^-$ as initiator.

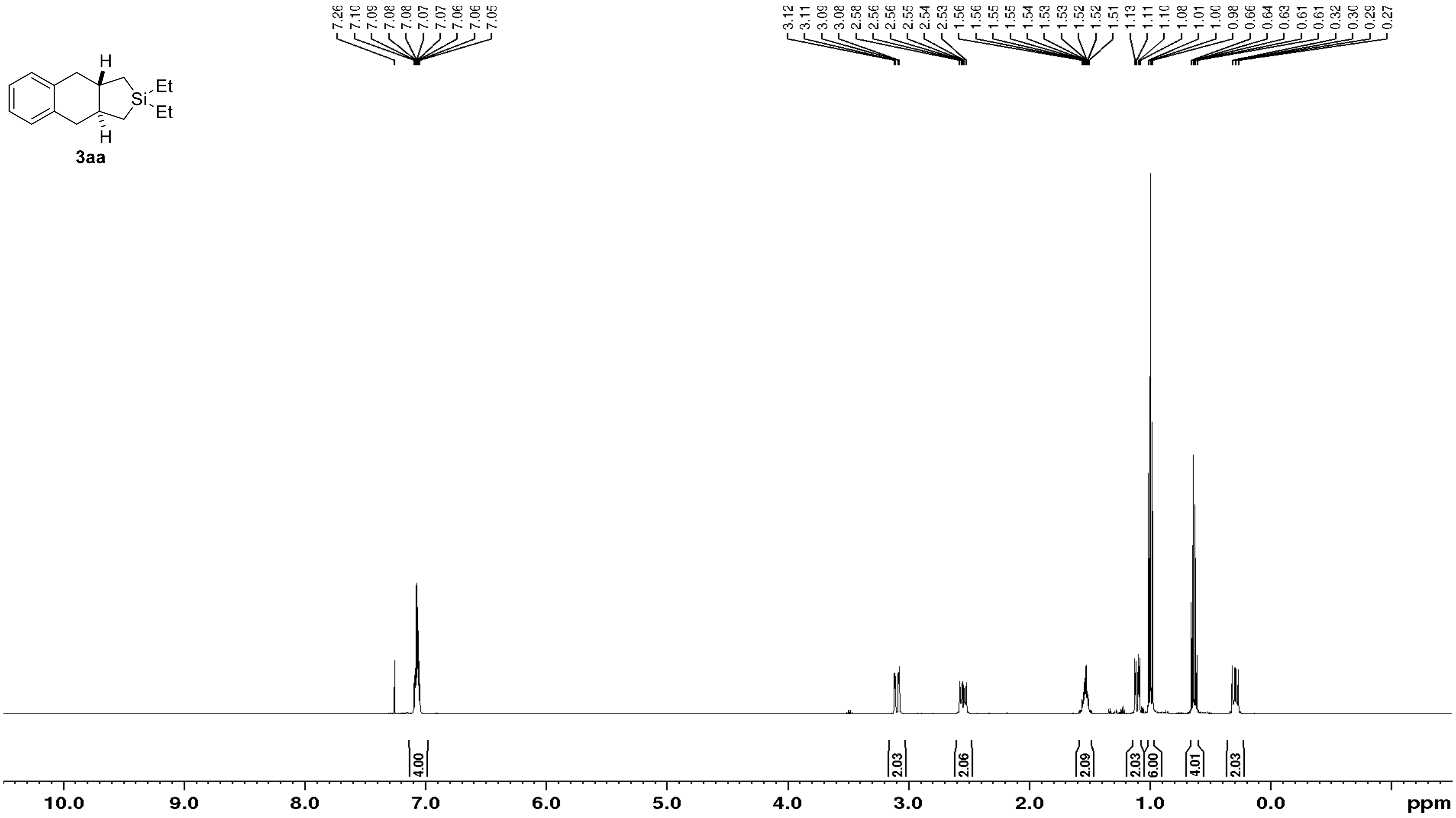

Figure S8. **$^{13}C\{^1H\}$ NMR** spectrum (126 MHz, $CDCl_3$, 298 K) of **3aa** from the reaction of VCP **1a** and Et_2SiH_2 (**2a**) using $Ph_3C^+[B(C_6F_5)_4]^-$ as initiator.

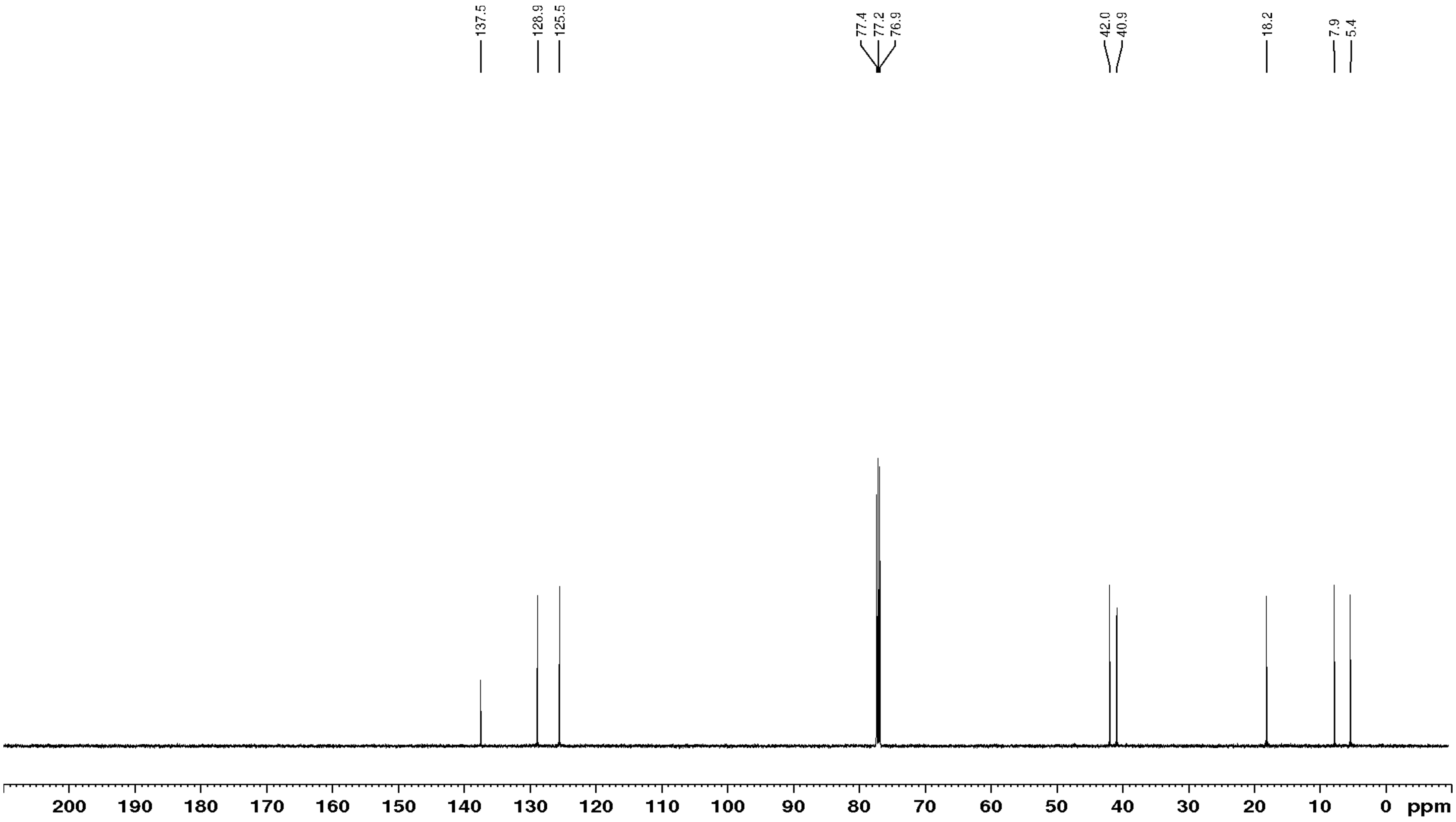

Figure S9. **$^{1}H/^{29}Si$ HMQC** NMR spectrum (500/99 MHz, $CDCl_3$, 298 K, optimized for J = 7 Hz) of **3aa** from the reaction of VCP **1a** and Et_2SiH_2 (**2a**) using $Ph_3C^+[B(C_6F_5)_4]^-$ as initiator.

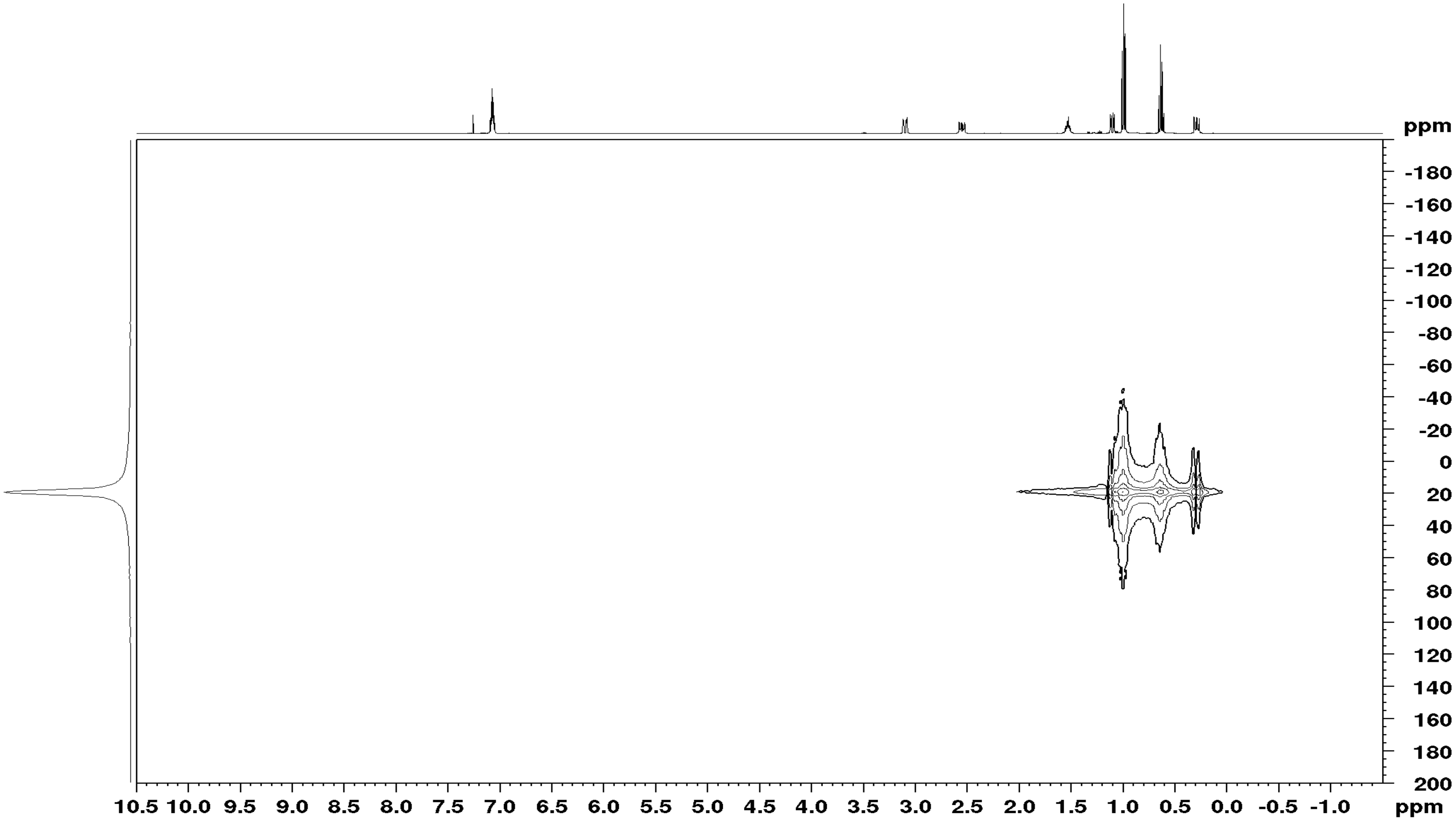

Figure S10. **^{1}H NMR** spectrum (500 MHz, $CDCl_3$, 298 K) of **3ba** from the reaction of VCP **1b** and Et_2SiH_2 (**2a**) using $Ph_3C^+[B(C_6F_5)_4]^-$ as initiator.

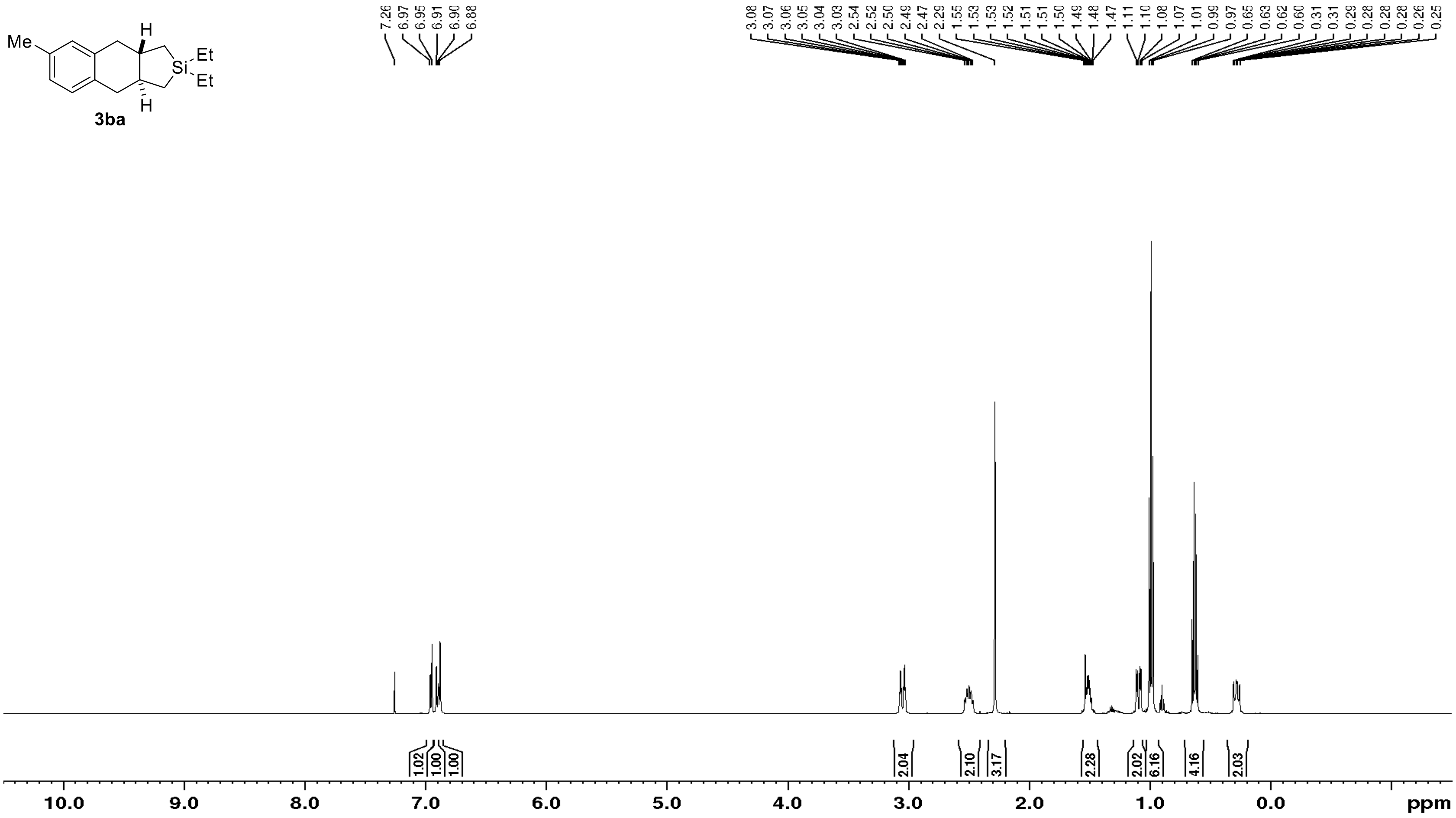

Figure S11. **$^{13}C\{^{1}H\}$** NMR spectrum (126 MHz, $CDCl_3$, 298 K) of **3ba** from the reaction of VCP **1b** and Et_2SiH_2 (**2a**) using $Ph_3C^+[B(C_6F_5)_4]^-$ as initiator.

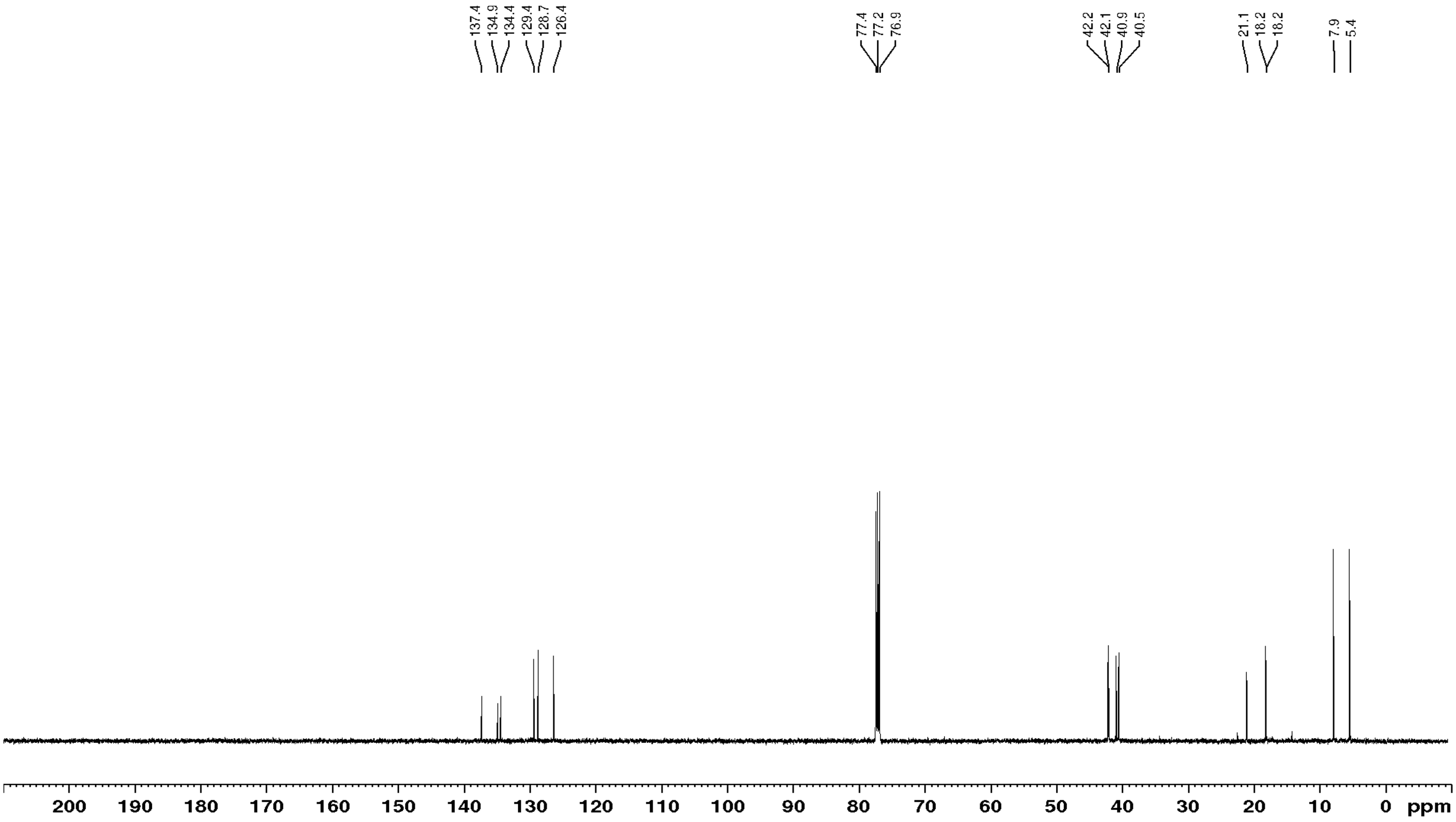

Figure S12. **$^1H/^{29}Si$ HMQC NMR** spectrum (500/99 MHz, $CDCl_3$, 298 K, optimized for J = 7 Hz) of **3ba** from the reaction of VCP **1b** and Et_2SiH_2 (**2a**) using $Ph_3C^+[B(C_6F_5)_4]^-$ as initiator.

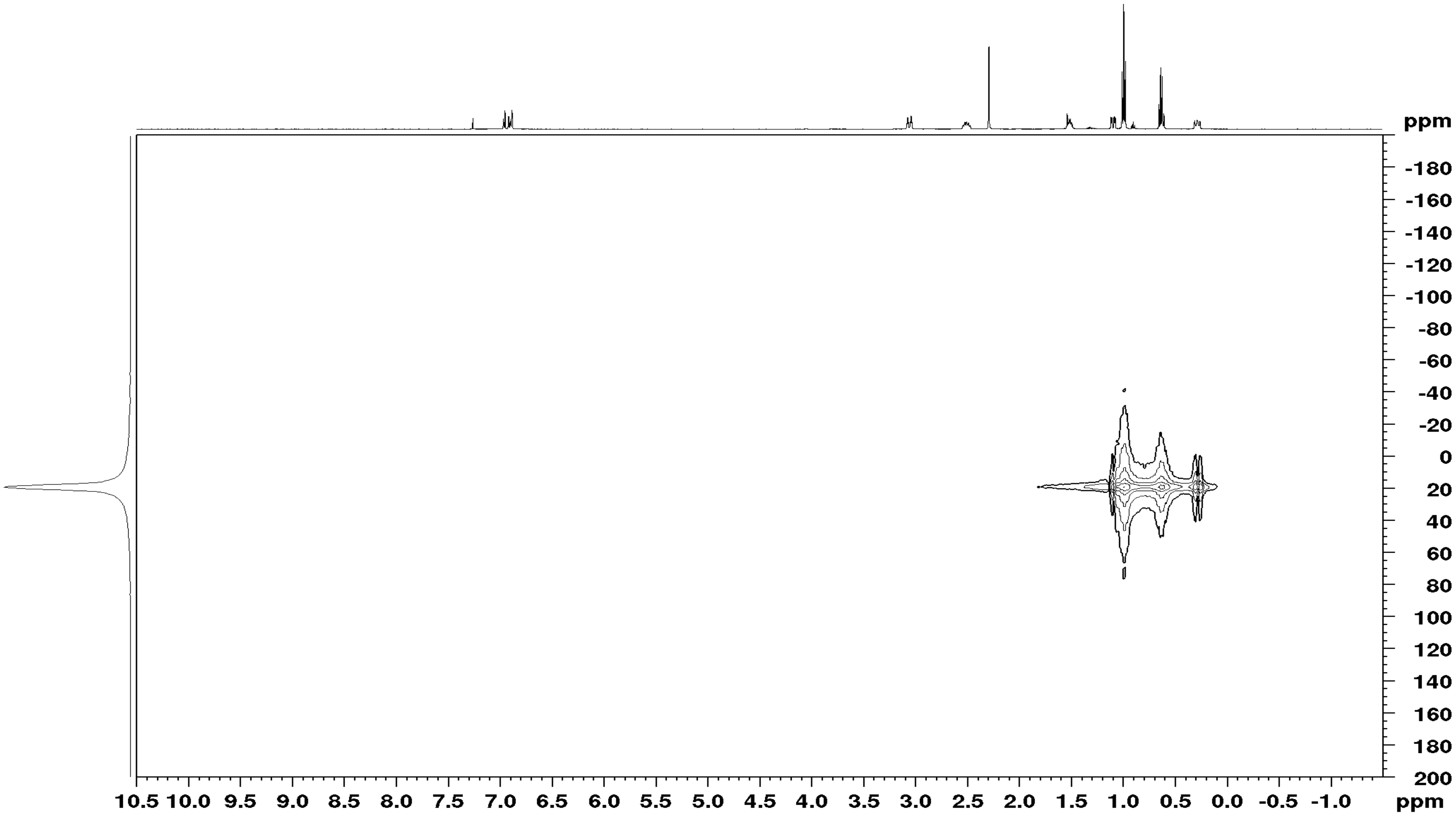

Figure S13. **^{1}H NMR** spectrum (500 MHz, $CDCl_3$, 298 K) of **3ca** from the reaction of VCP **1c** and Et_2SiH_2 (**2a**) using $Ph_3C^+[B(C_6F_5)_4]^-$ as initiator.

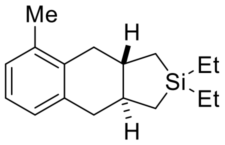

3ca

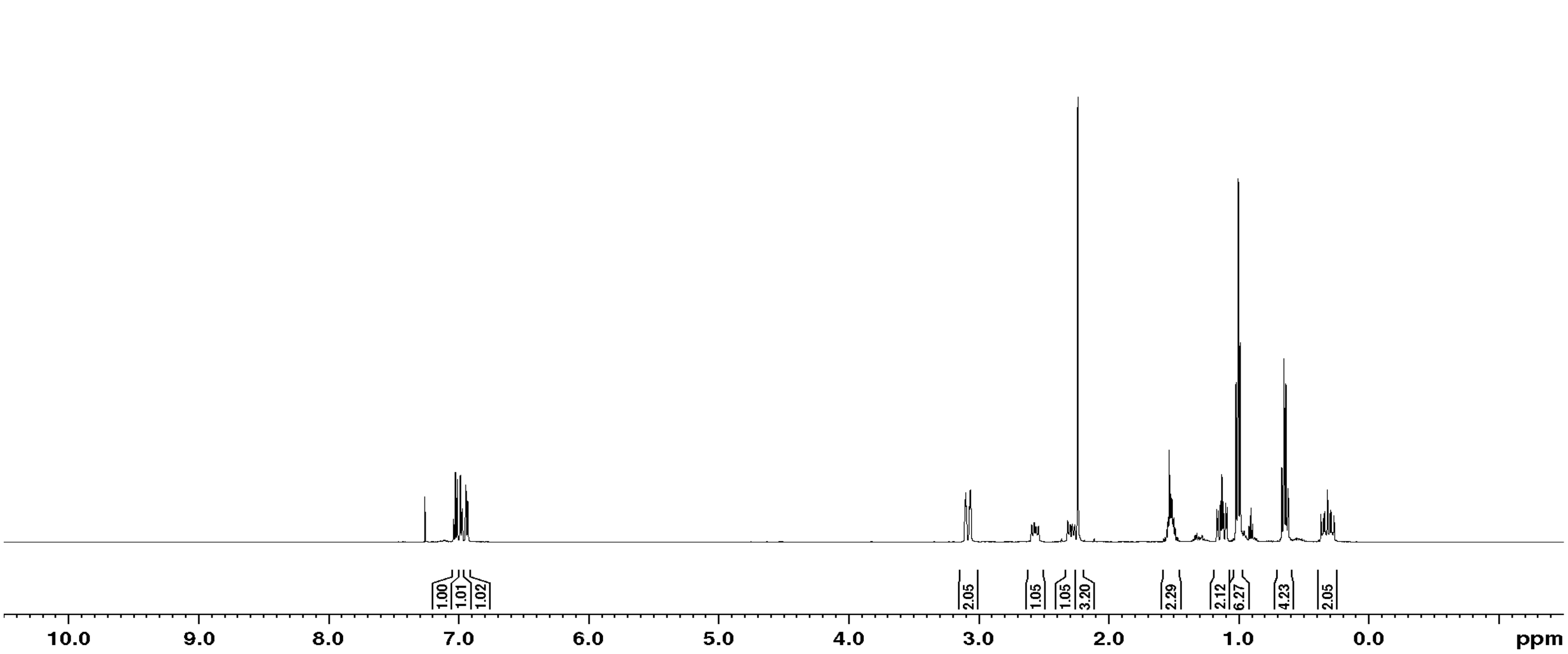

Figure S14. **$^{13}C\{^{1}H\}$ NMR** spectrum (126 MHz, $CDCl_3$, 298 K) of **3ca** from the reaction of VCP **1c** and Et_2SiH_2 (**2a**) using $Ph_3C^+[B(C_6F_5)_4]^-$ as initiator.

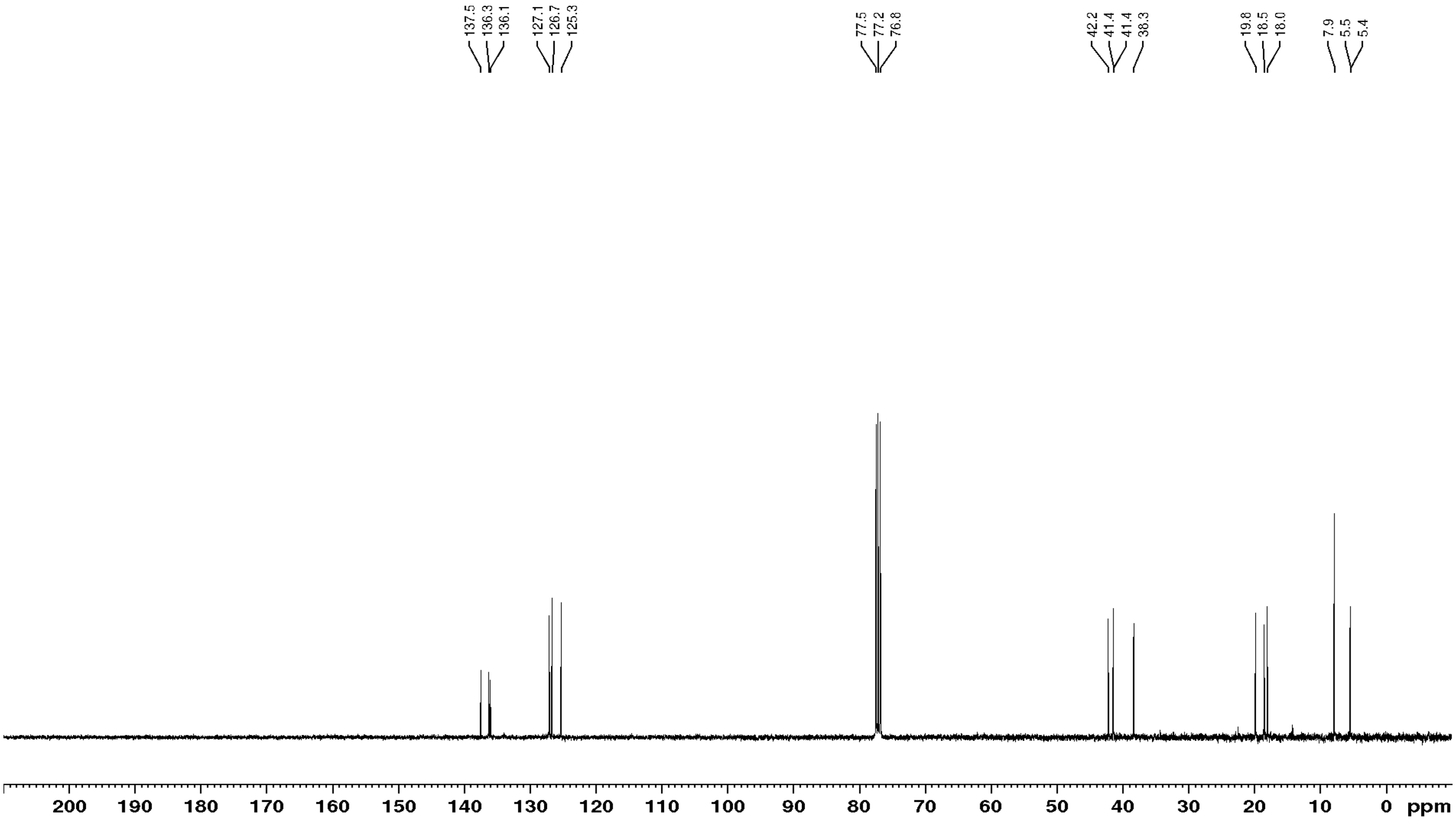

Figure S15. **$^{1}H/^{29}Si$ HMQC** NMR spectrum (500/99 MHz, $CDCl_3$, 298 K, optimized for J = 7 Hz) of **3ca** from the reaction of VCP **1c** and Et_2SiH_2 (**2a**) using $Ph_3C^+[B(C_6F_5)_4]^-$ as initiator.

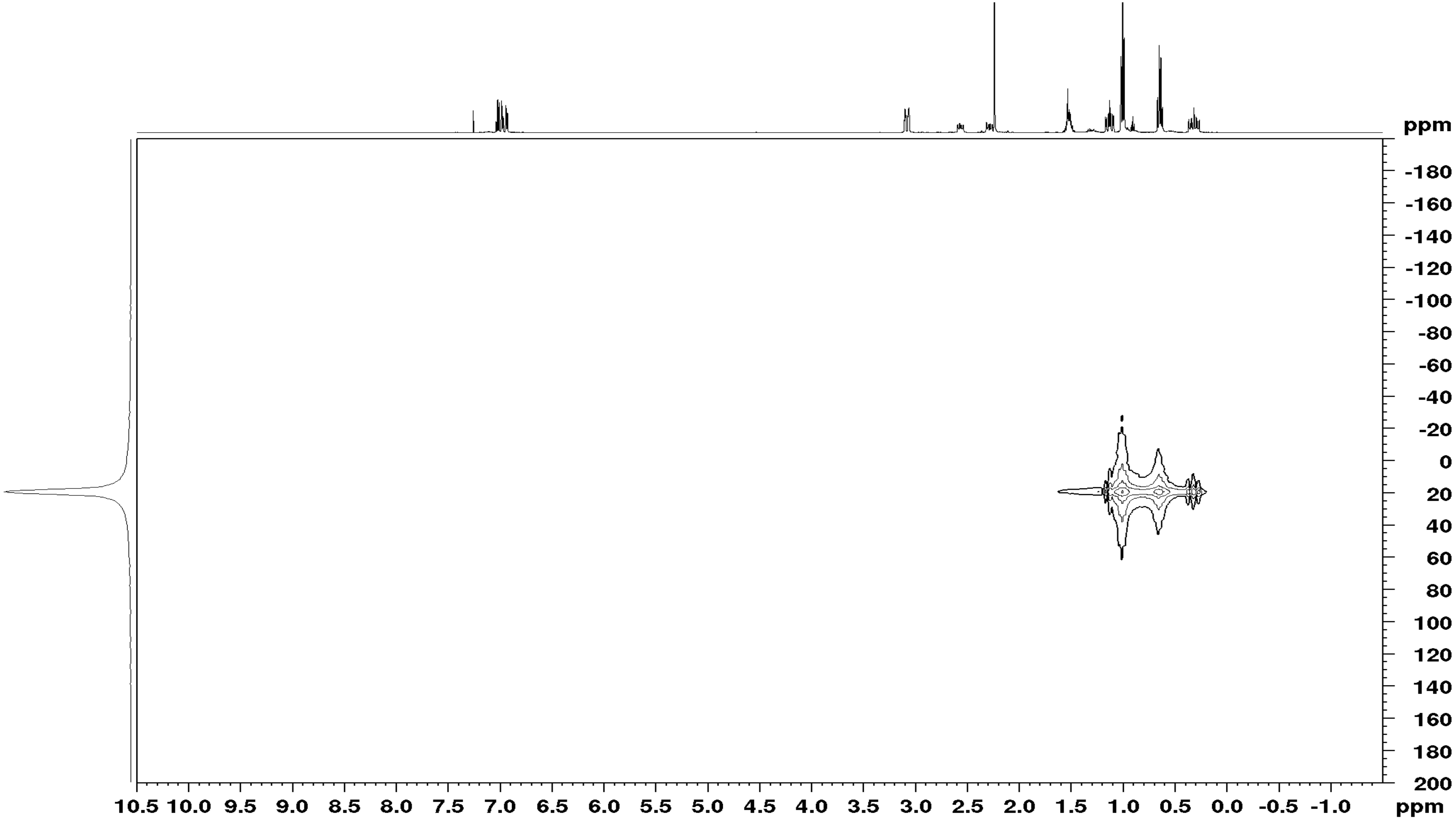

Figure S16. **^{1}H NMR** spectrum (500 MHz, $CDCl_3$, 298 K) of **3da** from the reaction of VCP **1d** and Et_2SiH_2 (**2a**) using $Ph_3C^+[B(C_6F_5)_4]^-$ as initiator.

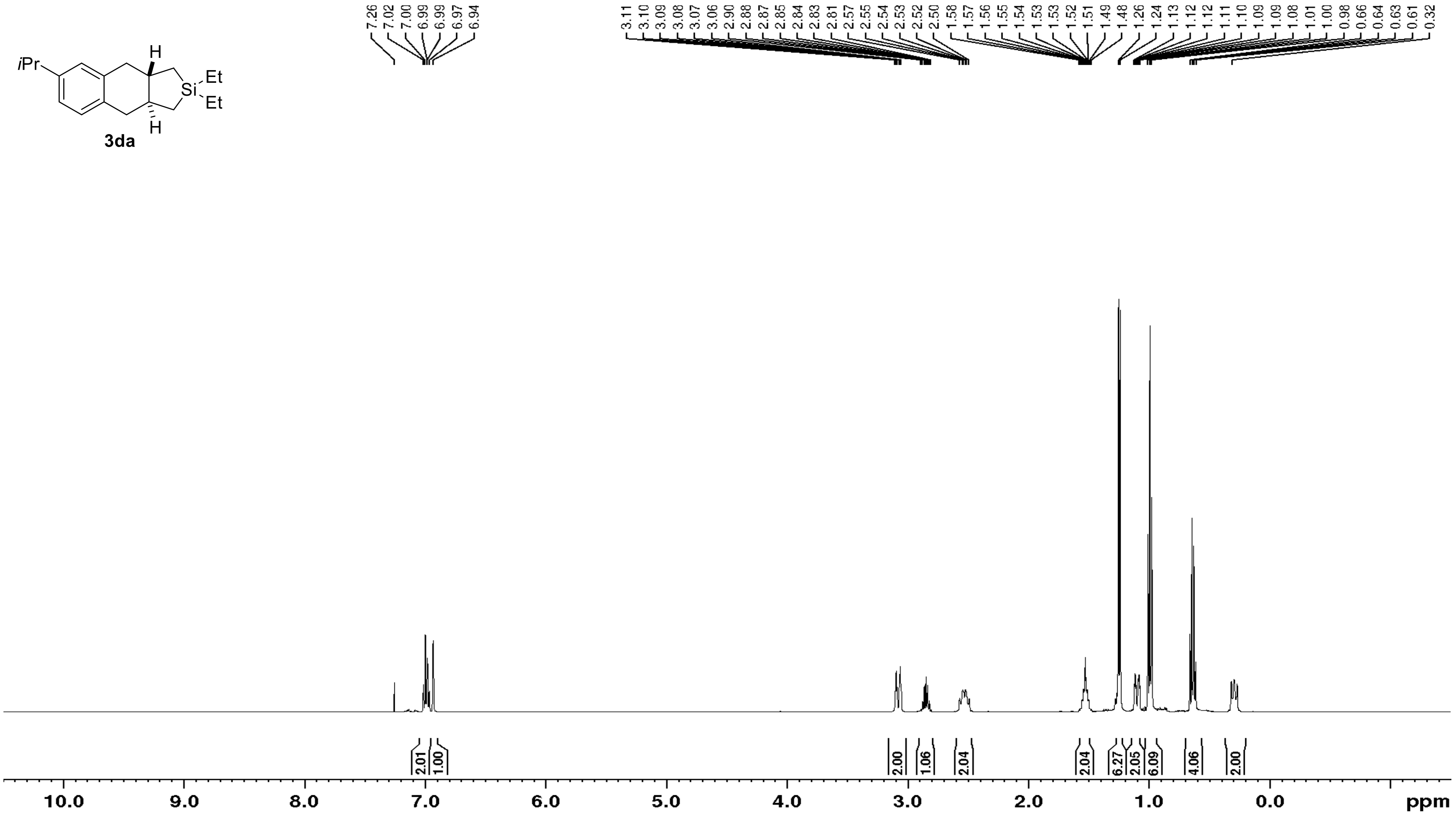

Figure S17. **$^{13}C\{^1H\}$ NMR** spectrum (126 MHz, $CDCl_3$, 298 K) of **3da** from the reaction of VCP **1d** and Et_2SiH_2 (**2a**) using $Ph_3C^+[B(C_6F_5)_4]^-$ as initiator.

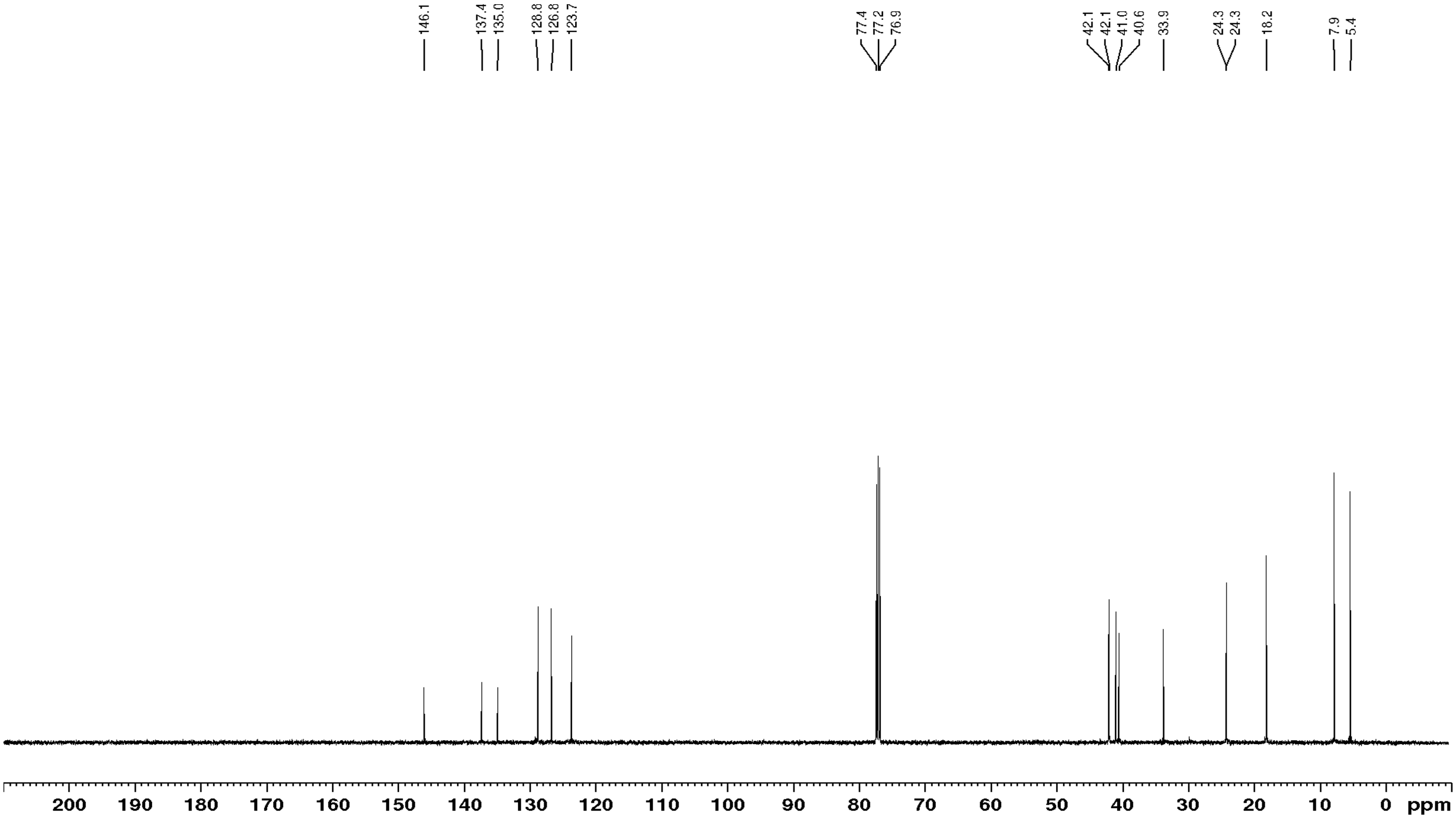

Figure S18. **$^{1}H/^{29}Si$ HMQC NMR** spectrum (500/99 MHz, $CDCl_3$, 298 K, optimized for J = 7 Hz) of **3da** from the reaction of VCP **1d** and Et_2SiH_2 (**2a**) using $Ph_3C^+[B(C_6F_5)_4]^-$ as initiator.

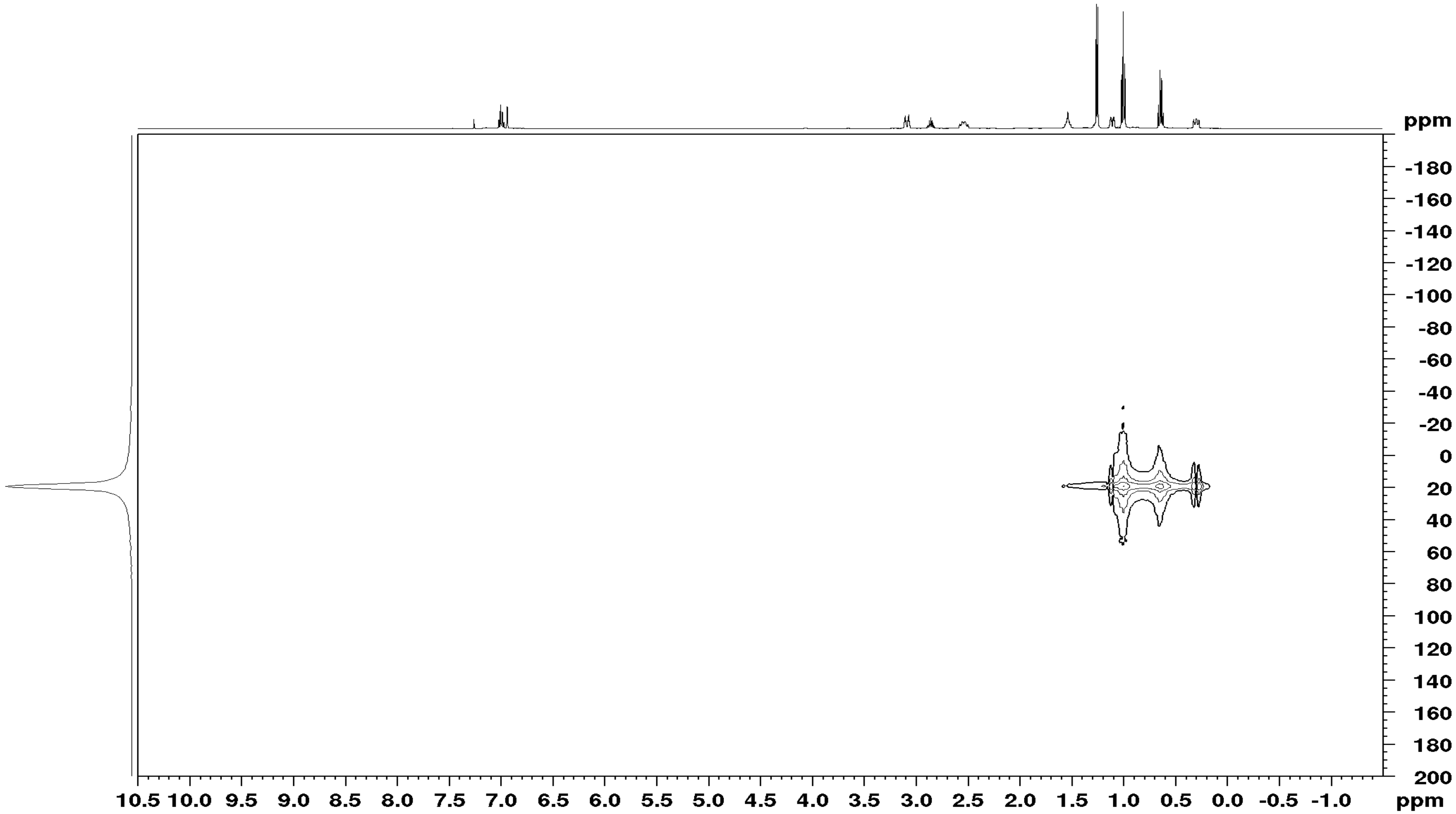

Figure S19. [1]**H NMR** spectrum (500 MHz, $CDCl_3$, 298 K) of **3ea/3aa** from the reaction of VCP **1e** and Et_2SiH_2 (**2a**) using $Ph_3C^+[B(C_6F_5)_4]^-$ as initiator (* for **3ea**).

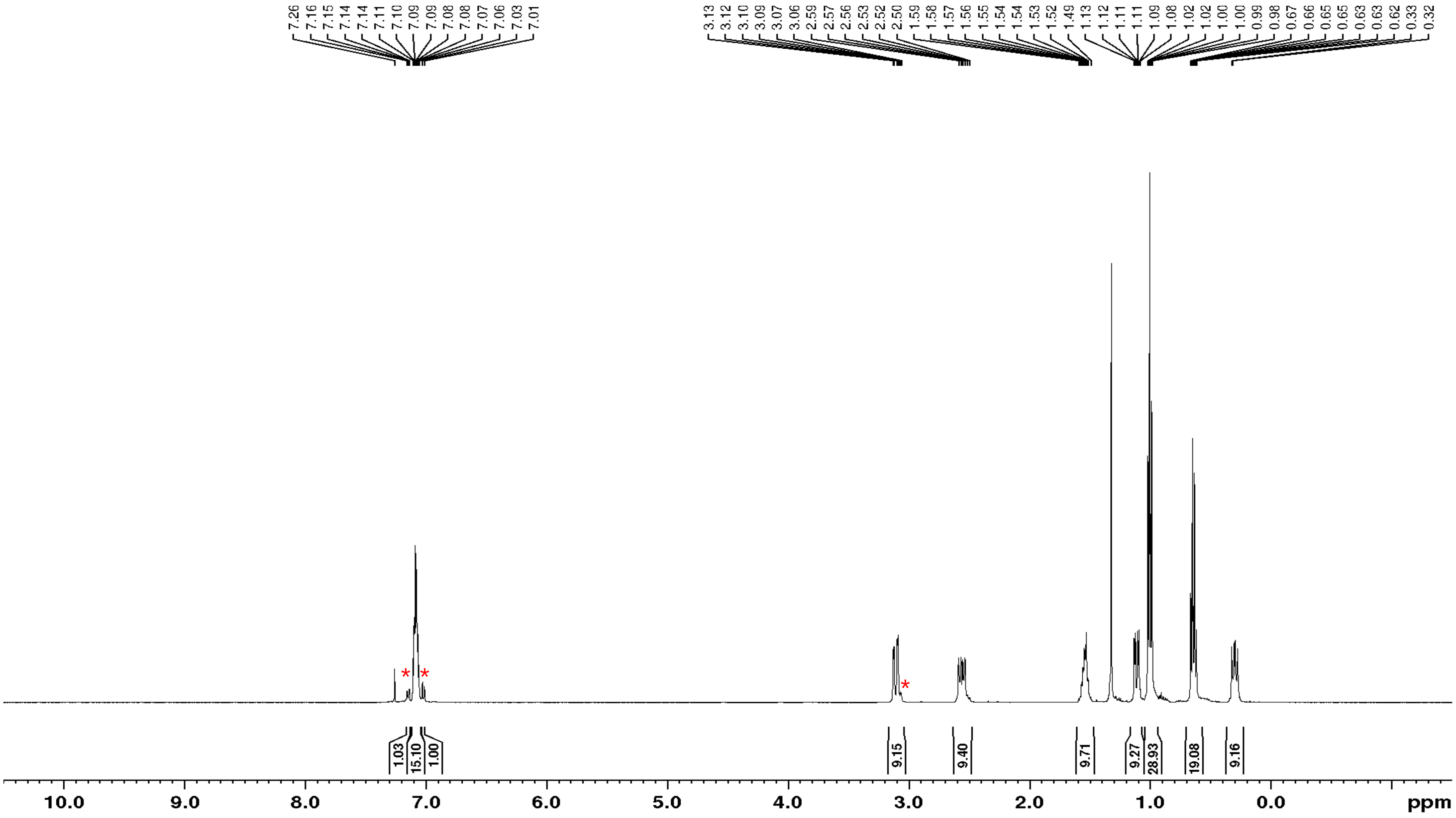

Figure S20. **^{1}H NMR** spectrum (500 MHz, $CDCl_3$, 298 K) of **3ga** from the reaction of VCP **1g** and Et_2SiH_2 (**2a**) using $Ph_3C^+[B(C_6F_5)_4]^-$ as initiator.

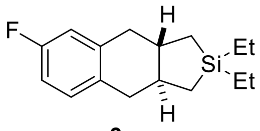

3ga

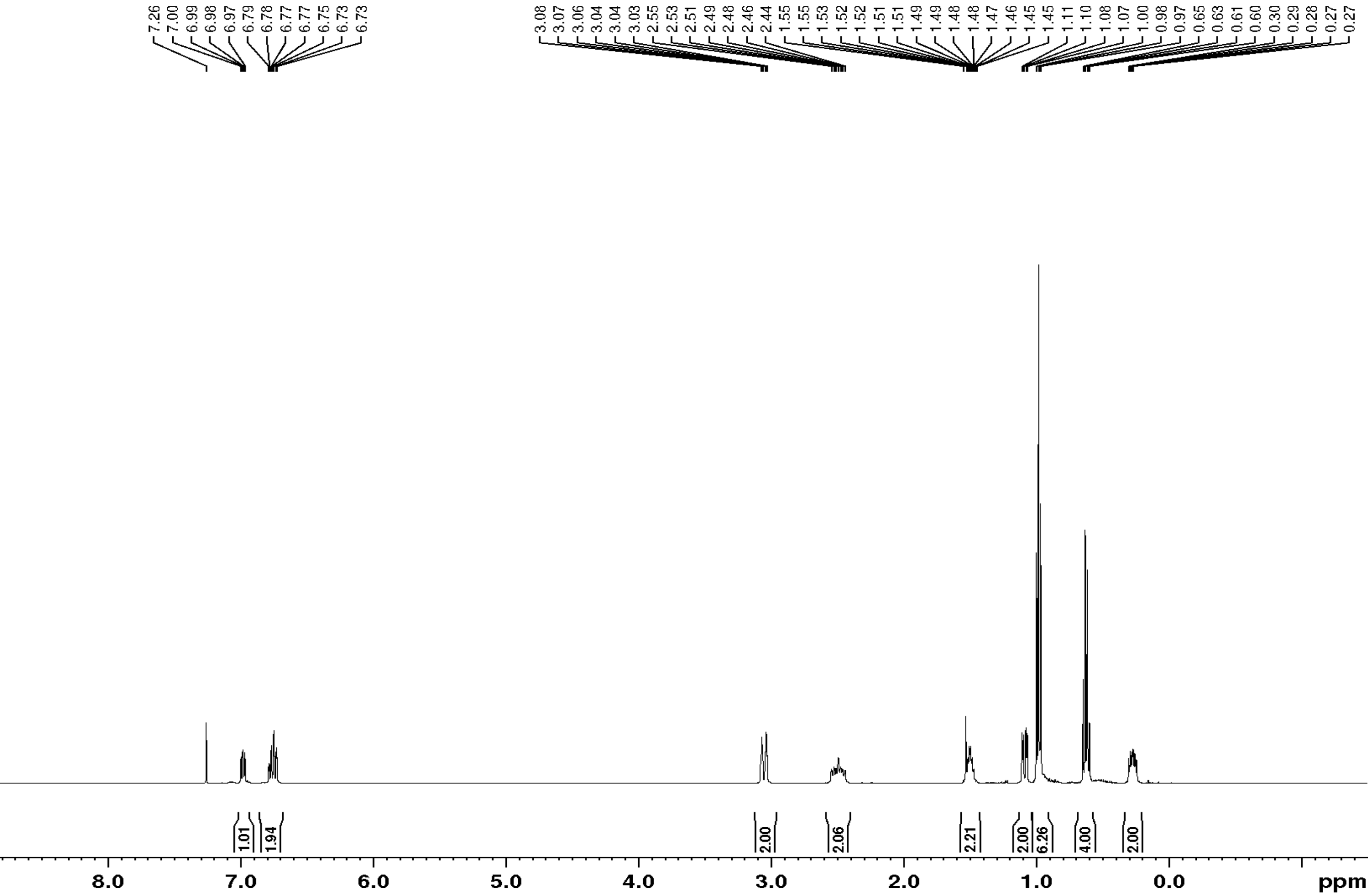

Figure S21. **^{13}C{^{1}H} NMR** spectrum (126 MHz, $CDCl_3$, 298 K) of **3ga** from the reaction of VCP **1g** and Et_2SiH_2 (**2a**) using $Ph_3C^+[B(C_6F_5)_4]^-$ as initiator.

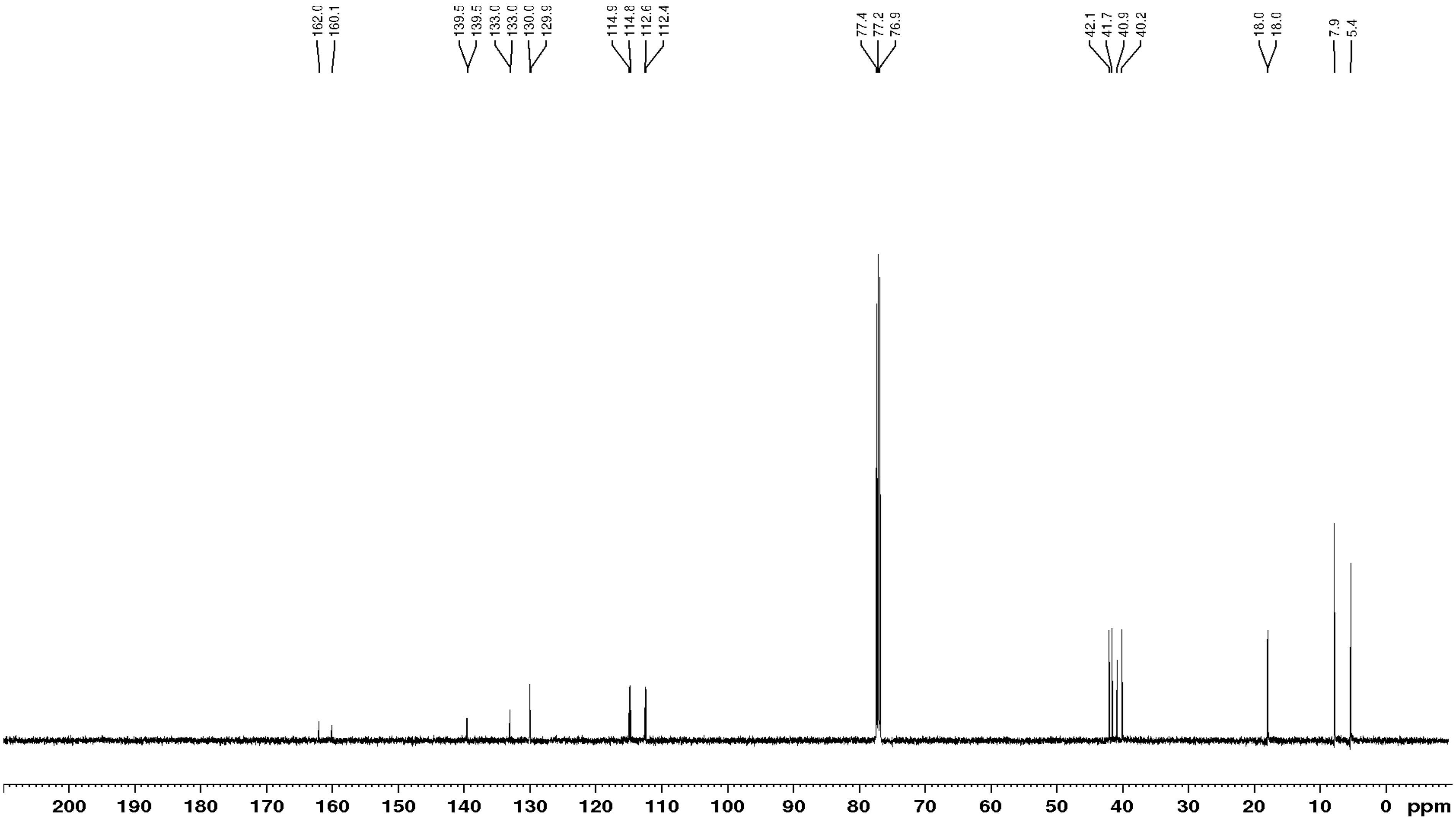

Figure S22. **^{1}H/^{29}Si HMQC NMR** spectrum (500/99 MHz, $CDCl_3$, 298 K, optimized for *J* = 7 Hz) of **3ga** from the reaction of VCP **1g** and Et_2SiH_2 (**2a**) using $Ph_3C^+[B(C_6F_5)_4]^-$ as initiator.

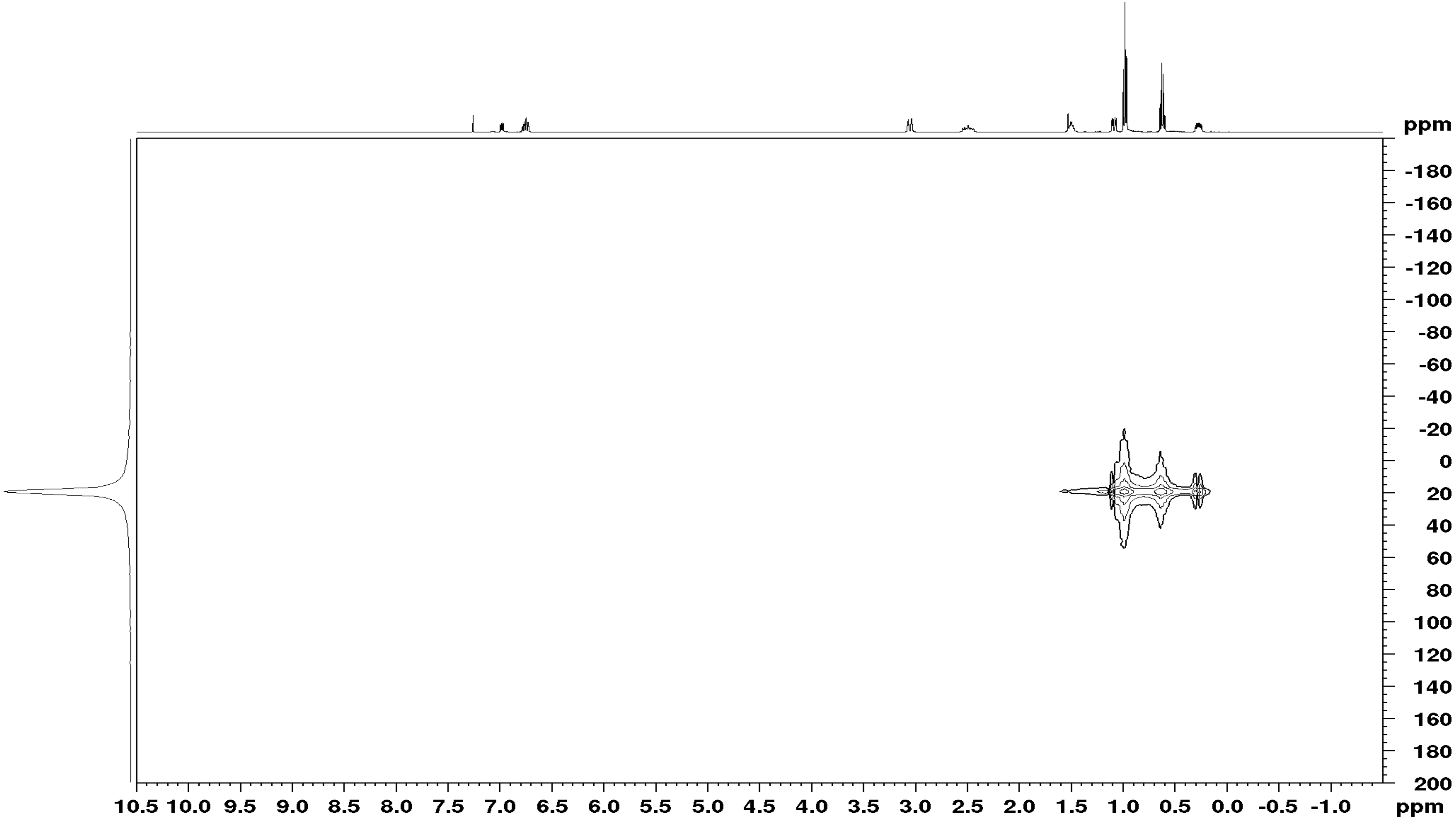

Figure S23. **^{19}F NMR** spectrum (471 MHz, $CDCl_3$, 298 K) of **3ga** from the reaction of VCP **1g** and Et_2SiH_2 (**2a**) using $Ph_3C^+[B(C_6F_5)_4]^-$ as initiator.

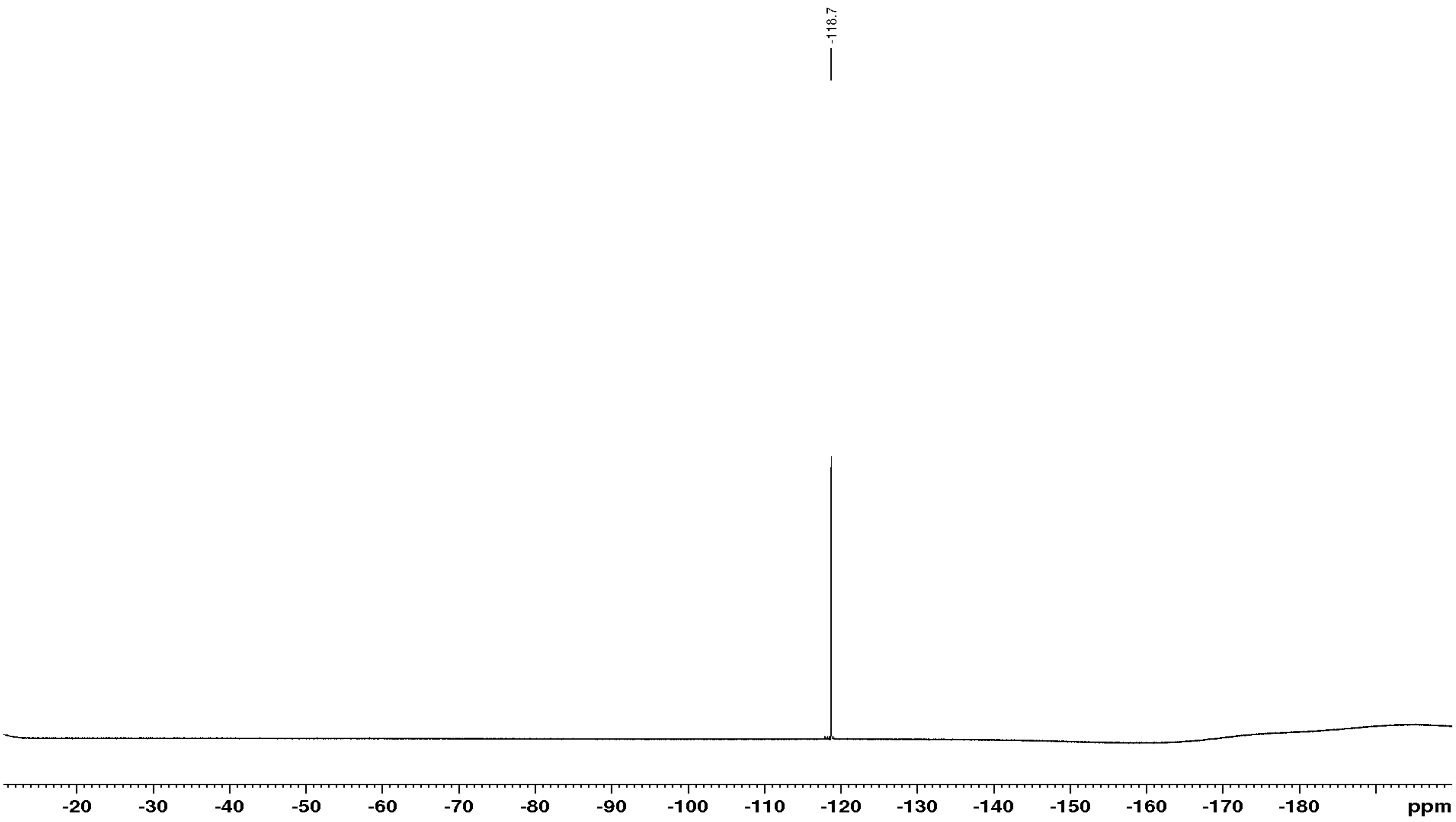

Figure S24. **^{1}H NMR** spectrum (500 MHz, $CDCl_3$, 298 K) of **3ha** from the reaction of VCP **1h** and Et_2SiH_2 (**2a**) using $Ph_3C^+[B(C_6F_5)_4]^-$ as initiator.

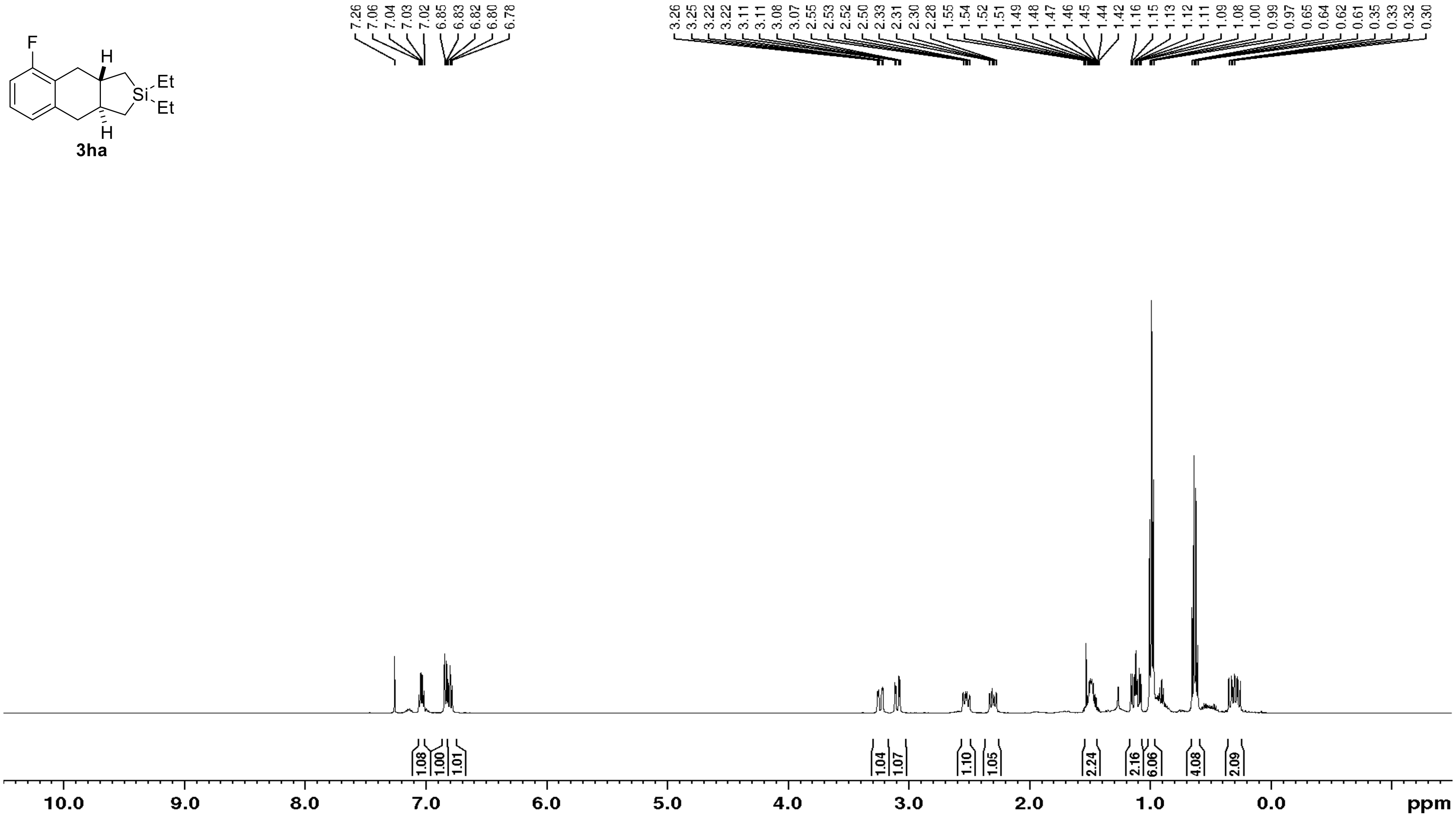

Figure S25. **$^{13}C\{^1H\}$ NMR** spectrum (126 MHz, $CDCl_3$, 298 K) of **3ha** from the reaction of VCP **1h** and Et_2SiH_2 (**2a**) using $Ph_3C^+[B(C_6F_5)_4]^-$ as initiator.

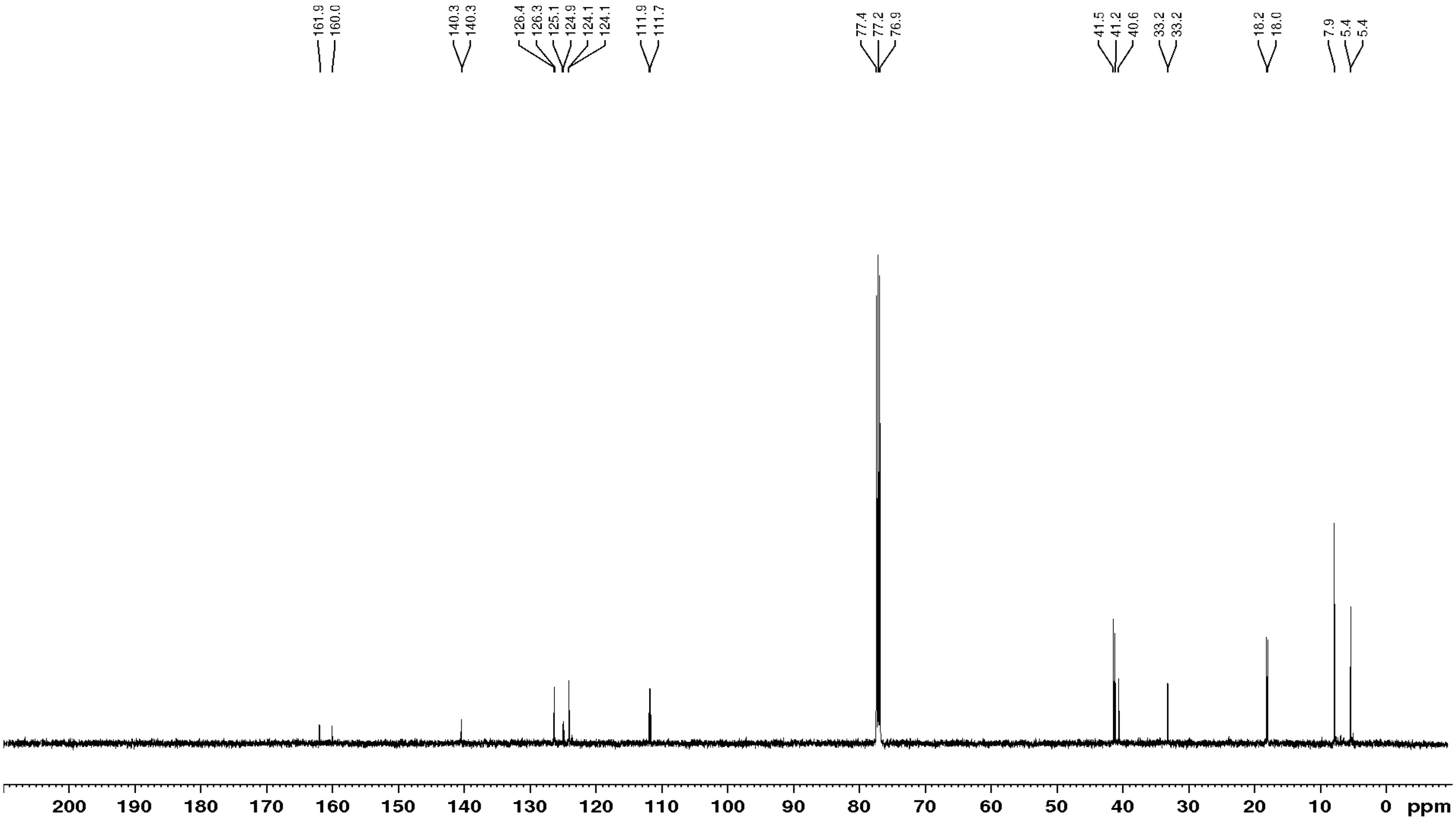

Figure S26. **^{1}H/^{29}Si HMQC NMR** spectrum (500/99 MHz, $CDCl_3$, 298 K, optimized for J = 7 Hz) of **3ha** from the reaction of VCP **1h** and Et_2SiH_2 (**2a**) using $Ph_3C^+[B(C_6F_5)_4]^-$ as initiator.

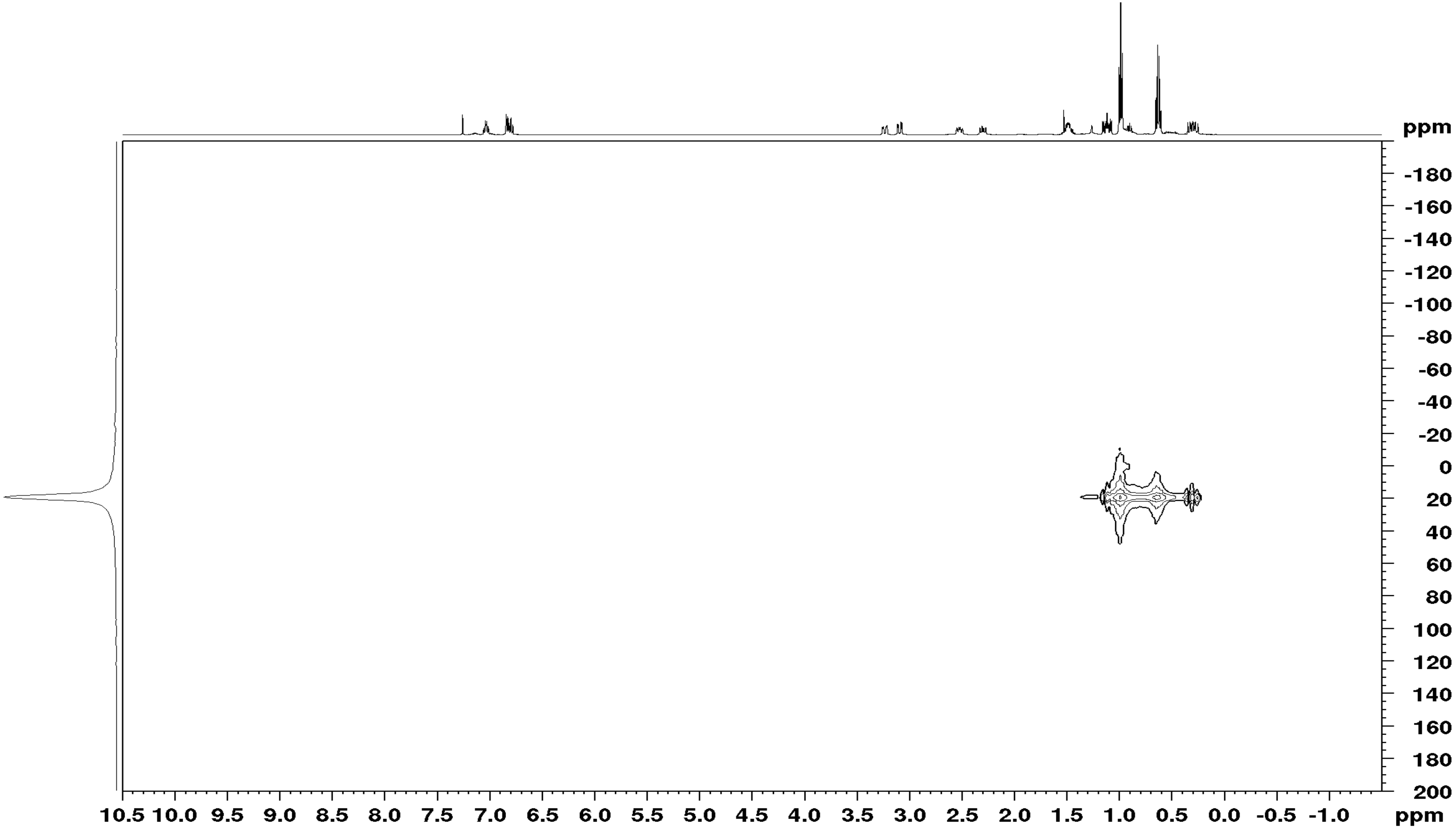

Figure S27. **^{19}F NMR** spectrum (471 MHz, $CDCl_3$, 298 K) of **3ha** from the reaction of VCP **1h** and Et_2SiH_2 (**2a**) using $Ph_3C^+[B(C_6F_5)_4]^-$ as initiator.

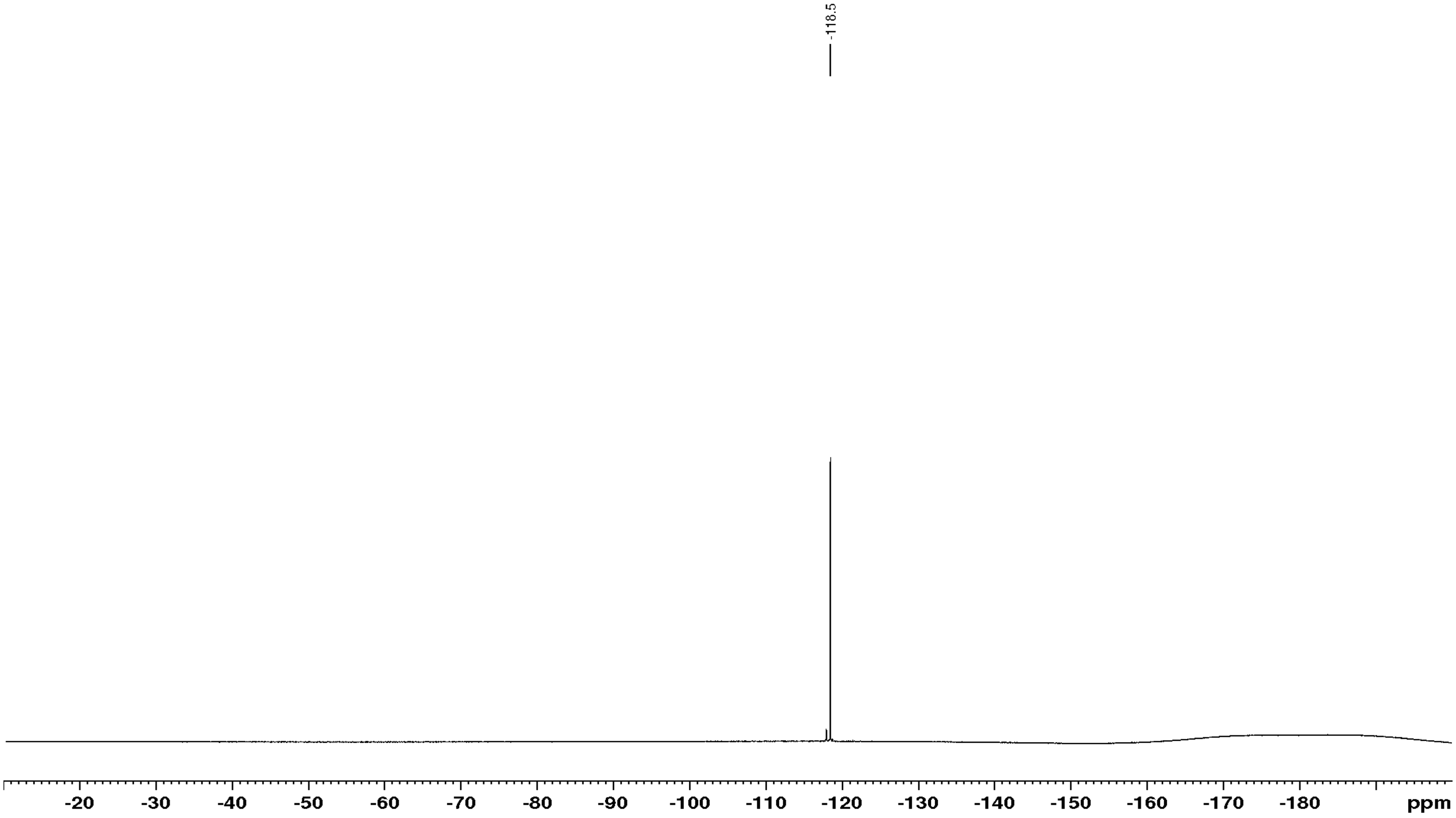

Figure S28. **^{1}H NMR** spectrum (500 MHz, $CDCl_3$, 298 K) of **3ia** from the reaction of VCP **1i** and Et_2SiH_2 (**2a**) using $Ph_3C^+[B(C_6F_5)_4]^-$ as initiator.

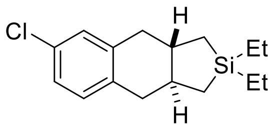

3ia

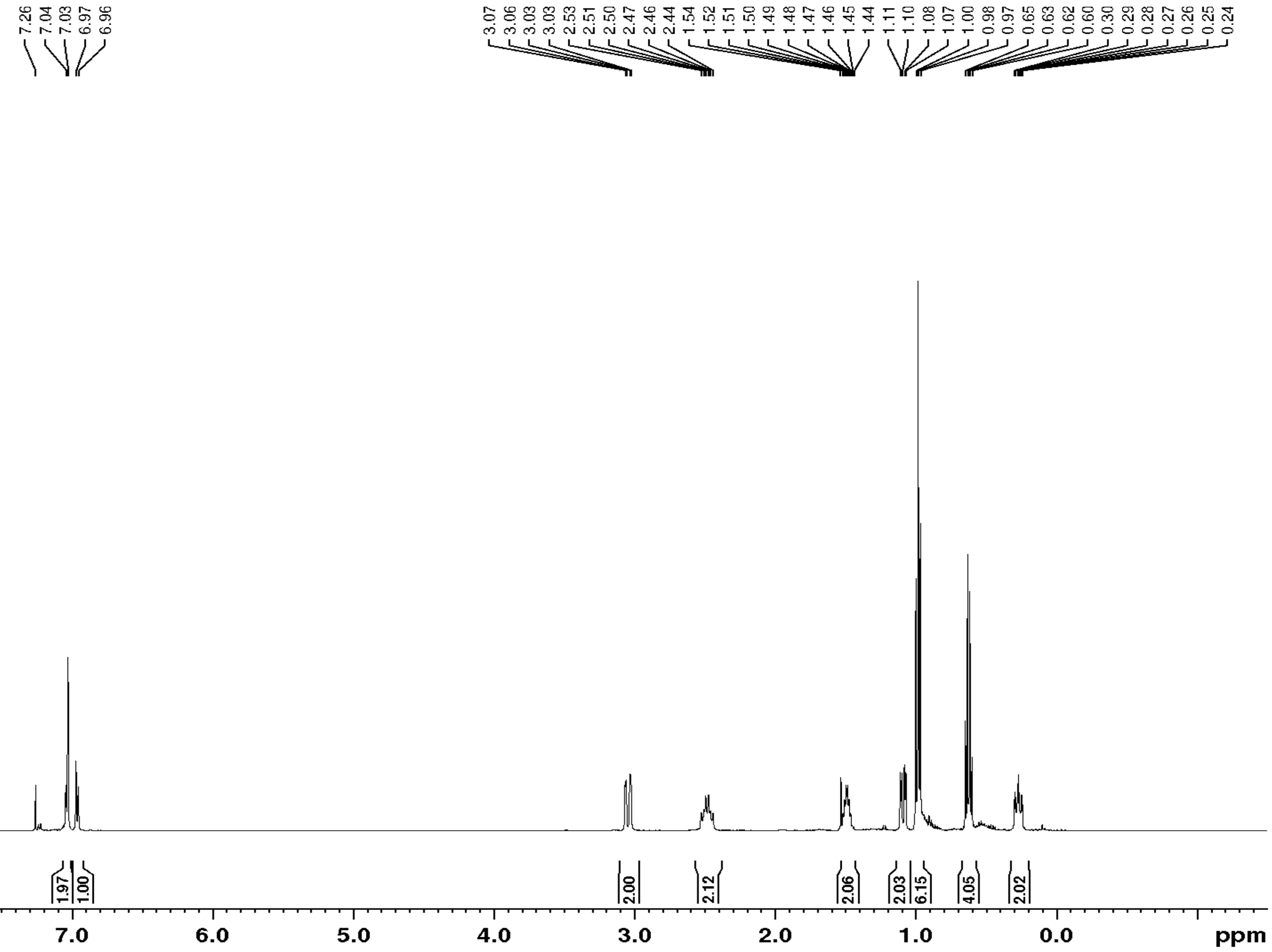

Figure S29. **$^{13}C\{^1H\}$ NMR** spectrum (126 MHz, $CDCl_3$, 298 K) of **3ia** from the reaction of VCP **1i** and Et_2SiH_2 (**2a**) using $Ph_3C^+[B(C_6F_5)_4]^-$ as initiator.

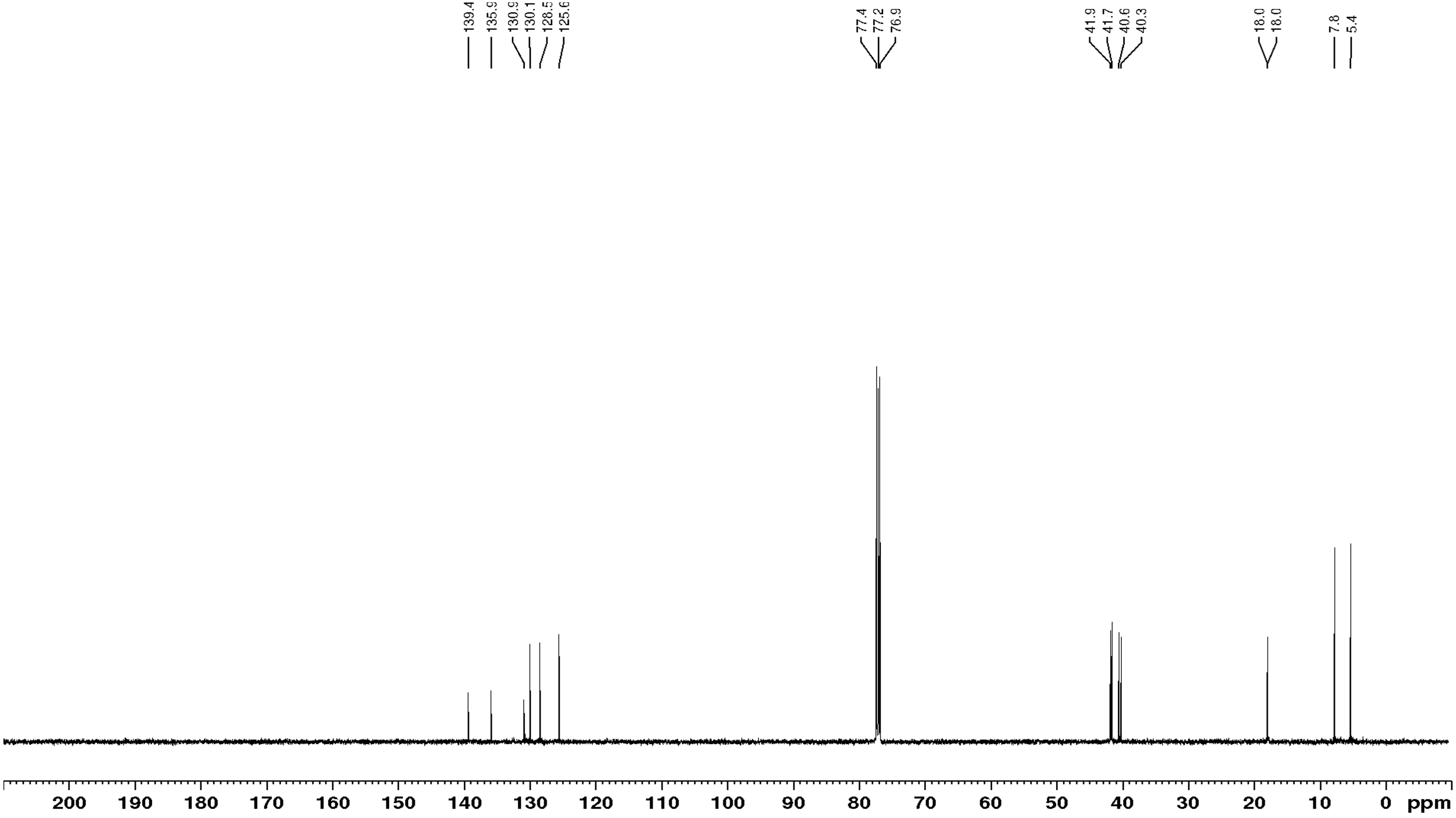

Figure S30. **$^{1}H/^{29}Si$ HMQC NMR** spectrum (500/99 MHz, $CDCl_3$, 298 K, optimized for J = 7 Hz) of **3ia** from the reaction of VCP **1i** and Et_2SiH_2 (**2a**) using $Ph_3C^+[B(C_6F_5)_4]^-$ as initiator.

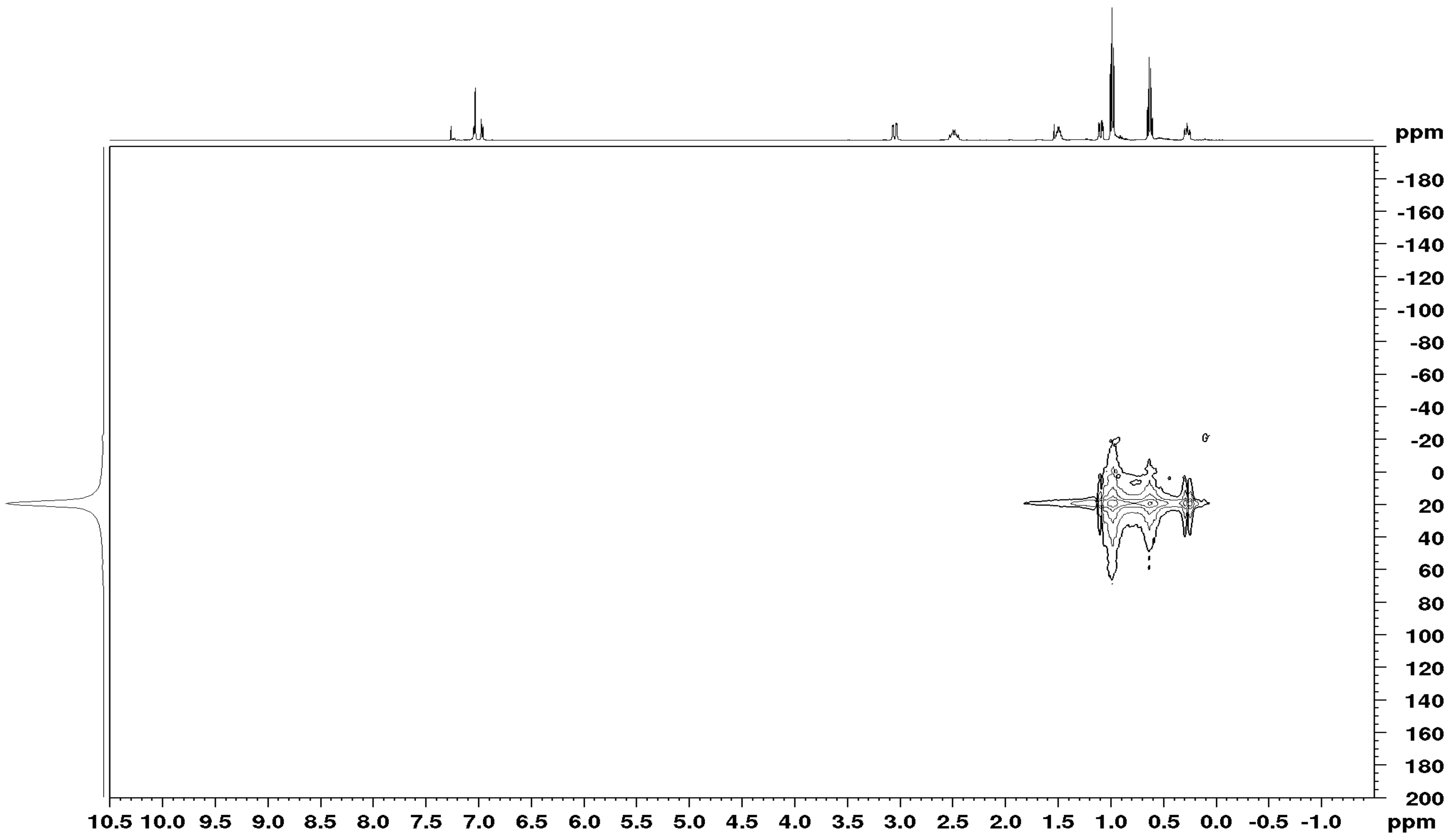

Figure S31. [1]**H NMR** spectrum (500 MHz, $CDCl_3$, 298 K) of **3ja** from the reaction of VCP **1j** and Et_2SiH_2 (**2a**) using $Ph_3C^+[B(C_6F_5)_4]^-$ as initiator.

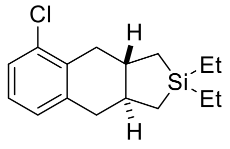

3ja

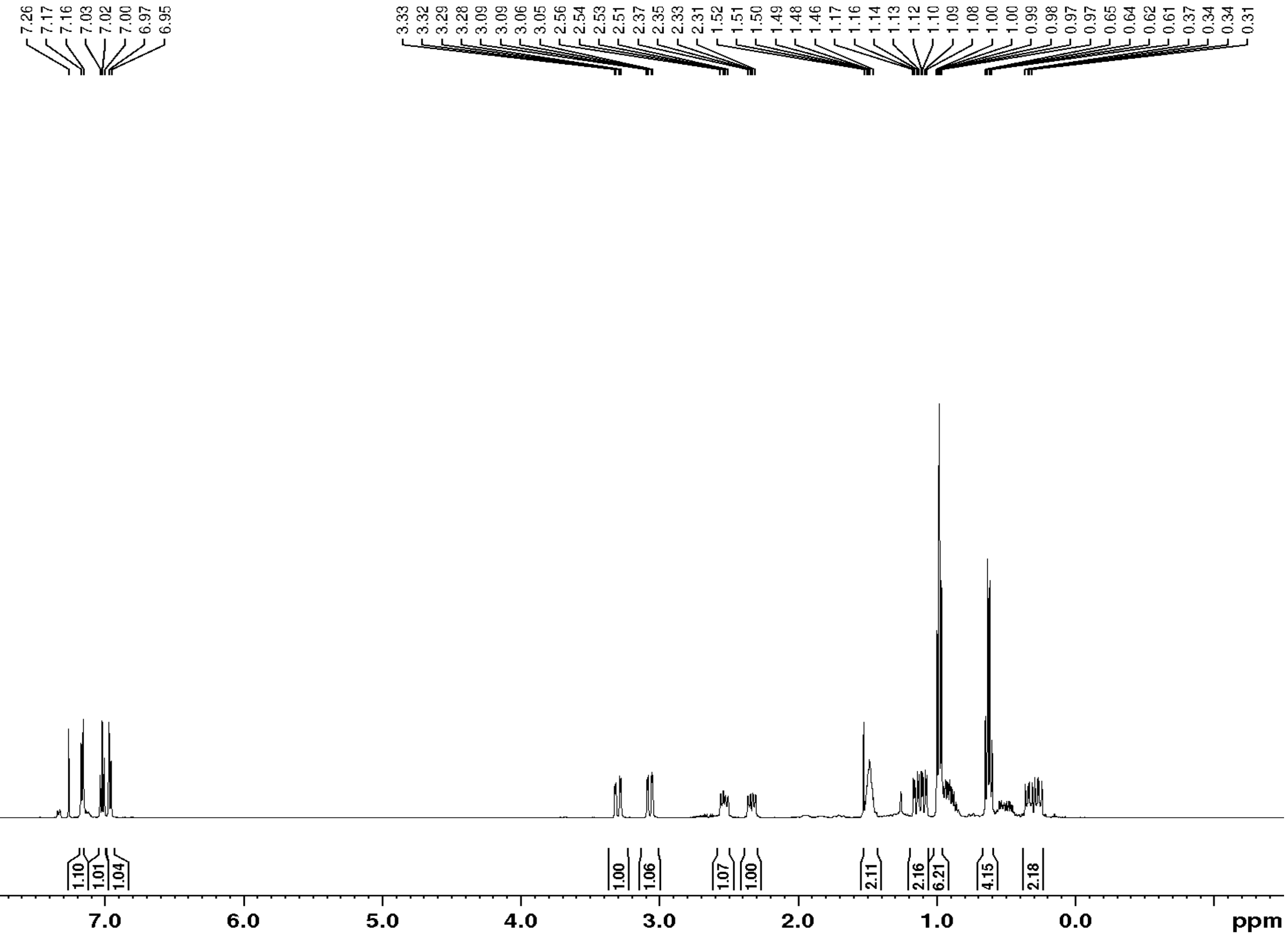

Figure S32. **$^{13}C\{^{1}H\}$ NMR** spectrum (126 MHz, $CDCl_3$, 298 K) of **3ja** from the reaction of VCP **1j** and Et_2SiH_2 (**2a**) using $Ph_3C^+[B(C_6F_5)_4]^-$ as initiator.

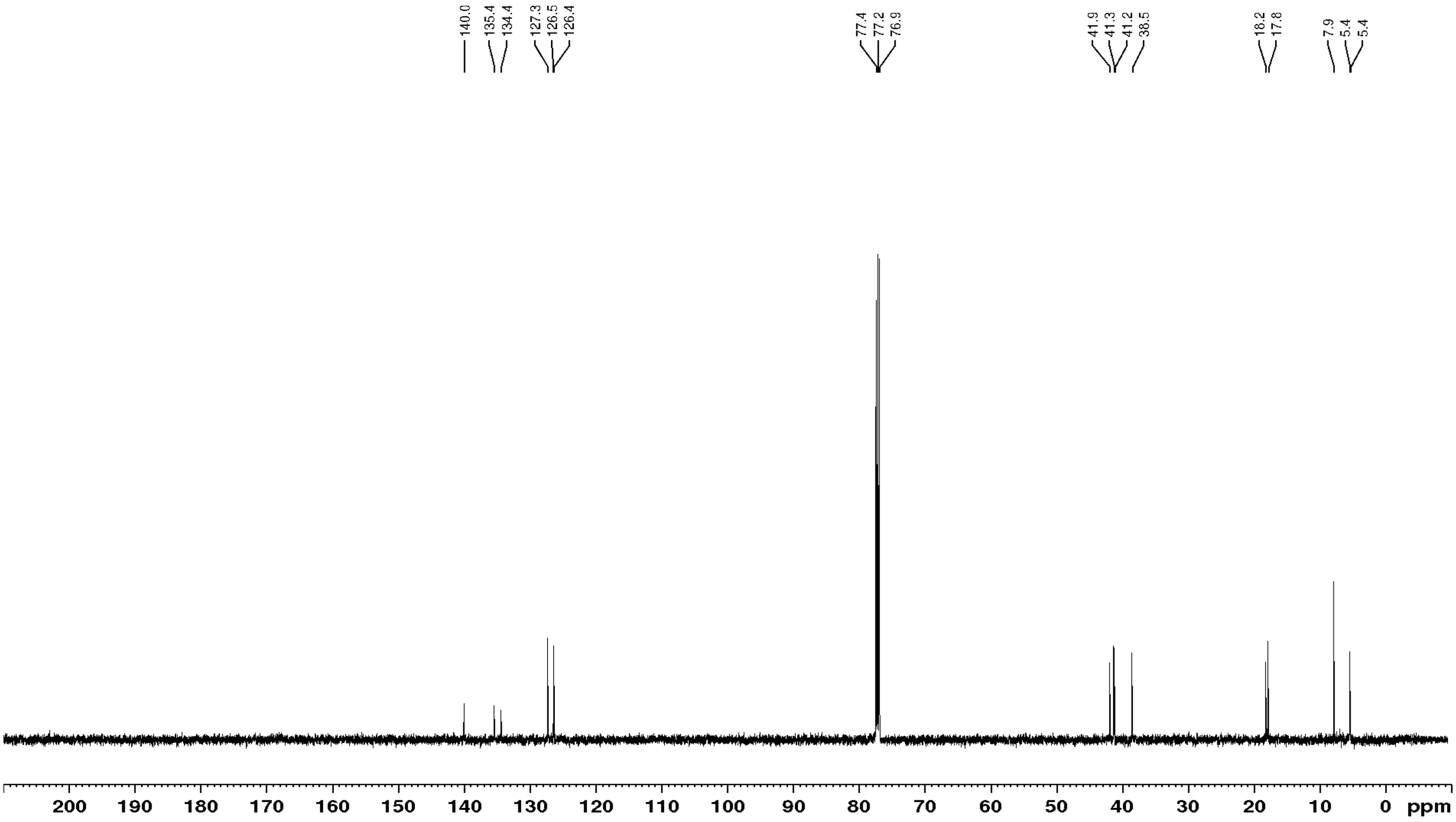

Figure S33. **^{1}H/^{29}Si HMQC NMR** spectrum (500/99 MHz, $CDCl_3$, 298 K, optimized for J = 7 Hz) of **3ja** from the reaction of VCP **1j** and Et_2SiH_2 (**2a**) using $Ph_3C^+[B(C_6F_5)_4]^-$ as initiator.

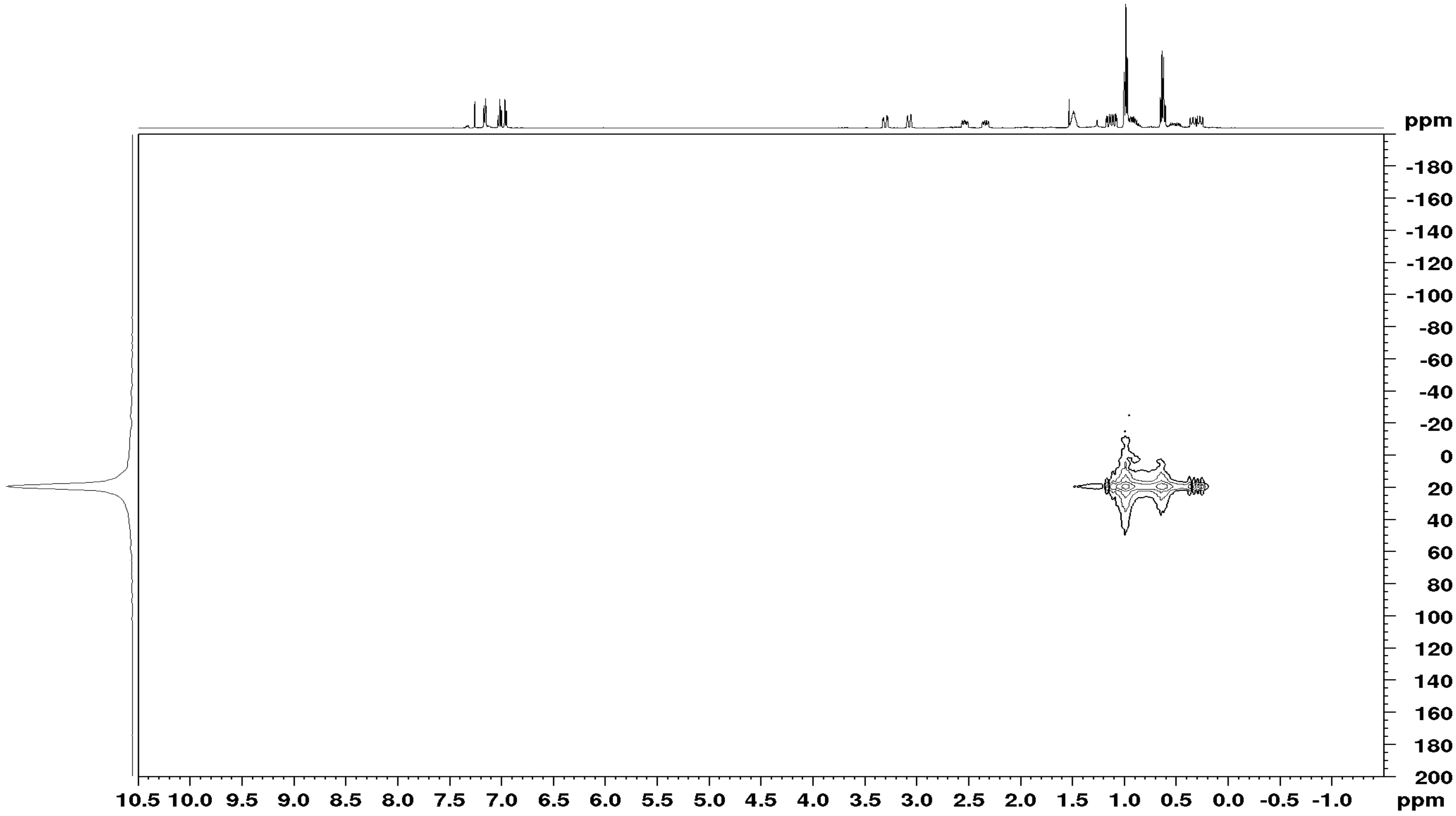

Figure S34. **^{1}H NMR** spectrum (500 MHz, $CDCl_3$, 298 K) of **3ka** from the reaction of VCP **1k** and Et_2SiH_2 (**2a**) using $Ph_3C^+[B(C_6F_5)_4]^-$ as initiator.

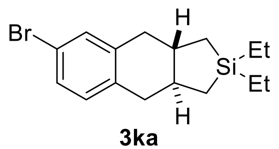

3ka

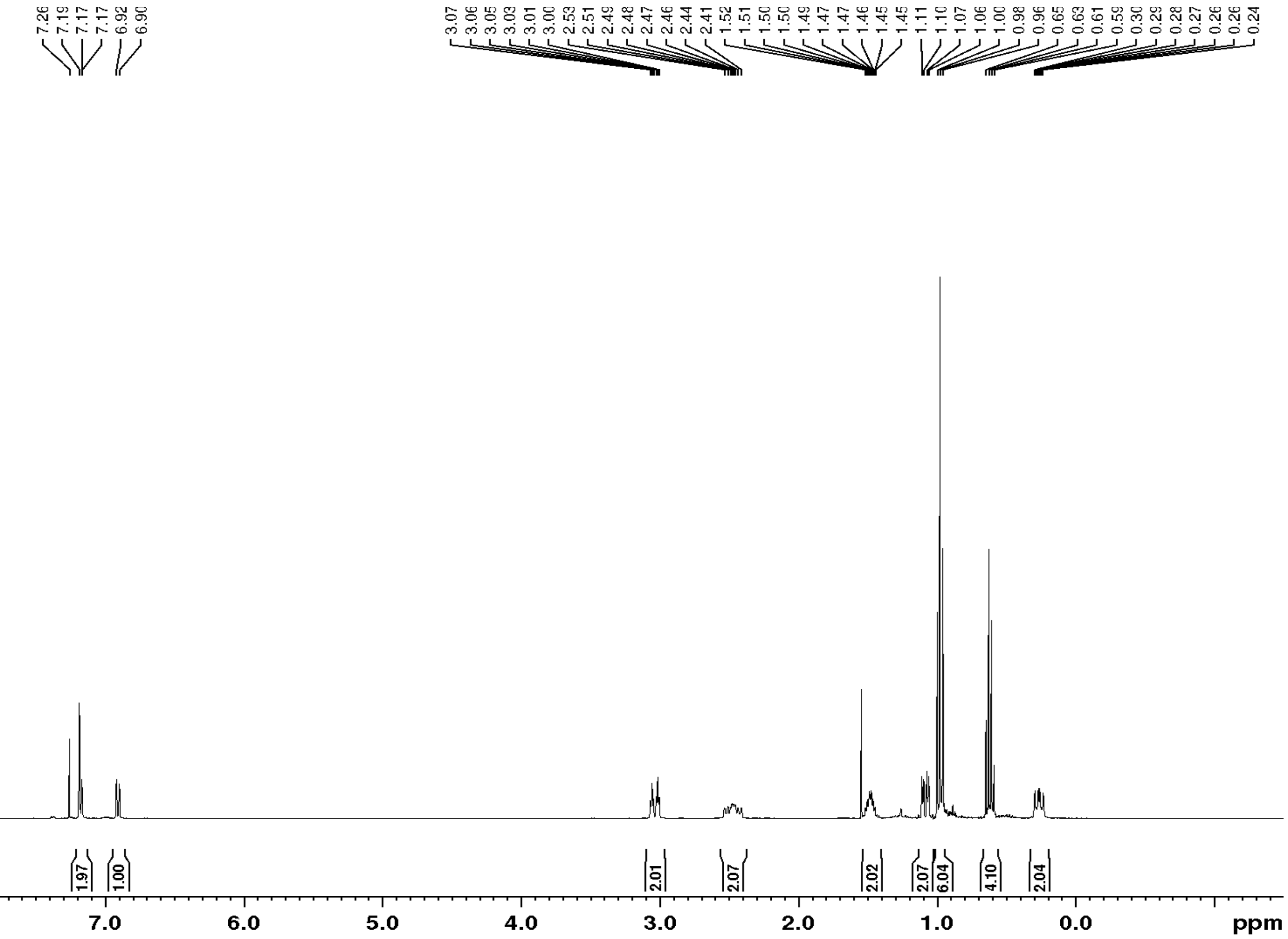

Figure S35. **$^{13}C\{^{1}H\}$ NMR** spectrum (101 MHz, $CDCl_3$, 298 K) of **3ka** from the reaction of VCP **1k** and Et_2SiH_2 (**2a**) using $Ph_3C^+[B(C_6F_5)_4]^-$ as initiator.

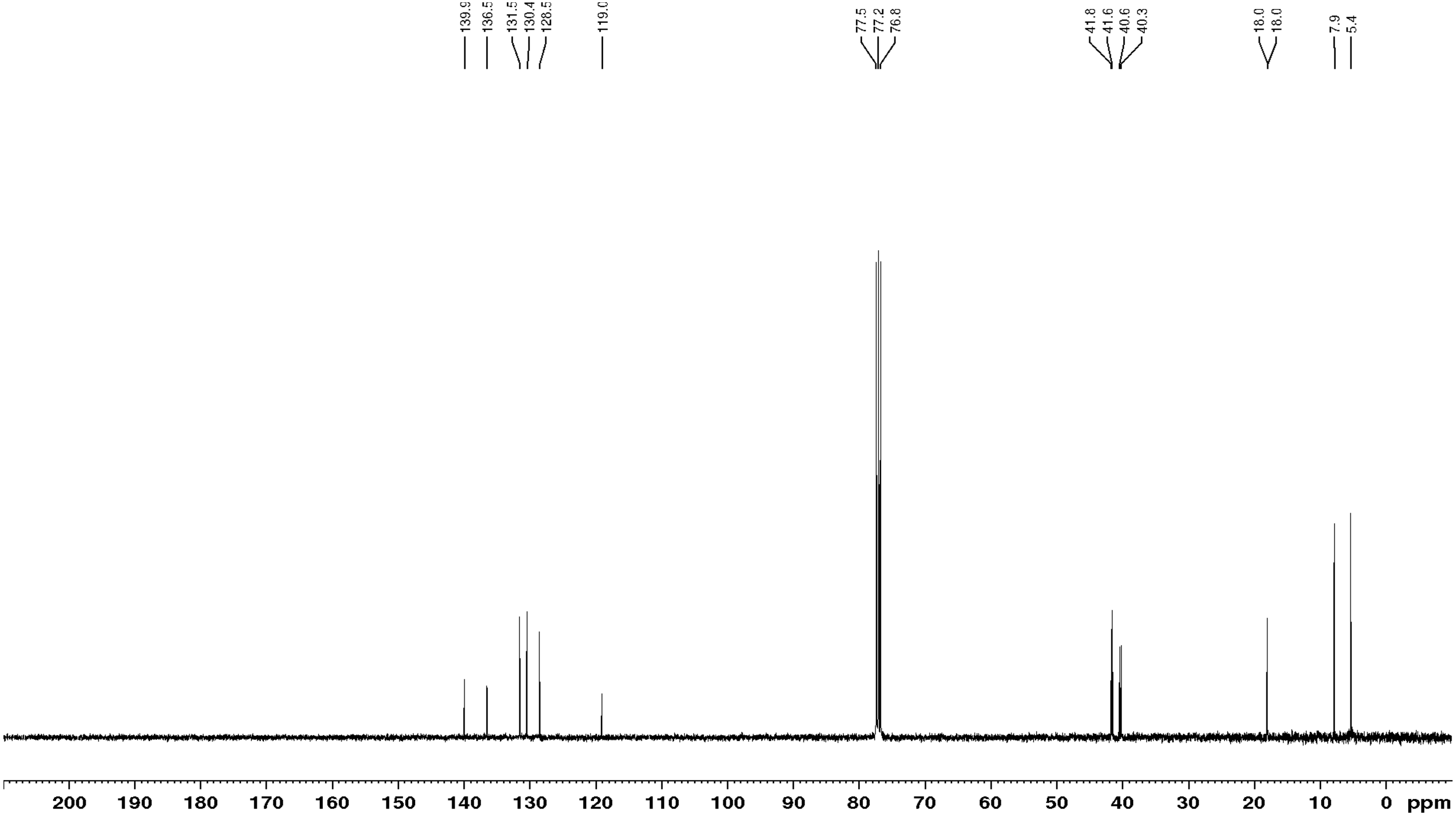

Figure S36. **$^{1}H/^{29}Si$ HMQC NMR** spectrum (500/99 MHz, $CDCl_3$, 298 K, optimized for *J* = 7 Hz) of **3ka** from the reaction of VCP **1k** and Et_2SiH_2 (**2a**) using $Ph_3C^+[B(C_6F_5)_4]^-$ as initiator.

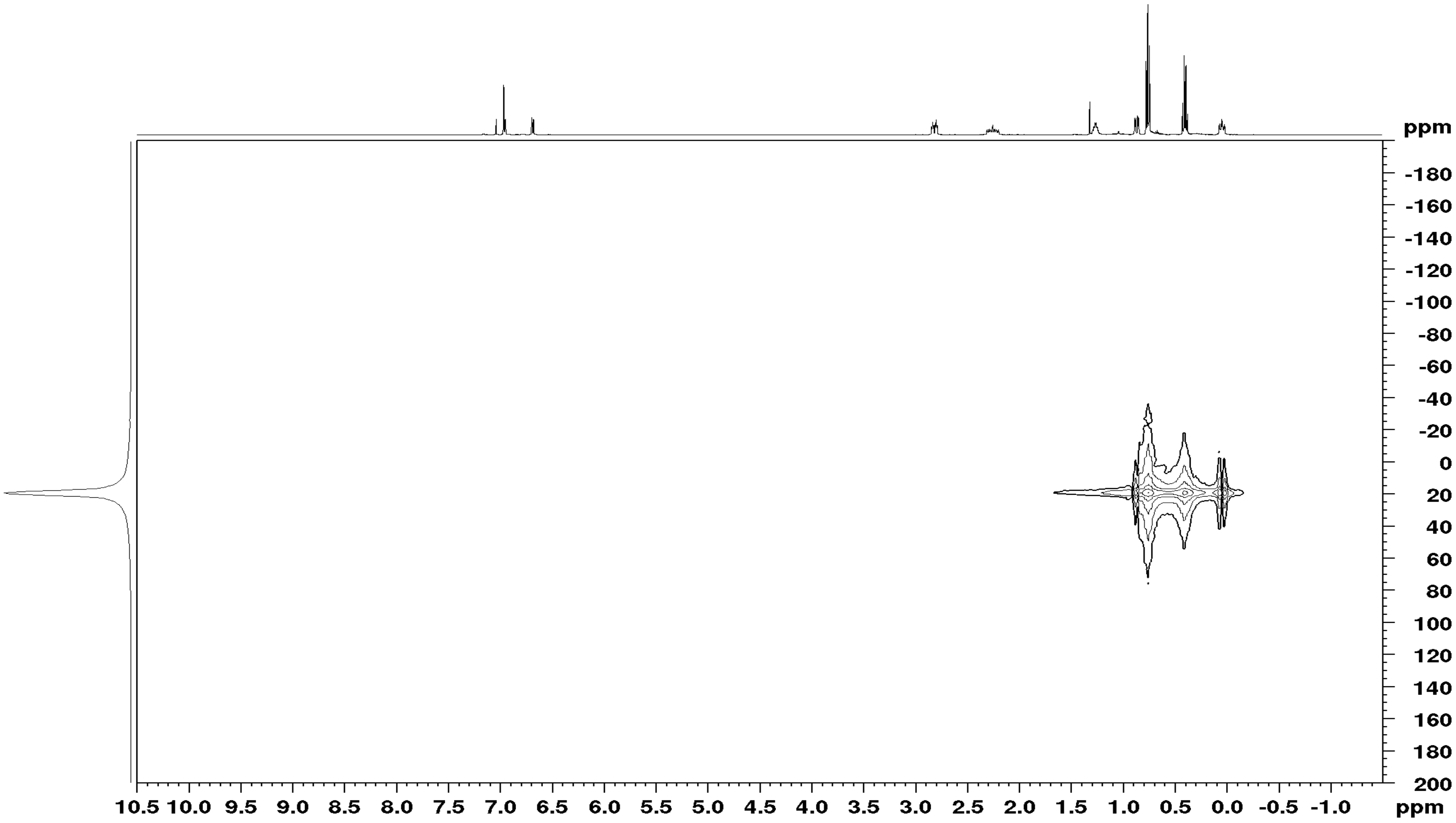

Figure S37. ^{1}**H NMR** spectrum (500 MHz, $CDCl_3$, 298 K) of **3la/3la'** from the reaction of VCP **1l** and Et_2SiH_2 (**2a**) using $Ph_3C^+[B(C_6F_5)_4]^-$ as initiator.

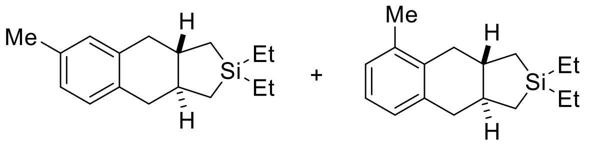

3la **3la'**

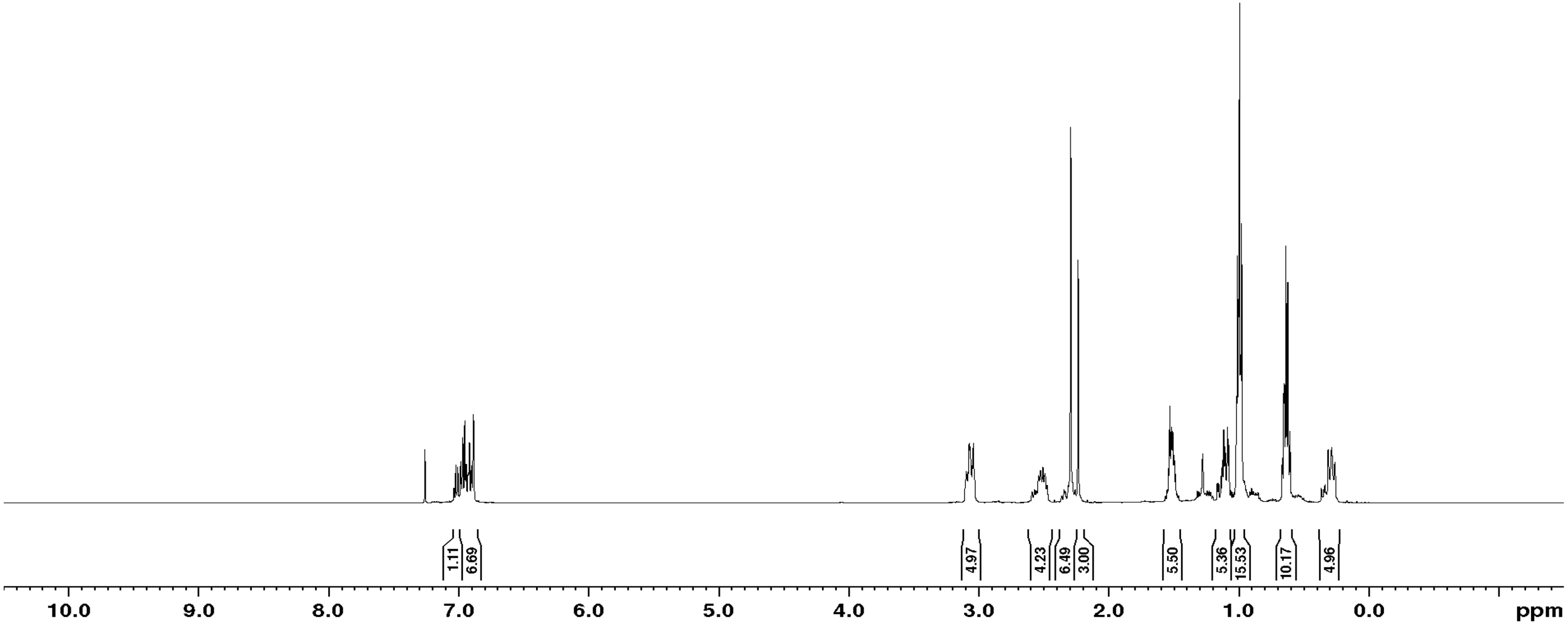

Figure S38. [1]**H NMR** spectrum (500 MHz, $CDCl_3$, 298 K) of **3ba**, **3la/3la'** and **3ca** from the reaction of VCP **1l** and Et_2SiH_2 (**2a**) using $Ph_3C^+[B(C_6F_5)_4]^-$ as initiator (# for **3ba** and * for **3ca**).

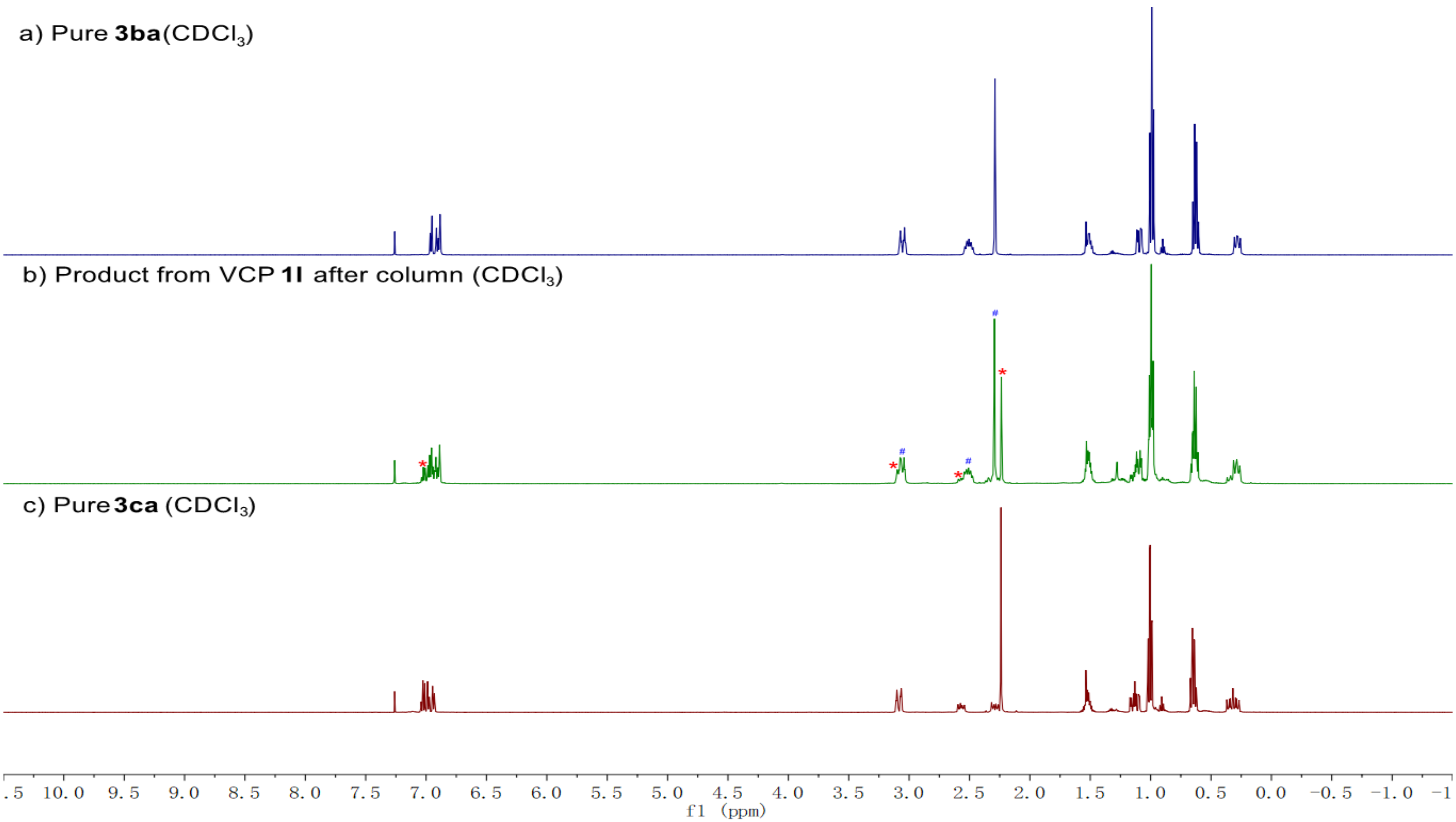

Figure S39. **^{1}H NMR** spectrum (500 MHz, $CDCl_3$, 298 K) of **3ma/3ma'** from the reaction of VCP **1m** and Et_2SiH_2 (**2a**) using $Ph_3C^+[B(C_6F_5)_4]^-$ as initiator.

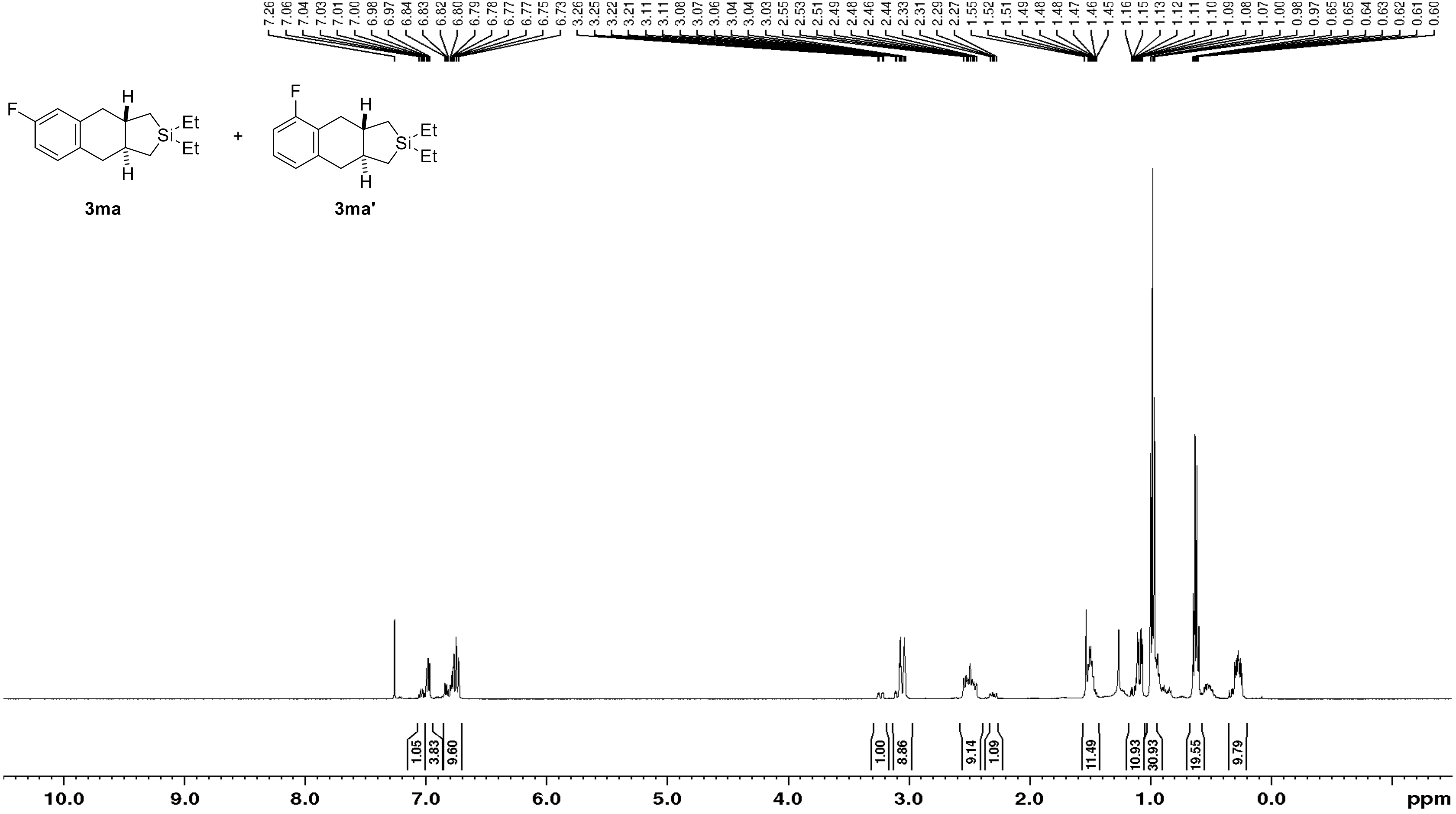

Figure S40. [1]**H NMR** spectrum (500 MHz, $CDCl_3$, 298 K) of **3ga**, **3ma/3ma'** and **3ha** from the reaction of VCP **1l** and Et_2SiH_2 (**2a**) using $Ph_3C^+[B(C_6F_5)_4]^-$ as initiator (* for **3ga** and # for **3ha**).

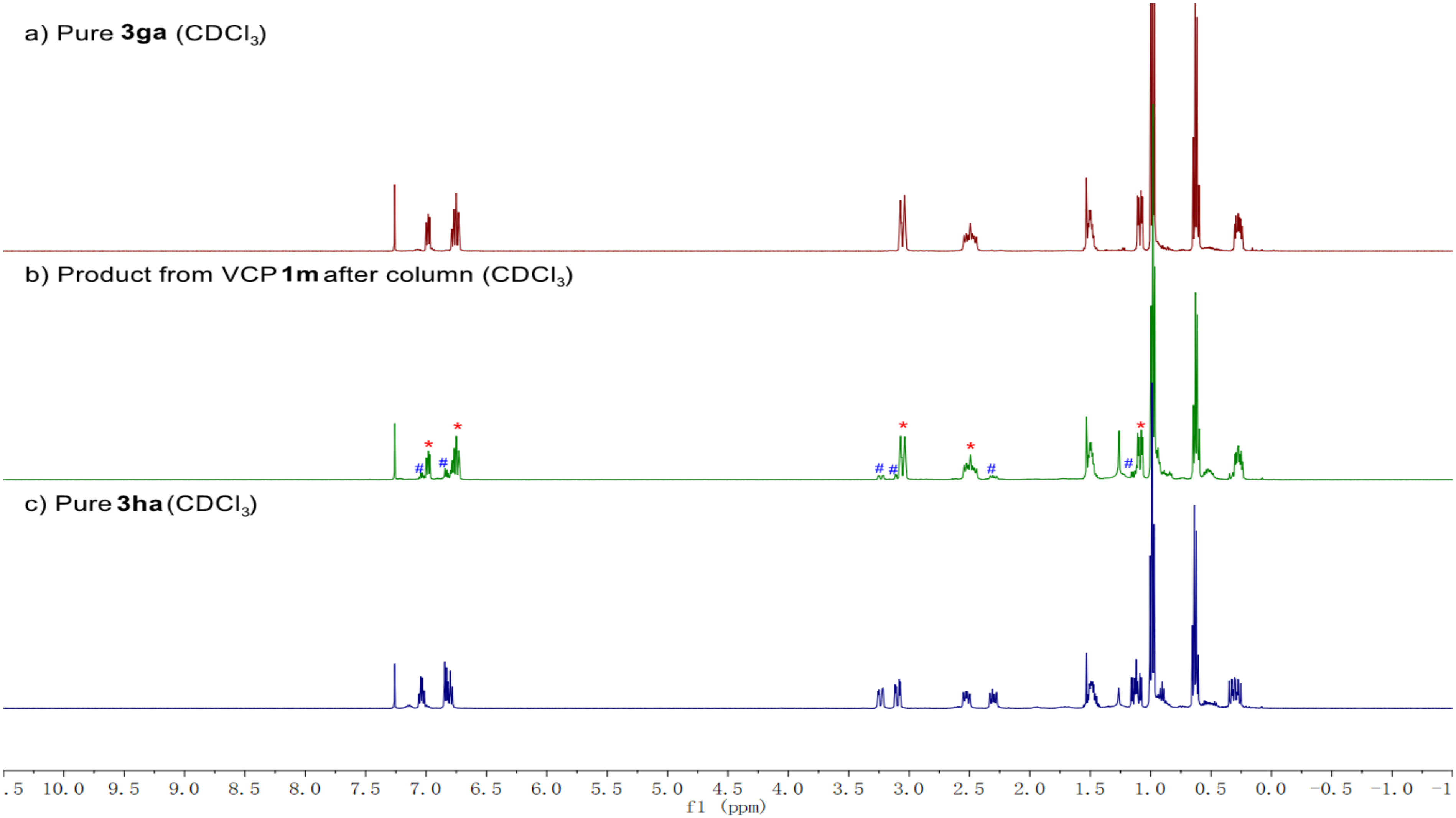

Figure S41. **^{1}H NMR** spectrum (500 MHz, $CDCl_3$, 298 K) of **3na/3na'** from the reaction of VCP **1n** and Et_2SiH_2 (**2a**) using $Ph_3C^+[B(C_6F_5)_4]^-$ as initiator.

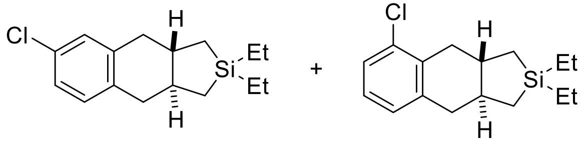

3na **3na'**

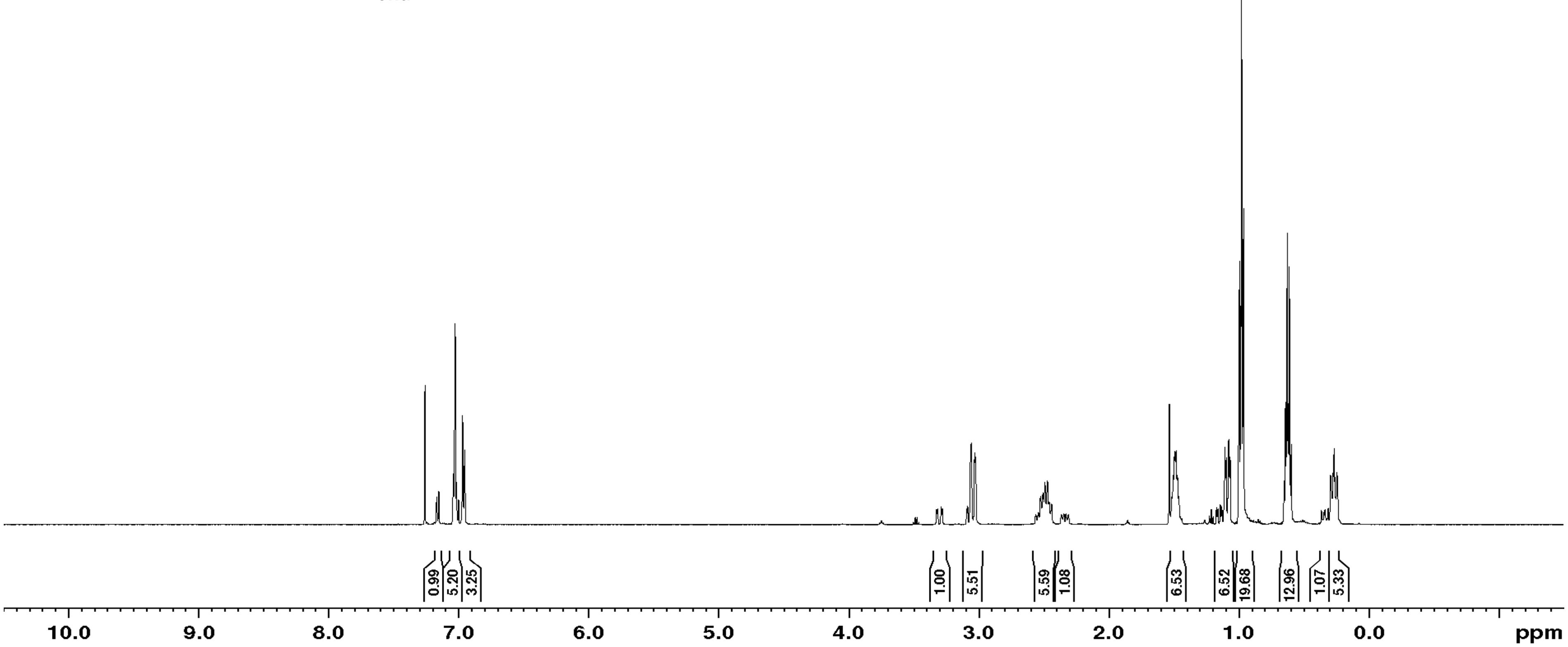

Figure S42. [1]**H NMR** spectrum (500 MHz, $CDCl_3$, 298 K) of **3ia**, **3na/3na'** and **3ja** from the reaction of VCP **1n** and Et_2SiH_2 (**2a**) using $Ph_3C^+[B(C_6F_5)_4]^-$ as initiator (* for **3ia** and # for **3ja**).

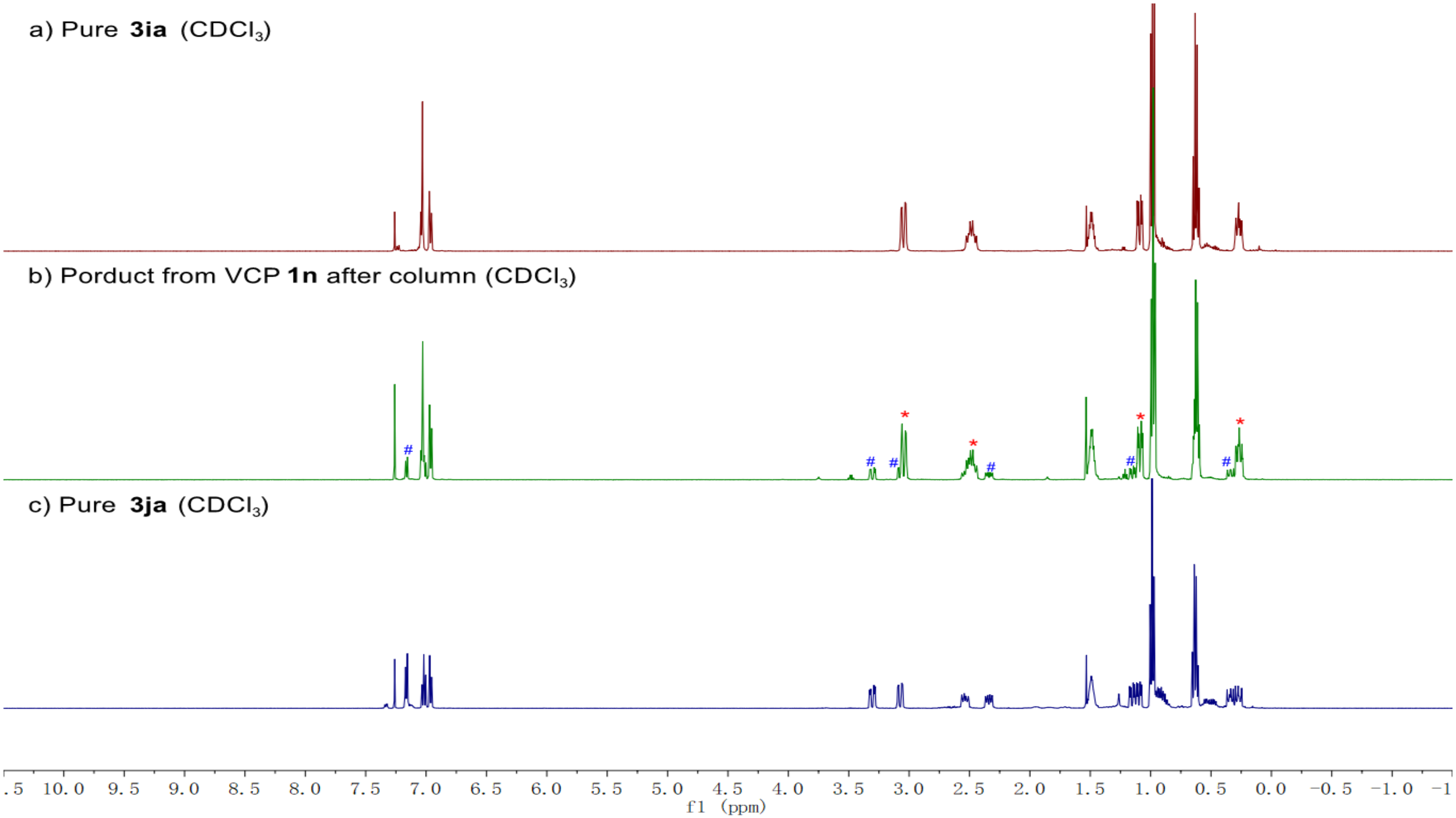

Figure S43. [1]**H NMR** spectrum (500 MHz, $CDCl_3$, 298 K) of **3oa/3oa'** from the reaction of VCP **1o** and Et_2SiH_2 (**2a**) using $Ph_3C^+[B(C_6F_5)_4]^-$ as initiator.

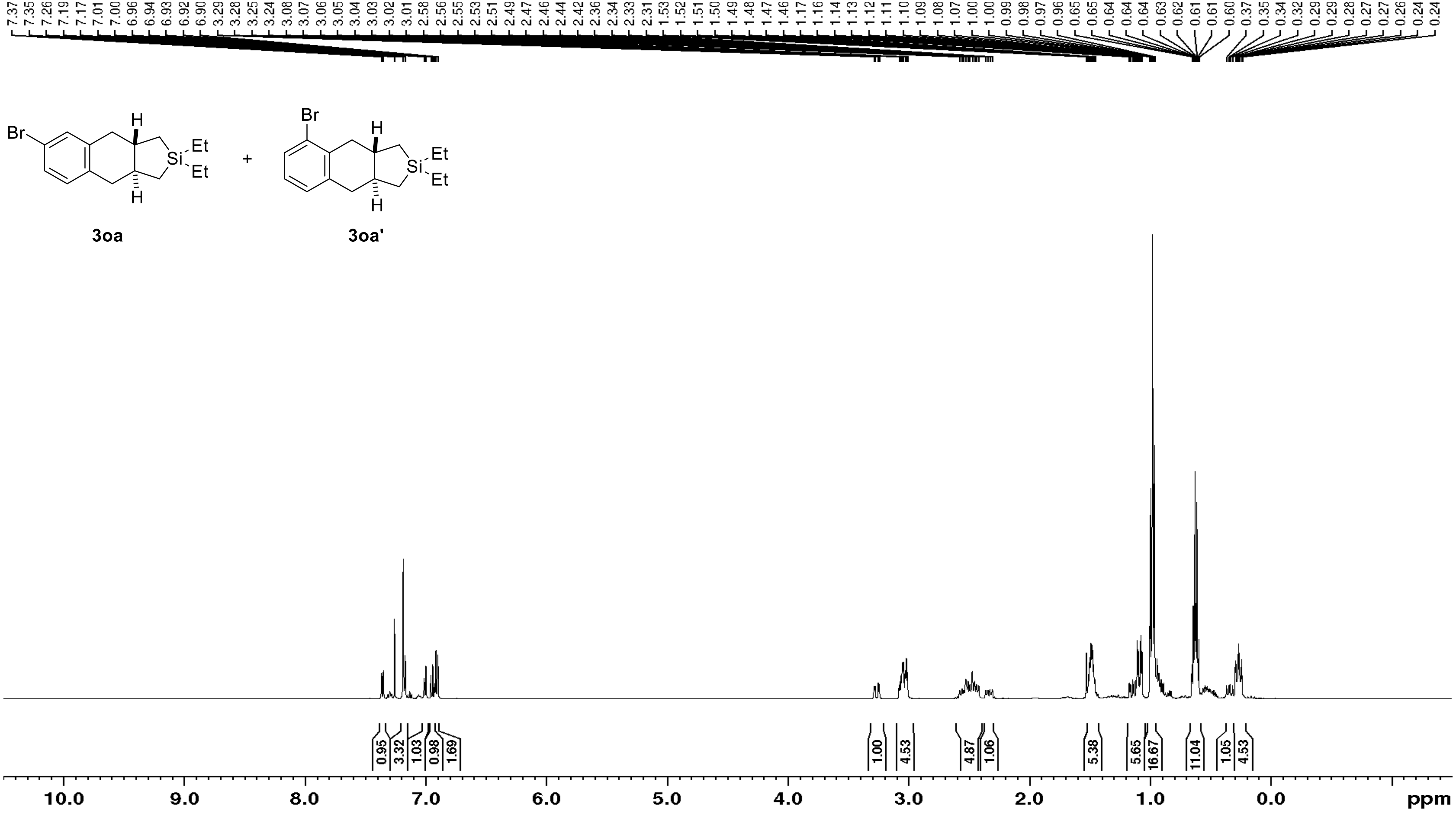

Figure S44. [1]**H NMR** spectrum (500 MHz, $CDCl_3$, 298 K) of **3oa/3oa'** and **3ka** from the reaction of VCP **1o** and Et_2SiH_2 (**2a**) using $Ph_3C^+[B(C_6F_5)_4]^-$ as initiator (* for **3ka** and # for **3oa'**).

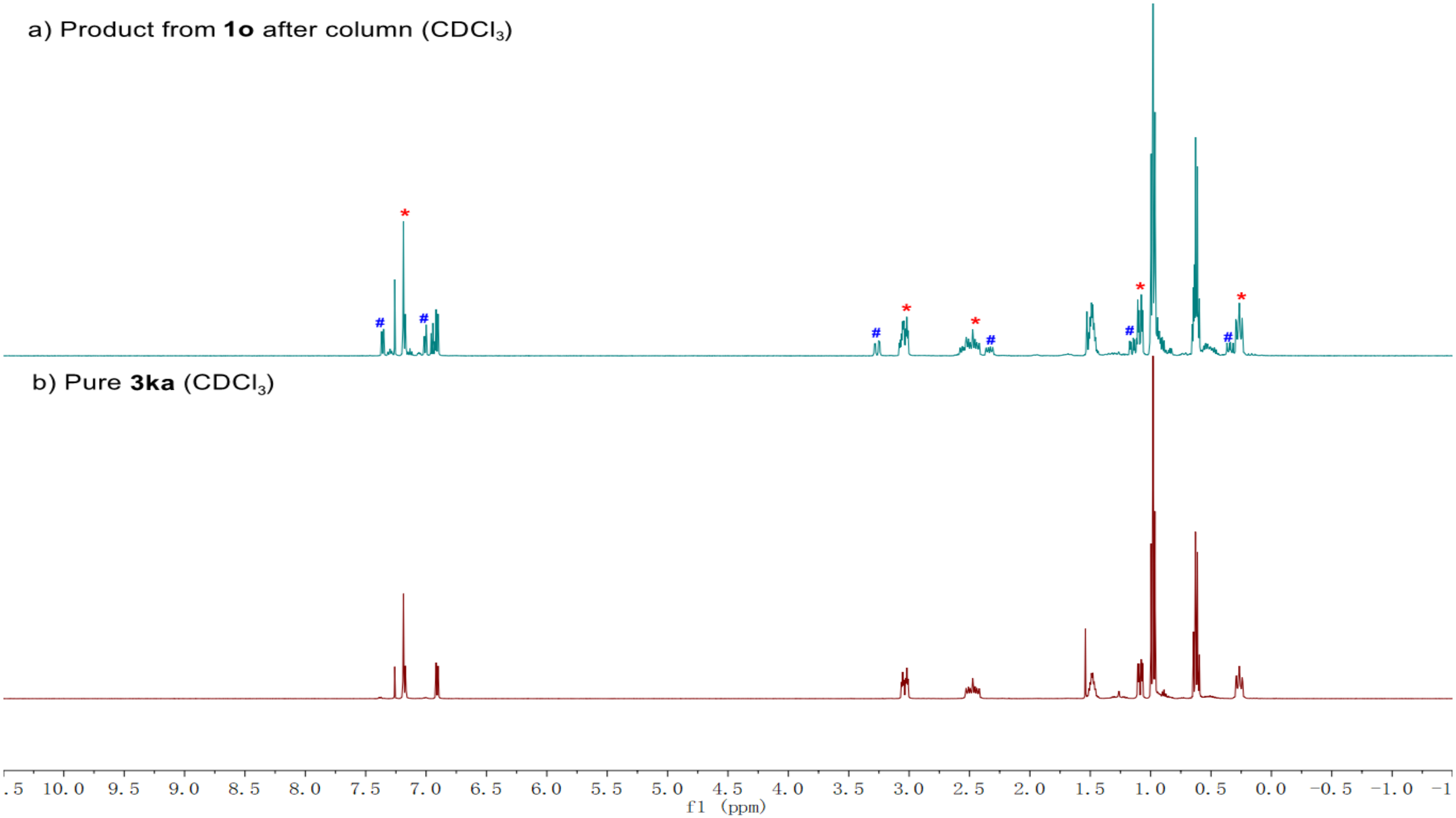

Figure S45. **^{1}H NMR** spectrum (500 MHz, $CDCl_3$, 298 K) of **3pa/3pa'** from the reaction of VCP **1p** and Et_2SiH_2 (**2a**) using $Ph_3C^+[B(C_6F_5)_4]^-$ as initiator (* for **3pa**).

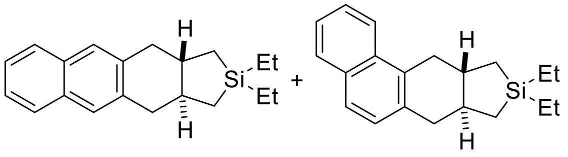

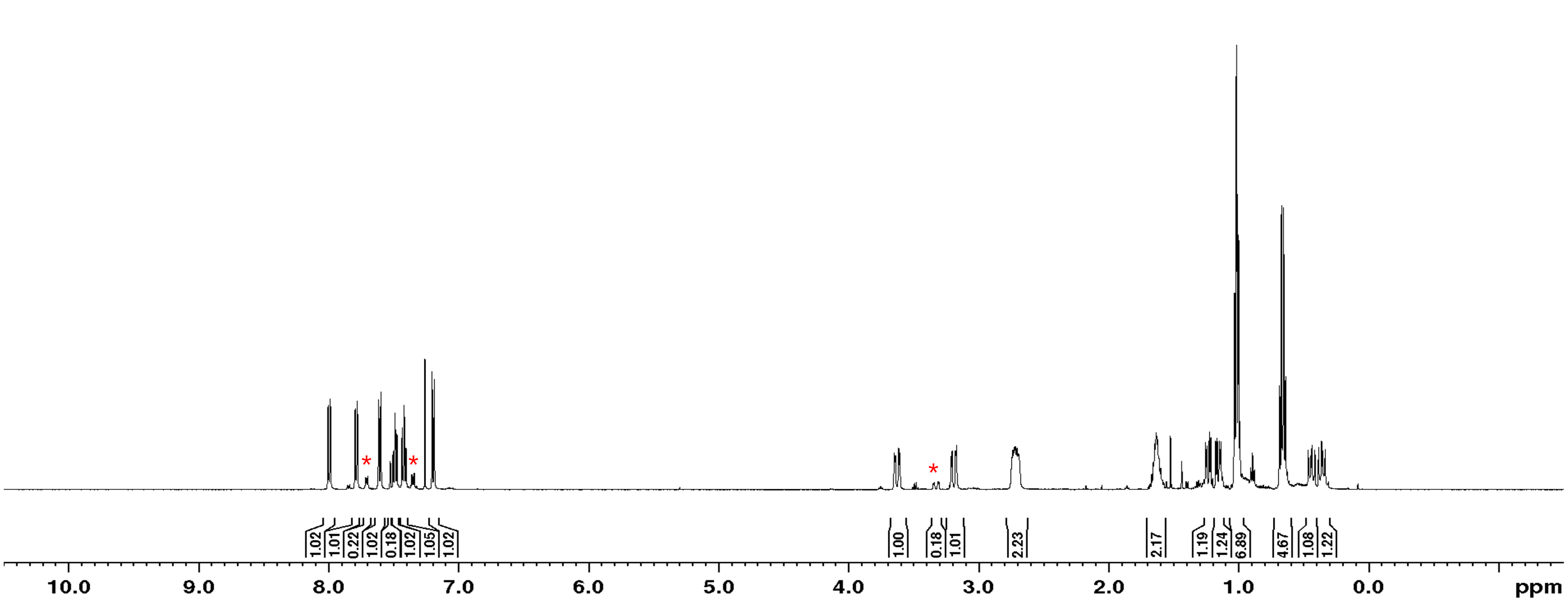

Figure S46. **$^{13}C\{^{1}H\}$ NMR** spectrum (126 MHz, $CDCl_3$, 298 K) of **3pa/3pa'** from the reaction of VCP **1p** and Et_2SiH_2 (**2a**) using $Ph_3C^+[B(C_6F_5)_4]^-$ as initiator.

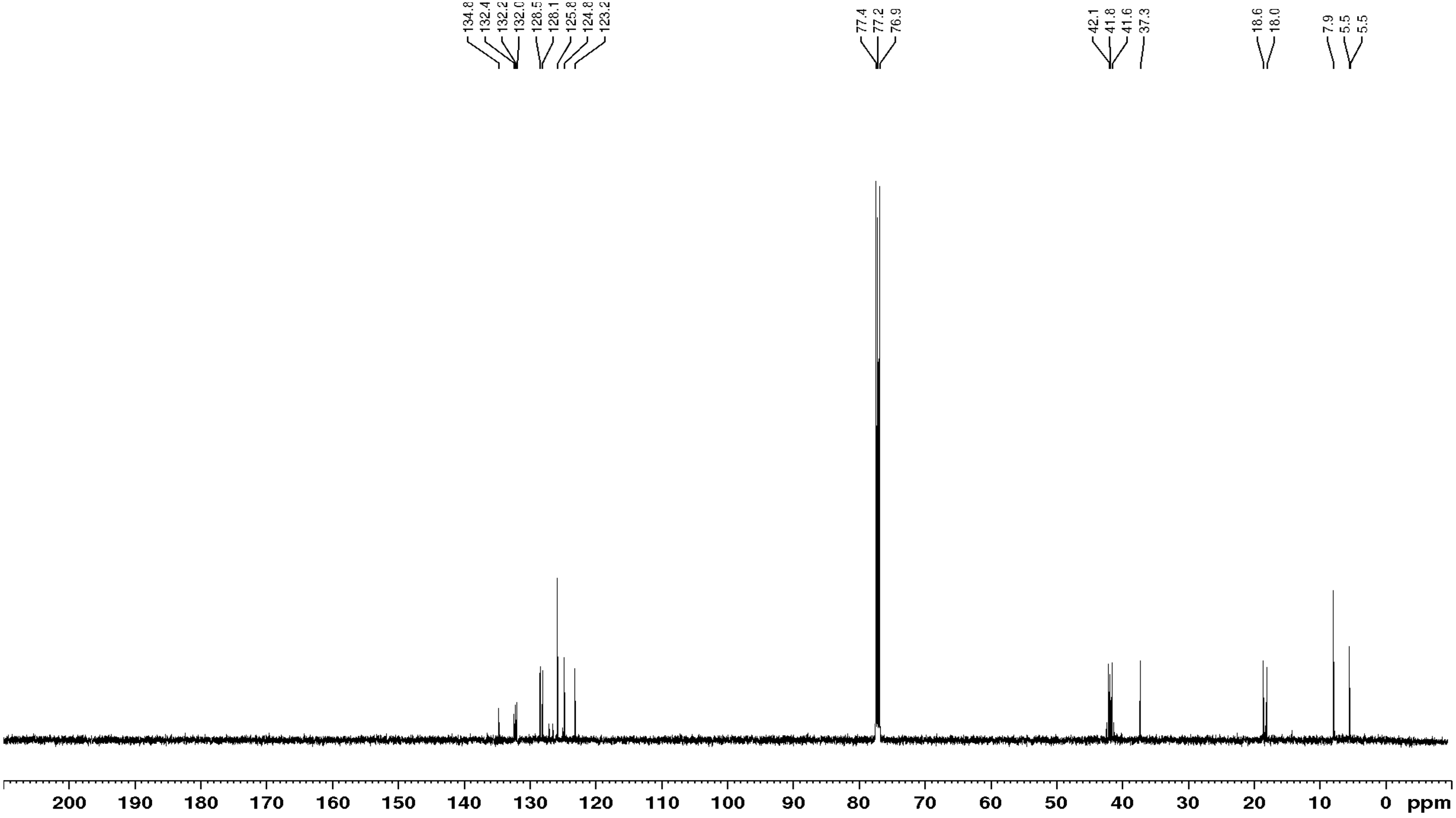

Figure S47. **$^1H/^{29}Si$ HMQC NMR** spectrum (500/99 MHz, $CDCl_3$, 298 K, optimized for J = 7 Hz) of **3pa/3pa'** from the reaction of VCP **1p** and Et_2SiH_2 (**2a**) using $Ph_3C^+[B(C_6F_5)_4]^-$ as initiator.

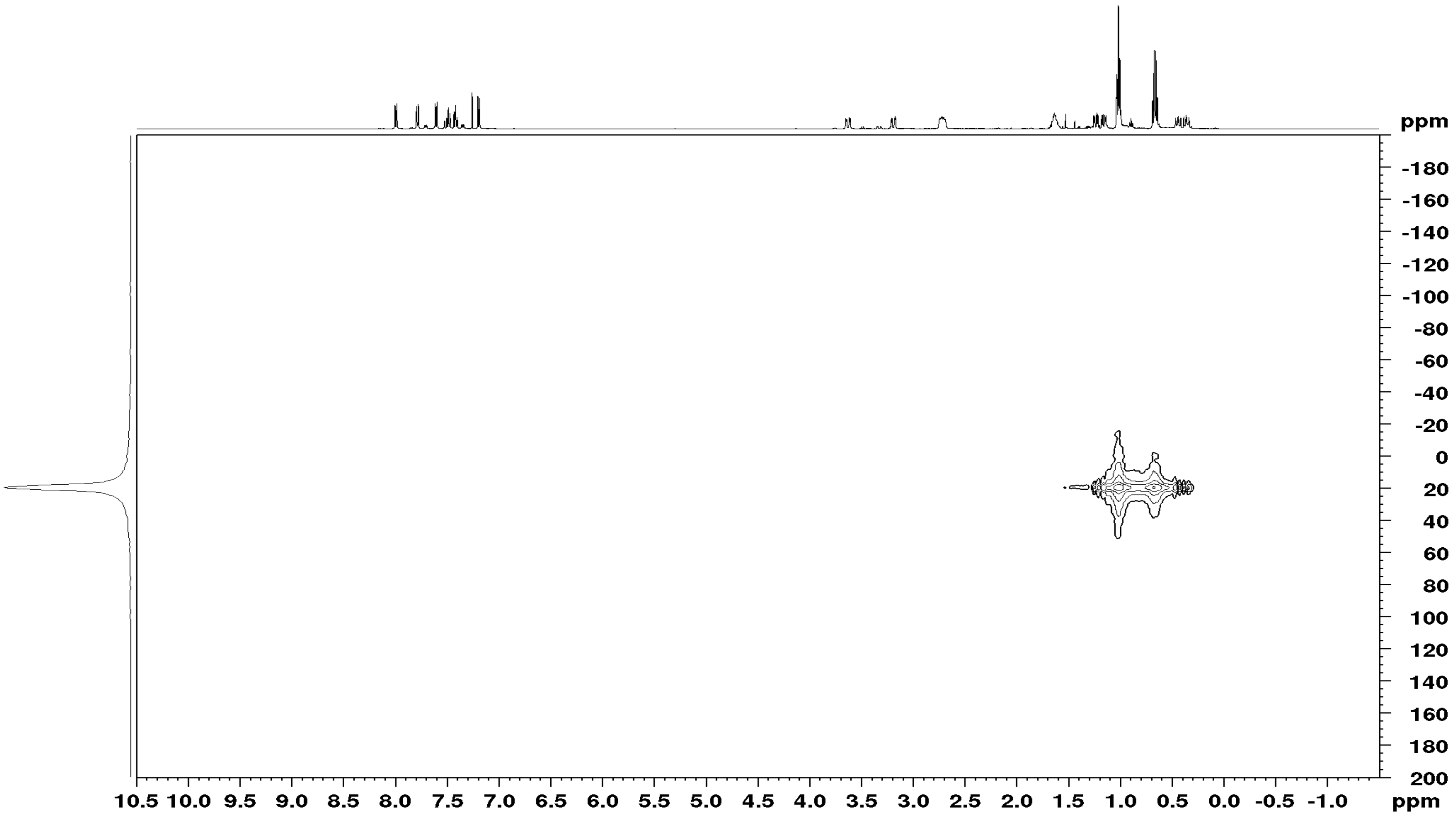

Figure S48. ^{1}**H NMR** spectrum (500 MHz, $CDCl_3$, 298 K) of **3qa** from the reaction of VCP **1q** and Et_2SiH_2 (**2a**) using $Ph_3C^+[B(C_6F_5)_4]^-$ as initiator.

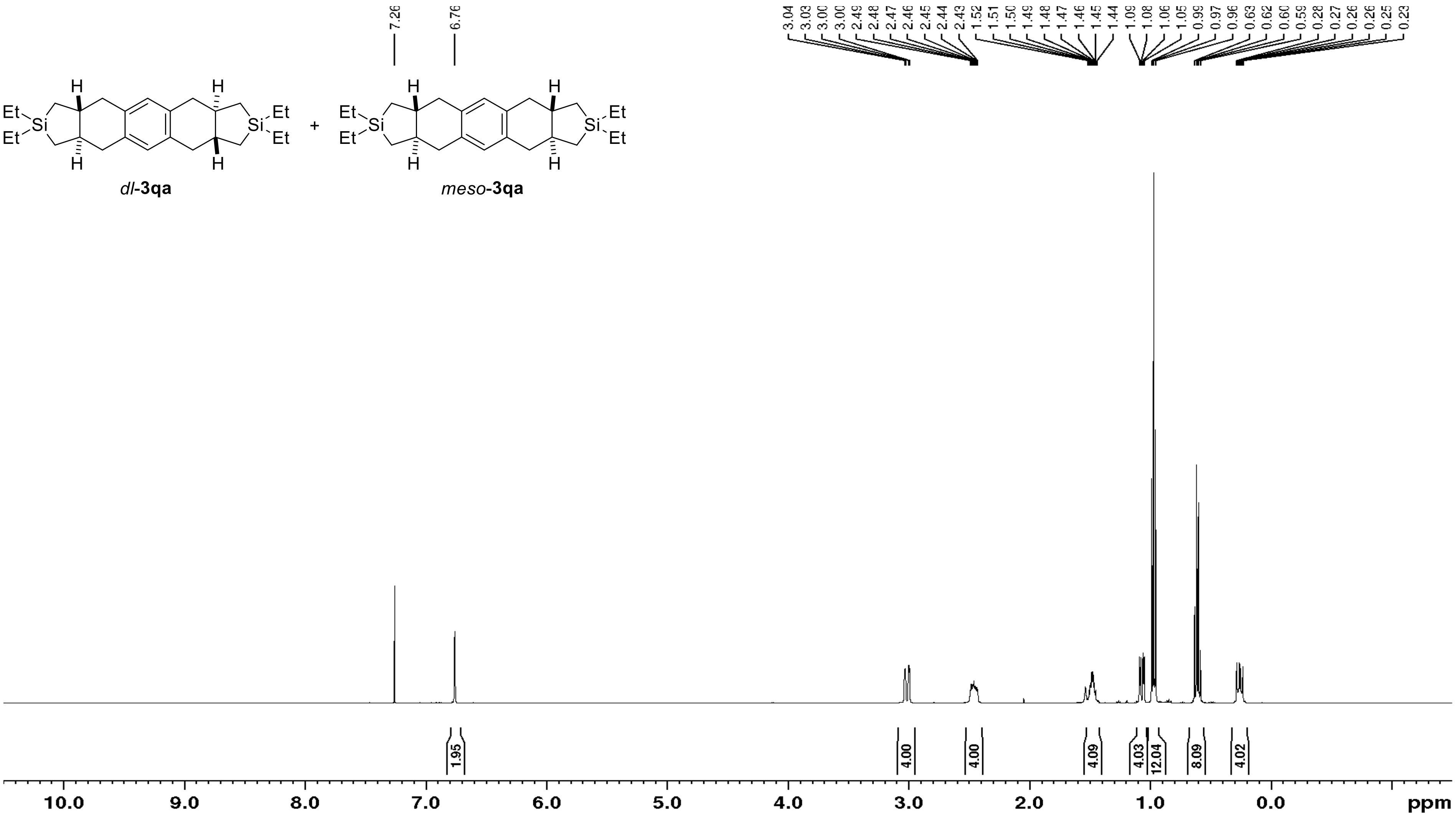

Figure S49. **$^{13}C\{^1H\}$ NMR** spectrum (126 MHz, $CDCl_3$, 298 K) of **3qa** from the reaction of VCP **1q** and Et_2SiH_2 (**2a**) using $Ph_3C^+[B(C_6F_5)_4]^-$ as initiator.

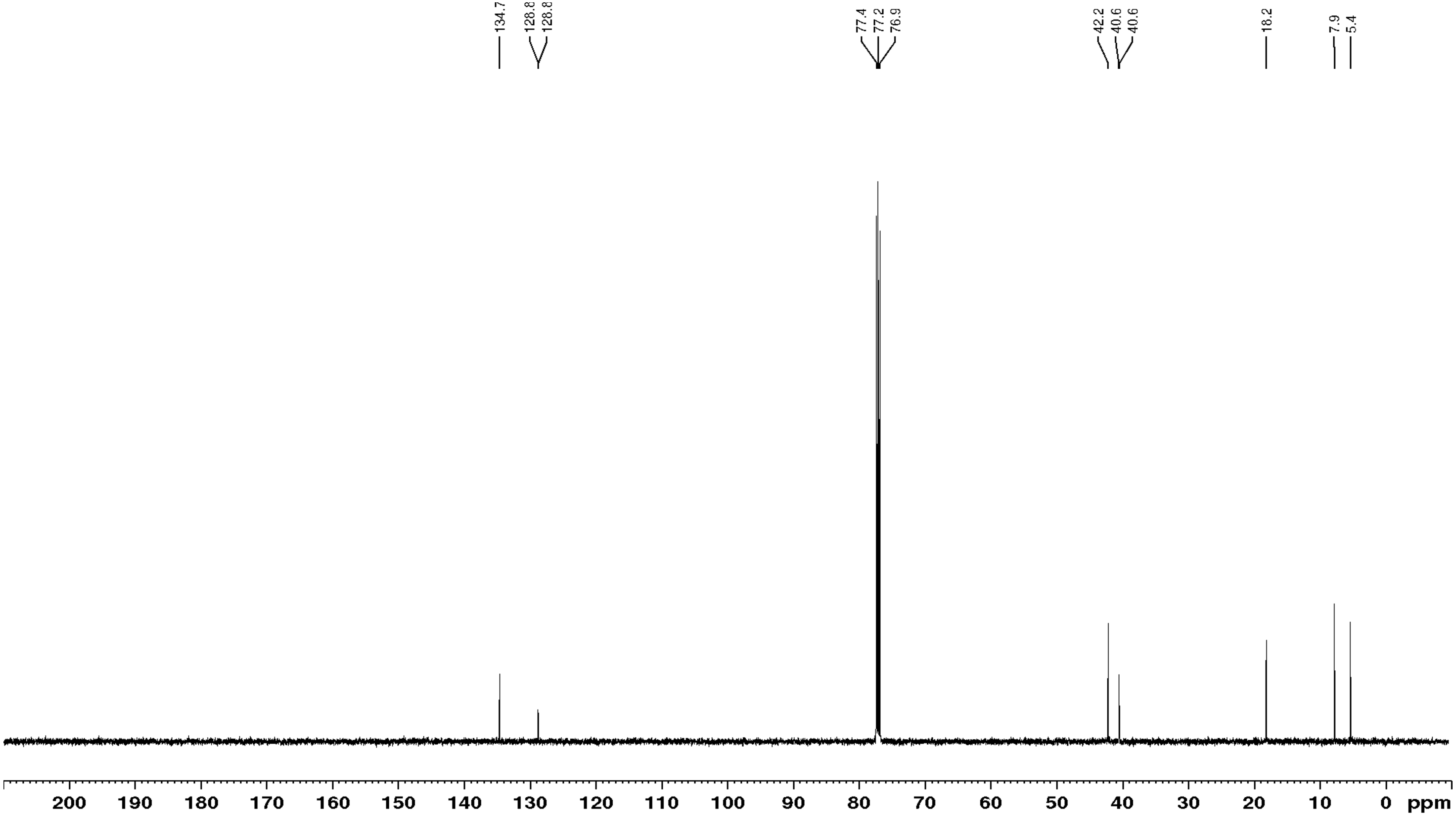

Figure S50. **$^1H/^{29}Si$ HMQC NMR** spectrum (500/99 MHz, $CDCl_3$, 298 K, optimized for *J* = 7 Hz) of **3qa** from the reaction of VCP **1q** and Et_2SiH_2 (**2a**) using $Ph_3C^+[B(C_6F_5)_4]^-$ as initiator.

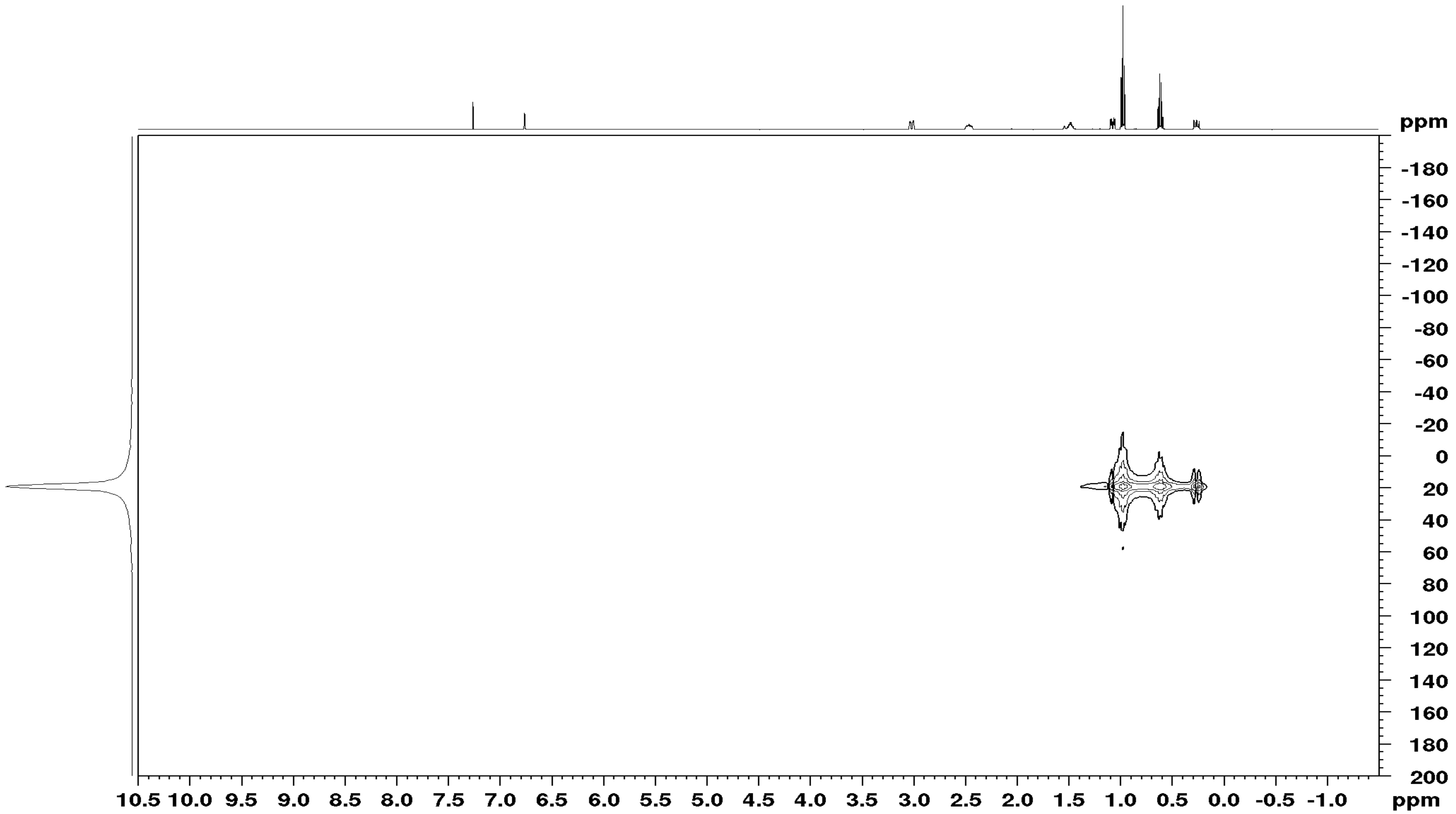

Figure S51. **^{1}H NMR spectrum** (500 MHz, $CDCl_3$, 298 K) of **3ac** from the reaction of VCP **1a** and $EtMe_2SiH$ (**2c**) using $Ph_3C^+[B(C_6F_5)_4]^-$ as initiator.

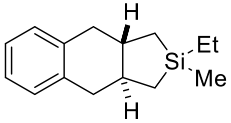

3ac

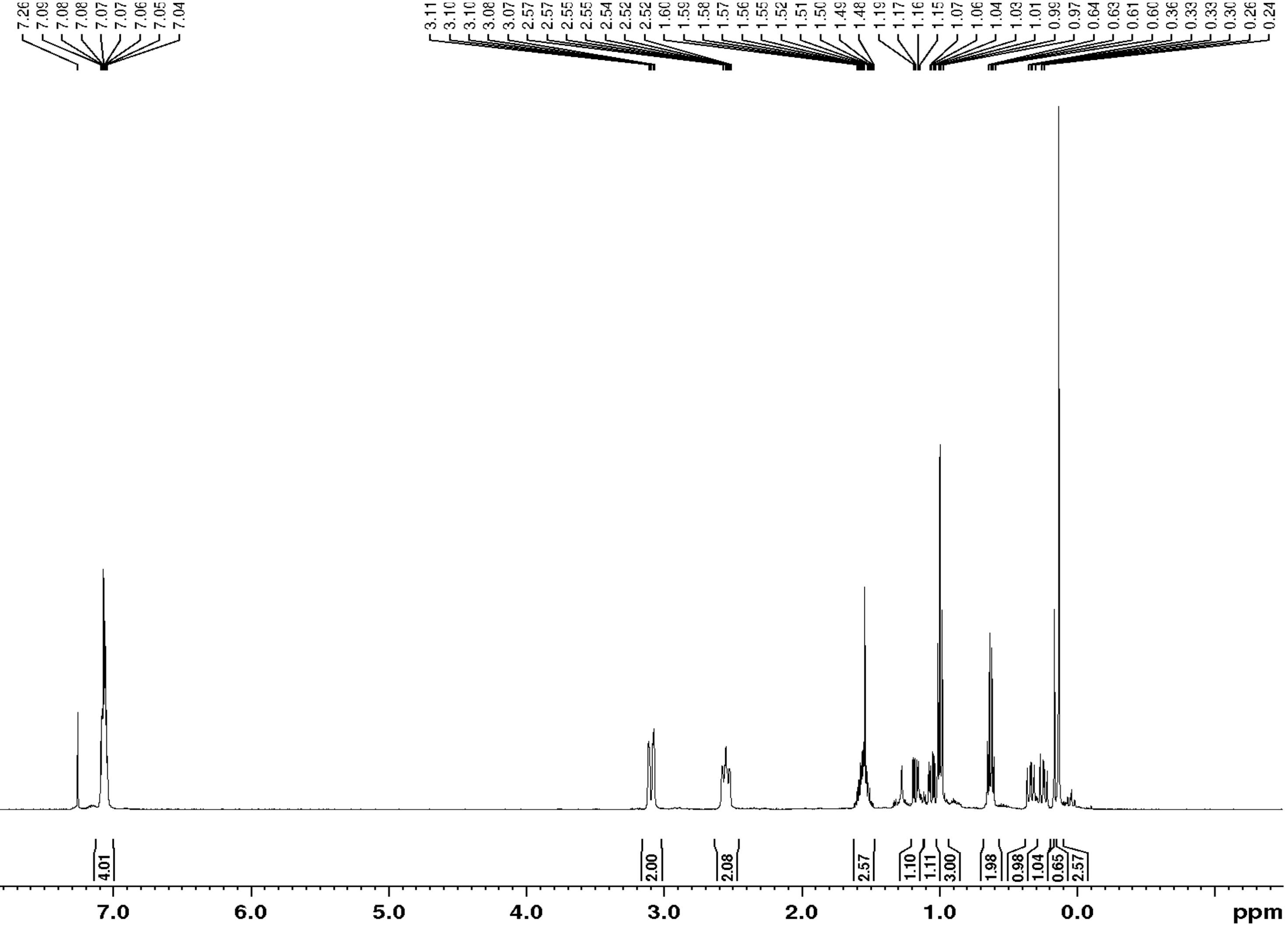

Figure S52. **$^{13}C\{^1H\}$ NMR** spectrum (101 MHz, $CDCl_3$, 298 K) of **3ac** from the reaction of VCP **1a** and $EtMe_2SiH$ (**2c**) using $Ph_3C^+[B(C_6F_5)_4]^-$ as initiator.

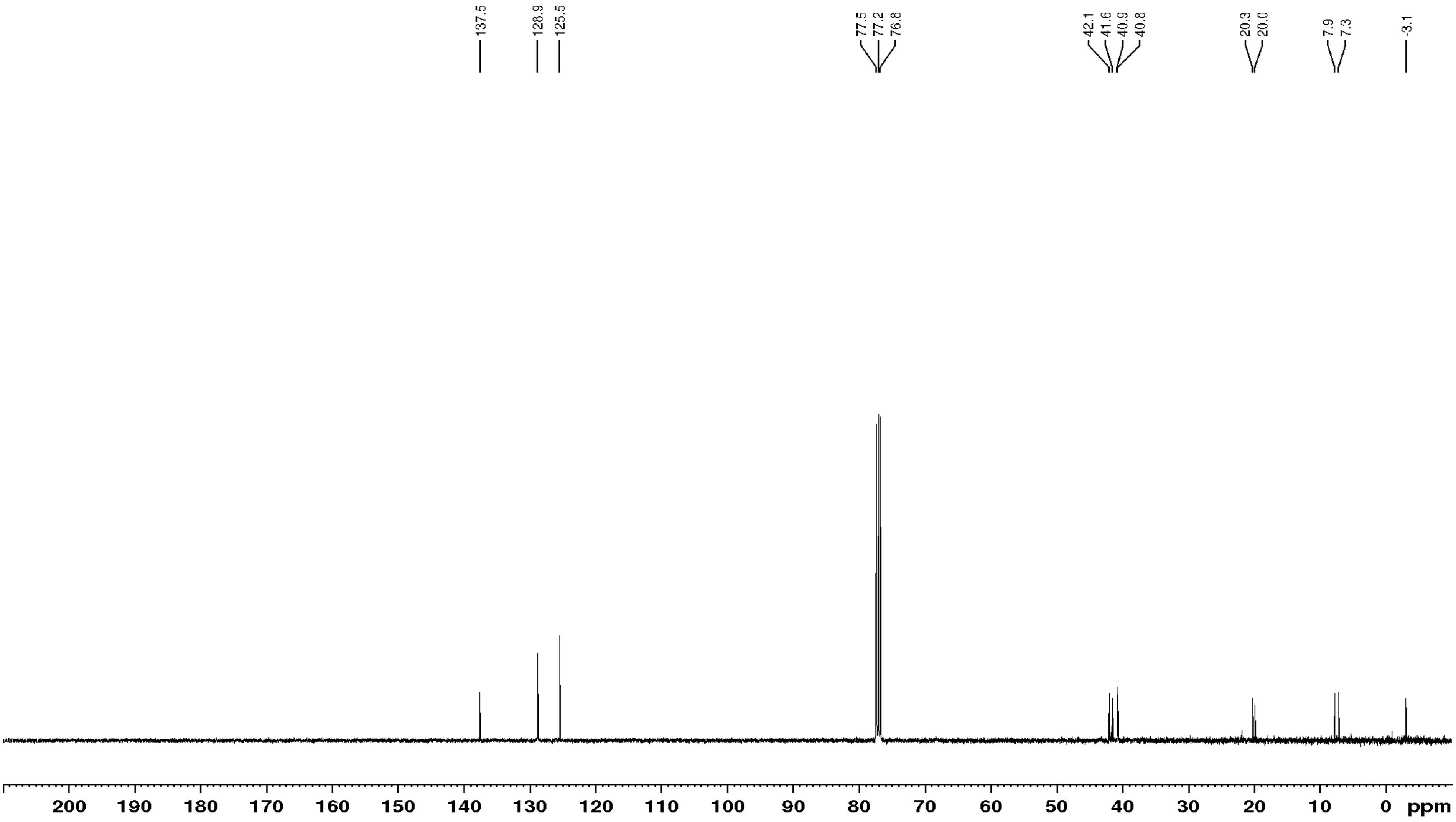

Figure S53. **^{1}H/^{29}Si HMQC NMR** spectrum (500/99 MHz, $CDCl_3$, 298 K, optimized for *J* = 7 Hz) of **3ac** from the reaction of VCP **1a** and $EtMe_2SiH$ (**2c**) using $Ph_3C^+[B(C_6F_5)_4]^-$ as initiator.

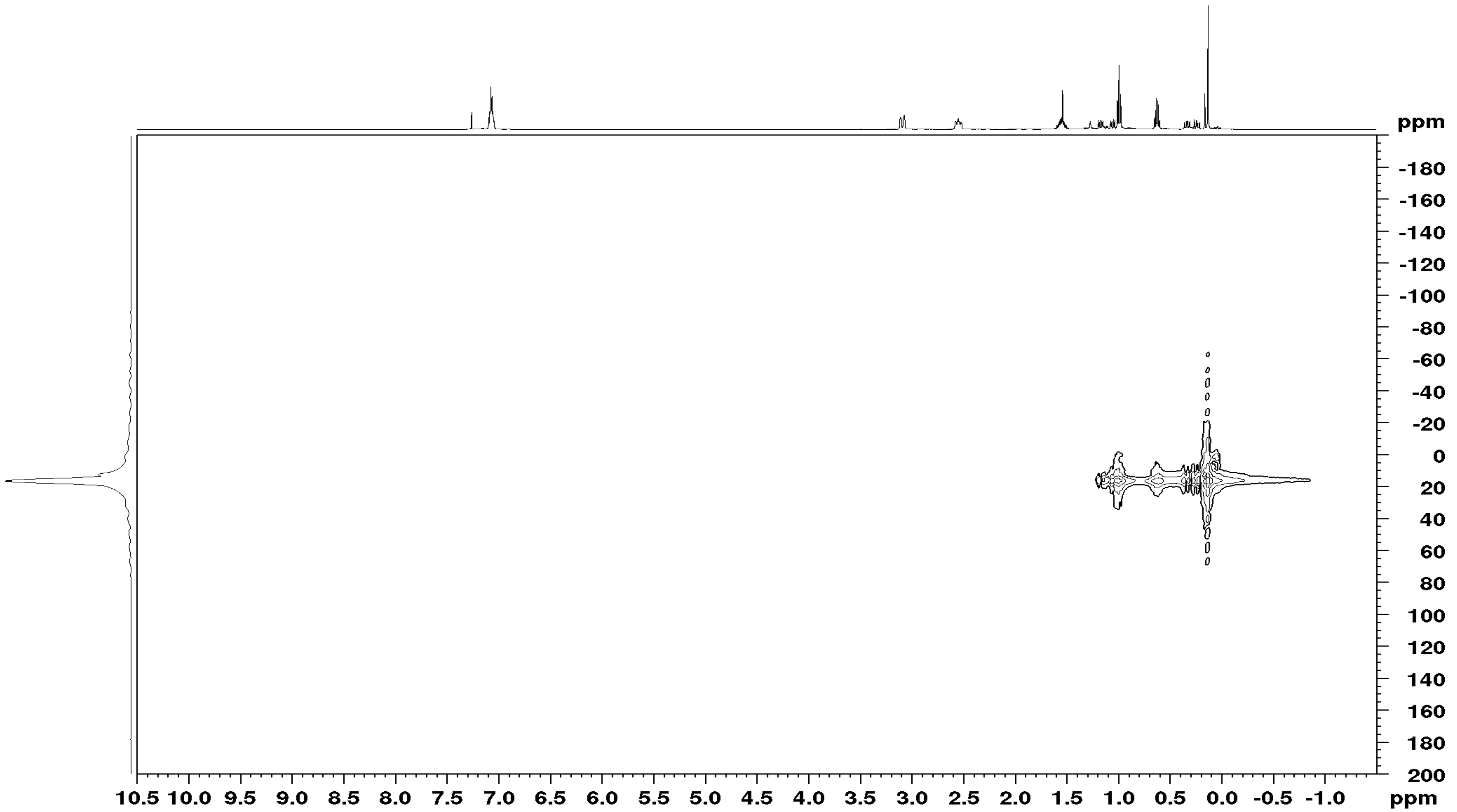

Figure S54. **^{1}H NMR** spectrum (500 MHz, $CDCl_3$, 298 K) of **3ad** from the reaction of VCP **1a** and $PhMe_2SiH$ (**2d**) using $Ph_3C^+[B(C_6F_5)_4]^-$ as initiator.

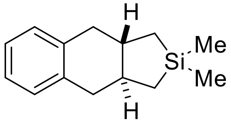

3ad

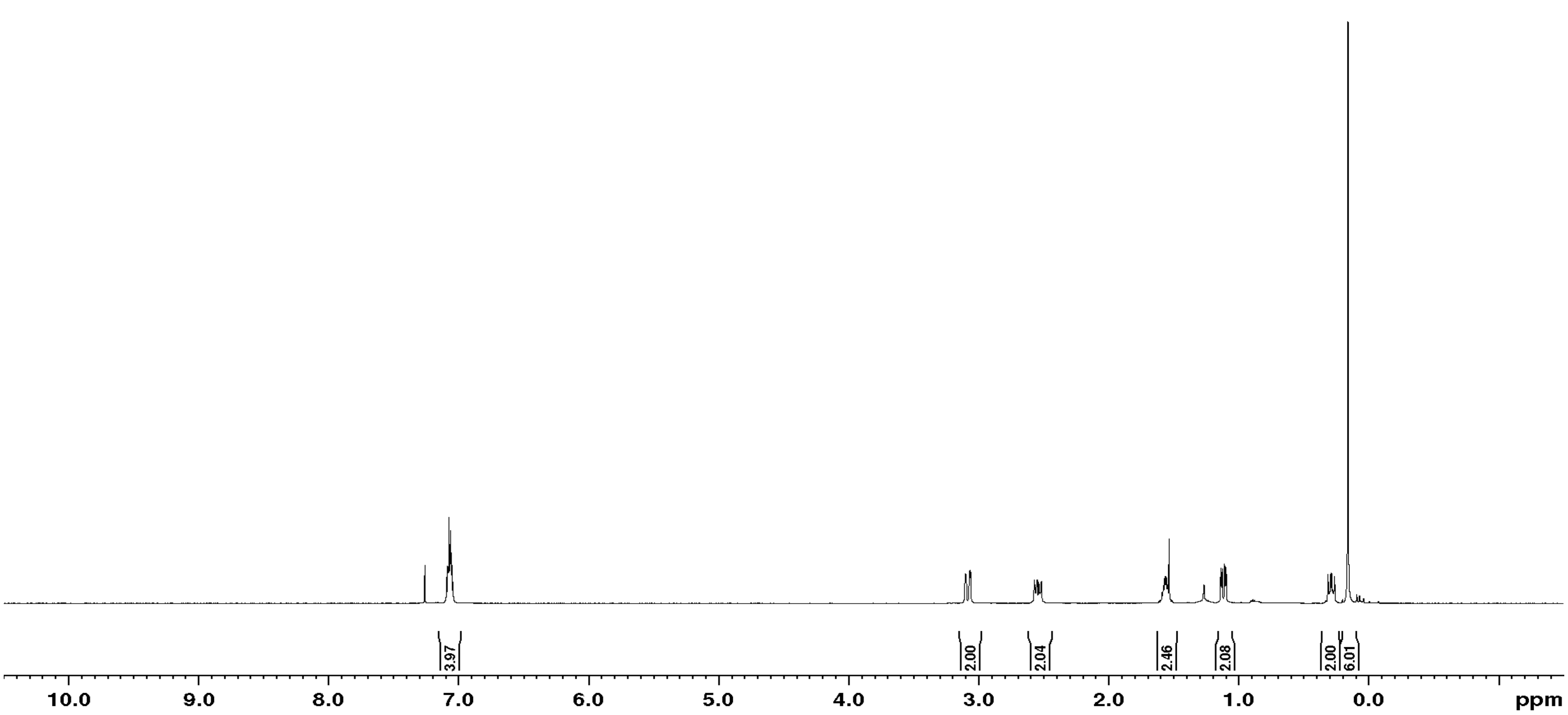

Figure S55. **$^{13}C\{^1H\}$ NMR** spectrum (101 MHz, $CDCl_3$, 298 K) of **3ad** from the reaction of VCP **1a** and $PhMe_2SiH$ (**2d**) using $Ph_3C^+[B(C_6F_5)_4]^-$ as initiator.

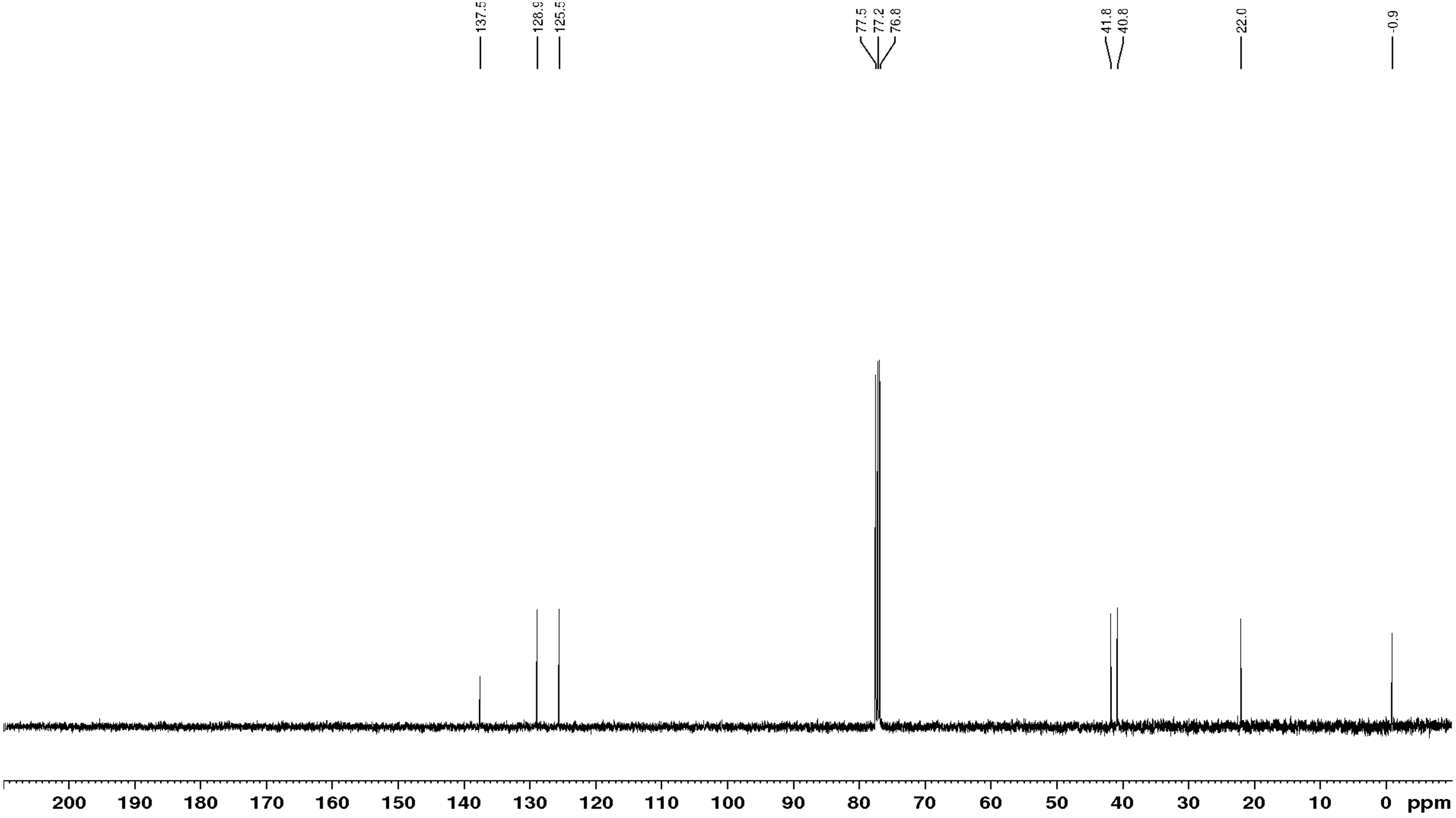

Figure S56. **$^1H/^{29}Si$ HMQC NMR** spectrum (500/99 MHz, $CDCl_3$, 298 K, optimized for *J* = 7 Hz) of **3ad** from the reaction of VCP **1a** and $PhMe_2SiH$ (**2d**) using $Ph_3C^+[B(C_6F_5)_4]^-$ as initiator.

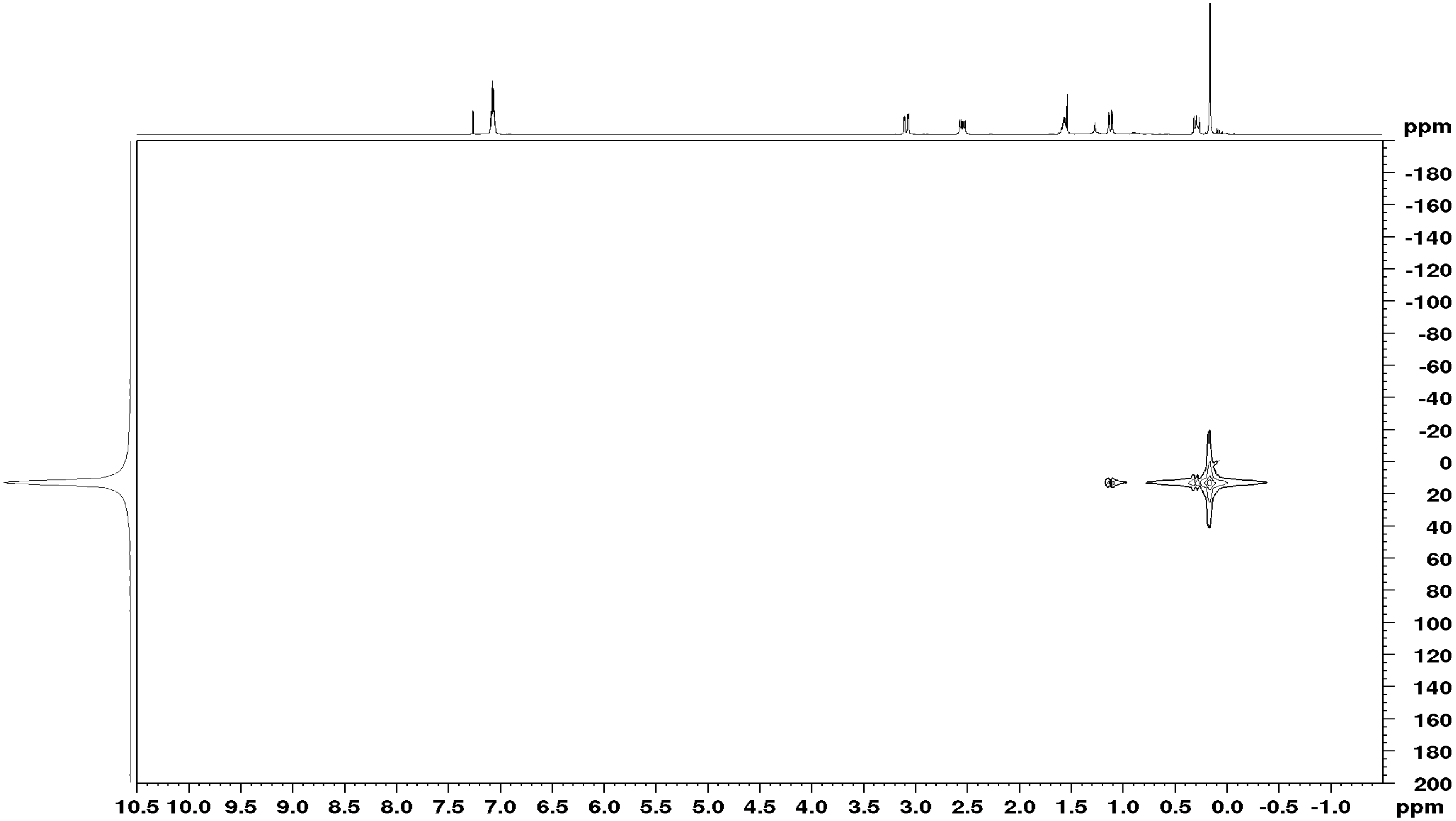

Figure S57. [1]**H NMR** spectrum (500 MHz, $CDCl_3$, 298 K) of **8a** from the reaction of VCP **7** and Et_2SiH_2 (**2a**) using $Ph_3C^+[B(C_6F_5)_4]^-$ as initiator.

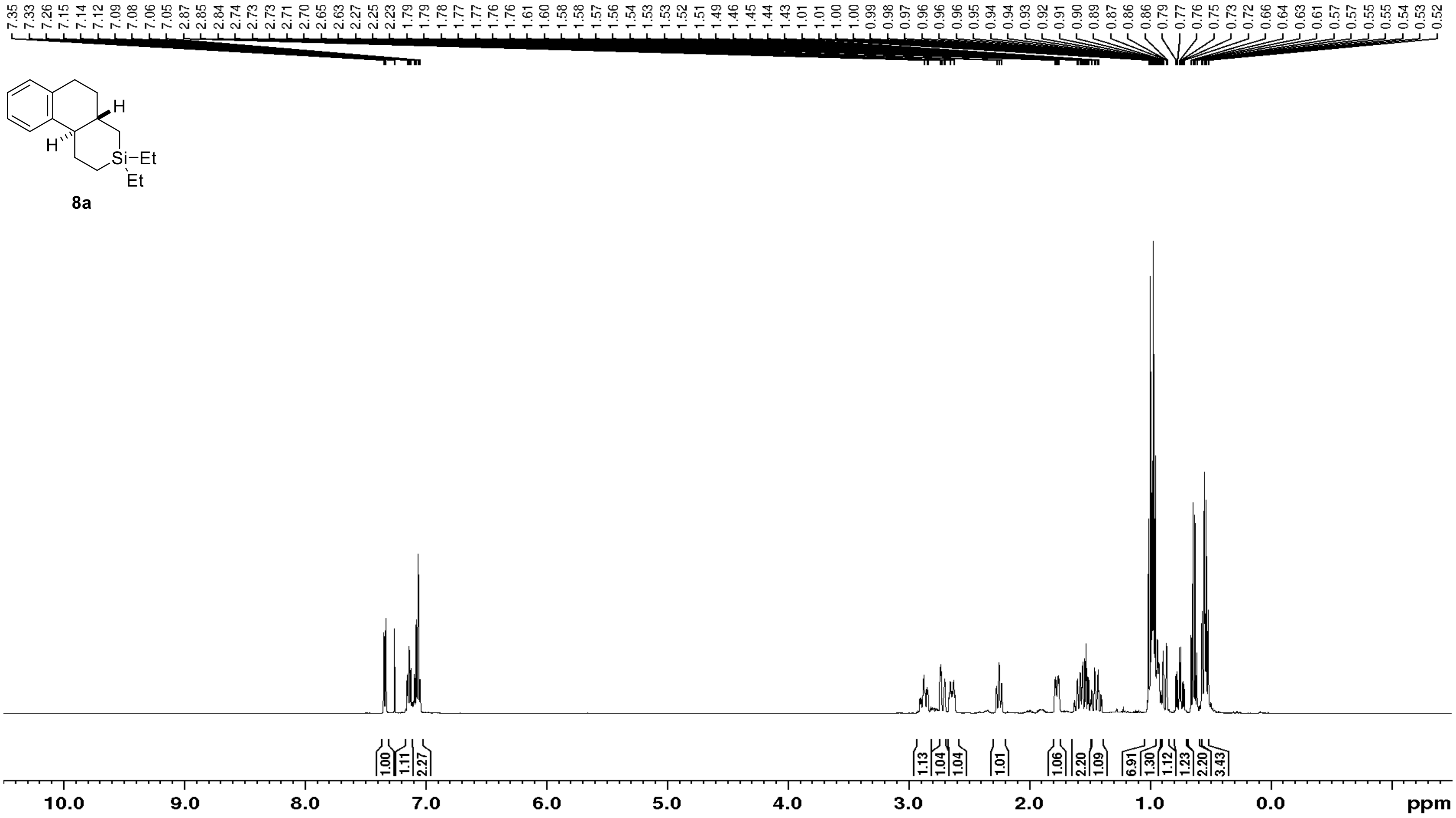

Figure S58. **$^{13}C\{^{1}H\}$ NMR** spectrum (126 MHz, $CDCl_3$, 298 K) of **8a** from the reaction of VCP **7** and Et_2SiH_2 (**2a**) using $Ph_3C^+[B(C_6F_5)_4]^-$ as initiator.

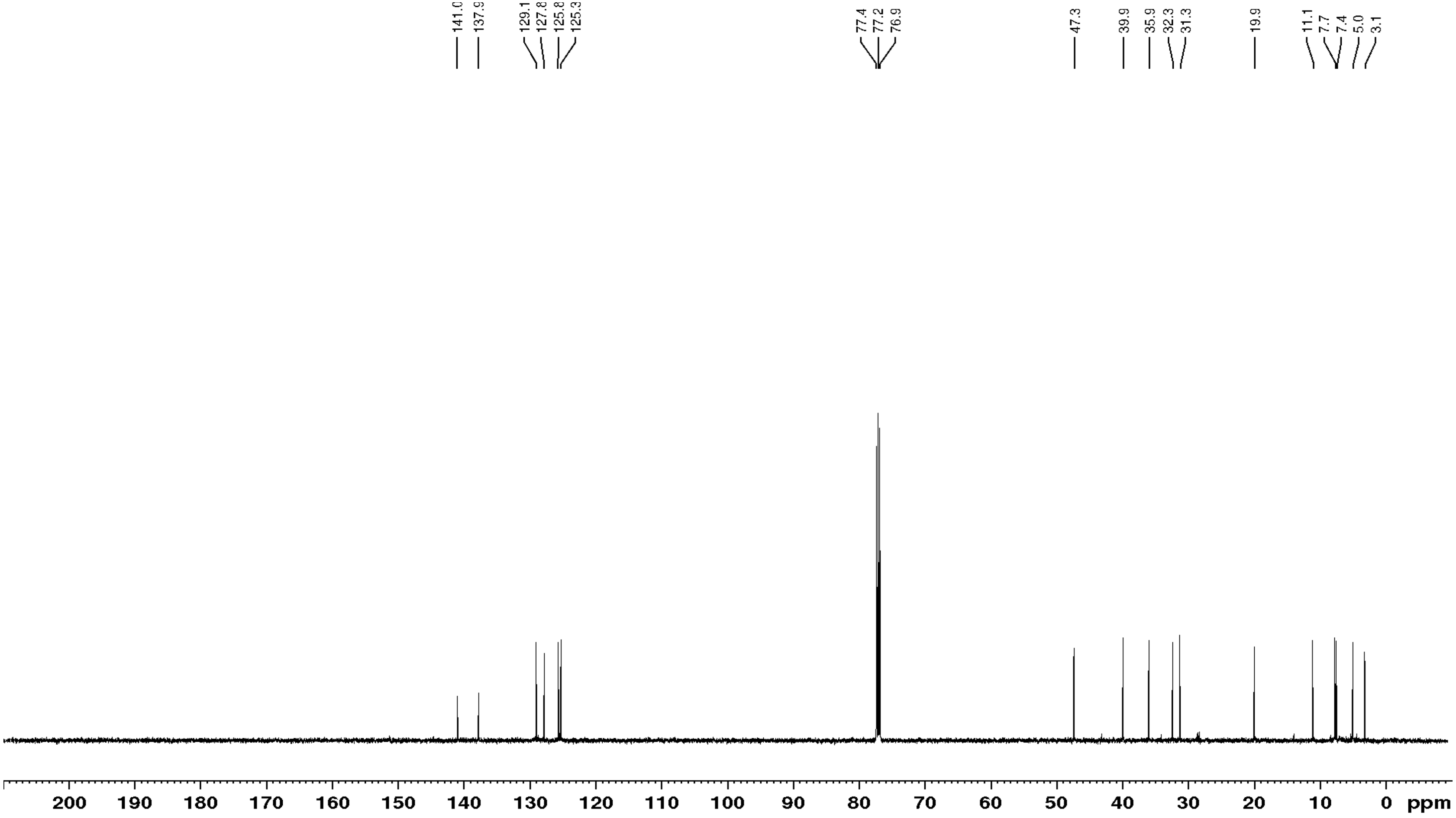

Figure S59. **^{1}H/^{29}Si HMQC NMR** spectrum (500/99 MHz, $CDCl_3$, 298 K, optimized for *J* = 7 Hz) of **8a** from the reaction of VCP **7** and Et_2SiH_2 (**2a**) using $Ph_3C^+[B(C_6F_5)_4]^-$ as initiator.

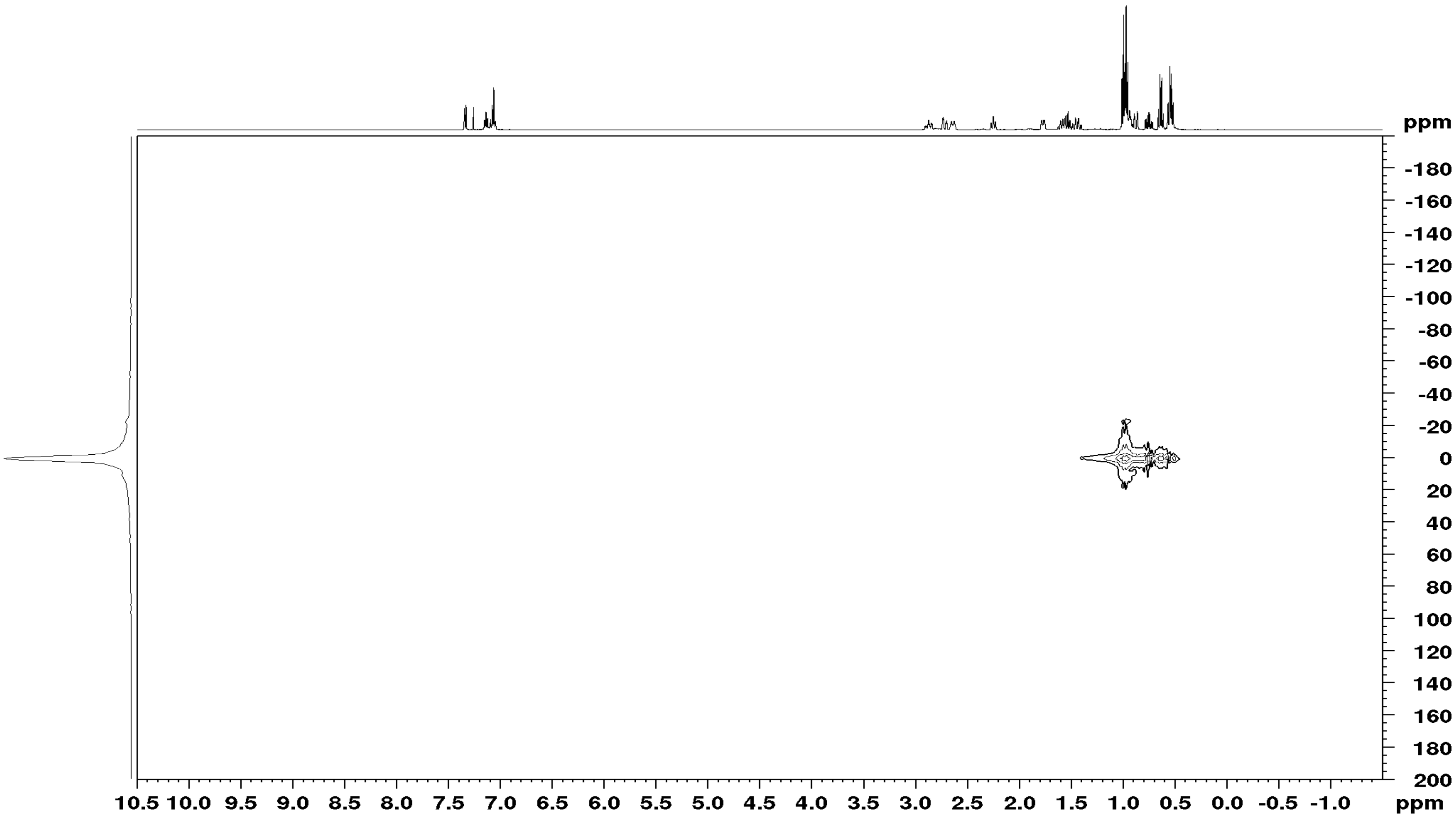

Figure S60. ^{1}**H NMR** spectrum (500 MHz, $CDCl_3$, 298 K) of **5aa** from the reaction of **9ab** and Et_3SiH (**2b**) using $Ph_3C^+[B(C_6F_5)_4]^-$ as initiator.

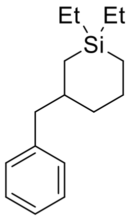

5aa

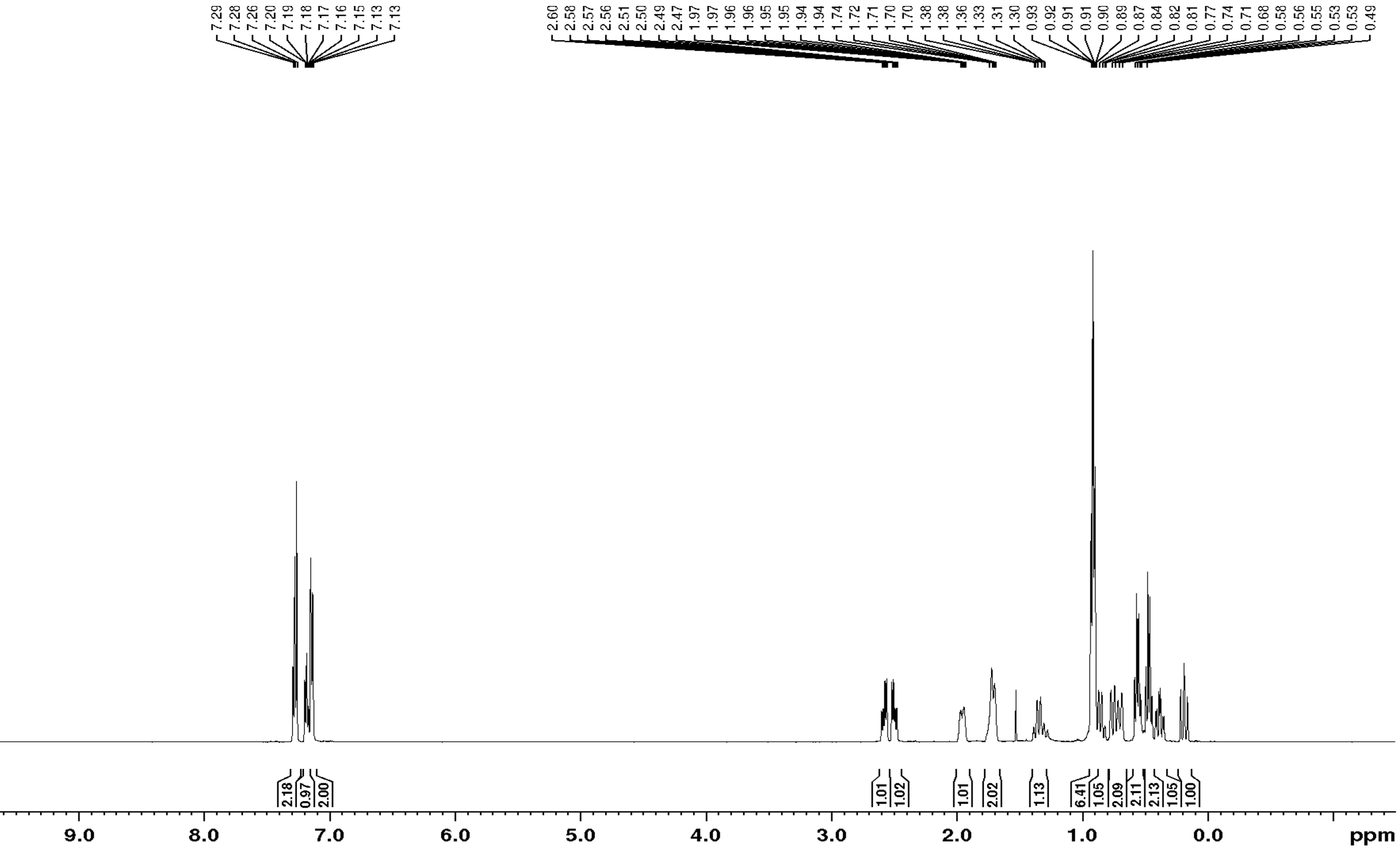

Figure S61. **$^{13}C\{^1H\}$ NMR** spectrum (126 MHz, $CDCl_3$, 298 K) of **5aa** from the reaction of **9ab** and Et_3SiH (**2b**) using $Ph_3C^+[B(C_6F_5)_4]^-$ as initiator.

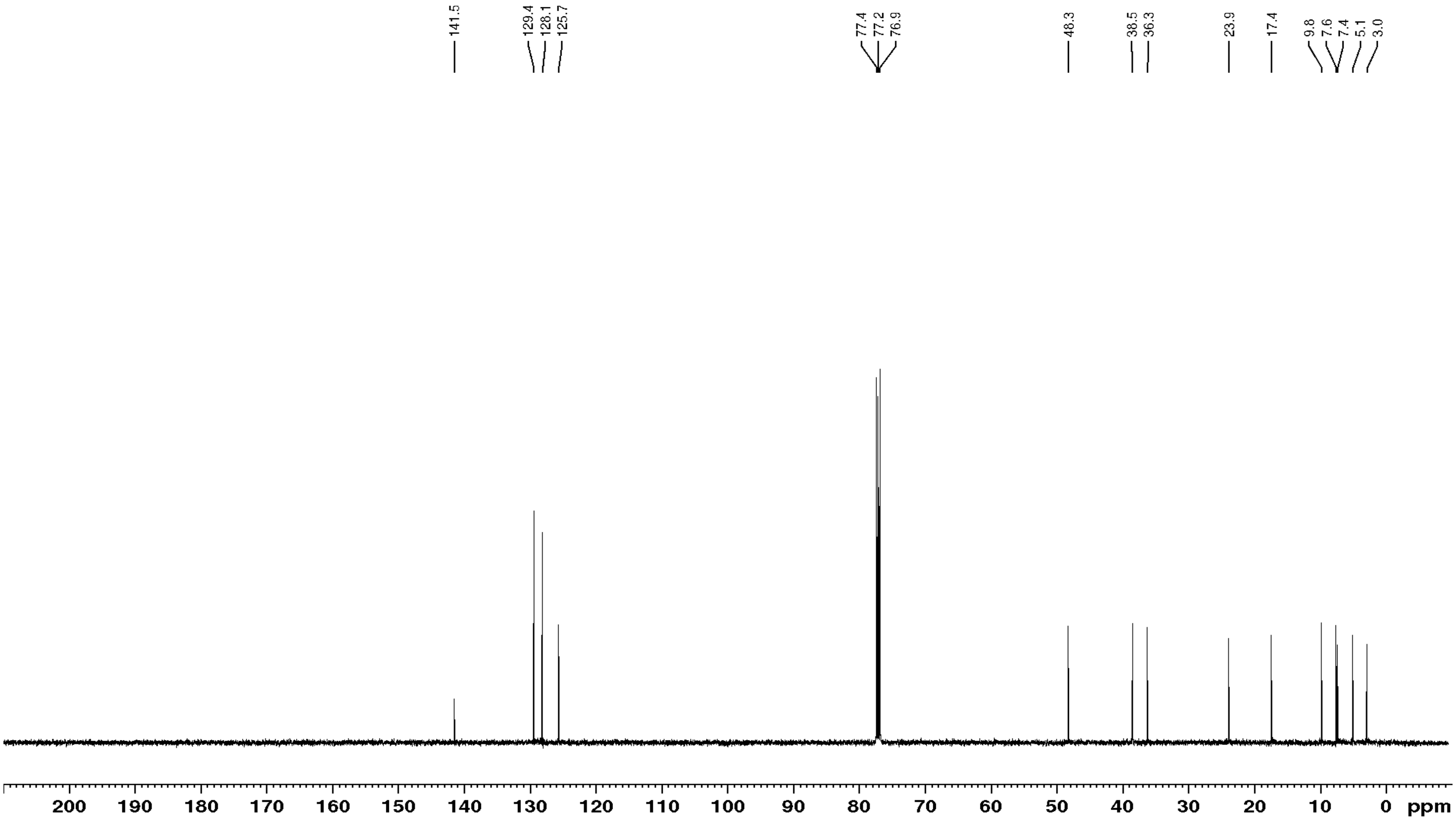

Figure S62. **$^{1}H/^{29}Si$ HMQC NMR** spectrum (500/99 MHz, $CDCl_3$, 298 K, optimized for J = 7 Hz) of **5aa** from the reaction of **9ab** and Et_3SiH (**2b**) using $Ph_3C^+[B(C_6F_5)_4]^-$ as initiator.

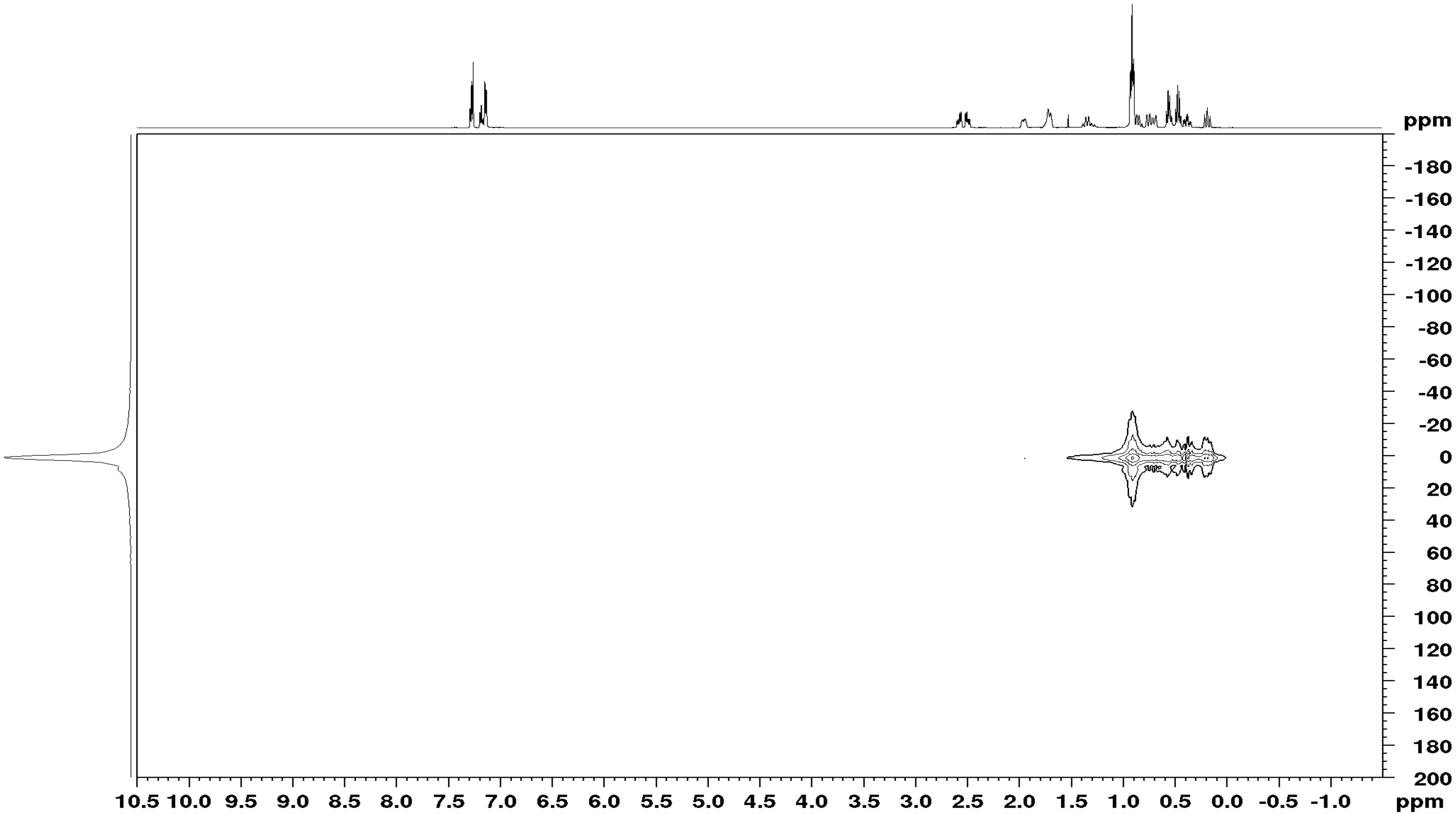

Figure S63. ^{1}**H NMR** spectrum (500 MHz, CD_2Cl_2, 298 K) of **3aa**-d_1 from the reaction of VCP **1a** and Et_3SiD (**2b**-d_1) using $Ph_3C^+[B(C_6F_5)_4]^-$ as initiator.

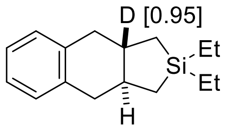

3aa-d_1

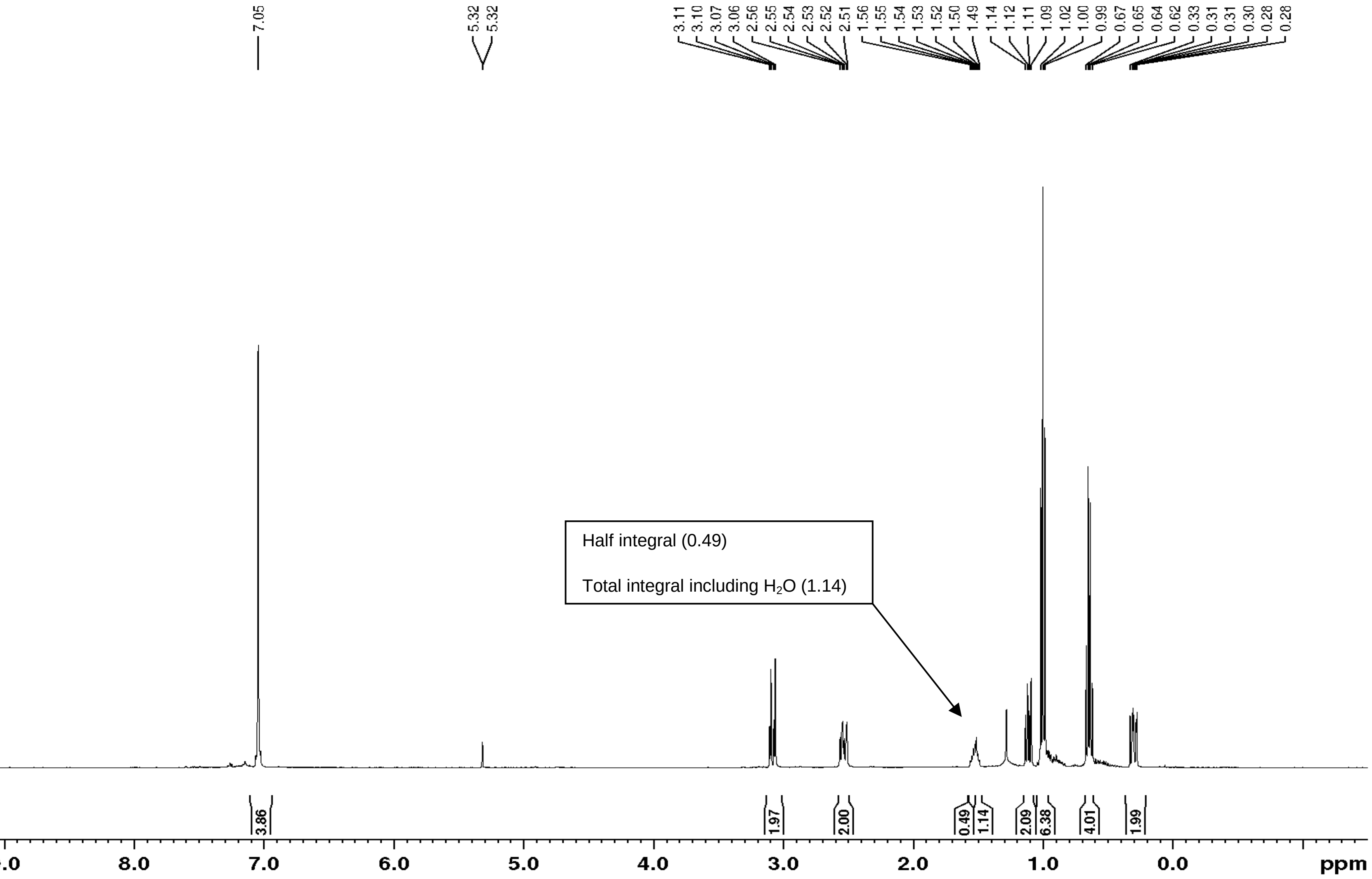

Figure S64. ^{2}**H NMR** spectrum (77 MHz, CD_2Cl_2/CH_2Cl_2, 298 K) of **3aa**-d_1 from the reaction of VCP **1a** and Et_3SiD (**2b**-d_1) using $Ph_3C^+[B(C_6F_5)_4]^-$ as initiator.

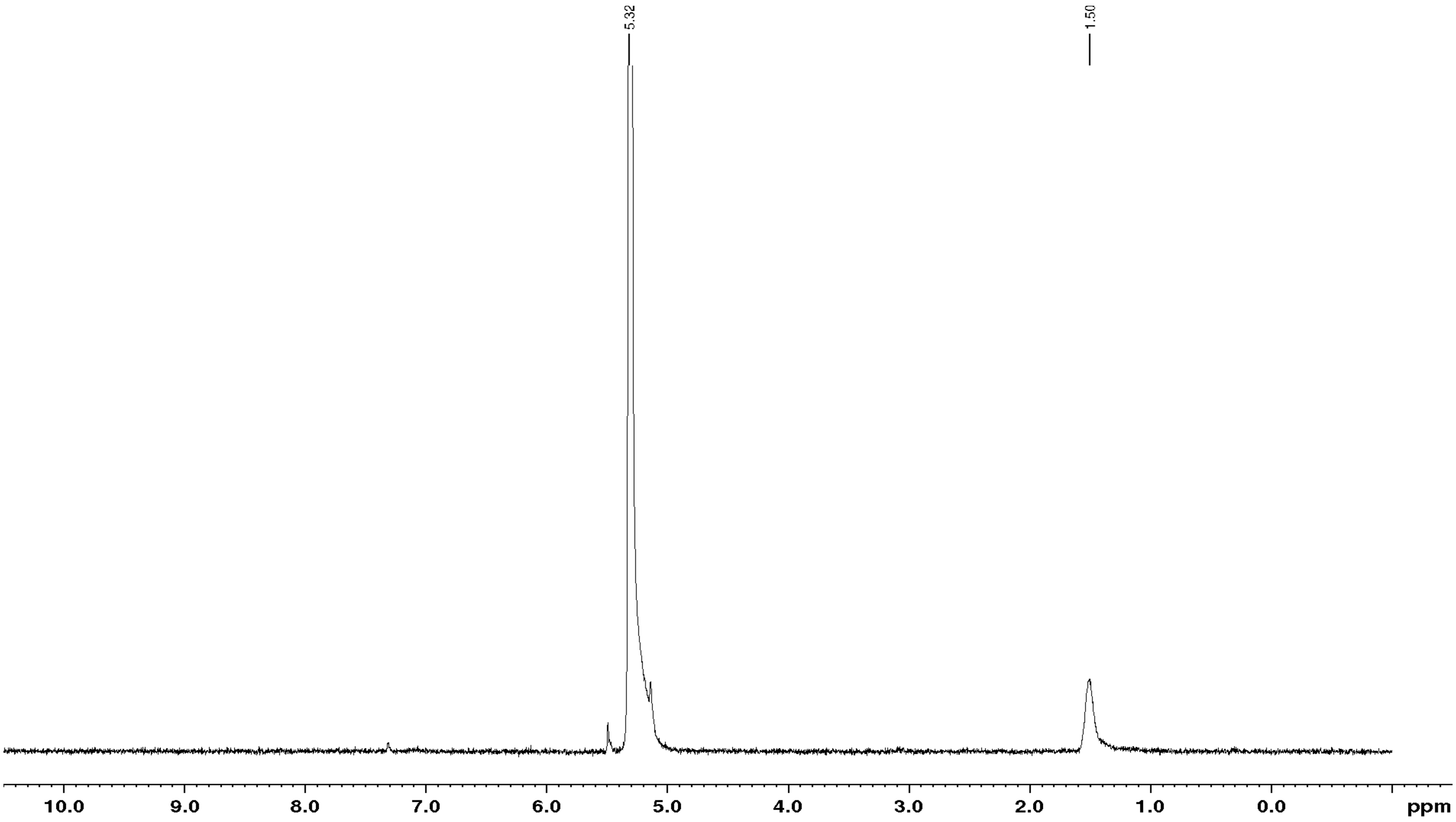

Figure S65. **^{1}H NMR** spectrum (500 MHz, CD_2Cl_2, 298 K) of **3aa**-*d* from the reaction of VCP **1a**-d_1 and Et_2SiH_2 (**2a**) using $Ph_3C^+[B(C_6F_5)_4]^-$ as initiator.

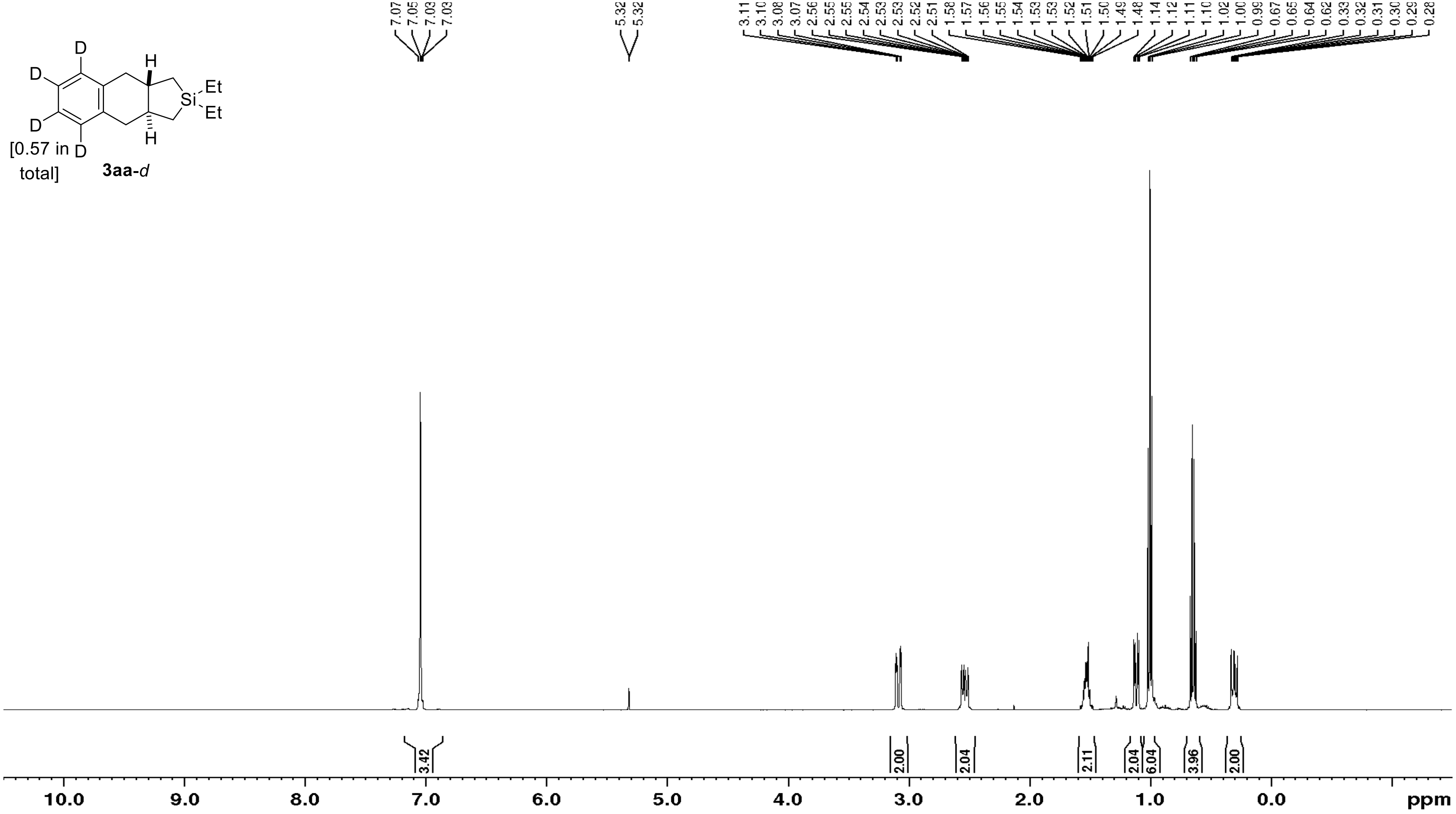

Figure S66. **^{2}H NMR** spectrum (77 MHz, CD_2Cl_2, 298 K) of **3aa**-*d* from the reaction of VCP **1a**-d_1 and Et_2SiH_2 (**2a**) using $Ph_3C^+[B(C_6F_5)_4]^-$ as initiator.

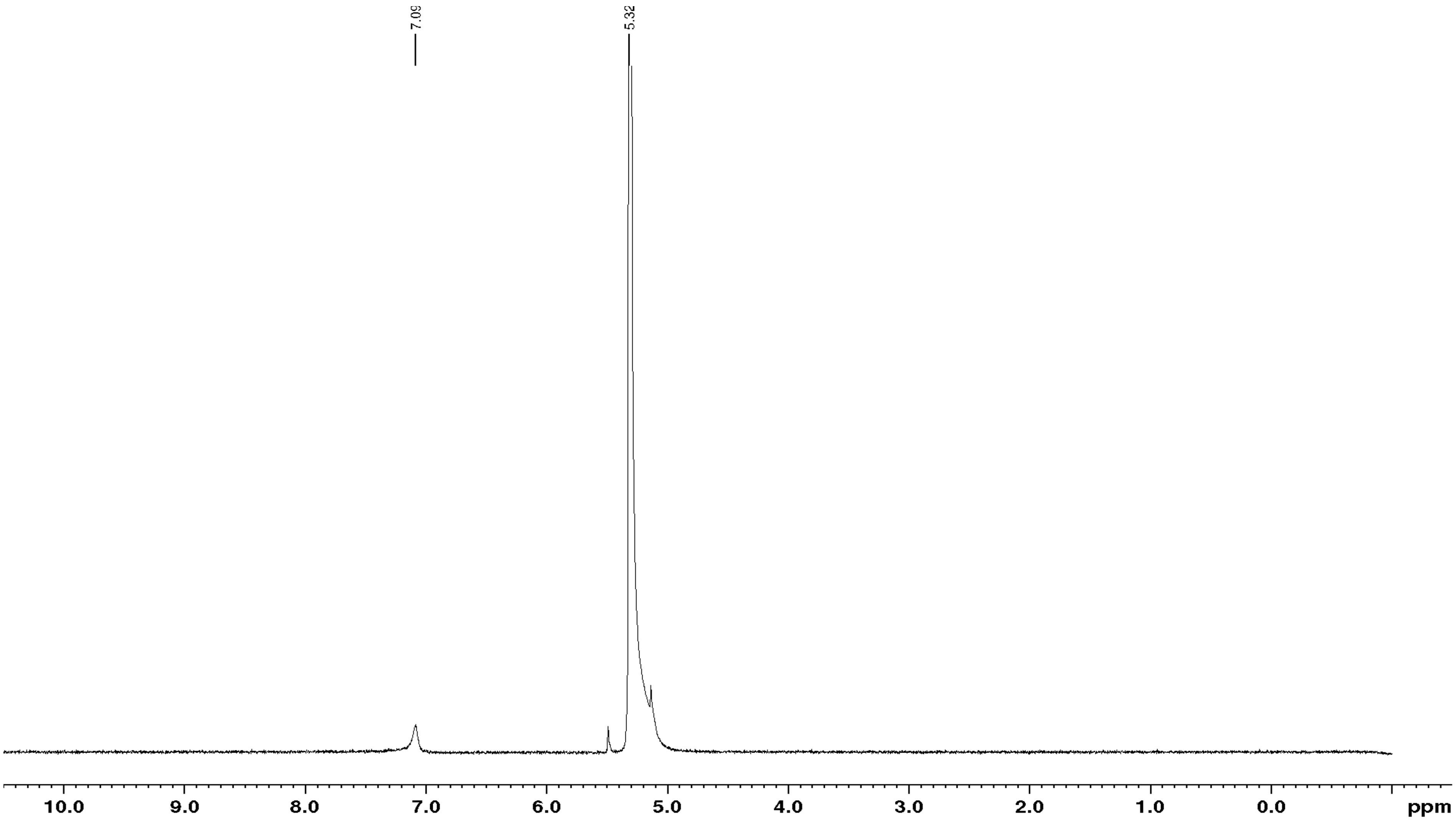

Figure S67. **$^{13}C\{^1H\}$ NMR** spectrum (126 MHz, $CDCl_3$, 298 K) of **3aa**-*d* from the reaction of VCP **1a**-d_1 and Et_2SiH_2 (**2a**) using $Ph_3C^+[B(C_6F_5)_4]^-$ as initiator (* partly deuterated carbon).

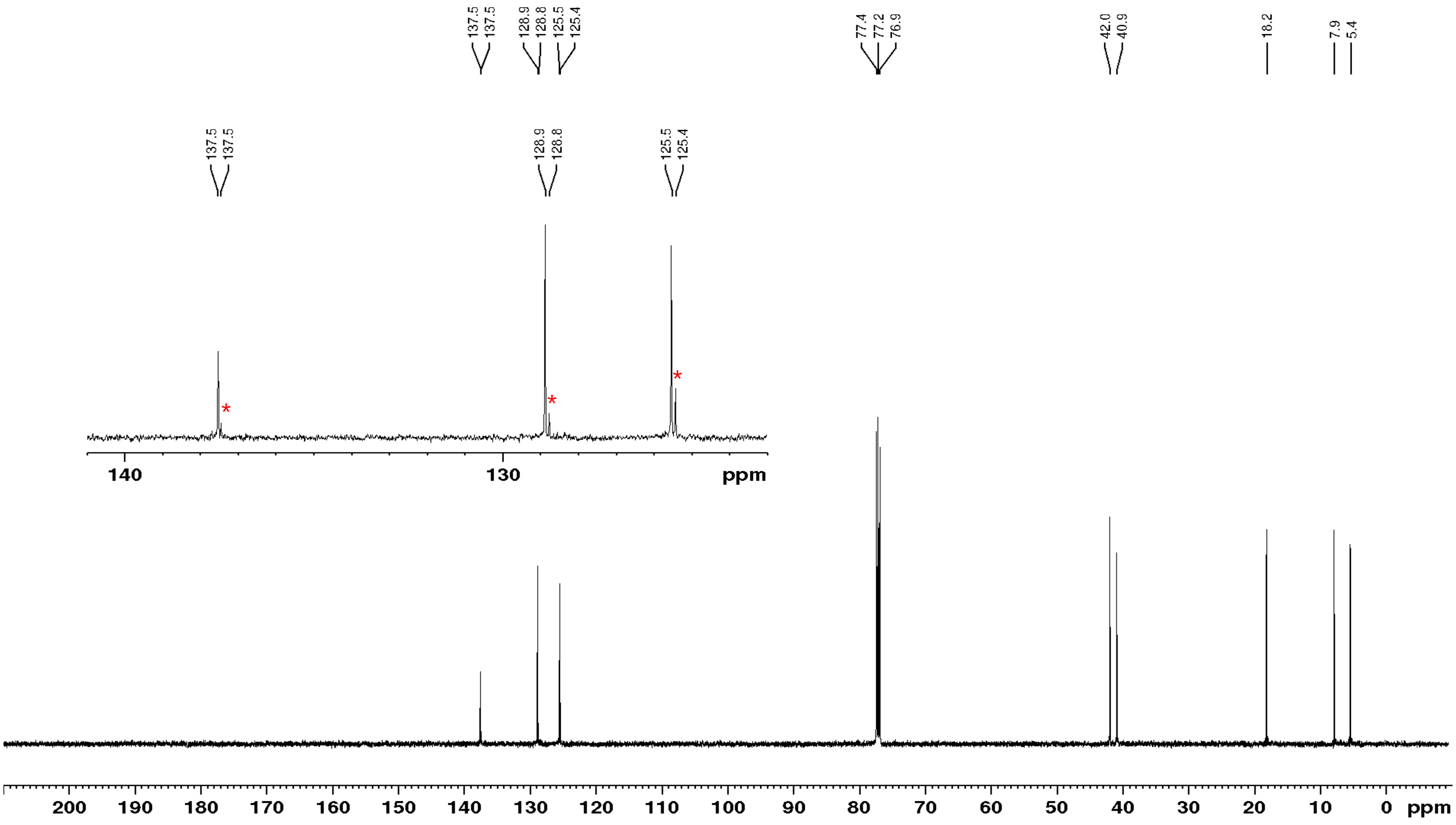

Figure S68. **^{1}H NMR** spectrum (500 MHz, $CDCl_3$, 298 K) of independently prepared **4aa** after treatment with catalytic amounts of $Ph_3C^+[B(C_6F_5)_4]^-$ (* for **4aa**, # for **4ab** and ~ for **4ac**).

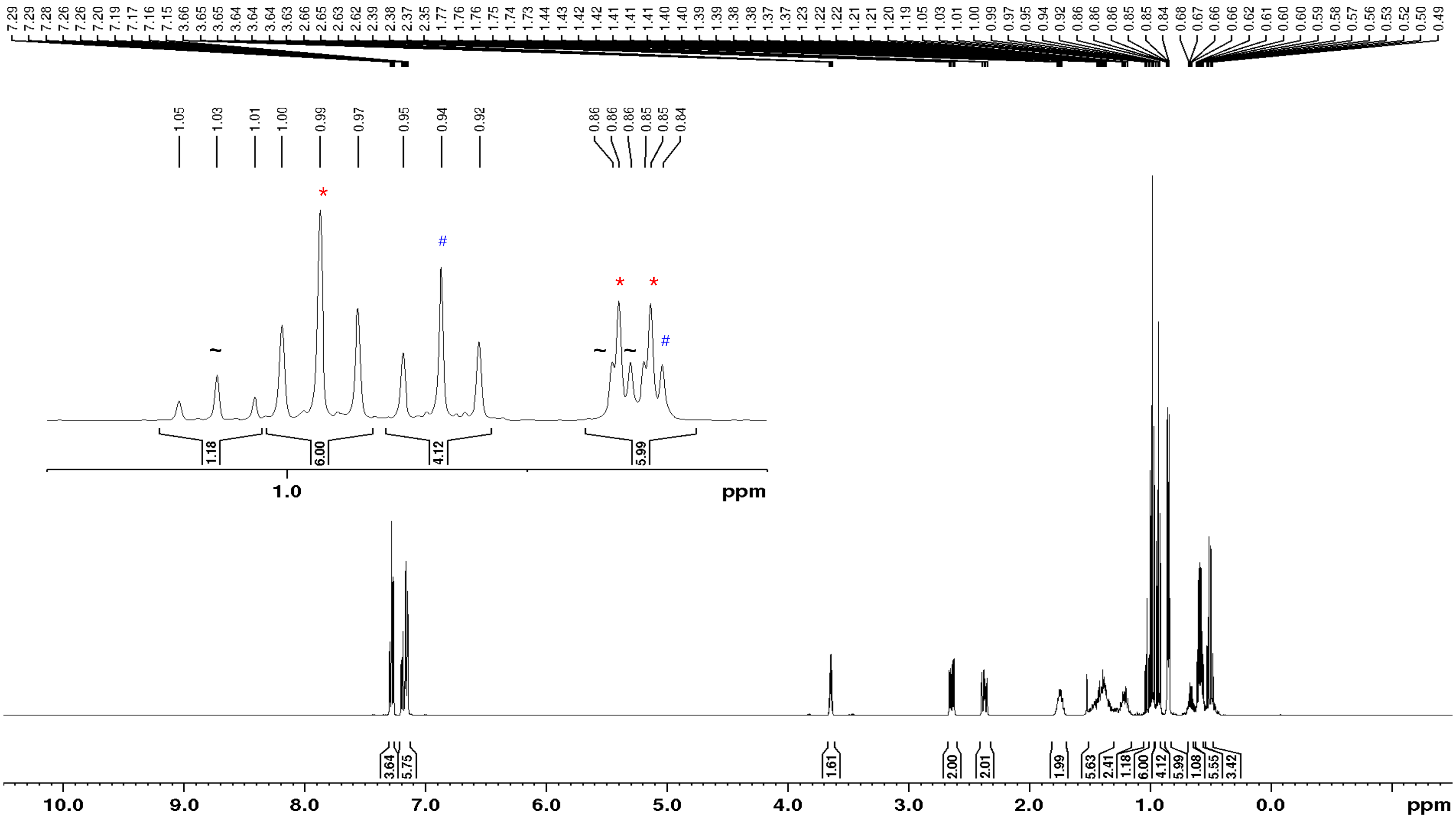

Figure S69. **$^{1}H/^{29}Si$ HMQC NMR** spectrum (500/99 MHz, $CDCl_3$, 298 K, optimized for J = 7 Hz) of independently prepared **4aa** after treatment with catalytic amounts of $Ph_3C^{+}[B(C_6F_5)_4]^{-}$ (* for **4aa**, # for **4ab** and ~ for **4ac**).

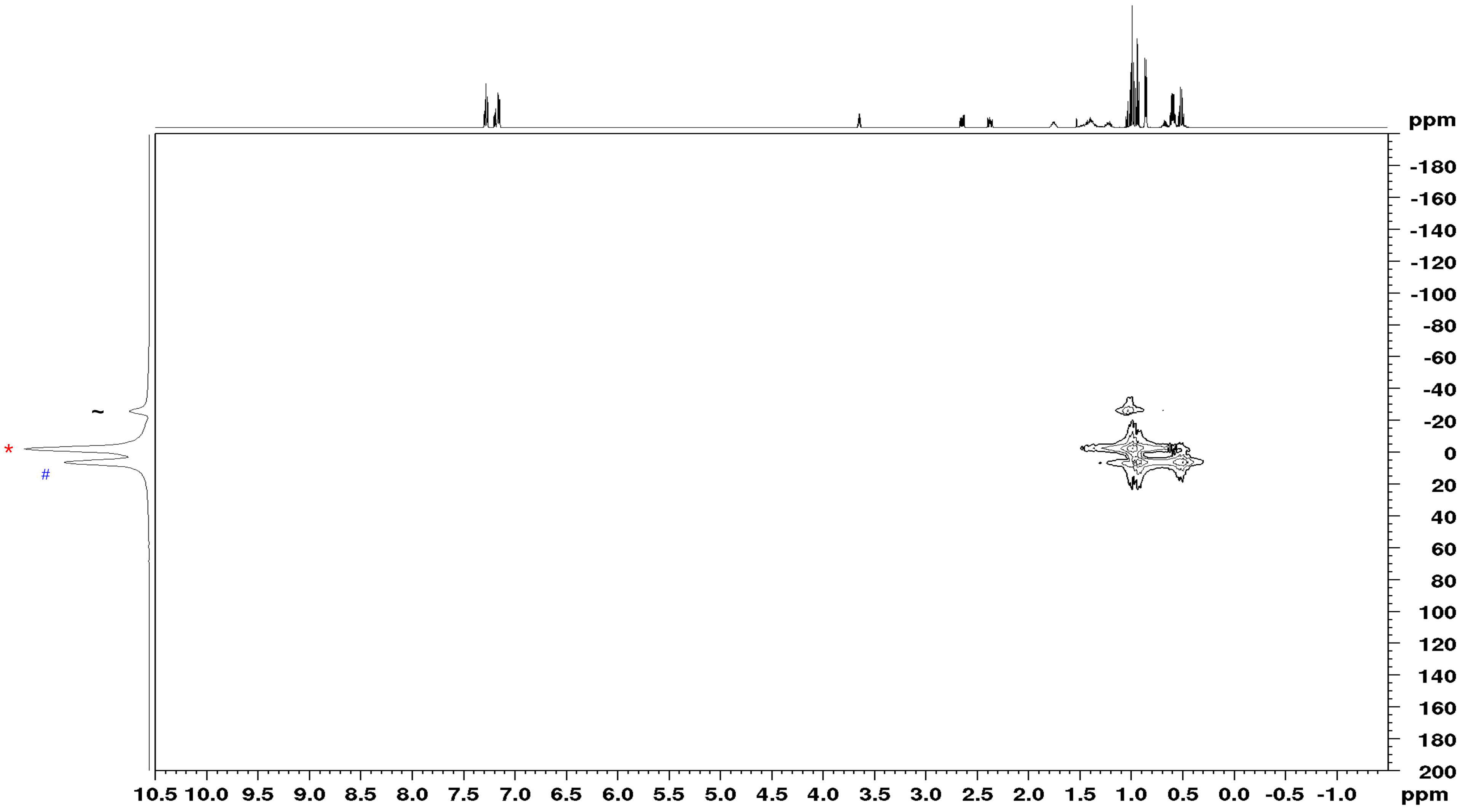

Figure S70. **$^{1}H/^{29}Si$ HMQC NMR** spectrum (500/99 MHz, $CDCl_3$, 298 K, optimized for *J* = 200 Hz) of independently prepared **4aa** after treatment with catalytic amounts of $Ph_3C^+[B(C_6F_5)_4]^-$ (* for **4aa** and **~** for **4ac**).

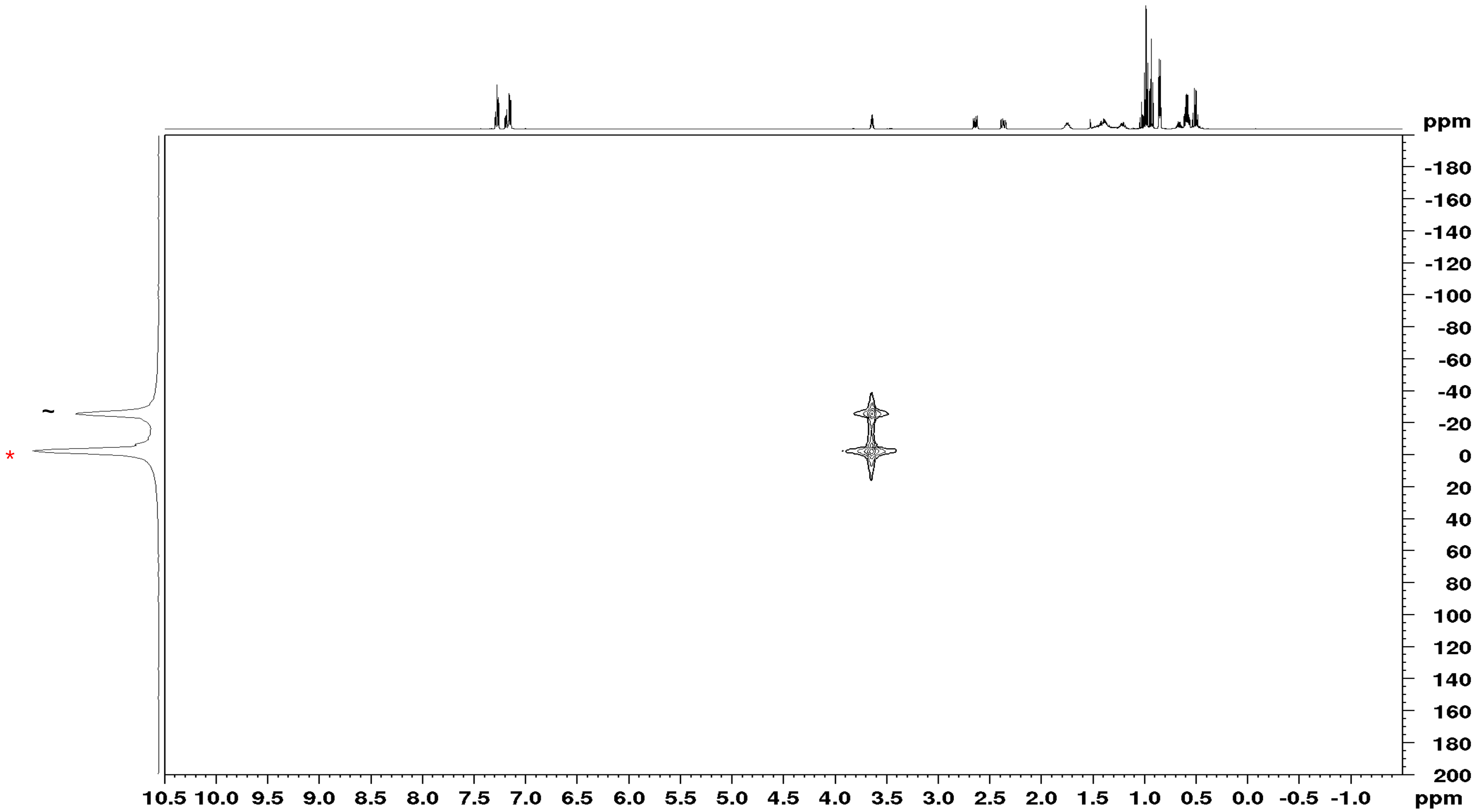

Figure S71. **^{1}H NMR** spectrum (500 MHz, $CDCl_3$, 298 K) of VCP **7**.

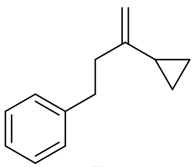

7

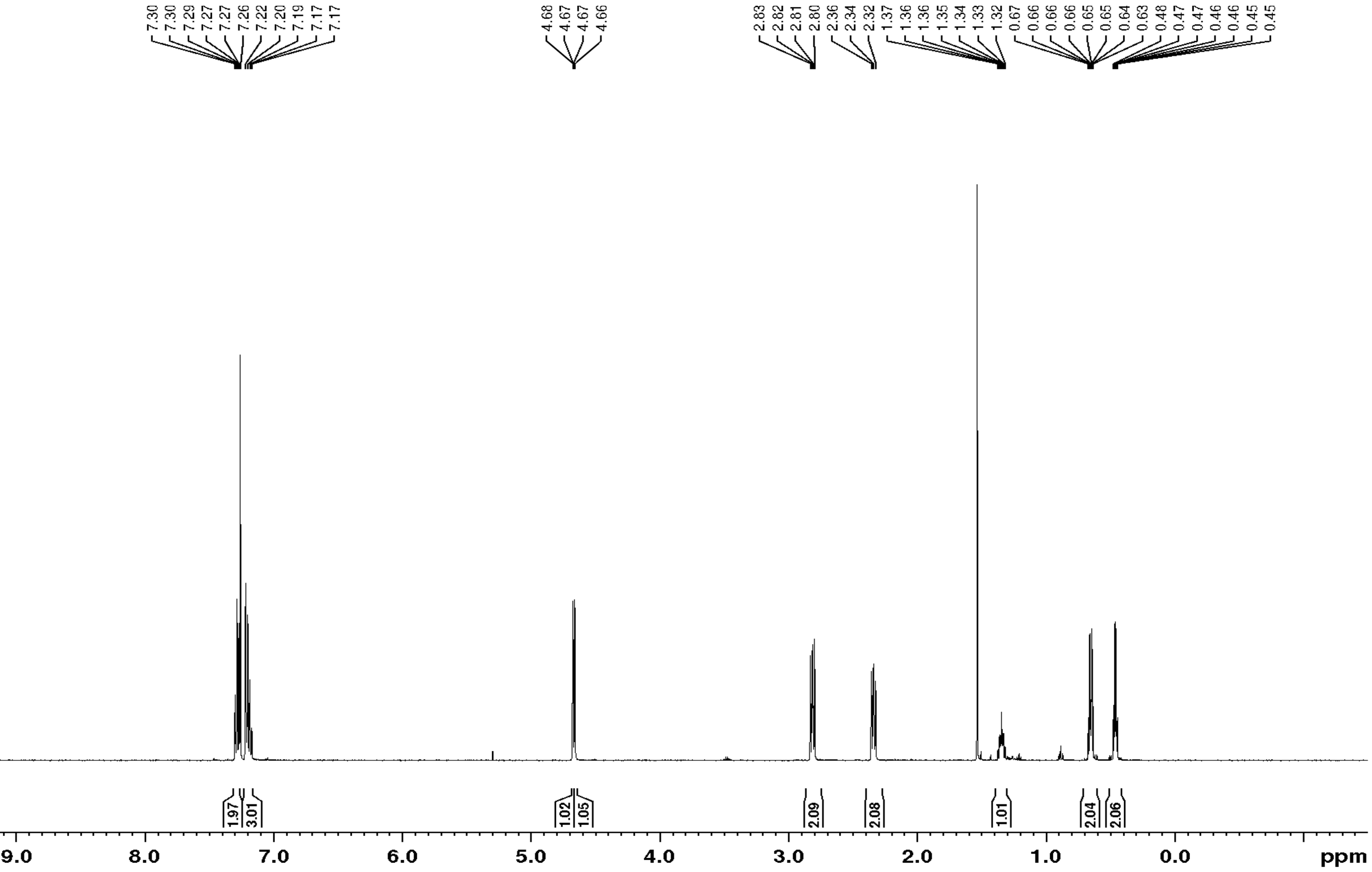

Figure S72. **$^{13}C\{^{1}H\}$ NMR** spectrum (126 MHz, $CDCl_3$, 298 K) of VCP **7**.

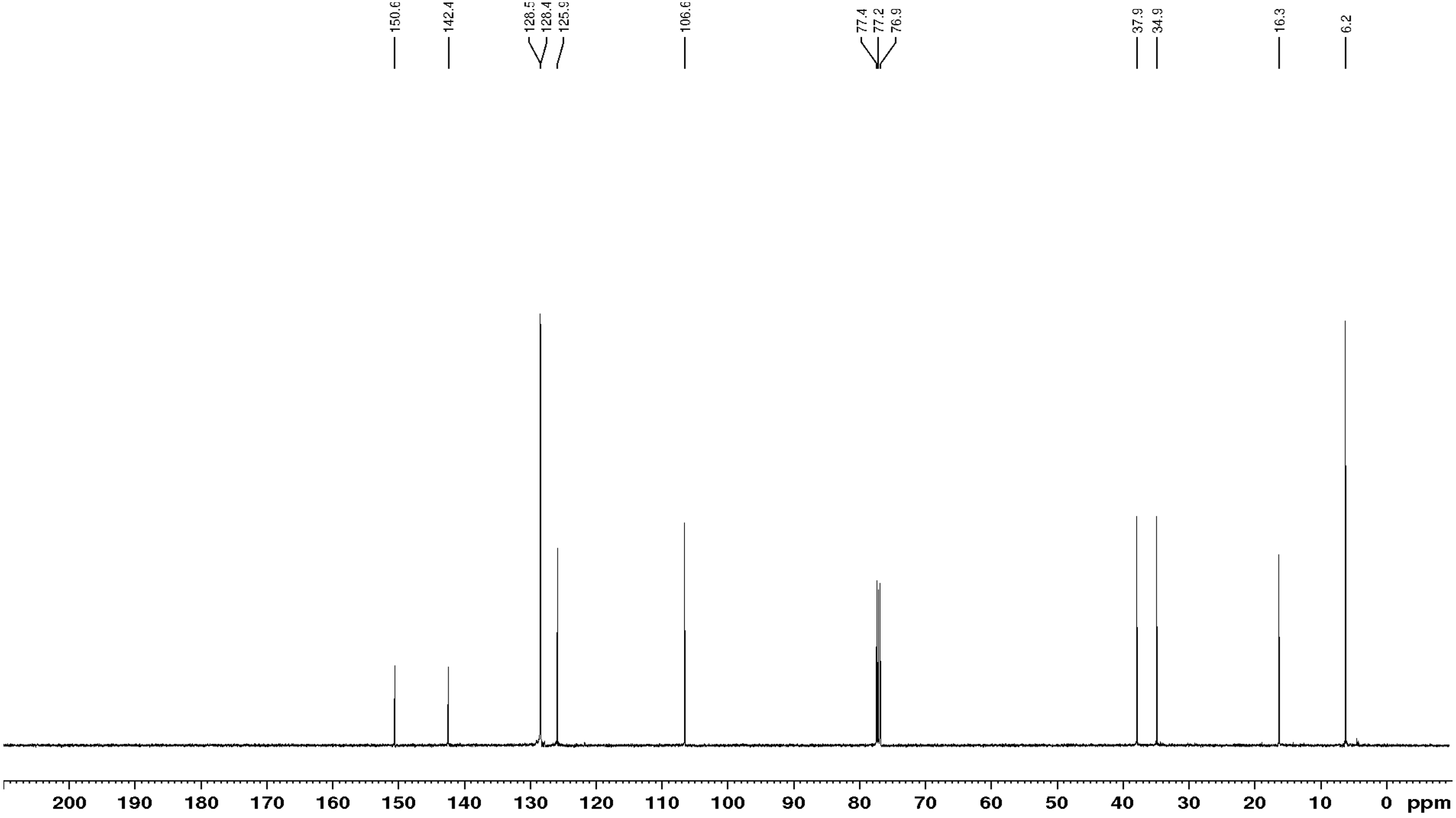

Figure S73. **^{1}H NMR** spectrum (500 MHz, $CDCl_3$, 298 K) of **9ab**.

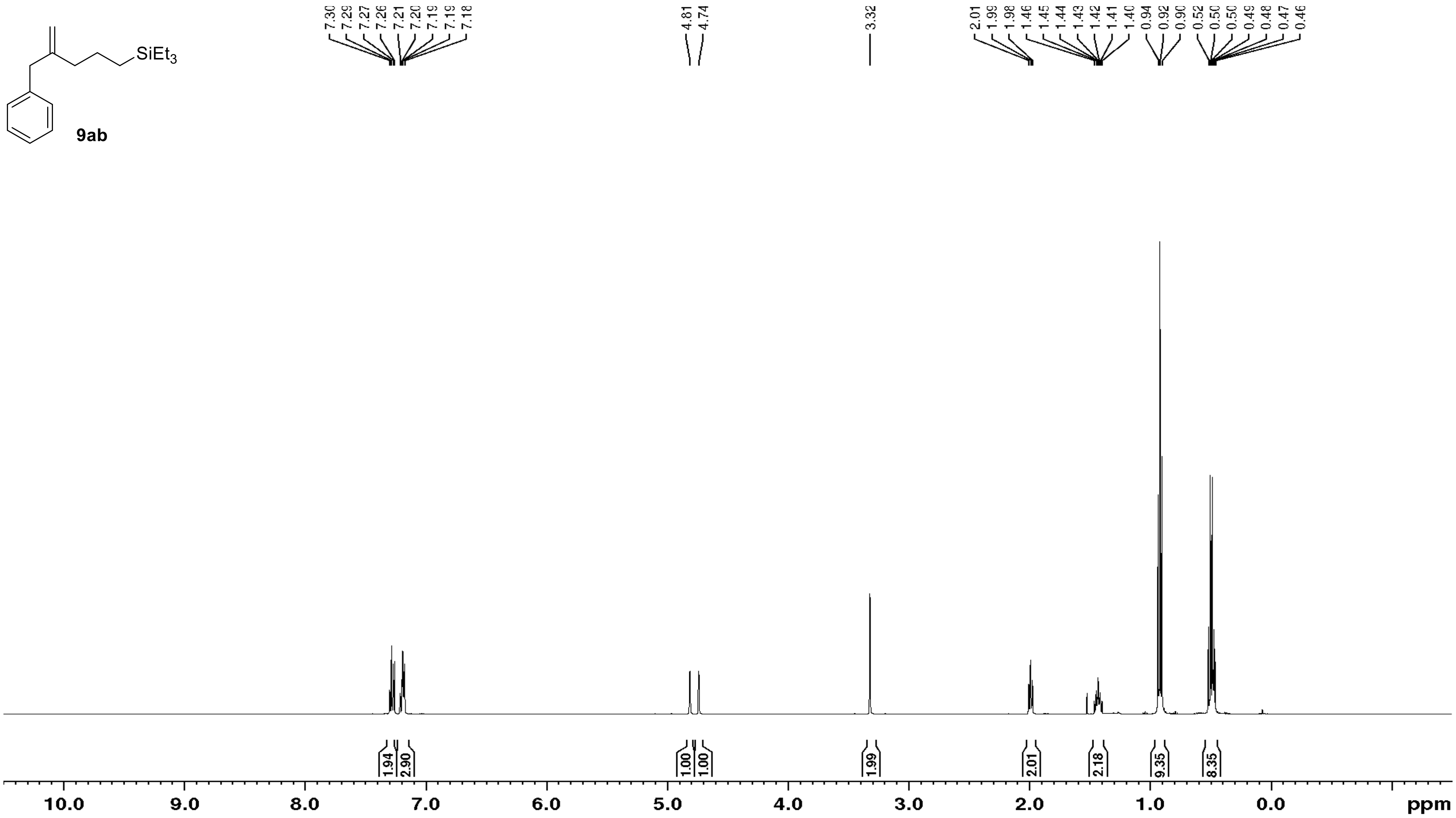

Figure S74. **$^{13}C\{^1H\}$ NMR** spectrum (126 MHz, $CDCl_3$, 298 K) of **9ab**.

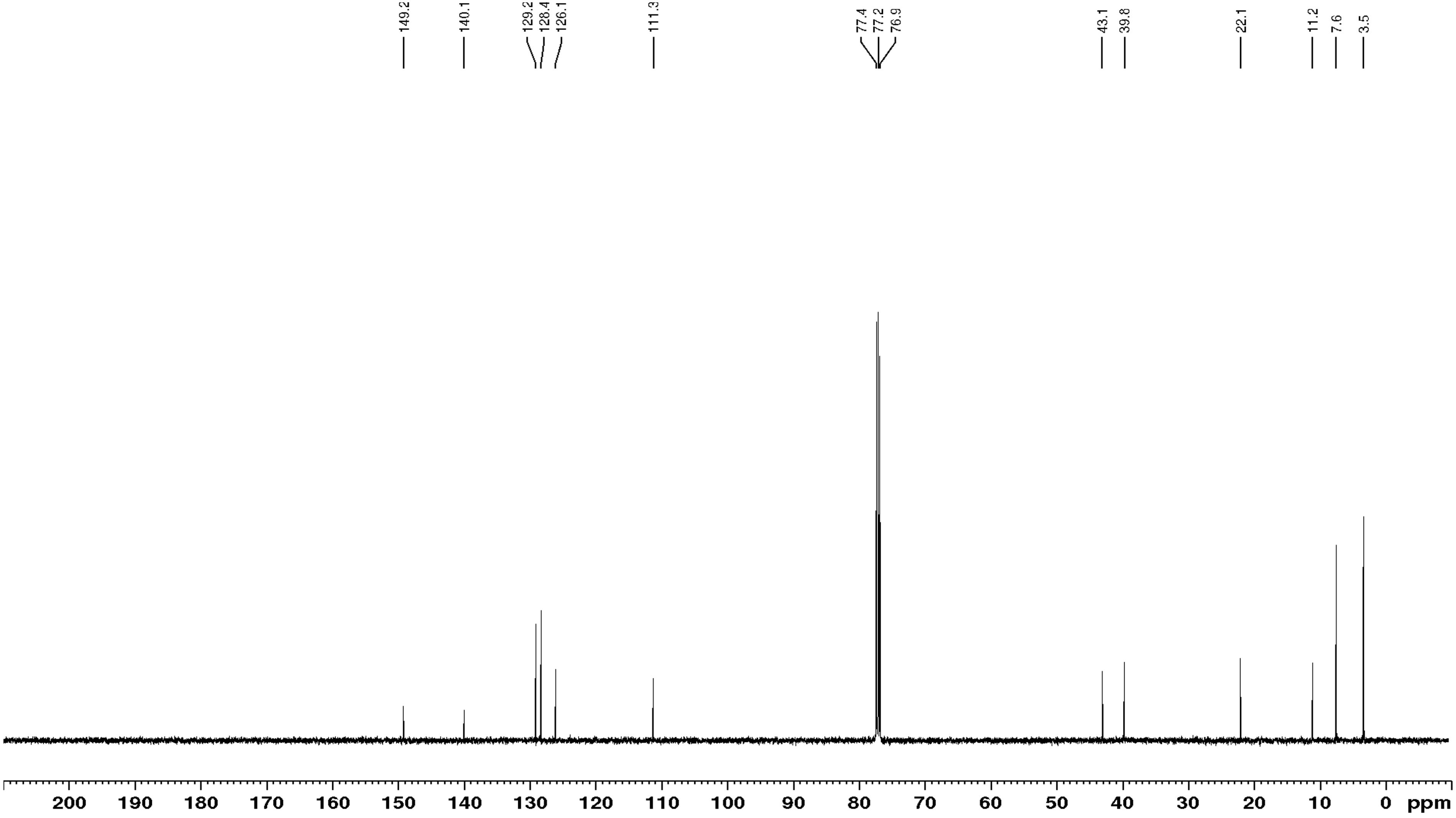

Figure S75. **$^{1}H/^{29}Si$ HMQC NMR** spectrum (500/99 MHz, $CDCl_3$, 298 K, optimized for J = 7 Hz) of **9ab**.

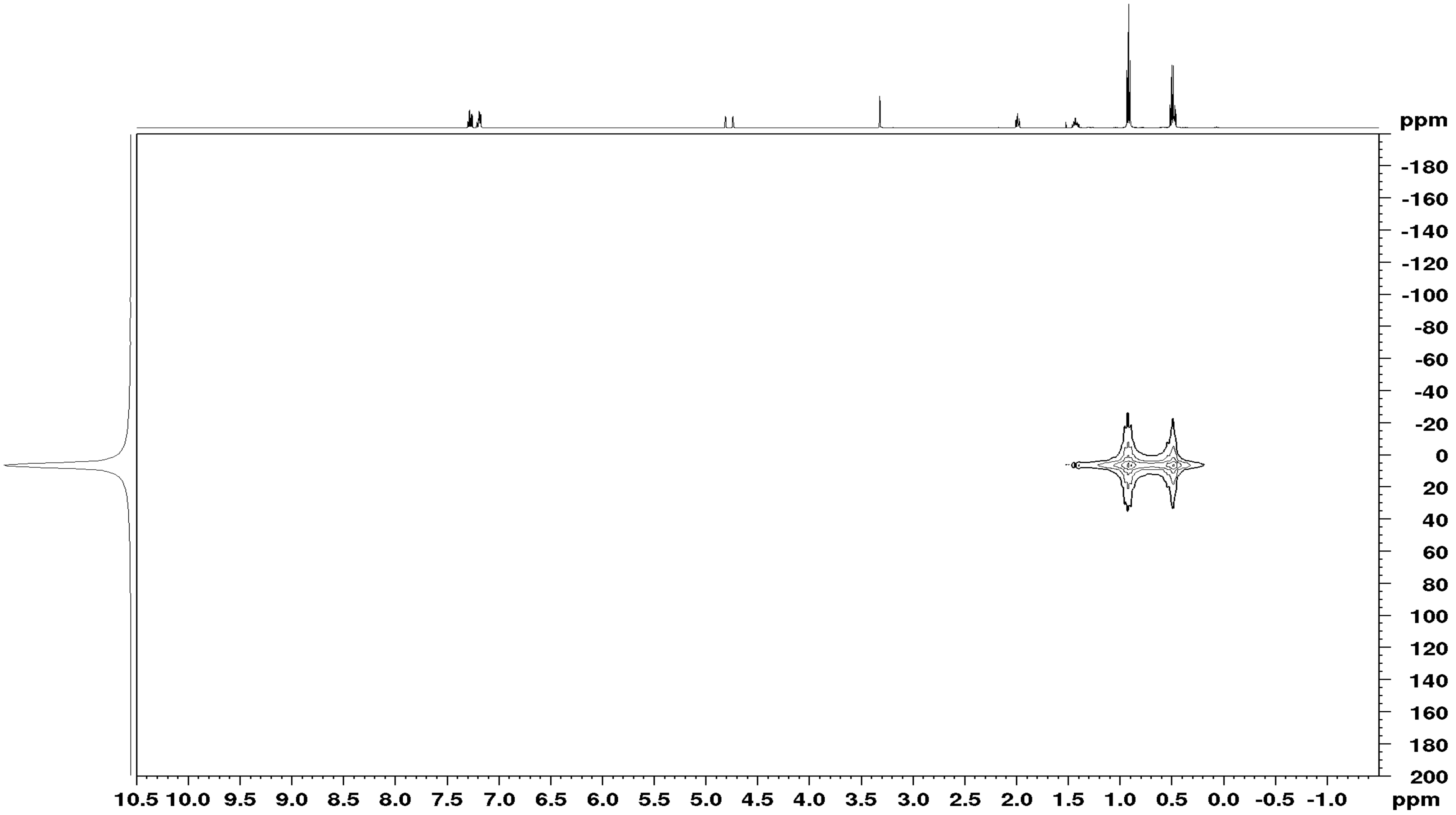

Figure S76. **^{1}H NMR** spectrum (500 MHz, $CDCl_3$, 298 K) of **4aa**.

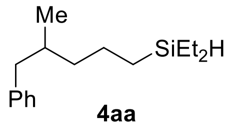

4aa

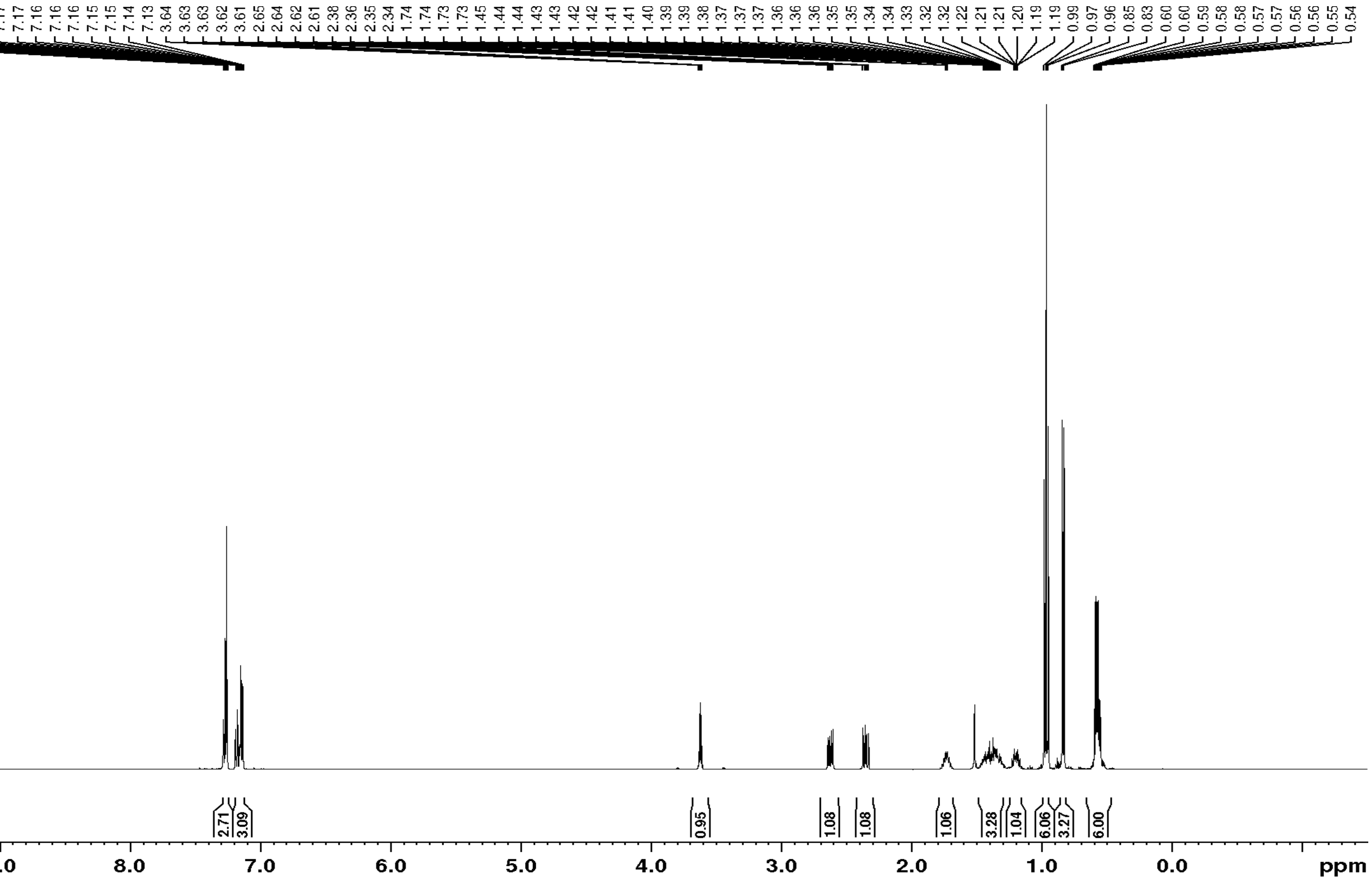

Figure S77. **$^{13}C\{^1H\}$ NMR** spectrum (126 MHz, $CDCl_3$, 298 K) of **4aa**.

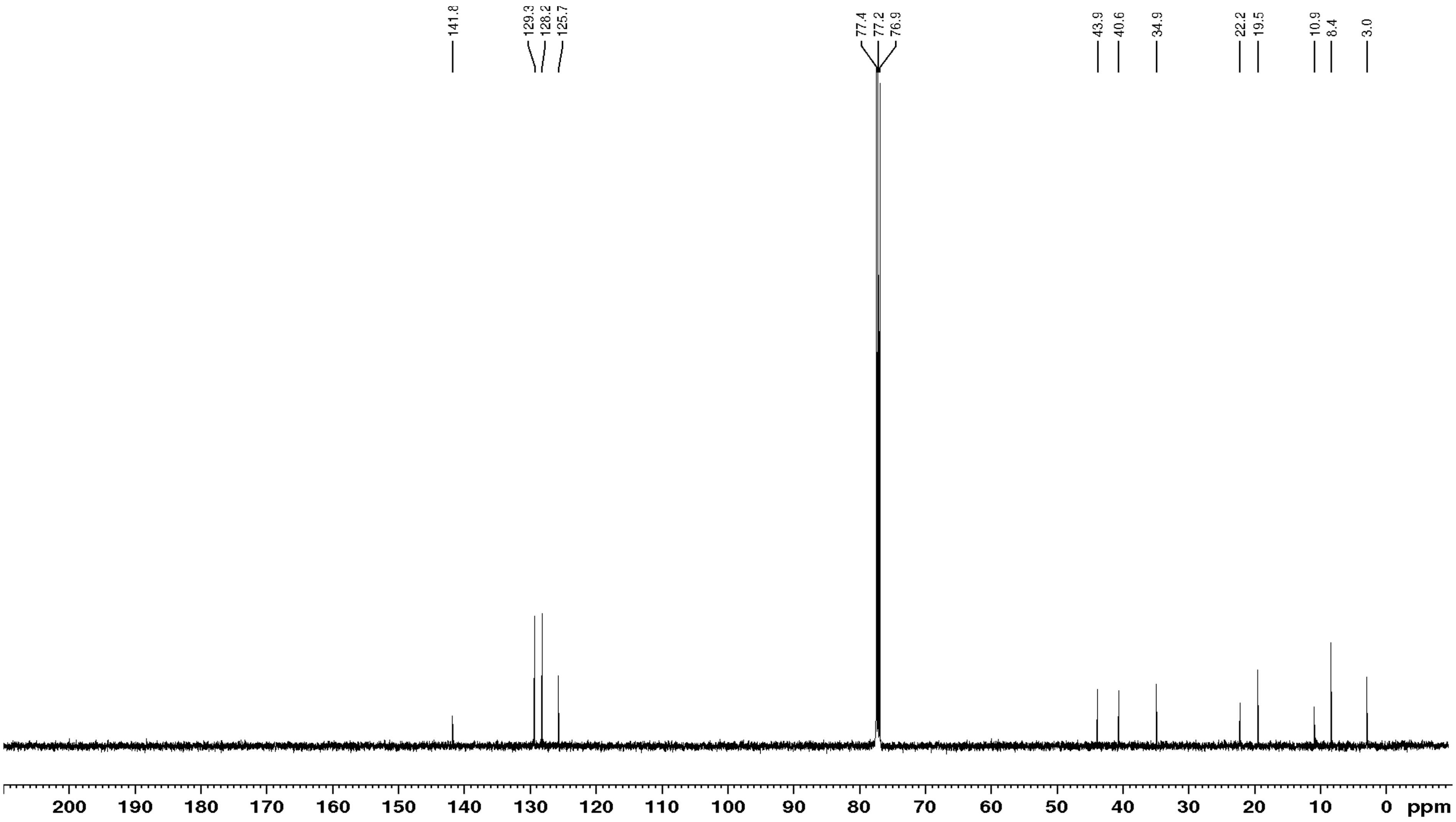

Figure S78. **^{1}H/^{29}Si HMQC NMR** spectrum (500/99 MHz, $CDCl_3$, 298 K, optimized for J = 7 Hz) of **4aa**.

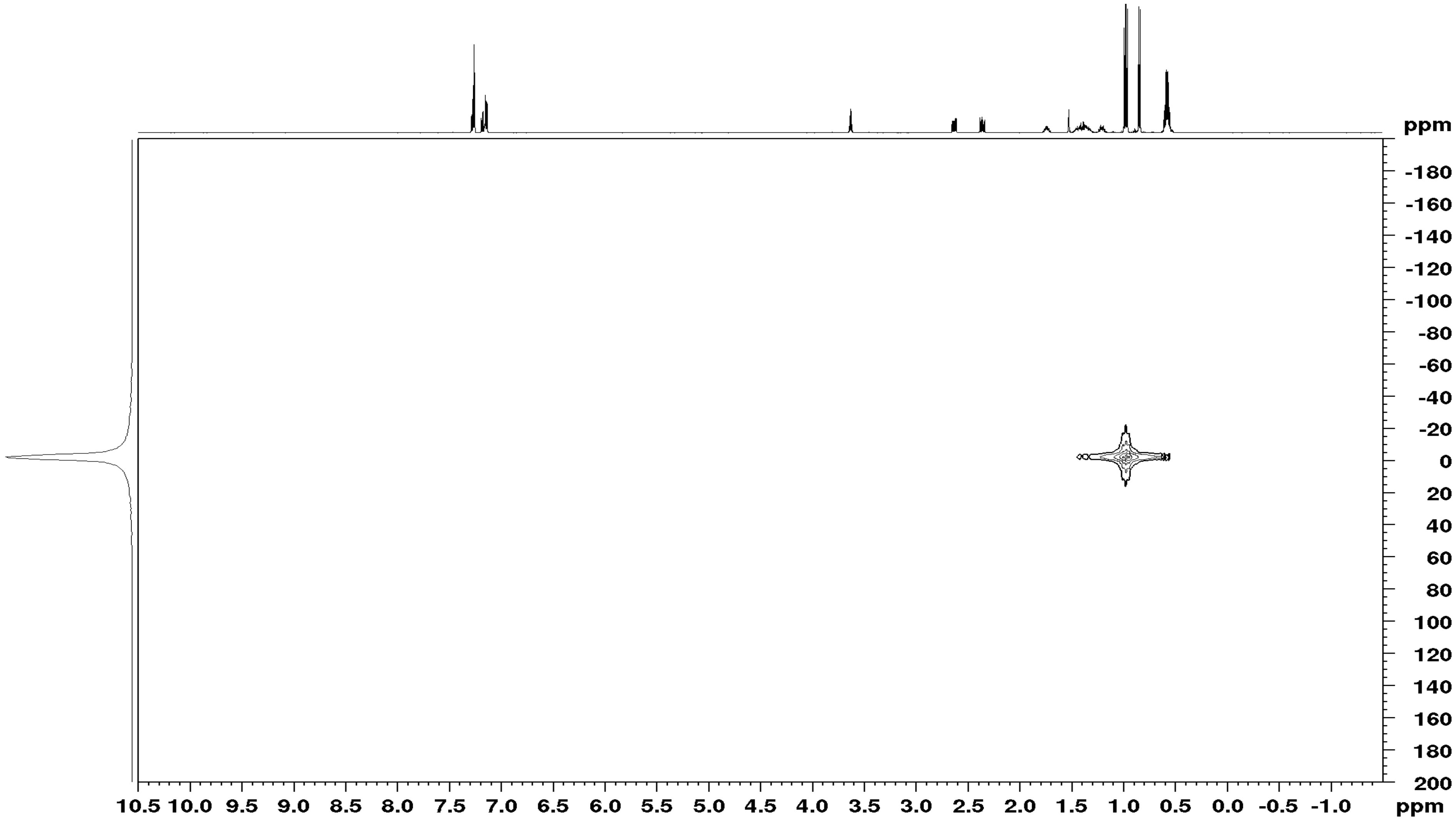

Figure S79. **$^1H/^{29}Si$ HMQC NMR** spectrum (500/99 MHz, $CDCl_3$, 298 K, optimized for J = 200 Hz) of **4aa**.

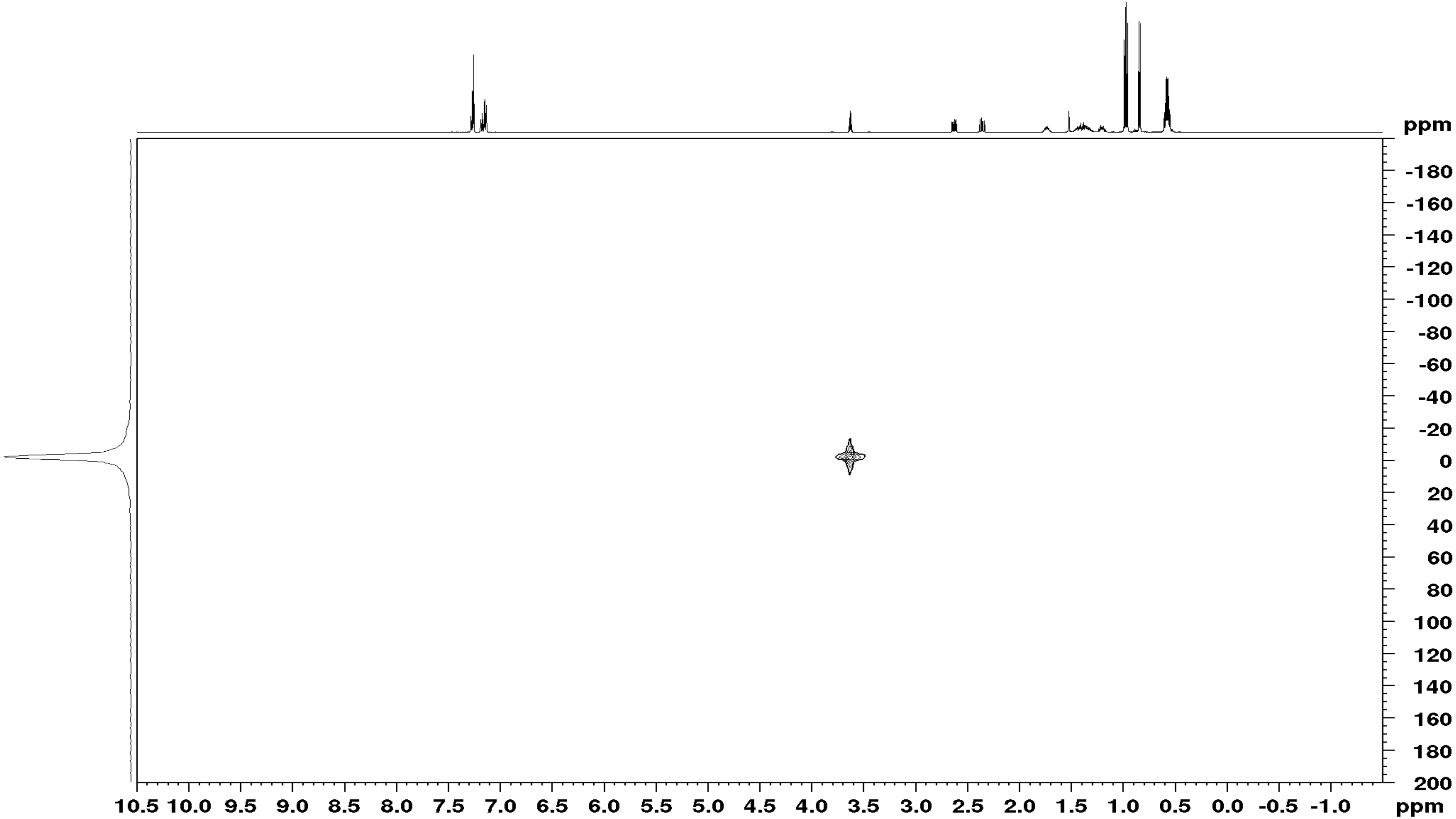

Figure S80. **^{1}H NMR** spectrum (500 MHz, $CDCl_3$, 298 K) of **4ab** from the reaction of VCP **1a** and Et_2SiH_2 (**2a**) using $Ph_3C^+[B(C_6F_5)_4]^-$ as initiator.

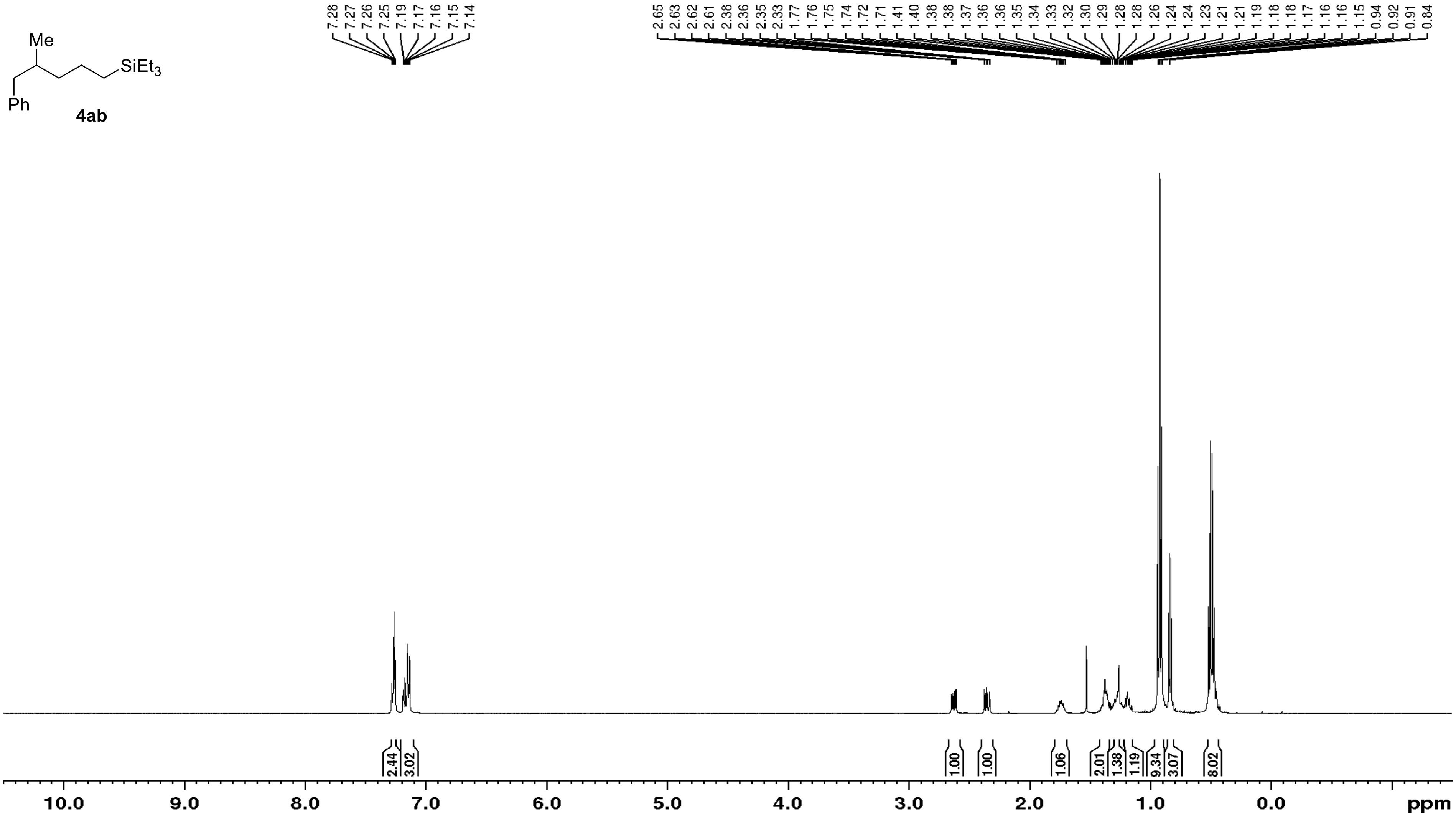

Figure S81. **$^{13}C\{^1H\}$ NMR** spectrum (126 MHz, $CDCl_3$, 298 K) of **4ab** from the reaction of VCP **1a** and Et_2SiH_2 (**2a**) using $Ph_3C^+[B(C_6F_5)_4]^-$ as initiator.

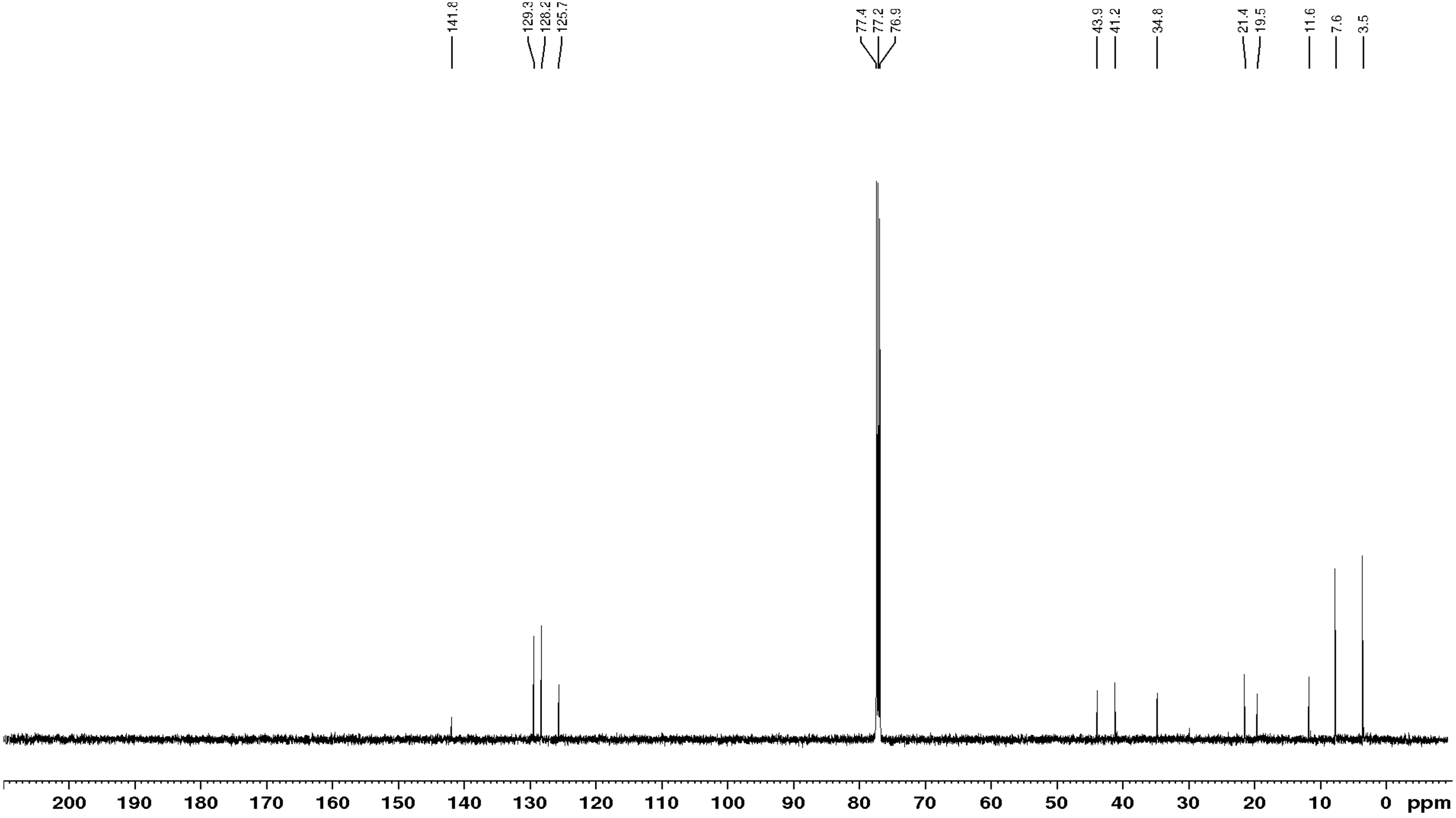

Figure S82. **$^{1}H/^{29}Si$ HMQC NMR** spectrum (500/99 MHz, $CDCl_3$, 298 K, optimized for J = 7 Hz) of **4ab** from the reaction of VCP **1a** and Et_2SiH_2 (**2a**) using $Ph_3C^+[B(C_6F_5)_4]^-$ as initiator.

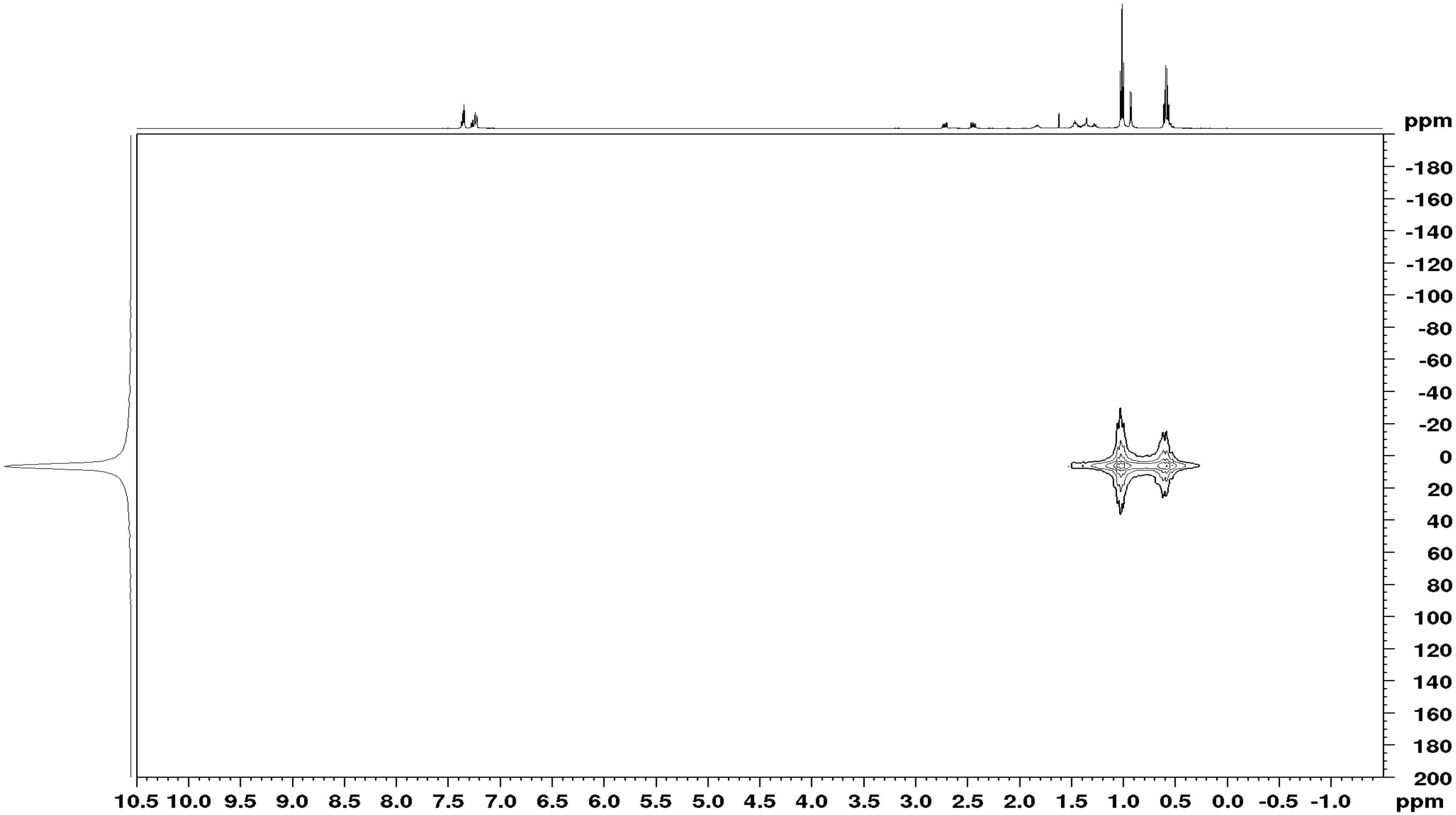

7 Crystallographic Data of 3qa

Data for the single-crystal structure determination were collected with an *Agilent* SuperNova diffractometer equipped with a CCD area Atlas detector and a mirror monochromator by utilizing Cu-Kα radiation (λ = 1.5418 Å). Software packages used: CrysAlis PRO for data collection, cell refinement, and data reduction,[S22] SHELXS-97 for structure solution,[S23] SHELXL-97 for structure refinement,[S24] and Mercury[S25] for graphics.

Molecular Structure of **3qa** (CCDC **2034954**)

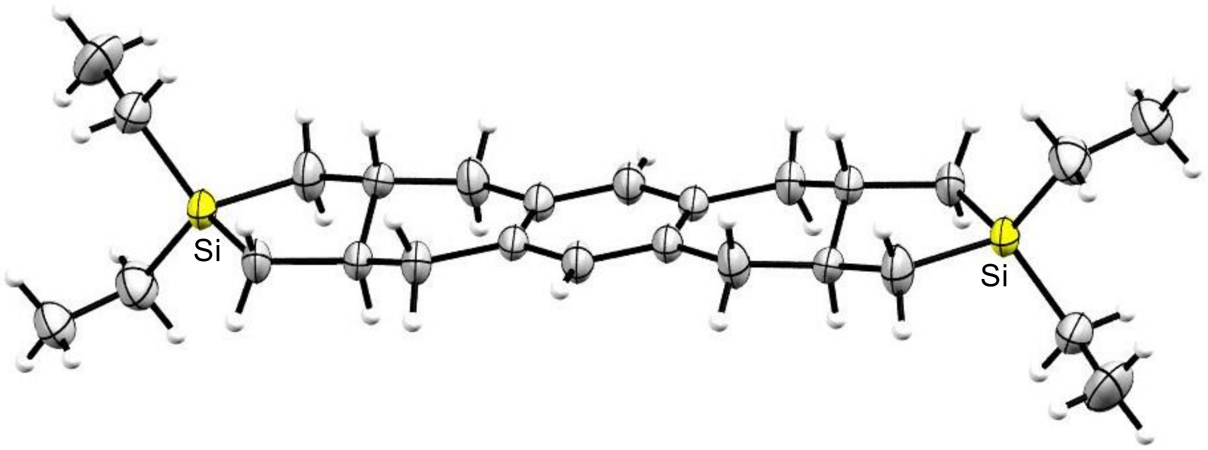

Figure S83. Molecular structure of **3qa** (thermal ellipsoids are shown at the 50% probability level). The crystal consists of a mixture of the two bis-*trans*-isomers *dl*-**3qa** and *meso*-**3qa** (dr = 53:47). Due of this mixture, the positions of the atoms C2 and C3 were refined by a split atom model. Only the molecular structure of *meso*-**3qa** is shown for clarity.

Empirical formula	$C_{26}H_{42}Si_2$	
Formula weight	410.78	
Temperature	150.01(10) K	
Wavelength	1.54184 Å	
Crystal system	Monoclinic	
Space group	$P2_1/c$	
Unit cell dimensions	a = 12.1094(5) Å	α = 90°
	b = 11.0077(4) Å	β = 103.617(4)°
	c = 9.4271(3) Å	γ = 90°
Volume	1221.28(8) $Å^3$	
Z	2	
Density (calculated)	1.117 Mg/m^3	
Absorption coefficient	1.360 mm^{-1}	
F(000)	452	
Crystal size	0.22 × 0.17 × 0.07 mm^3	

Theta range for data collection	3.76 to 67.50°
Index ranges	–14<=h<=9, –11<=k<=13, –10<=l<=11
Reflections collected	4197
Independent reflections	2195 [R(int) = 0.0199]
Completeness to theta = 67.50°	100.0 %
Absorption correction	Semi-empirical from equivalents
Max. and min. transmission	0.9132 and 0.7559
Refinement method	Full-matrix least-squares on F^2
Data / restraints / parameters	2195 / 12 / 149
Goodness-of-fit on F^2	1.053
Final R indices [I>2sigma(I)]	R1 = 0.0430, wR2 = 0.1145
R indices (all data)	R1 = 0.0493, wR2 = 0.1202
Largest diff. peak and hole	0.275 and –0.272 e.Å^{-3}

8 Computational Data

All density functional theory (DFT) calculations were performed with the Gaussian 16 package.[S26] The 3D structures of the optimized species were generated using CYLview.[S27] Geometry optimization and vibrational frequency calculations of all stationary points were carried out at M06-2X[S28]/6-31G(d,p) level of theory. To get more accurate energies, single-point energies were computed at M06-2X/cc-PVTZ level for all the species along the reaction pathway. The solvent effect was treated with the polarizable continuum model (PCM) in both geometry optimization and single-point calculation,[S29] with chlorobenzene as solvent. To simplify the computations without sacrificing the understanding of the reaction mechanism, the counteranion $[B(C_6F_5)_4]^-$ was not taken into consideration because the influence of the counteranion on relative energies could be eliminated by error cancellation. Activation free energy barriers here are defined as the free energy difference between the transition state and the lowest-energy stationary point before it along the reaction pathway.

8.1 Results of the DFT Calculations

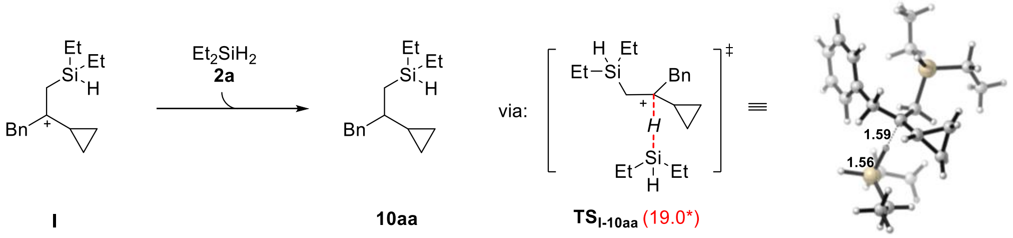

Figure S84. Intermolecular hydride transfer from Et_2SiH_2 (**2a**) to the benzylic *β*-silylcarbenium ion **I**. The computed activation barrier (referring to **I**, labeled with an asterisk) is given in kcal/mol.

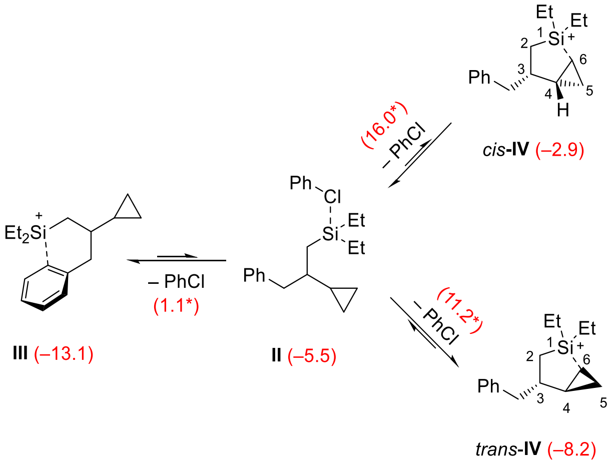

Scheme S9. General equilibria of inter- and intramolecular donor-stabilized silylium ions. The computed Gibbs free reaction energies and barriers (labeled with an asterisk) are given in kcal/mol.

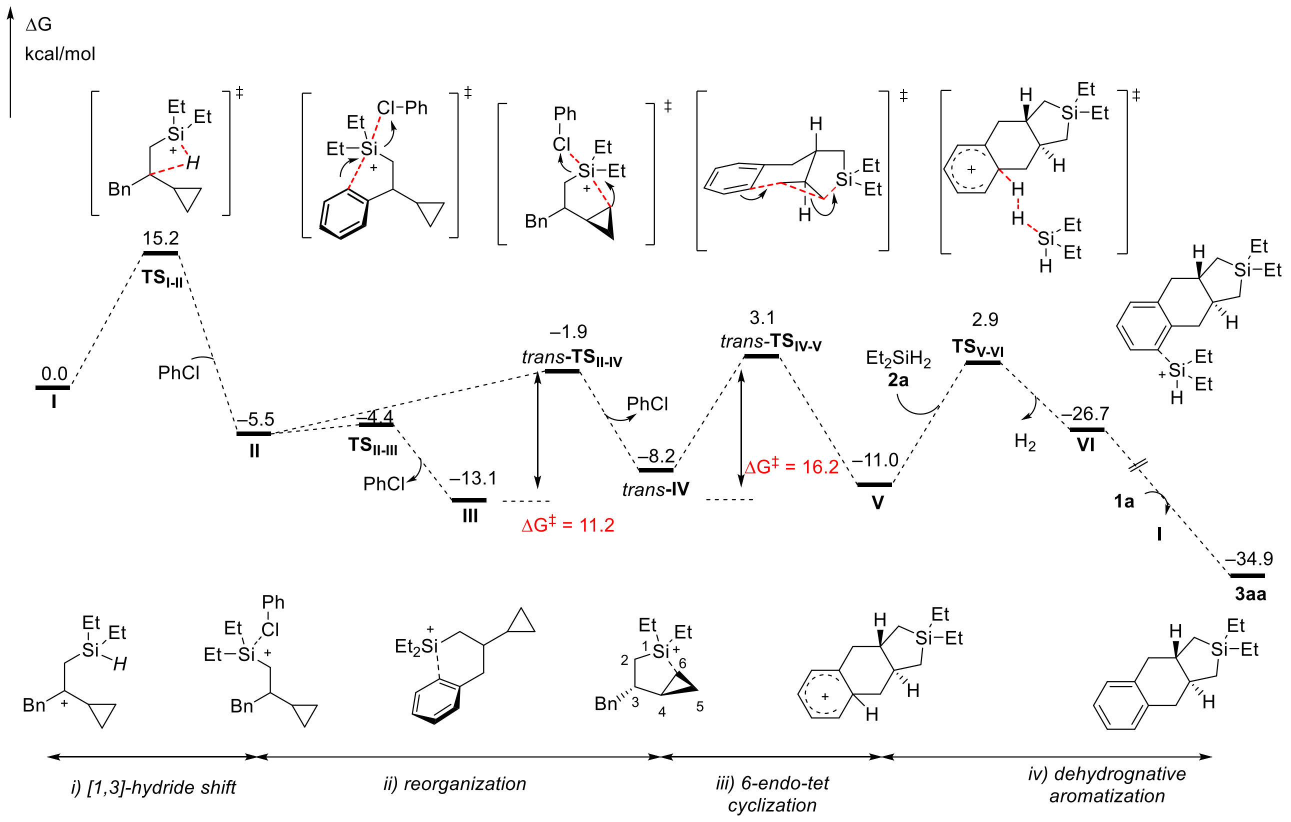

Figure S85. Gibbs free energy profile for the transformation of **I** to product **3aa** with Et_2SiH_2 (**2a**) via a 6-*endo*-*tet* cyclization (path a).

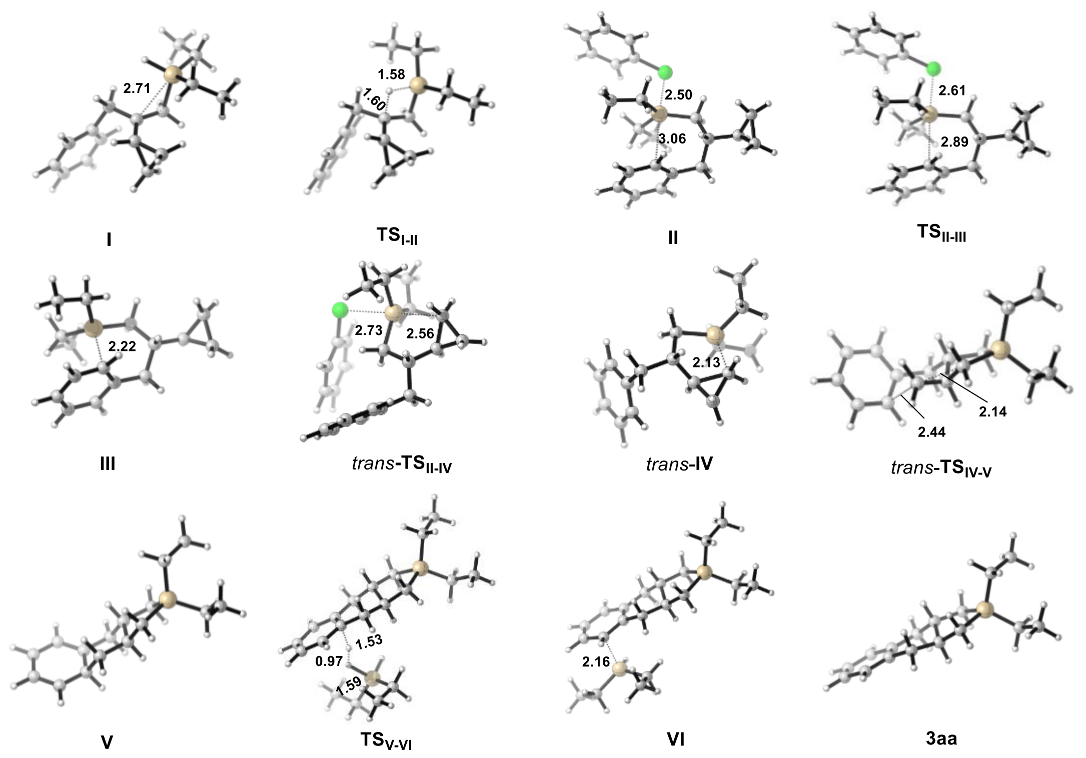

Figure S86. Optimized 3D structures of the intermediates and transition states involved in path a (with Et_2SiH_2 (**2a**), distances are in Å). Color code: H, white; C, gray; Si, brown; Cl, green.

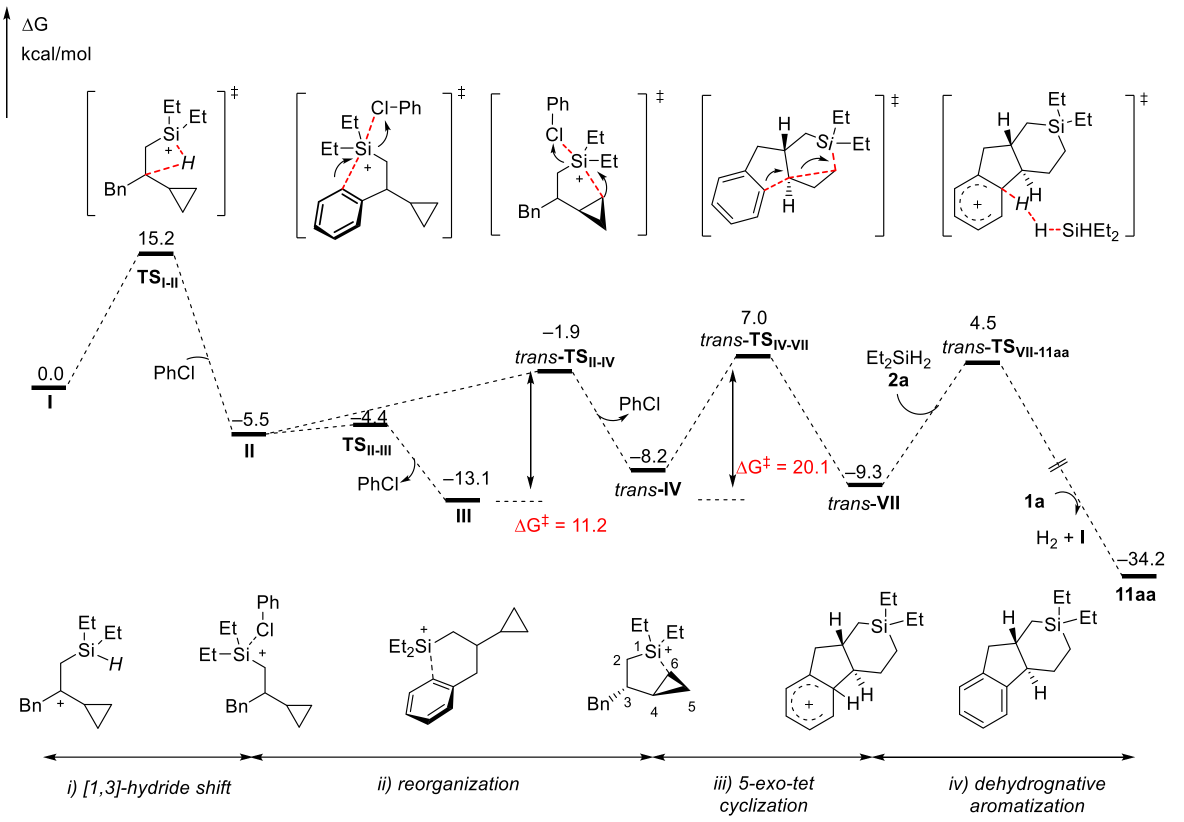

Figure S87. Gibbs free energy profile for the transformation of **I** to **11aa** with Et_2SiH_2 (**2a**) via a 5-*exo*-*tet* cyclization (path b).

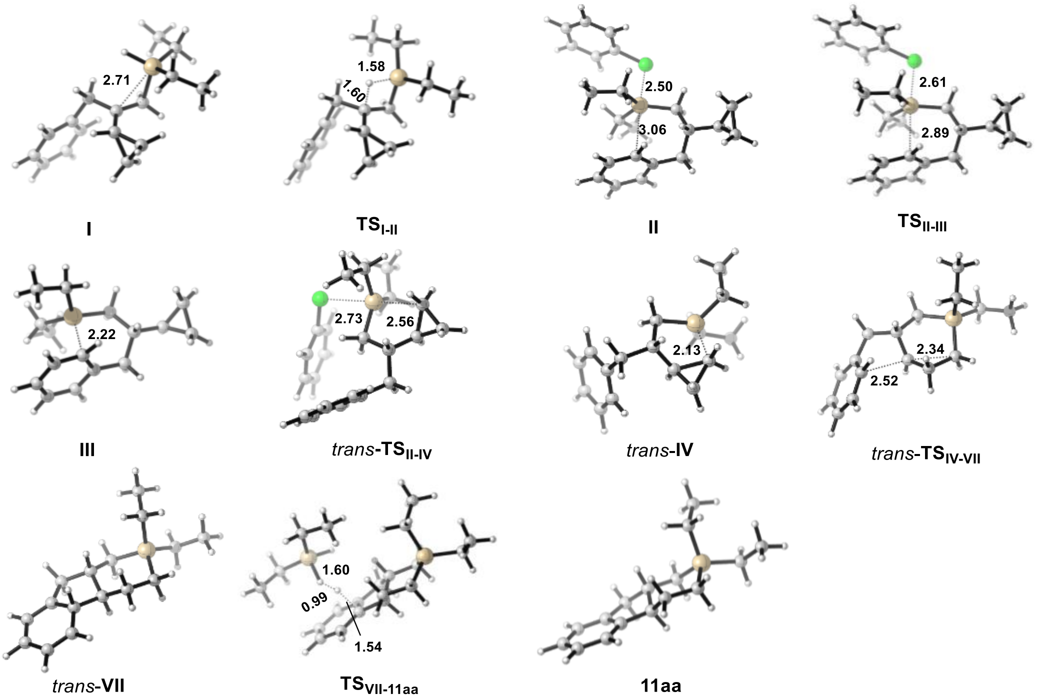

Figure S88. Optimized 3D structures of the intermediates and transition states involved in path a (with Et_2SiH_2 (**2a**), distances are in Å). Color code: H, white; C, gray; Si, brown; Cl, green.

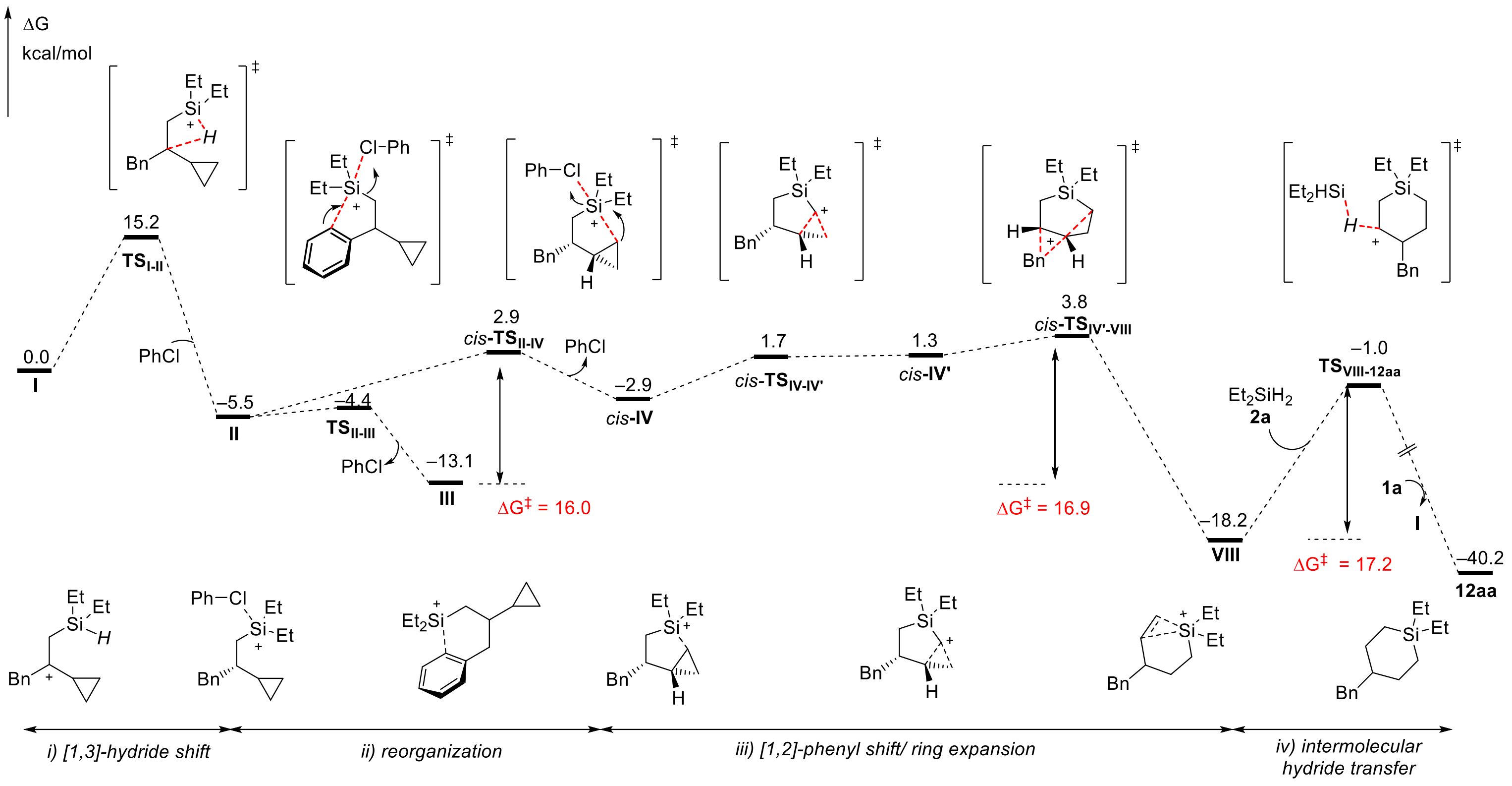

Figure S89. Gibbs free energy profile for the transformation of **I** to **12aa** with Et_2SiH_2 (**2a**) via a [1,2]-benzyl shift/ring expansion (path c).

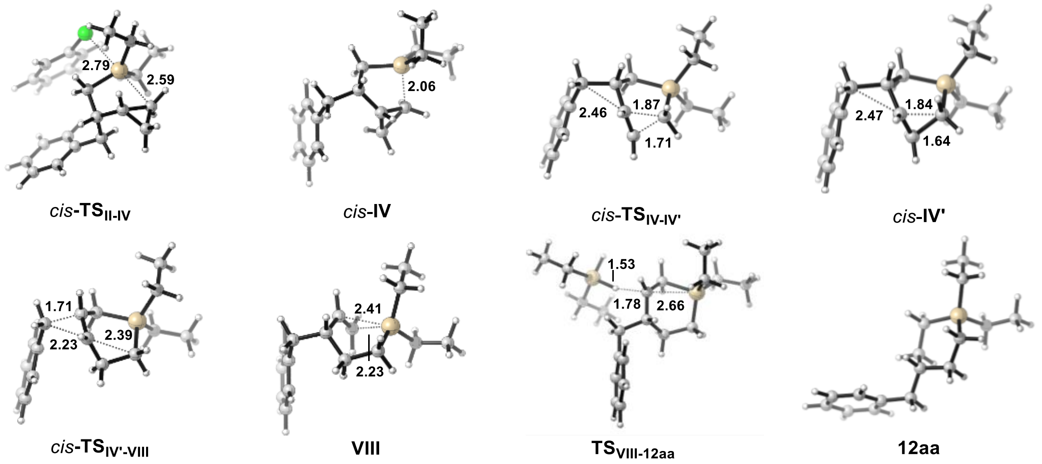

Figure S90. Optimized 3D structures of the intermediates and transition states involved in path c (with Et_2SiH_2 (**2a**), distances are in Å). Color code: H, white; C, gray; Si, brown; Cl, green.

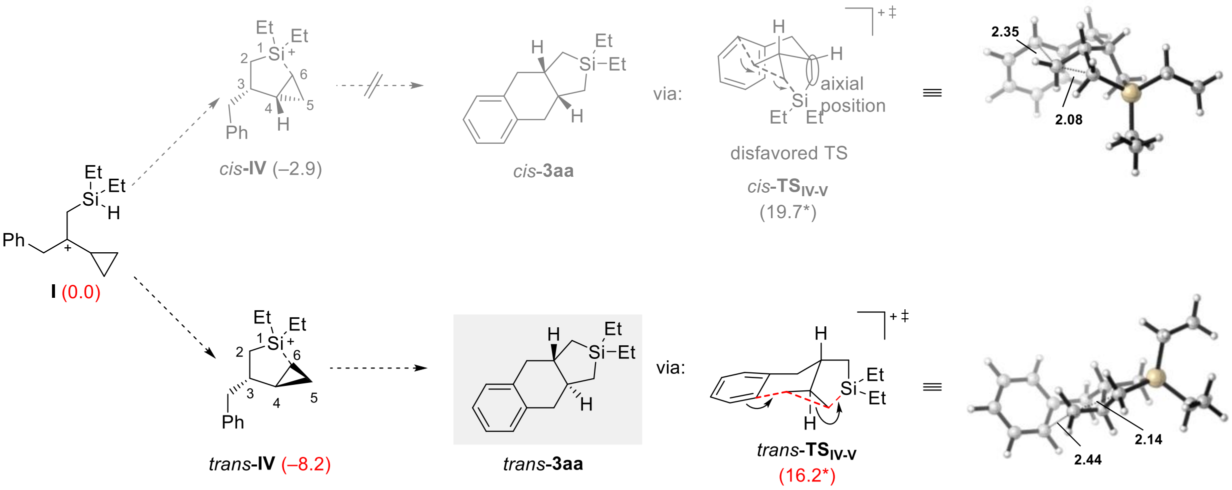

Scheme S10. Key transition states for the 6-*endo*-*tet* cyclization pathway with *cis*- and *trans*-**IV**: *cis*-**IV** produces *cis*-**3aa** (via *cis*-$\mathbf{TS}_{IV\text{-}V}$) and *trans*-**IV** gives *trans*-**3aa** (via *trans*-$\mathbf{TS}_{IV\text{-}V}$).

As shown in Scheme S10, there is a direct relationship between the relative configuration of bicyclo[3.1.0]hex-2-silyl cation intermediate **IV** and the configuration of tricyclic product **3aa**. Our computational results indicated that the more stable *trans*-bicyclo[3.1.0]hex-2-silyl cation **IV** produces *trans*-**3aa** with a lower activation barrier (via *trans*-$\mathbf{TS}_{IV\text{-}V}$), and therefore, the observed tricyclic product **3aa** should be in *trans* ring junction with the two H atoms in axial position.

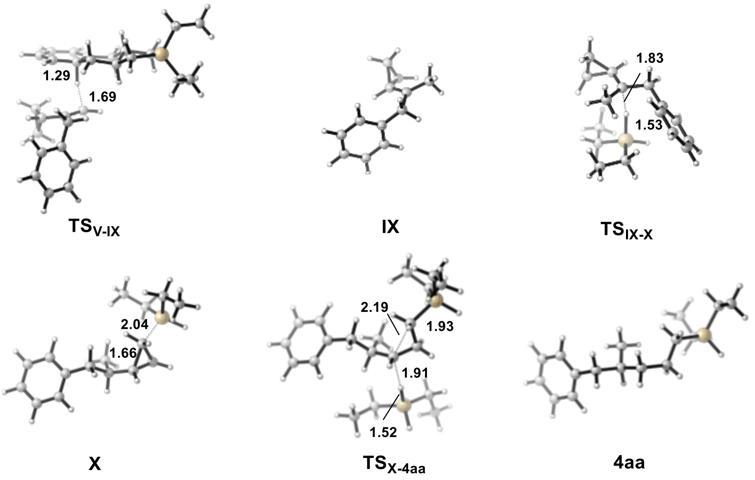

Figure S91. Optimized 3D structures of the intermediates and transition states involved in the formation of **4aa** with Et_2SiH_2 (**2a**), distances are in Å). Color code: H, white; C, gray; Si, brown.

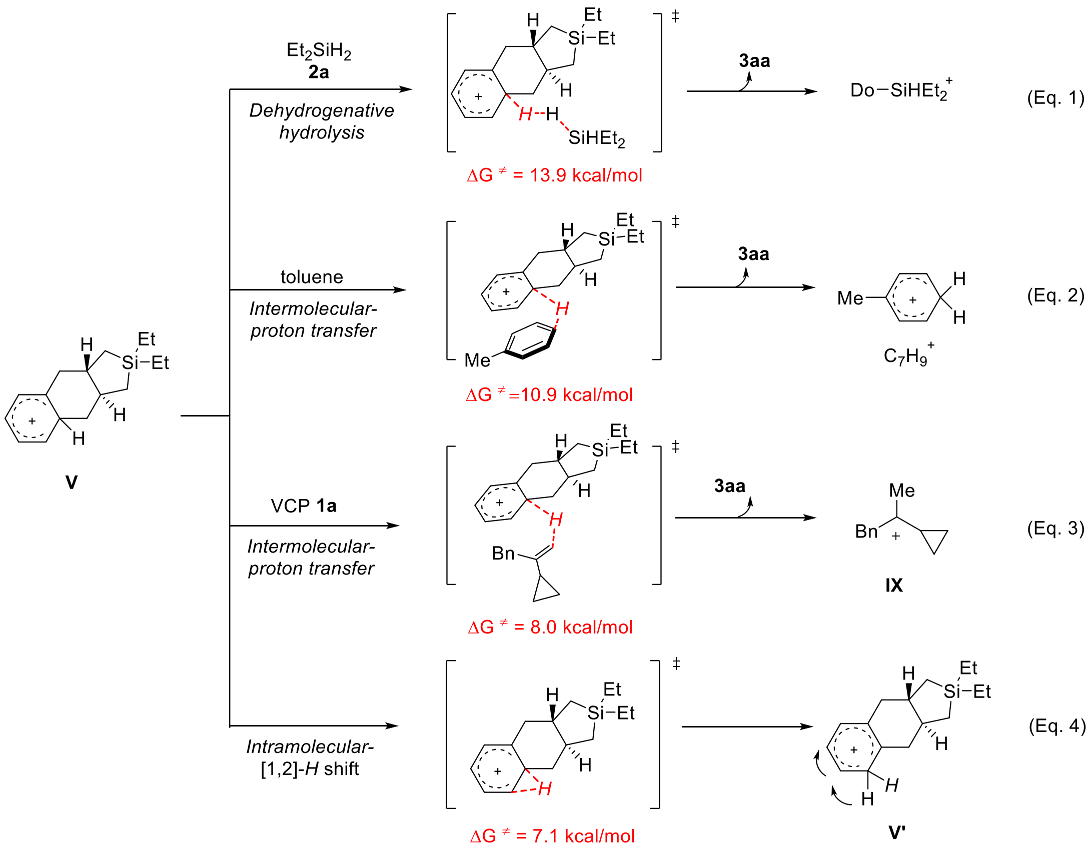

Scheme S11. Calculated activation barriers of the deprotonation of tricyclic Wheland intermediate **V** via dehydrogenative hydrolysis (Eq. 1), intermolecular proton transfer (Eq. 2 and Eq. 3, with toluene and VPC **1a** as acceptor, respectively), and intramolecular [1,2]-H shift (Eq. 4).

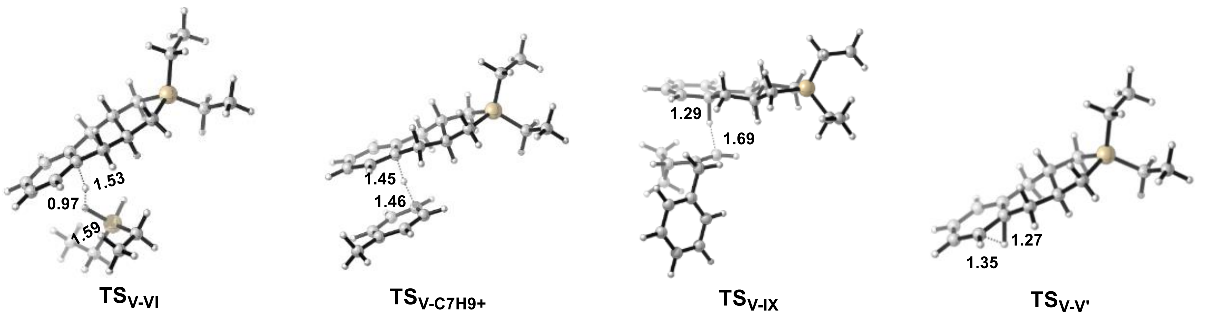

Figure S92. Optimized 3D structures of the transition states involved in Scheme S11. Color code: H, white; C, gray; Si, brown.

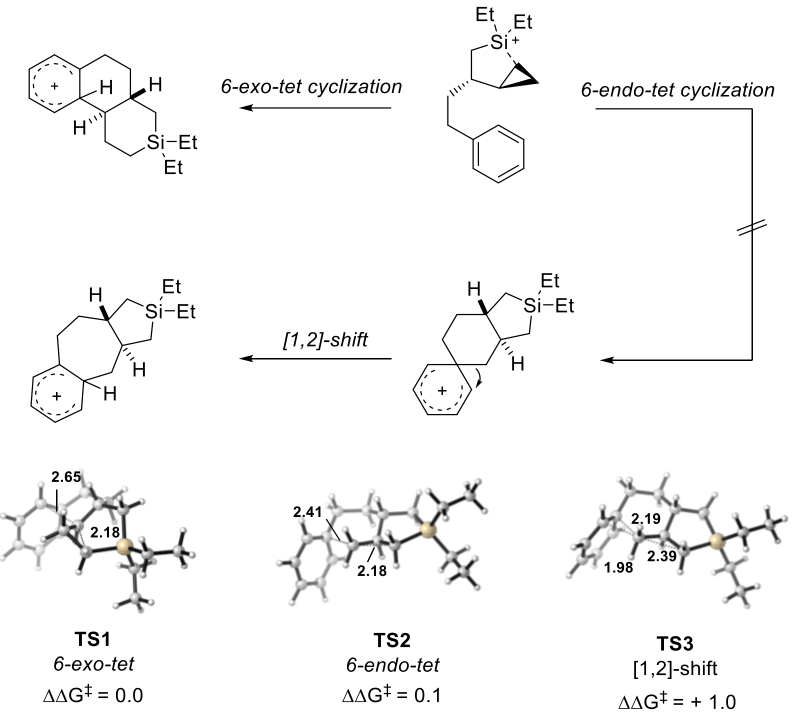

Scheme S12. Mechanistic explanation for the formation of the 6/6/6-fused ring system from phenethyl-substituted VCP **7** (top) and comparison of the key annulation transition states (bottom). The relative activation barrier is given with respect to 6-*exo-tet* cyclization transition state **TS1**, in kcal/mol). Color code: H, white; C, gray; Si, brown; Cl, green.

As shown in Scheme S12, the formation of the 6/6/6-fused ring system with phenethyl-substituted VCP **7** is kinetically controlled, in which the related 6-*exo-tet* cyclization transition state requires a lower activation barrier than the pathway related to the formation of the 6/7/5-fused ring system ($\Delta\Delta G^{\ddagger}$ = +1.0 kcal/mol).

Table S1. M06-2X/6-31G(d,p) computed free energies in benzene (G, in a.u.), M06-2X/cc-pVTZ calculated single point energies in benzene (E_{sol}, in a.u.), and corrected free energies (G_c, in a.u.) for all stationary points involved.

Species	G	E_{sol}	G_c
**I** → **3aa** (via *trans*-**IV**) with Et_2SiH_2 (**2a**)			
**1a**	-465.27522	-465.61589	-465.42801
**2a**	-448.90774	-449.10867	-448.99211
**I**	-913.41379	-913.96732	-913.64381
**TS$_{I-II}$**	-913.38965	-913.94536	-913.61960
**II**	-1605.07829	-1605.83865	-1605.42636
**TS$_{II-III}$**	-1605.07698	-1605.83812	-1605.42464
**III**	-913.43748	-913.99506	-913.66465
trans-**TS$_{II-IV}$**	-1605.07169	-1605.83142	-1605.42053
trans-**IV**	-913.42761	-913.98615	-913.65682
trans-**TS$_{IV-V}$**	-913.41156	-913.96639	-913.63881
**V**	-913.43729	-913.99153	-913.66134
**TS$_{V-VI}$**	-1362.32233	-1363.09095	-1362.63123
**VI**	-1361.20135	-1361.95997	-1361.50833
H_2	-1.16858	-1.16899	-1.17008
**3aa**	-913.07692	-913.62373	-913.30579
cis-**TS$_{IV-V}$**	-913.40641	-913.96284	-913.63327
**I** → **10aa** (via intermolecular hydride transfer) with Et_2SiH_2 (**2a**)			
**TS$_{I-10aa}$**	-1362.29446	-1363.07017	-1362.60562
**10aa**	-914.20054	-914.76751	-914.43287
trans-**IV** → **11aa** (via 5-*exo*-*tet* cyclization of *trans*-**IV**)			
trans-**IV**	-913.42761	-913.42761	-913.42761
trans-**TS$_{IV-VII}$**	-913.40495	-913.95981	-913.63266
trans-**VII**	-913.43443	-913.98701	-913.65856
trans-**TS$_{VII-11aa}$**	-1361.19935	-1361.95846	-1361.50634
**11aa**	-913.07558	-913.62309	-913.30449

**I** → **12aa** (via *cis*-**IV**) with Et_2SiH_2 (**2a**)			
cis-$\mathbf{TS_{II\text{-}IV}}$	-1605.06488	-1605.82687	-1605.41288
cis-**IV**	-913.41958	-913.97739	-913.64849
cis-$\mathbf{TS_{IV\text{-}IV'}}$	-913.41207	-913.96785	-913.64110
cis-**IV'**	-913.41271	-913.96819	-913.64171
cis-$\mathbf{TS_{IV'\text{-}VIII}}$	-913.40903	-913.96399	-913.63769
**VIII**	-913.44349	-914.00247	-913.67290
cis-$\mathbf{TS_{VIII\text{-}12aa}}$	-1362.32538	-1363.10237	-1362.63764
**12aa**	-914.41634	-914.81945	-914.48416
**V** → **4aa** with Et_2SiH_2 (**2a**)			
**V**	-913.43729	-913.99153	-913.66134
$\mathbf{TS_{V\text{-}IX}}$	-1378.70372	-1379.612322	-1379.076544
**IX**	-465.672471	-466.02015	-465.81979
$\mathbf{TS_{IX\text{-}X}}$	-914.56336	-915.13084	-914.79244
**X**	-914.59214	-915.16736	-914.82260
$\mathbf{TS_{X\text{-}4aa}}$	-1363.47508	-1364.26900	-1363.78841
**4aa**	-915.41506	-915.99951	-915.64767
Intra- and intermolecular proton transfer			
$\mathbf{TS_{V\text{-}VI}}$	-1362.32233	-1363.09095	-1362.63123
$\mathbf{TS_{V\text{-}C_7H_9+}}$	-1184.77075	-1185.52493	-1185.08055
$\mathbf{TS_{V\text{-}IX}}$	-1378.70372	-1379.612322	-1379.076544
$\mathbf{TS_{V\text{-}V'}}$	-913.381707	-913.9344087	-913.610351
Intramolecular annulation of phenethyl-substituted VCP **7**			
**TS1**	-952.67892	-953.27335	-952.91842
**TS2**	-952.67937	-953.27348	-952.91829
**TS3**	-952.67944	-953.27375	-952.91675

8.2 Cartesian Coordinates of the Optimized Stationary Points

1a

C	2.59548100	2.24141800	-0.31499100
C	2.31360600	3.44742000	-0.80839700
H	1.92444200	3.57381700	-1.81367900
H	2.46573600	4.34598900	-0.21764700
C	2.37424300	0.97123600	-1.06703900
C	1.09477900	0.20527900	-0.83591900
C	1.35971900	0.86044900	-2.16302000
H	3.26434500	0.35379200	-1.16889200
H	0.35366200	0.67076800	-0.19426700
H	1.14740200	-0.87536800	-0.76983600
H	1.59737300	0.22556100	-3.00860800
H	0.77460100	1.73994400	-2.40716900
C	3.16834000	2.06163400	1.07475600
H	2.50049500	1.41497200	1.65651000
H	3.21162400	3.03257500	1.57944700
C	4.55035500	1.43982000	1.04176900
C	5.60935400	2.12687100	0.43980500
C	4.79155200	0.17582300	1.58126700
C	6.88231600	1.56864400	0.39035600
H	5.42425500	3.10594100	0.00432200
C	6.06560500	-0.38898300	1.53167300
H	3.97374800	-0.36975800	2.04505300
C	7.11448500	0.30671400	0.93774400
H	7.69508000	2.11636800	-0.07635600
H	6.23652100	-1.37283400	1.95735500
H	8.10710600	-0.13025600	0.89924300

2a

Si	4.18955100	3.92377900	0.89352700
H	4.26406300	3.13635000	2.15561200
H	2.74541400	4.11589500	0.57785300
C	4.97915500	5.61281900	1.17370200
H	6.05466200	5.47358900	1.33590200
H	4.88214600	6.20207400	0.25427600
C	5.01629100	2.98332300	-0.51587900
H	6.05161100	2.76325600	-0.23073400
H	4.51788200	2.01430900	-0.63114600
C	4.35688600	6.37087600	2.35281000
H	4.47023200	5.81140800	3.28627400
H	4.82226000	7.35038700	2.49559100
H	3.28605400	6.53374000	2.19660700
C	4.98606700	3.75603600	-1.84098700
H	5.44066500	3.18201000	-2.65343900
H	3.95972100	3.99344000	-2.13832100
H	5.53143600	4.70136100	-1.76177800

I

C	1.01423700	0.56993700	0.46457000
C	1.07672200	1.91151300	0.01390300
H	1.98466100	2.22989600	-0.50210600
H	0.16268900	2.26651600	-0.46679700
C	2.16190000	-0.27546700	0.60871500
C	2.99025300	-0.45014500	-0.70266200
C	3.59100600	0.23103700	0.44717600
H	2.01669800	-1.19210300	1.16844100
H	2.65552200	0.13732200	-1.54973200
H	3.24430800	-1.48163300	-0.91295500

H	4.31314000	-0.30645200	1.04967900
H	3.74033600	1.30329100	0.39892300
Si	1.04723300	2.96928100	1.72740300
H	0.11410800	2.23701800	2.61448100
C	2.78252100	3.04220700	2.42174400
H	2.68570900	3.46356700	3.42988600
H	3.16957900	2.02705600	2.56230200
C	0.35689300	4.62922700	1.20238900
H	0.51922000	5.30890800	2.04861000
H	0.96459300	5.01869700	0.37760600
C	3.74517800	3.90188500	1.59089900
H	3.40808200	4.94112400	1.55208100
H	4.74879400	3.89411800	2.02161700
H	3.83087900	3.55243000	0.55601100
C	-1.12787100	4.60112100	0.82196900
H	-1.74223100	4.23353100	1.64800200
H	-1.48137800	5.60126500	0.56151900
H	-1.31490000	3.95818400	-0.04382400
C	-0.31917000	-0.03200500	0.79797700
H	-0.27414500	-0.47859200	1.79647200
H	-1.09923300	0.73346900	0.79184000
C	-0.60065500	-1.11138800	-0.23956300
C	-0.44684700	-2.46107500	0.07938700
C	-0.97142000	-0.74700900	-1.53597200
C	-0.66878700	-3.43810300	-0.88763600
H	-0.16817800	-2.74920900	1.08908200
C	-1.19259300	-1.72439500	-2.50159300
H	-1.09793600	0.30215200	-1.78981600
C	-1.03919700	-3.07124100	-2.17918900
H	-0.55699000	-4.48576000	-0.62948600
H	-1.48678600	-1.43419300	-3.50453700
H	-1.21299400	-3.83294200	-2.93164300

TS$_{I-II}$

C	0.99784400	0.77313800	0.79129300
C	1.34747600	1.82485400	-0.18741400
H	2.31311600	1.71945700	-0.67801000
H	0.55089700	2.05791300	-0.89169900
C	2.02927500	-0.16869300	1.26745000
C	2.76934200	-0.94651700	0.18478800
C	3.50715400	0.02933100	1.02642900
H	1.74837100	-0.73695200	2.14820800
H	2.55343600	-0.68165800	-0.84445500
H	2.88628600	-2.00707200	0.37132500
H	4.15963700	-0.34684900	1.80493600
H	3.84467400	0.94942200	0.56183100
Si	1.36042500	2.94067900	1.34602100
H	0.86716700	1.65790400	2.12003500
C	3.05349600	3.34733000	2.02175200
H	2.88991200	4.00659100	2.88115700
H	3.52021200	2.43843000	2.41168800
C	0.01998400	4.23763300	1.43119300
H	0.02410000	4.62169100	2.45656800
H	0.38266600	5.05573500	0.79524000
C	3.94972700	4.03237800	0.97847900
H	3.48997900	4.94516600	0.58987900
H	4.90748000	4.30823500	1.42435100
H	4.15675200	3.37640800	0.12808000
C	-1.39310400	3.81926200	1.01305500
H	-1.80640800	3.07501400	1.69812900
H	-2.05906600	4.68443900	1.02581200

H	-1.41387200	3.40789700	-0.00043800
C	-0.43930000	0.27429100	0.83001800
H	-0.63473500	-0.20787400	1.79106600
H	-1.12796900	1.11435900	0.71908600
C	-0.63213800	-0.70688600	-0.31235700
C	-0.31702400	-2.05771200	-0.14368000
C	-1.11123600	-0.26713000	-1.54802600
C	-0.47164500	-2.95358900	-1.19647300
H	0.04787600	-2.40899400	0.81778000
C	-1.26629200	-1.16346000	-2.60291800
H	-1.38541600	0.77601500	-1.68433200
C	-0.94342000	-2.50663500	-2.42968900
H	-0.22923000	-4.00120000	-1.05276300
H	-1.64620100	-0.81232100	-3.55641600
H	-1.06643900	-3.20561000	-3.25015400

II

C	2.01597000	1.33639700	0.72333900
C	3.06370300	1.78208000	-0.27570100
C	1.68444800	2.50893100	1.67150100
C	4.51139100	1.77794300	0.11660700
C	3.99339300	0.76528800	-0.86869600
H	2.73930500	2.58763600	-0.93199300
C	0.78351300	0.80464500	-0.01755400
Si	0.60744100	2.20537300	3.17015100
H	1.23667200	3.32916000	1.09275300
H	2.63505200	2.89165300	2.06565300
H	5.14753400	2.57339900	-0.25308200
H	4.75809800	1.42587400	1.11398500
H	4.27569000	0.87127700	-1.90938600
H	3.89287300	-0.26070500	-0.52787000
C	-1.20636900	2.59847100	3.20204600
C	1.26324400	1.17240800	4.58149600
C	-1.67801100	3.61013500	2.15299800
H	-1.73080500	1.64211900	3.08336000
H	-1.44578300	2.93558000	4.21828100
C	0.21260600	0.86260800	5.65857700
H	1.66198900	0.24404400	4.15896100
H	2.12669100	1.69037500	5.01448200
H	-1.43653700	3.27385200	1.14087600
H	-1.21827300	4.59156500	2.30177100
H	-2.76132200	3.73774700	2.20868200
H	-0.64621300	0.33566200	5.23262500
H	-0.15704800	1.77268200	6.14020500
H	0.64433700	0.22811400	6.43555800
H	2.46271600	0.52320400	1.31223300
C	1.32809600	3.77470000	8.02996600
C	0.02372500	4.09838500	8.39860200
C	-0.88273800	4.56448500	7.44877500
C	-0.49968400	4.69740200	6.11537300
C	0.79927900	4.34248400	5.78189300
C	1.73544200	3.89685100	6.70356600
H	2.03668800	3.42556000	8.77247800
H	-0.28510600	3.99550700	9.43274800
H	-1.89281600	4.83019900	7.73927500
H	-1.18717300	5.07065900	5.36425000
H	2.74732800	3.65538300	6.39806700
Cl	1.28788700	4.43708400	4.07831300
H	0.37921100	1.60456800	-0.65011500
C	-0.31724100	0.26816200	0.86705900
C	-1.66156600	0.52216700	0.56869000

C	-0.02620800	-0.54207800	1.97477400
C	-2.68199800	-0.01289700	1.34928300
H	-1.90640500	1.13711600	-0.29312400
C	-1.04791300	-1.07102400	2.76785600
H	1.00399200	-0.81567300	2.18646300
C	-2.37872400	-0.80632000	2.45758200
H	-3.71704900	0.19237700	1.09635300
H	-0.79872700	-1.70559800	3.61259600
H	-3.17472700	-1.22064000	3.06680400
H	1.11709500	0.01075700	-0.69703300

TS$_{II-III}$

C	2.02520100	1.31960000	0.70518800
C	3.05741700	1.79365500	-0.29565500
C	1.68953500	2.46280600	1.68423200
C	4.50963500	1.79062000	0.07976400
C	3.98716200	0.79625000	-0.92134900
H	2.71988800	2.61119400	-0.93017100
C	0.78301700	0.79993500	-0.02663300
Si	0.57674700	2.10622300	3.14385100
H	1.25094600	3.30244800	1.12694500
H	2.63276100	2.82820900	2.10998300
H	5.13562100	2.59853500	-0.28002600
H	4.77025400	1.41964300	1.06660500
H	4.25650300	0.92730000	-1.96260300
H	3.89733300	-0.23771900	-0.60251700
C	-1.21272400	2.59859500	3.19025400
C	1.24126700	1.13818200	4.59660700
C	-1.63434000	3.62257700	2.13165400
H	-1.79719300	1.67568900	3.09356900
H	-1.41554400	2.96583400	4.20392000
C	0.19221100	0.85816500	5.68282600
H	1.66658100	0.20341200	4.21663000
H	2.08900700	1.69722100	5.00989800
H	-1.43172800	3.25580200	1.12155600
H	-1.10895500	4.57355300	2.25745500
H	-2.70592300	3.82157300	2.20186500
H	-0.66454300	0.31255600	5.27722300
H	-0.18209800	1.78289400	6.13161100
H	0.62739200	0.25427200	6.48195100
H	2.48221000	0.49721400	1.27309900
C	1.30589600	3.75595000	8.03652000
C	0.00073200	4.08552200	8.39680800
C	-0.89454200	4.56068100	7.44098000
C	-0.49924500	4.69613700	6.11143500
C	0.80018500	4.33552600	5.78287300
C	1.72390800	3.88126800	6.71377300
H	2.00672500	3.39973400	8.78313800
H	-0.31706600	3.98040900	9.42804000
H	-1.90543400	4.83161200	7.72404800
H	-1.17901200	5.07651800	5.35672000
H	2.73710400	3.63536300	6.41608700
Cl	1.30000800	4.42999800	4.08745500
H	0.37976300	1.60285000	-0.65579000
C	-0.31135000	0.27978500	0.87426300
C	-1.65963500	0.50939700	0.57054400
C	-0.00959900	-0.49332100	2.00728400
C	-2.67146100	-0.02290700	1.36246400
H	-1.91237600	1.09915100	-0.30630100
C	-1.02694900	-1.02351600	2.80977100
H	1.02228600	-0.76201500	2.21678400

C	-2.35914300	-0.78830200	2.48960300
H	-3.70914000	0.16040100	1.10369400
H	-0.76927300	-1.63670200	3.66765200
H	-3.15080300	-1.20241100	3.10437300
H	1.09775300	-0.00022900	-0.70760600

III

C	2.03823900	1.24684200	0.68327900
C	3.04659800	1.73708400	-0.33023200
C	1.54521800	2.38580000	1.59239600
C	4.49223000	1.83902900	0.05664600
C	4.04363000	0.77294400	-0.90484700
H	2.66741900	2.50590400	-1.00061400
C	0.83835700	0.59822900	-0.02522500
Si	0.32731200	1.82671800	2.90545800
H	1.07655000	3.15974400	0.97148500
H	2.39826100	2.85082700	2.10021800
H	5.06900600	2.66934200	-0.33289100
H	4.76487700	1.52905100	1.06088200
H	4.31533900	0.87621100	-1.94857300
H	4.01549100	-0.25006200	-0.54235600
C	-1.30296000	2.73707500	2.99208600
C	1.15482200	1.54125400	4.55600300
C	-1.58865300	3.66992100	1.80749300
H	-2.12044600	2.02185700	3.13005200
H	-1.26075300	3.31347700	3.92564300
C	0.29874300	0.98837900	5.69914100
H	2.06290600	0.94585800	4.40941500
H	1.50598400	2.55275000	4.81152600
H	-1.58963700	3.12874200	0.85675200
H	-0.84342300	4.46611100	1.73731100
H	-2.56710100	4.14172600	1.92164500
H	0.11872300	-0.08188100	5.58341400
H	-0.67204800	1.48881600	5.76146500
H	0.80427800	1.12806800	6.65712400
H	2.54441300	0.48983700	1.30061800
H	0.45539400	1.28138300	-0.79171300
C	-0.28769700	0.23095900	0.90327500
C	-1.62512800	0.36113100	0.51325800
C	-0.01728800	-0.25782200	2.21308200
C	-2.65341700	-0.05522500	1.35093000
H	-1.85380800	0.76498200	-0.46799100
C	-1.07764500	-0.72115200	3.03665900
H	0.99907800	-0.56331400	2.46262400
C	-2.38651400	-0.60528400	2.61321300
H	-3.68136200	0.03931100	1.01642100
H	-0.84704000	-1.17495400	3.99323900
H	-3.19962900	-0.94841000	3.24187400
H	1.17013000	-0.30963300	-0.54422200

trans-$\mathbf{TS_{II\text{-}IV}}$

C	1.97404900	0.95829400	0.82275000
C	1.74836700	1.79114500	2.06793700
C	0.62443400	0.87069900	0.09129900
C	1.01700000	1.08382600	3.23848300
C	2.46951500	1.55255500	3.32532600
H	1.42945700	2.81673400	1.88293300
C	3.09150500	1.52539800	-0.06615000
Si	-0.73364100	0.93744000	1.37395600
H	0.55022100	-0.01660400	-0.54440000

H	0.49284500	1.74650700	-0.55905400
H	0.30520800	1.61349200	3.86400700
H	0.93266700	0.00093400	3.22784400
H	2.66571900	2.39554400	3.97555500
H	3.21100400	0.76122100	3.34197500
C	-1.41189400	-0.61934400	2.15154900
C	-1.57374500	2.56112600	1.75066200
C	-0.73379600	-1.89554100	1.63521200
H	-1.30974000	-0.52866800	3.23854700
H	-2.49117300	-0.64910800	1.96126100
C	-2.94199700	2.39933600	2.42737100
H	-0.90642900	3.16200100	2.37727500
H	-1.66414200	3.10641800	0.80293600
H	0.34529500	-1.88535200	1.81925100
H	-0.88448300	-2.01839700	0.55930200
H	-1.14502300	-2.77566400	2.13285500
H	-2.85562100	1.86920600	3.37984000
H	-3.63961300	1.84458700	1.79357300
H	-3.38292400	3.37746600	2.62967700
H	2.26436600	-0.05208800	1.14299500
C	-0.73528800	2.40346100	-3.74392700
C	-1.35583300	3.65082600	-3.71873400
C	-2.33057800	3.92942600	-2.76399000
C	-2.69055700	2.96626200	-1.82319700
C	-2.04859200	1.73577800	-1.86970900
C	-1.07837500	1.42464000	-2.81390900
H	0.01797500	2.18235200	-4.49245500
H	-1.08220000	4.40508200	-4.44797100
H	-2.82023200	4.89668300	-2.74716300
H	-3.45703300	3.16269400	-1.08123100
H	-0.60117700	0.45050900	-2.82436300
Cl	-2.48999800	0.51270900	-0.67252500
H	2.85298500	2.56781600	-0.30881300
H	4.02781700	1.52699800	0.50261800
C	3.24385600	0.72285100	-1.33567200
C	2.66208700	1.15161200	-2.53180100
C	3.91413900	-0.50343000	-1.31691300
C	2.74551200	0.37127800	-3.68345800
H	2.14450200	2.10806500	-2.56280900
C	3.99940600	-1.28592000	-2.46519000
H	4.37748000	-0.84361400	-0.39372500
C	3.41269700	-0.85097000	-3.65215600
H	2.29813000	0.72149100	-4.60882400
H	4.52749500	-2.23356000	-2.43506900
H	3.48133800	-1.45805800	-4.54874900

trans-**IV**

C	0.60164900	-0.96481600	0.11889100
C	-0.15879300	0.19146800	-0.55874800
H	-0.08675600	0.15770500	-1.64816600
H	0.23341400	1.15739300	-0.21920900
C	-0.08117700	-1.16494200	1.46057100
C	-1.58431400	-1.72767600	1.32879900
C	-0.35541800	-2.44554500	2.04380200
H	-0.04183200	-0.31553600	2.14234000
H	-1.86675000	-2.38515700	0.50885000
H	-2.35229500	-1.50615700	2.06471700
H	-0.47354100	-2.52997300	3.11652500
H	-0.03624400	-3.33862800	1.51882800
Si	-1.90042500	-0.00904500	0.11263100
C	-2.47606900	1.33685800	1.26765400

H	-1.70449200	1.53544300	2.01980200
H	-2.51506200	2.24273800	0.64623400
C	-3.23111100	-0.61659800	-1.04430800
H	-2.82658400	-1.41849000	-1.67128400
H	-4.05304400	-1.04916400	-0.46281600
C	-3.84226500	1.08928400	1.92004900
H	-3.82816500	0.21408300	2.57622300
H	-4.14131100	1.94571700	2.52771000
H	-4.62069800	0.92797900	1.16883800
C	-3.74353200	0.54282600	-1.91771500
H	-2.93780700	0.99106500	-2.50674700
H	-4.50305300	0.18731700	-2.61709500
H	-4.19629300	1.33271100	-1.31129600
C	2.12334000	-0.75946200	0.26851700
H	0.44782200	-1.88110800	-0.46528900
H	2.55517500	-1.67922400	0.67743900
H	2.53710700	-0.62623400	-0.73576100
C	2.49684100	0.41246500	1.14428500
C	2.62014900	0.25068600	2.52862500
C	2.70388200	1.68291500	0.59824300
C	2.92744500	1.33304000	3.34897200
H	2.48513500	-0.73712900	2.96445000
C	3.01234500	2.76811800	1.41558200
H	2.63450600	1.81871600	-0.47827600
C	3.12140200	2.59608500	2.79340000
H	3.02413300	1.18867700	4.42014000
H	3.17452300	3.74634200	0.97476500
H	3.36534800	3.43977900	3.43038800

trans-**TS**$_{IV\text{-}V}$

C	0.25421000	0.62930300	0.65311200
C	-1.13189300	0.92502400	1.23778800
H	-1.32304500	1.99851900	1.31296300
H	-1.21894100	0.49805300	2.24427000
C	0.23422500	-0.83412100	0.24421300
C	-0.90729700	-1.06723700	-0.83597800
C	1.21679700	-1.29849500	-0.67179200
H	0.05230500	-1.51027800	1.08459400
H	-0.69882400	-0.60748300	-1.80758300
H	-1.18067900	-2.11488700	-0.95977500
H	1.43229900	-2.35863600	-0.77162300
H	1.59267900	-0.63659300	-1.44896200
Si	-2.29795200	0.03053200	0.02923300
C	-3.56022000	-1.09253700	0.84090900
H	-3.02952300	-1.81775000	1.46968700
H	-4.16168200	-0.48298200	1.52692100
C	-3.06451900	1.15775000	-1.25884200
H	-2.27867200	1.79600900	-1.68057600
H	-3.44351500	0.54684700	-2.08681700
C	-4.46629900	-1.81048500	-0.16853000
H	-3.88454200	-2.42045000	-0.86680300
H	-5.17569600	-2.47221300	0.33423300
H	-5.04454100	-1.09506400	-0.76109600
C	-4.19219000	2.01718600	-0.66717600
H	-3.82869600	2.64206500	0.15451900
H	-4.62141400	2.68164100	-1.42104300
H	-5.00286700	1.39540700	-0.27483400
C	1.48746400	0.88742800	1.54872600
H	0.38498700	1.22384000	-0.26396800
H	1.51394500	1.94496400	1.82500700
H	1.39909100	0.29839900	2.46803100

C	2.71714300	0.50389700	0.76804100
C	3.15118200	-0.83829800	0.73792600
C	3.32677100	1.41838400	-0.09348700
C	4.17847800	-1.24056800	-0.12958600
H	2.75438200	-1.54573500	1.46198200
C	4.34033600	1.01339400	-0.95839100
H	2.99563700	2.45306800	-0.09266400
C	4.76173200	-0.31816000	-0.98802500
H	4.51244800	-2.27290300	-0.12191700
H	4.80190200	1.73928000	-1.61970700
H	5.54875800	-0.62536300	-1.66755900

V

C	0.53101100	0.27533200	-0.54192000
C	-0.27691900	1.36958400	-1.25189600
H	-0.08893000	1.38420200	-2.32902500
H	0.00076300	2.35402000	-0.85375500
C	-0.08911200	0.03463100	0.84278300
C	-1.58951100	-0.25114800	0.63928400
C	0.62649300	-1.07783400	1.59752200
H	0.01644100	0.96803400	1.41775000
H	-1.72766900	-1.28754100	0.30442600
H	-2.15524700	-0.12805100	1.56695700
H	0.24217000	-1.19538000	2.61278200
H	0.51992600	-2.03232300	1.07086900
Si	-2.07637500	0.95065700	-0.76415600
C	-3.02868100	2.45141000	-0.14475200
H	-2.48332900	2.88262300	0.70399300
H	-3.02771800	3.22026900	-0.92724600
C	-3.01956600	0.09044000	-2.14761500
H	-2.39795500	-0.72818700	-2.53154600
H	-3.91575500	-0.38196100	-1.72674800
C	-4.46834600	2.11146400	0.26346100
H	-4.48956100	1.34278900	1.04299500
H	-4.99785700	2.98664100	0.64968800
H	-5.04118000	1.72873300	-0.58730600
C	-3.40745800	1.04441100	-3.28514100
H	-2.52495900	1.52629700	-3.71862800
H	-3.92743400	0.52223900	-4.09290700
H	-4.06952500	1.83907300	-2.92654100
C	2.04395400	0.59355200	-0.40919500
H	0.43428600	-0.66228000	-1.11116200
H	2.50531300	0.71043300	-1.39209300
H	2.14668200	1.53580100	0.14281500
C	2.67774200	-0.51604400	0.35037900
C	2.19439300	-0.75028200	1.72330100
C	3.56054700	-1.40884300	-0.22633600
C	2.81668900	-1.85042800	2.46700000
H	2.21671900	0.17806600	2.31039300
C	4.05542600	-2.47745500	0.52399300
H	3.87767900	-1.27769000	-1.25475300
C	3.69519600	-2.70518000	1.87295700
H	2.51296100	-1.99010900	3.50070600
H	4.75132900	-3.16453400	0.05092600
H	4.12333400	-3.54114000	2.41158300

TS$_{V-VI}$

C	0.57690400	-0.13597300	-0.62892000
C	-0.25002300	1.01898600	-1.22195900
H	-0.37604100	0.91761200	-2.30401200

H	0.27113500	1.96984900	-1.04469900
C	0.32178200	-0.19842000	0.88907200
C	-1.19765800	-0.17037800	1.13738800
C	1.00732600	-1.42181100	1.49375900
H	0.76421000	0.71359300	1.32928900
H	-1.60578400	-1.18009800	0.99590700
H	-1.43933000	0.13368500	2.16023000
H	0.95518600	-1.40250800	2.58846600
H	0.46860900	-2.32309600	1.17241400
Si	-1.86951100	0.98300400	-0.22210500
C	-2.36030200	2.68136500	0.43114000
H	-1.53510100	3.06959400	1.04142400
H	-2.46251300	3.37304300	-0.41426800
C	-3.30008000	0.21636900	-1.17938900
H	-2.95799800	-0.73432000	-1.60743500
H	-4.10459400	-0.03930700	-0.47878200
C	-3.65832400	2.64967100	1.24866300
H	-3.57677800	1.96242000	2.09730100
H	-3.91391500	3.63569900	1.64660000
H	-4.50188000	2.31256600	0.63762500
C	-3.83320200	1.13654700	-2.28545900
H	-3.04286500	1.40466100	-2.99446600
H	-4.63931000	0.66495000	-2.85434000
H	-4.22637800	2.06956300	-1.86872200
C	2.07108300	-0.01566000	-0.92202900
H	0.21237200	-1.08574000	-1.05161000
H	2.27773100	-0.16445400	-1.98699500
H	2.39774300	1.01021800	-0.69435300
C	2.93463800	-0.96814600	-0.12960500
C	2.45225300	-1.57864900	1.06145900
C	4.27048000	-1.18382100	-0.50713400
C	3.30199500	-2.47224800	1.77122300
C	5.10227100	-1.99120300	0.25045300
H	4.64856300	-0.69404000	-1.39925700
C	4.61431500	-2.65215200	1.39199400
H	2.90681900	-2.96771700	2.65330200
H	6.13699900	-2.12588900	-0.04687800
H	5.26628800	-3.30115700	1.96508600
H	3.28708700	-0.39197600	1.55397700
Si	3.89627800	1.56680000	2.56737000
H	3.25138500	2.27808500	1.44339800
H	4.01638100	0.08067700	1.99355200
C	5.70695000	1.96762900	2.76394500
H	5.76012200	3.00882500	3.10593200
H	6.12240600	1.35462100	3.56991100
C	2.83682700	1.34137500	4.08475500
H	2.83191700	2.29425900	4.62706200
H	1.80267200	1.16671400	3.76721900
C	6.50299100	1.78352500	1.46533800
H	6.09848900	2.40107800	0.65865200
H	7.54763900	2.06679500	1.61018700
H	6.48160300	0.74133500	1.13359800
C	3.32840600	0.20421800	4.99211700
H	2.68650100	0.10625300	5.87000100
H	3.32038500	-0.75532300	4.46550000
H	4.34762700	0.38247800	5.34473500

VI

C	0.41391500	-0.10323900	-0.74333900
C	-0.40998200	1.00370900	-1.42476800
H	-0.57101400	0.79776800	-2.48718600

H	0.13237900	1.95651200	-1.35754100
C	0.21449500	0.00644200	0.78053900
C	-1.29423900	0.08537400	1.07693100
C	0.90253700	-1.16161100	1.48249100
H	0.69073700	0.94752800	1.10296800
H	-1.72100000	-0.92666200	1.06355000
H	-1.49572100	0.50633900	2.06651500
H	0.86458900	-1.06114500	2.57223400
H	0.34735700	-2.08364300	1.25256500
Si	-1.99873500	1.09163500	-0.38016200
C	-2.46995800	2.85135000	0.10122100
H	-1.62793400	3.29937300	0.64351400
H	-2.59606300	3.44958800	-0.80959600
C	-3.45574600	0.23049900	-1.20912000
H	-3.12494100	-0.76239000	-1.53927500
H	-4.24145500	0.05546100	-0.46380700
C	-3.74413600	2.91015400	0.95414800
H	-3.63722700	2.31807600	1.86916200
H	-3.98895600	3.93371600	1.25127900
H	-4.60487600	2.51118000	0.40767500
C	-4.01765800	1.02524100	-2.39515500
H	-3.24550900	1.21347200	-3.14849400
H	-4.83705900	0.49566100	-2.88915200
H	-4.40155600	1.99859000	-2.07290700
C	1.89899600	-0.06102200	-1.09965200
H	0.00815500	-1.08155700	-1.04714200
H	2.05419300	-0.31520200	-2.15299200
H	2.26052300	0.96957700	-0.97467500
C	2.75864200	-0.95449900	-0.23318600
C	2.31636800	-1.39692800	1.02096400
C	4.02620300	-1.35204200	-0.67825500
C	3.21197200	-2.13958600	1.84677200
C	4.87228100	-2.16554300	0.07695900
H	4.35398100	-1.02064400	-1.66017500
C	4.46302800	-2.57998200	1.33384500
H	2.80677200	-2.63306600	2.73309900
H	5.83305600	-2.47448700	-0.31760600
H	5.08814600	-3.22355800	1.94427200
Si	4.17844300	-0.48552800	2.83781300
H	4.63478000	0.30994300	1.68355900
C	5.55136000	-1.18086700	3.90339500
H	5.58802800	-0.52531000	4.78328700
H	5.25975300	-2.16607200	4.28624100
C	2.84363100	0.31577900	3.86663800
H	3.34554200	1.19858400	4.28870400
H	2.04152800	0.70530400	3.23286400
C	6.93051800	-1.22828400	3.23383700
H	7.23910300	-0.23448100	2.90038200
H	7.68389900	-1.59627800	3.93359200
H	6.94342200	-1.88532300	2.35994500
C	2.30183000	-0.56041200	5.00347200
H	1.60241100	0.00173300	5.62554300
H	1.77071400	-1.43960000	4.62641600
H	3.10908800	-0.91672100	5.64956000

H_2

H	4.18964600	-0.97917700	0.35025900
H	3.45206700	-0.97917700	0.35025900

3aa

C	0.58948400	-0.13333900	-0.62621100
C	-0.25127400	1.00875000	-1.23061000
H	-0.37711200	0.89799500	-2.31227700
H	0.26344400	1.96506200	-1.06352000
C	0.32421000	-0.19873200	0.88949700
C	-1.19830500	-0.17777700	1.13093400
C	1.01526900	-1.41855000	1.49507700
H	0.76401900	0.71179900	1.32897000
H	-1.60180500	-1.18926900	0.98441400
H	-1.44786600	0.12083800	2.15398300
H	0.97055500	-1.38153200	2.58970200
H	0.44777900	-2.31309000	1.19874200
Si	-1.86484200	0.97293100	-0.22695200
C	-2.35887400	2.67452200	0.42355200
H	-1.53274600	3.06257300	1.03268000
H	-2.46183600	3.36627100	-0.42192400
C	-3.30773500	0.21597800	-1.17992800
H	-2.97350000	-0.73732000	-1.60849500
H	-4.11132200	-0.03444300	-0.47603000
C	-3.65500200	2.64529200	1.24361300
H	-3.57221300	1.95684600	2.09124600
H	-3.90920600	3.63101600	1.64409800
H	-4.50078600	2.30942300	0.63453100
C	-3.84053100	1.13702000	-2.28490500
H	-3.05099000	1.39940900	-2.99703200
H	-4.65249500	0.67127100	-2.85098800
H	-4.22523500	2.07356400	-1.86770400
C	2.08587400	0.00942300	-0.89510100
H	0.24312000	-1.08981400	-1.05156700
H	2.29683400	-0.10596800	-1.96453400
H	2.38143500	1.03709300	-0.63695000
C	2.94409600	-0.95259900	-0.09863600
C	2.45303000	-1.59181500	1.04838600
C	4.26710700	-1.18305300	-0.49211800
C	3.30162500	-2.43690700	1.77220600
C	5.10149500	-2.02591700	0.23135000
H	4.64195000	-0.68667500	-1.38431200
C	4.61448500	-2.65788200	1.37518700
H	2.91612400	-2.92814700	2.66269900
H	6.12411800	-2.19029100	-0.09322200
H	5.25473500	-3.31734900	1.95252700

cis-**TS$_{IV-V}$**

C	0.12866200	-1.47165600	0.19241100
C	-0.60331100	-0.73725200	-0.93872400
H	0.04210000	0.01096900	-1.41663200
H	-0.92000400	-1.43520100	-1.71840700
C	0.23991300	-0.55133600	1.41360400
C	1.10674900	0.58795000	1.32590500
C	-0.96001200	0.51767100	1.52999900
H	0.34008000	-1.08671500	2.35667200
H	1.18681500	1.15532800	0.40125800
H	1.47173700	1.06557600	2.23022600
H	-1.38233000	0.44290700	2.53156900
H	-0.66598300	1.57661200	1.40716200
Si	-2.02552900	0.12348200	-0.03689600
H	-0.48411700	-2.32804200	0.49458200
C	-2.65746200	1.70214600	-0.82648300
H	-1.80489500	2.36903900	-1.00424500
H	-3.06246000	1.45898200	-1.81602700
C	-3.42098200	-1.04568700	0.42178500

H	-3.00013000	-1.95890800	0.85839500
H	-4.02387500	-0.58111900	1.21095600
C	-3.72358400	2.40666200	0.02467800
H	-3.34072200	2.65669000	1.01958400
H	-4.05655800	3.33650900	-0.44283900
H	-4.60443900	1.77211200	0.16178200
C	-4.30225400	-1.38690800	-0.78945200
H	-3.71782900	-1.84596200	-1.59302500
H	-5.09659800	-2.08672800	-0.51845300
H	-4.77800500	-0.49045100	-1.19896200
C	1.53683700	-2.03118100	-0.16700200
H	1.45265200	-2.59445100	-1.10012500
H	1.85091700	-2.72691500	0.61720900
C	2.53549000	-0.91997700	-0.30069400
C	3.17529800	-0.41617700	0.85310000
C	2.72843300	-0.25305200	-1.51421300
C	3.99361200	0.72340500	0.77975400
H	3.12523300	-0.98053100	1.78123600
C	3.52125800	0.88922800	-1.57564100
H	2.24691000	-0.62990200	-2.41176100
C	4.14576800	1.38852500	-0.42788500
H	4.49656200	1.08034100	1.67241800
H	3.65461400	1.39793400	-2.52472500
H	4.76124900	2.27901300	-0.48815100

TS$_{I\text{-}10aa}$

C	-0.55742000	0.31923800	-0.87536600
C	0.13716000	0.79957300	0.31906600
H	-0.41985100	1.57330500	0.85272100
H	0.36644100	-0.02384300	1.00033300
C	-1.41126500	1.21803600	-1.66103500
C	-2.34776000	2.20840300	-0.99933700
C	-1.12510900	2.72212300	-1.65294600
H	-1.73952700	0.80887800	-2.61116900
H	-2.38291800	2.20375200	0.08619200
H	-3.30314200	2.34960500	-1.49146200
H	-1.20358900	3.21019900	-2.61639100
H	-0.31777300	3.07380300	-1.02155500
Si	1.92562300	1.49906200	-0.09438900
C	1.99496200	3.34397400	0.29028900
H	3.06054000	3.56028200	0.44378900
H	1.70107600	3.93245600	-0.58492400
C	3.13069200	0.53510400	0.97603800
H	3.06942500	0.93150300	1.99756300
H	2.81208200	-0.51251500	1.02615200
C	1.19833600	3.78190000	1.52620200
H	1.42071300	3.15674500	2.39711600
H	1.42860400	4.81510200	1.79657300
H	0.11884800	3.72970100	1.34905700
C	4.57085700	0.62262000	0.45458300
H	4.92562500	1.65720700	0.41162200
H	5.25775200	0.06671800	1.09819800
H	4.64601900	0.20227300	-0.55303700
Si	-2.86630300	-1.33234000	0.49222800
C	-2.46924800	-1.29104400	2.32240400
H	-3.08537100	-2.06013200	2.80413400
H	-1.42909800	-1.60083200	2.47745300
C	-2.73085700	0.07786100	2.96545100
H	-3.77853100	0.37263100	2.86052600
H	-2.49697900	0.05936300	4.03235400
H	-2.11870800	0.86229000	2.50933700

C	-4.46196500	-0.49820200	-0.01422800
H	-5.26684600	-0.98167700	0.55424000
H	-4.44215200	0.54495100	0.32030900
C	-4.74378600	-0.58723800	-1.51949500
H	-5.69395400	-0.10939300	-1.76950500
H	-3.96128600	-0.09220000	-2.10217400
H	-4.79565700	-1.62746200	-1.85152900
H	-1.72204000	-0.51987500	-0.18903500
H	-2.69763100	-2.66788300	-0.13074500
H	2.17412200	1.26970000	-1.54055300
C	0.04483400	-0.81521500	-1.68955200
H	0.51173200	-0.30468100	-2.54354100
H	-0.77009500	-1.40752400	-2.11970400
C	1.07566500	-1.68553900	-1.01107600
C	0.75953300	-2.46159500	0.10891900
C	2.37838000	-1.73251400	-1.51378100
C	1.72968300	-3.24443800	0.72751700
H	-0.25352500	-2.46494700	0.50242400
C	3.34947800	-2.52506600	-0.90456100
H	2.63593800	-1.14309500	-2.38972100
C	3.02943900	-3.27555800	0.22294000
H	1.46886600	-3.83601100	1.59864800
H	4.35580700	-2.54952000	-1.30955900
H	3.78494000	-3.88751900	0.70391300

10aa

C	0.57835900	0.37348500	-1.06628700
C	1.00881500	1.84231800	-0.86470500
H	2.03035800	1.97327100	-1.24136900
H	0.38691200	2.47125200	-1.51304500
C	1.65973000	-0.64524100	-0.72881200
C	3.07802600	-0.51372200	-1.19664200
C	2.75318700	-0.37698300	0.26680000
H	1.28310200	-1.66648800	-0.72135400
H	3.36297500	0.38618700	-1.73235200
H	3.58843300	-1.41099000	-1.52792200
H	3.04629800	-1.17494700	0.93934400
H	2.82796300	0.61802300	0.69291100
Si	0.88033800	2.65187100	0.85570900
C	2.55280100	3.33540100	1.43249100
H	2.34603500	4.00227100	2.27998200
H	3.17859800	2.53183600	1.83706600
C	-0.34639500	4.08783100	0.76580100
H	0.15572000	4.93676200	0.28226800
H	-1.17545700	3.80328700	0.10668400
C	3.32093000	4.09977400	0.34696600
H	2.70466600	4.88303200	-0.10737500
H	4.21814200	4.58085400	0.74727700
H	3.64152100	3.42988000	-0.45697200
C	-0.88822600	4.50628000	2.13646100
H	-0.08167600	4.79360700	2.81980300
H	-1.57363200	5.35638900	2.06290200
H	-1.43498800	3.68000700	2.60224600
H	0.41459500	1.68568800	1.89263900
C	-0.69646300	-0.01218600	-0.28551100
H	-0.42758600	-0.18640700	0.76218500
H	-1.05246000	-0.97517500	-0.67480000
C	-1.82086400	0.99627600	-0.33733200
C	-2.22517100	1.57439000	-1.54636500
C	-2.48412300	1.37742200	0.83244700
C	-3.25273900	2.51258600	-1.58200900

H	-1.72513100	1.29054300	-2.46930300
C	-3.51849700	2.31139900	0.80210600
H	-2.17717100	0.94206100	1.78036600
C	-3.90288000	2.88650100	-0.40578500
H	-3.54766000	2.95359600	-2.52913800
H	-4.01607700	2.59536000	1.72441800
H	-4.70228300	3.61990100	-0.43333800
H	0.35985800	0.24724900	-2.13676800

trans-**TS**$_{\mathbf{IV\text{-}VII}}$

C	0.35610600	-0.28411900	-0.30067400
C	-0.58416500	0.94216800	-0.52593100
H	-0.82040100	0.97131100	-1.59516200
H	-0.03552200	1.86290400	-0.29487900
C	0.59882200	-0.34140400	1.15937700
C	-1.39731100	0.23440500	2.23257100
C	-0.33019100	-0.94394800	2.09334700
H	1.33925900	0.35251500	1.56239300
H	-2.16290400	-0.17617600	2.89866200
H	-0.93771900	1.07701500	2.75922600
H	0.11333200	-1.14898000	3.06749300
H	-0.82573500	-1.82622800	1.68391100
Si	-2.13723200	0.80909600	0.56685100
C	-2.99248900	2.46429100	0.78078000
H	-2.27591400	3.17807600	1.20375800
H	-3.25801100	2.85041000	-0.21059400
C	-3.30926700	-0.49772100	-0.10973000
H	-2.75470500	-1.41914900	-0.32648600
H	-4.02836800	-0.75804000	0.67660600
C	-4.24368000	2.37247500	1.66666400
H	-3.99706900	2.01229400	2.67035900
H	-4.72630400	3.34672600	1.77638800
H	-4.98203800	1.68473100	1.24258200
C	-4.05112400	-0.02447700	-1.36854600
H	-3.35419800	0.24615800	-2.16817400
H	-4.71136900	-0.80295300	-1.75876400
H	-4.66663800	0.85550100	-1.15823200
C	1.72081300	-0.19976200	-1.00318700
H	-0.18653000	-1.18726100	-0.60177200
H	1.64916000	-0.58780200	-2.02305600
H	2.03163200	0.84723900	-1.07377600
C	2.72564300	-0.96426700	-0.16131300
C	2.30951200	-2.09720700	0.56364100
C	4.02448900	-0.48680200	0.02790100
C	3.17349900	-2.71668200	1.47726400
H	1.35318700	-2.56793100	0.35309400
C	4.88272300	-1.11431300	0.92342600
H	4.35377600	0.39436500	-0.51439900
C	4.45595000	-2.22358000	1.66119700
H	2.83832700	-3.59331700	2.02103900
H	5.88637000	-0.72704200	1.06442800
H	5.12923100	-2.69875000	2.36588800

trans-**VII**

C	0.41349000	-0.10811000	-0.29797700
C	-0.54445800	1.07599100	-0.42865000
H	-0.81111400	1.23689900	-1.47961400
H	-0.04264900	1.99082700	-0.08548700
C	0.82778000	-0.40366100	1.16159000
C	-1.40028100	0.07306600	2.26194600

C	-0.28620700	-0.96708900	2.03811100
H	1.21459700	0.52148100	1.61341500
H	-2.20464400	-0.37027900	2.85885400
H	-0.99945300	0.90619300	2.85455200
H	0.12583900	-1.28976000	3.00171300
H	-0.69536300	-1.86512900	1.55489000
Si	-2.08854800	0.76145000	0.63396600
C	-3.06520200	2.34476800	0.92181400
H	-2.41986900	3.06466300	1.43967100
H	-3.30461700	2.79557300	-0.04915200
C	-3.18317300	-0.53261400	-0.19938400
H	-2.58405700	-1.42196300	-0.43323000
H	-3.93732100	-0.86513300	0.52499600
C	-4.35094200	2.10919200	1.72640600
H	-4.13239700	1.67038700	2.70536100
H	-4.89845400	3.03996200	1.89808200
H	-5.02500900	1.42258900	1.20385500
C	-3.86880200	-0.01149300	-1.46952100
H	-3.13607000	0.32854400	-2.20871100
H	-4.48180800	-0.78156600	-1.94599100
H	-4.52275800	0.83745500	-1.24553000
C	1.75778500	0.02523100	-1.04213500
H	-0.08333400	-1.01652200	-0.67347800
H	1.72973300	-0.22568700	-2.10617300
H	2.12540000	1.06079800	-0.97109400
C	2.71142400	-0.81529100	-0.26163500
C	2.03785100	-1.34703300	0.93116300
C	4.05664600	-1.03600700	-0.47351100
C	2.88323500	-1.88851100	1.99750200
H	1.57303900	-2.28371100	0.51904100
C	4.78637800	-1.64850600	0.54719300
H	4.55468800	-0.68265400	-1.36884100
C	4.21944600	-2.05720900	1.78174600
H	2.41370300	-2.22478200	2.91726900
H	5.85338000	-1.79423000	0.40350900
H	4.85736800	-2.49794500	2.53773000

trans-**TS**$_{\mathbf{VII\text{-}11aa}}$

C	-0.69228600	-0.74474700	0.93939000
C	-2.14238200	-0.76779500	1.41727500
H	-2.26199700	-0.15320500	2.31688800
H	-2.41689400	-1.79440900	1.69596600
C	-0.44906200	-1.58797600	-0.33582200
C	-2.61231800	-1.04303000	-1.53479900
C	-1.07305000	-1.01908200	-1.60842200
H	-0.87443800	-2.59029100	-0.15997700
H	-3.03568300	-0.58956700	-2.43776800
H	-2.95195600	-2.08723400	-1.52424500
H	-0.72903500	-1.59111000	-2.47834200
H	-0.71441600	0.00980900	-1.75084000
Si	-3.27266500	-0.16800000	0.01347300
C	-5.07213700	-0.60630100	0.35575400
H	-5.16071100	-1.69914800	0.38920800
H	-5.33688500	-0.25158400	1.35963300
C	-3.08434800	1.69687500	-0.21756600
H	-2.05177100	1.91927200	-0.51463800
H	-3.70574900	1.99397700	-1.07201800
C	-6.04381600	-0.02580400	-0.68050300
H	-5.79023400	-0.35457900	-1.69372000
H	-7.07537400	-0.33121700	-0.48460000
H	-6.01830000	1.06844500	-0.67557700

C	-3.46350700	2.51656600	1.02168700
H	-2.82252400	2.26764700	1.87380000
H	-3.37008900	3.59176500	0.84374600
H	-4.49781100	2.32228500	1.32457800
C	0.37597200	-1.26073600	1.92619900
H	-0.41898100	0.29603900	0.68438500
H	0.63800100	-0.54898000	2.71287900
H	0.02012500	-2.18094200	2.40987400
C	1.52653800	-1.61486000	1.02153700
C	1.05647300	-1.75557400	-0.31226500
C	2.88351100	-1.77581200	1.30910100
C	1.93872900	-2.15258300	-1.34104800
C	3.75237100	-2.08104700	0.26576200
H	3.25325600	-1.64706700	2.32131100
C	3.28577300	-2.27859800	-1.04699900
H	1.57208400	-2.29682300	-2.35209600
H	4.81508100	-2.17600800	0.46675400
H	3.99134300	-2.53254800	-1.83007500
H	1.69662900	-0.35868000	-0.24772900
Si	2.71112700	1.60425300	0.38231000
H	1.95393700	1.32565500	1.62229900
H	2.23168500	0.40500600	-0.56553700
C	2.05700400	3.08190600	-0.54757400
H	2.35931600	3.96909200	0.02320100
H	2.56316600	3.15062000	-1.51582200
C	4.56180100	1.39836000	0.50831400
H	4.93571800	2.30861100	0.99471300
H	4.78003300	0.57140100	1.19074600
C	0.53376700	3.04885100	-0.72393800
H	0.02163000	2.98070900	0.24133200
H	0.17678800	3.95107500	-1.22558700
H	0.22629200	2.19182600	-1.33213400
C	5.25374600	1.19376100	-0.84561400
H	6.33583800	1.12156700	-0.71742300
H	4.91124600	0.27323600	-1.32762200
H	5.05553800	2.02358600	-1.52932100

11aa

C	0.49514500	0.07062200	-0.22476400
C	-0.54357200	1.17946000	-0.40394700
H	-0.80244600	1.30322200	-1.46288000
H	-0.11881400	2.13612800	-0.06884600
C	0.90768600	-0.16115300	1.24940500
C	-1.39409800	0.12008600	2.28334000
C	-0.17966100	-0.81208100	2.10316900
H	1.14009600	0.82535700	1.68959500
H	-2.18306400	-0.38806900	2.84992800
H	-1.09025500	0.98630400	2.88743600
H	0.23123700	-1.08457800	3.08320800
H	-0.48566600	-1.75164100	1.62294500
Si	-2.07389100	0.75853500	0.63279000
C	-3.17911600	2.26551700	0.89701900
H	-2.60206600	3.03170400	1.42947400
H	-3.42888300	2.69955800	-0.07934400
C	-3.07227300	-0.60436400	-0.21798300
H	-2.41162500	-1.45424800	-0.43082800
H	-3.82130500	-0.97961200	0.49106400
C	-4.46250100	1.93660000	1.67108400
H	-4.23477400	1.50929200	2.65323300
H	-5.08147400	2.82369800	1.83424700
H	-5.07283300	1.20452300	1.13177100

C	-3.75905100	-0.13477400	-1.50687000
H	-3.03069900	0.24516900	-2.23085400
H	-4.31364800	-0.94245700	-1.99354100
H	-4.46793000	0.67508100	-1.30439500
C	1.84091700	0.24263400	-0.95711200
H	0.06708200	-0.88295300	-0.57398300
H	1.78516600	0.00392200	-2.02314300
H	2.18221500	1.28422000	-0.86884600
C	2.75234700	-0.67669700	-0.17550800
C	2.20807800	-0.91983400	1.09315500
C	3.95985700	-1.25349200	-0.54669900
C	2.86720000	-1.74348300	1.99672500
C	4.62158500	-2.08511700	0.36057700
H	4.38116600	-1.07059300	-1.53142100
C	4.07939300	-2.32871100	1.62132700
H	2.44836800	-1.93833100	2.98031000
H	5.56137000	-2.55010100	0.07945500
H	4.60038200	-2.98165100	2.31461600

cis-$\mathbf{TS}_{\mathbf{II\text{-}IV}}$

C	0.50144500	2.23292200	-0.48035900
C	1.94084000	2.66448700	-0.23343600
C	0.58714700	0.75766800	-0.91295100
C	2.38910300	3.72520600	0.68038200
C	2.63568100	2.28098900	1.10745300
H	2.58090200	2.52315400	-1.10313600
C	-0.43055300	2.39862800	0.73196800
Si	1.97407700	-0.00993400	0.08277400
H	0.86086400	0.66927100	-1.97085000
H	-0.36058100	0.22873500	-0.77032100
H	3.25967400	4.30124500	0.39325900
H	1.65087400	4.25964900	1.26600700
H	3.67149300	1.96165700	1.12733700
H	2.03807000	1.99743100	1.96403800
C	3.62669600	-0.33066800	-0.73125200
C	1.66217900	-0.76053700	1.76662400
C	3.63979700	-0.13620000	-2.25395800
H	4.37883300	0.30780300	-0.25594400
H	3.91229800	-1.35756000	-0.47289500
C	2.73026000	-1.77642600	2.19510300
H	1.56866500	0.02822800	2.51792800
H	0.67230300	-1.23067200	1.71237900
H	3.41934400	0.89724300	-2.53382800
H	2.90736400	-0.77877300	-2.75050300
H	4.62405000	-0.38325900	-2.65623100
H	3.71861100	-1.31356700	2.25763300
H	2.79434900	-2.61303900	1.49364500
H	2.48971200	-2.18436600	3.17895100
H	0.10151400	2.84171400	-1.29959400
C	-2.79426400	-2.37632700	-0.80168700
C	-3.03806900	-2.99981300	0.42030500
C	-1.97733200	-3.46358000	1.19532400
C	-0.66392400	-3.30641200	0.75710000
C	-0.45248600	-2.67280800	-0.46107400
C	-1.48932000	-2.20545100	-1.25787700
H	-3.61871300	-2.01508100	-1.40665000
H	-4.05751800	-3.12913000	0.76747000
H	-2.16559300	-3.95545100	2.14313800
H	0.17284600	-3.67480500	1.34130700
H	-1.28319600	-1.72727600	-2.20887000
Cl	1.20740400	-2.45980500	-1.02371000

H	-0.12607800	1.71921100	1.53855700
H	-0.34693400	3.41887500	1.12031900
C	-1.87436300	2.12489000	0.37812200
C	-2.49114400	0.91522400	0.70604400
C	-2.61482000	3.09171300	-0.31025000
C	-3.82282000	0.68183200	0.36428700
H	-1.93139600	0.14881900	1.24053300
C	-3.94128500	2.85926700	-0.65792300
H	-2.14686000	4.03957100	-0.56535800
C	-4.55067300	1.65212300	-0.31781200
H	-4.28958500	-0.25908500	0.63759200
H	-4.50273100	3.62260400	-1.18682100
H	-5.58811100	1.47225600	-0.57964700

cis-**IV**

C	-0.08168800	1.37853900	-0.02036900
C	0.54980100	0.48763200	-1.09906200
H	-0.09650000	-0.36364700	-1.34703900
H	0.74664000	1.03798100	-2.02225600
C	0.07653400	0.68997000	1.34198400
C	-0.61802600	-0.48017200	1.78669500
C	0.94575500	-0.68742800	1.44502600
H	0.44163300	1.32517200	2.14568600
H	-1.26259700	-1.01572800	1.09974400
H	-0.82614200	-0.61099300	2.84142800
H	1.55869600	-0.80212600	2.33166300
H	0.81046500	-1.60606700	0.86626100
Si	2.06288800	-0.14270000	-0.19471600
H	0.52367000	2.28902800	0.03901900
C	2.88230600	-1.69837900	-0.81908100
H	2.10969700	-2.42364000	-1.10003300
H	3.39048300	-1.42879900	-1.75391400
C	3.27951100	1.14129400	0.40296500
H	2.74863600	2.02132800	0.78040000
H	3.83083000	0.72439900	1.25358100
C	3.88351000	-2.31543300	0.16826600
H	3.39785900	-2.61293300	1.10282400
H	4.34887300	-3.20704400	-0.25695700
H	4.68301200	-1.61178100	0.41728500
C	4.25438400	1.53787000	-0.71929700
H	3.72614300	1.94739200	-1.58532600
H	4.95465100	2.29951900	-0.37004600
H	4.84258800	0.68067600	-1.05983200
C	-1.52614000	1.86723300	-0.29189700
H	-1.49783200	2.39259700	-1.25220300
H	-1.78262500	2.61201100	0.46860100
C	-2.58740300	0.79644800	-0.31943000
C	-3.40941500	0.58930900	0.79331300
C	-2.75971100	-0.02321800	-1.43977000
C	-4.35671900	-0.43097700	0.80233500
H	-3.29868900	1.23263500	1.66297100
C	-3.70382600	-1.04802800	-1.43330100
H	-2.16017700	0.15206300	-2.32932200
C	-4.49958700	-1.25901900	-0.30941100
H	-4.98475400	-0.57726500	1.67513700
H	-3.82425600	-1.67555300	-2.31039100
H	-5.23645400	-2.05527700	-0.30505600

cis-**TS**$_{\mathbf{IV\text{-}IV'}}$

C	0.20325200	-1.54116700	0.20708700

C	-0.49079000	-0.63691200	-0.82190700
H	0.17589100	0.17270200	-1.13999500
H	-0.72182600	-1.22092500	-1.71738500
C	0.26705000	-0.91384300	1.57125100
C	0.30656300	0.46202400	1.79277800
C	-1.34201800	0.01596100	1.79674100
H	0.42013400	-1.57226500	2.42445700
H	0.43998700	1.14008600	0.95426100
H	0.60731000	0.83559500	2.76479800
H	-1.62668000	-0.89638100	2.34117000
H	-1.61366600	0.79787800	2.51457600
Si	-2.03913100	0.07884600	0.00409700
H	-0.35758500	-2.47666400	0.31044100
C	-2.43946400	1.84110000	-0.49186500
H	-1.57356900	2.48297900	-0.28994700
H	-2.57737100	1.86045400	-1.57974600
C	-3.53263500	-1.04787800	-0.10757100
H	-3.23461000	-2.06627100	0.16968600
H	-4.27156500	-0.73246400	0.63869300
C	-3.69108000	2.38713000	0.21024900
H	-3.57720900	2.37994400	1.29887500
H	-3.89773100	3.41659000	-0.09160100
H	-4.57461000	1.78841600	-0.03091900
C	-4.15614300	-1.03530600	-1.51143000
H	-3.43558200	-1.35334000	-2.27113200
H	-5.01540900	-1.70742000	-1.57015400
H	-4.50131600	-0.03320100	-1.78409500
C	1.66395500	-1.94612100	-0.17598300
H	1.58582500	-2.48287300	-1.12570900
H	2.04526700	-2.65671000	0.56466700
C	2.60648400	-0.77558700	-0.30645700
C	3.31324600	-0.30578900	0.80587300
C	2.77429700	-0.12365500	-1.53163800
C	4.15677200	0.79697400	0.70099000
H	3.21287500	-0.81782100	1.76104700
C	3.61714600	0.98012100	-1.64045200
H	2.24697000	-0.49141000	-2.40821100
C	4.30732200	1.44471700	-0.52350500
H	4.70231900	1.14459000	1.57209300
H	3.73937200	1.47218300	-2.59971000
H	4.96686400	2.30186600	-0.60848500

cis-**IV’**

C	0.91326600	-0.07210100	1.39011300
C	-0.05320500	-1.11083000	0.78208000
H	0.09908900	-1.18284300	-0.30063900
H	0.18649300	-2.09576900	1.19353400
C	0.39863700	1.33749900	1.22807100
C	-0.46281800	1.67974500	0.16502500
C	-1.43379700	1.23352100	1.40444200
H	0.80858800	2.12831900	1.85319900
H	-0.55557600	0.99053300	-0.66977300
H	-0.59870900	2.72617100	-0.08055100
H	-0.95112400	1.19552500	2.41002200
H	-2.09986600	2.08781100	1.52774800
Si	-1.86036000	-0.62653300	1.13121600
H	1.02308100	-0.26115900	2.46345500
C	-3.01720000	-0.86103900	-0.32218400
H	-2.62356700	-0.32860200	-1.19597800
H	-3.00692800	-1.92430300	-0.59097800
C	-2.53862400	-1.35223500	2.71947000

H	-1.80008900	-1.20913800	3.51770400
H	-3.43003900	-0.78981000	3.02077700
C	-4.45029700	-0.40392500	-0.01319600
H	-4.48202600	0.65625600	0.25614800
H	-5.10495300	-0.54537700	-0.87622800
H	-4.87700900	-0.96887600	0.82107100
C	-2.87739000	-2.84327600	2.56716000
H	-1.99847400	-3.42503600	2.27267600
H	-3.25058800	-3.26180300	3.50465400
H	-3.64742900	-3.00036600	1.80561600
C	2.35061000	-0.12064600	0.78659800
H	2.70890000	-1.14300200	0.93810200
H	3.00153100	0.53893500	1.36993300
C	2.41586700	0.25272100	-0.67451600
C	2.57739100	1.58979700	-1.05440600
C	2.28897700	-0.71685900	-1.67318900
C	2.59096500	1.95282900	-2.39814400
H	2.70627600	2.35300900	-0.28905200
C	2.30296800	-0.35743100	-3.01953900
H	2.19018500	-1.76252000	-1.39348000
C	2.44919500	0.97837400	-3.38447200
H	2.72152900	2.99370400	-2.67537100
H	2.20669700	-1.12289300	-3.78245800
H	2.46330200	1.25850500	-4.43244800

cis-**TS$_{IV'-VIII}$**

C	0.79344200	-0.34567300	1.42135000
C	-0.19403300	-1.09357200	0.55329200
H	-0.03210000	-0.86834200	-0.50713100
H	-0.06993400	-2.17161500	0.67795500
C	0.66131300	1.05877700	1.52738100
C	-0.18006900	1.81673200	0.60458800
C	-1.65229000	1.42601100	1.02041900
H	1.17980400	1.56927900	2.33812900
H	-0.01945800	1.46836600	-0.42326100
H	-0.00577300	2.89057900	0.65972900
H	-1.87388500	1.84265900	2.00653000
H	-2.30561300	1.93686900	0.30654400
Si	-1.95007200	-0.46410400	0.97254400
H	1.05021300	-0.82867800	2.36428500
C	-3.16712500	-0.93742300	-0.37512900
H	-2.83400900	-0.49352600	-1.32111700
H	-3.12660900	-2.02406400	-0.51722800
C	-2.50361000	-1.08036000	2.65987500
H	-1.72972800	-0.83919500	3.39966200
H	-3.39150600	-0.51058100	2.96055100
C	-4.60286500	-0.49855000	-0.05318400
H	-4.66854200	0.58518900	0.08746100
H	-5.29251500	-0.76759500	-0.85709400
H	-4.96615400	-0.97083000	0.86492300
C	-2.80831600	-2.58433400	2.67653600
H	-1.93162300	-3.17394500	2.39030000
H	-3.11945900	-2.91906900	3.66930800
H	-3.61273700	-2.83328000	1.97742900
C	2.34543900	-0.16171700	0.71450600
H	2.64101000	-1.20657600	0.83541300
H	2.98321600	0.44260600	1.36039000
C	2.39100200	0.26485900	-0.71396600
C	2.64033700	1.60028700	-1.05449600
C	2.22795300	-0.67898700	-1.73401400
C	2.71966800	1.98434700	-2.38841500

H	2.78088300	2.34075100	-0.26961100
C	2.30281100	-0.29387200	-3.06956000
H	2.05498300	-1.72072400	-1.47764100
C	2.54699400	1.03743600	-3.39738400
H	2.91929200	3.02000000	-2.64095700
H	2.17809100	-1.03462400	-3.85181300
H	2.60850100	1.33734800	-4.43802400

VIII

C	-0.24408700	-1.09429300	-0.65965600
C	-0.30293600	0.21149700	-1.04348300
H	0.20210500	0.98917700	-0.47182300
H	-0.66462700	0.48014900	-2.03283900
C	0.37252200	-1.57988600	0.62087900
C	0.14013500	-0.58737100	1.76541700
C	-1.36264300	-0.28628500	1.91460900
H	-0.11336100	-2.52847900	0.87651000
H	0.69469200	0.33874300	1.58268400
H	0.54350200	-1.01048200	2.69024900
H	-1.88126700	-1.15982800	2.32469200
H	-1.52990800	0.54511100	2.60589200
Si	-2.12455100	0.11078200	0.24630900
H	-0.64427300	-1.84457900	-1.34227300
C	-2.56882300	1.90214200	-0.06917300
H	-1.77516900	2.54240100	0.32853200
H	-2.63707400	2.09678200	-1.14361800
C	-3.38137800	-1.09664000	-0.42037700
H	-3.02970400	-2.12318300	-0.27153900
H	-4.25841200	-0.98750000	0.23191900
C	-3.91126600	2.20957000	0.62046900
H	-3.87197100	2.01005000	1.69549900
H	-4.16602200	3.26368100	0.49137900
H	-4.72689800	1.61768900	0.19571000
C	-3.77569900	-0.84456500	-1.88167500
H	-2.92283900	-0.96203400	-2.55733900
H	-4.54549000	-1.54991400	-2.20076600
H	-4.17283300	0.16519400	-2.01946800
C	1.87954900	-1.90512800	0.37398000
H	1.95229300	-2.65818000	-0.41650900
H	2.24614200	-2.36960700	1.29450200
C	2.71747200	-0.70046200	0.02733900
C	3.30666200	0.07180000	1.03383000
C	2.90583500	-0.32050900	-1.30554600
C	4.05443600	1.20307600	0.71787500
H	3.18414800	-0.22316500	2.07316700
C	3.65475300	0.80937300	-1.62567000
H	2.47004000	-0.92229000	-2.09977500
C	4.22761800	1.57593100	-0.61344900
H	4.50784400	1.78864600	1.51092400
H	3.79600900	1.08654400	-2.66522800
H	4.81388800	2.45462400	-0.86103000

cis-**TS**$_{\mathbf{VIII\text{-}12aa}}$

C	0.61120400	0.58361100	0.45324700
C	1.74952100	0.97654400	-0.27594800
H	1.66398500	0.92689900	-1.36505900
H	2.28459700	1.85197400	0.08599400
C	-0.20514000	-0.63433400	0.11799800
C	0.31009000	-1.37321500	-1.11930900
C	1.73060600	-1.93800400	-0.90400400

H	-0.03948300	-1.28071200	0.99926500
H	0.28800700	-0.69959400	-1.98488500
H	-0.38408500	-2.18643000	-1.34748900
H	1.67292200	-2.82521200	-0.26373200
H	2.15293400	-2.26757200	-1.85852100
Si	2.88670700	-0.68502400	-0.09555600
H	0.54037100	0.92351700	1.48787100
C	4.42959700	-0.25841200	-1.06855700
H	4.14791600	-0.07553200	-2.11132300
H	4.85460800	0.67642900	-0.68699300
C	3.24609200	-1.02344500	1.71155400
H	2.30995300	-1.23209300	2.24346400
H	3.81937700	-1.95876800	1.75045300
C	5.46278600	-1.39342300	-0.97915500
H	5.05323600	-2.34005000	-1.34499500
H	6.34549300	-1.16280000	-1.58014000
H	5.79608400	-1.55012500	0.05096400
C	4.02440000	0.10497700	2.40100200
H	3.46686300	1.04694300	2.38213800
H	4.22658600	-0.13630400	3.44695500
H	4.98654700	0.28142100	1.91094000
C	-1.73266800	-0.37212100	0.09282400
H	-1.96606300	0.20543900	-0.80916800
H	-1.99901400	0.24709000	0.95668500
C	-2.52954300	-1.65356700	0.11315800
C	-2.64329500	-2.39104100	1.29549600
C	-3.14939700	-2.13132100	-1.04285000
C	-3.36184000	-3.58194500	1.32167100
H	-2.17149400	-2.02509600	2.20479700
C	-3.87050500	-3.32378100	-1.01993000
H	-3.06774200	-1.56388900	-1.96657100
C	-3.97750700	-4.05127500	0.16203300
H	-3.44630900	-4.14159100	2.24749600
H	-4.34922400	-3.68177000	-1.92548000
H	-4.54015600	-4.97869800	0.18201000
Si	-1.21054300	3.21445100	-0.22341800
H	-0.02277300	4.08749700	-0.41325700
C	-2.25910900	3.74208400	1.23864100
H	-3.01627200	2.97077100	1.42037400
H	-1.63008000	3.78246800	2.13420600
C	-2.16608600	3.01206200	-1.82560700
H	-3.05404300	2.39863200	-1.63191100
H	-2.53917400	4.00284000	-2.11243100
C	-2.93347300	5.10123500	0.99423600
H	-3.60673700	5.06182600	0.13322800
H	-3.52415600	5.40762800	1.86107300
H	-2.19421600	5.88478300	0.80359300
C	-1.32981700	2.41587000	-2.96466700
H	-1.00448500	1.39667700	-2.73056100
H	-1.90135600	2.36930900	-3.89489800
H	-0.43307200	3.01287200	-3.15506600
H	-0.58180700	1.85825600	0.09659200

12aa

C	-0.14959700	-0.15637400	-1.11949200
C	1.28522400	-0.68826800	-1.29267800
C	-0.84229800	-0.62376800	0.17114200
C	1.26329900	-0.60156800	1.66490100
C	-0.16892200	-0.07863900	1.44038900
H	-0.79357200	-1.72188200	0.20495000
H	1.67727300	-0.17845800	2.58561200

H	1.23038400	-1.68865300	1.80826400
H	-0.78933500	-0.32992900	2.30583800
H	-0.16086300	1.01833200	1.37670000
Si	2.36016200	-0.21525900	0.18187100
C	3.97451700	-1.18757700	0.22226000
H	3.73567500	-2.25334900	0.30530300
H	4.48904400	-1.06544500	-0.73728100
C	2.75366800	1.63201700	0.13588100
H	1.81789800	2.19553500	0.05311400
H	3.19317800	1.91937600	1.09773700
C	4.89831000	-0.76093800	1.37258700
H	4.40439200	-0.87826600	2.34113700
H	5.81671200	-1.35187700	1.39791500
H	5.18552100	0.29002900	1.27831000
C	3.70115000	2.01606800	-1.00972600
H	3.27989900	1.74323700	-1.98126000
H	3.90266900	3.08961800	-1.02952400
H	4.66222300	1.50266200	-0.91573800
C	-2.32795000	-0.21743500	0.12651600
H	-0.14315100	0.94188200	-1.12687100
H	-0.76205400	-0.46262800	-1.97448400
H	1.71345700	-0.32296500	-2.23149600
H	1.25771100	-1.78209200	-1.36966200
H	-2.39364500	0.87629000	0.13198800
H	-2.75381300	-0.55758000	-0.82301200
C	-3.14755400	-0.78011200	1.26143600
C	-3.58977500	0.02526700	2.31154100
C	-3.46149500	-2.14161000	1.29165000
C	-4.32353300	-0.51186400	3.36603600
H	-3.35407300	1.08472400	2.30243100
C	-4.19357600	-2.68363100	2.34185000
H	-3.13078200	-2.78052300	0.47836300
C	-4.62722100	-1.86887600	3.38474700
H	-4.65823900	0.13069400	4.17216100
H	-4.42995200	-3.74141100	2.34562300
H	-5.19924100	-2.28904400	4.20339000

TS_{V-IX}

C	0.86736800	-0.07861800	-0.38427500
C	0.13509600	1.13568300	-0.98104000
H	0.02482400	1.05367900	-2.06635500
H	0.71521300	2.04693300	-0.78147600
C	0.58355000	-0.12180100	1.12862100
C	-0.93800500	-0.02119100	1.34243700
C	1.19721400	-1.36436900	1.76924000
H	1.05235000	0.77481500	1.56539800
H	-1.39545300	-1.00547400	1.17559800
H	-1.18755400	0.28272700	2.36350400
H	1.13992600	-1.31062200	2.86194800
H	0.61480100	-2.24277700	1.46570200
Si	-1.50597400	1.19057200	-0.01429600
C	-1.90054500	2.90552700	0.65920900
H	-1.06440200	3.22959100	1.29158500
H	-1.94274800	3.61711000	-0.17464200
C	-2.96115500	0.53202900	-1.01317400
H	-2.67112800	-0.43109100	-1.45162400
H	-3.79412800	0.31640800	-0.33265100
C	-3.21340900	2.94052100	1.45271000
H	-3.19279400	2.23067700	2.28634100
H	-3.41179900	3.93200800	1.86919400
H	-4.06535000	2.67300200	0.81924000

C	-3.41233400	1.50329400	-2.11193500
H	-2.59202200	1.73366600	-2.79978800
H	-4.23426800	1.09360500	-2.70557400
H	-3.75503600	2.45154400	-1.68521600
C	2.37508700	-0.06821800	-0.64600100
H	0.44524300	-0.99714700	-0.82212400
H	2.59981400	-0.09984700	-1.71628200
H	2.78688800	0.88465000	-0.27447800
C	3.10144600	-1.18366900	0.04960600
C	2.65098500	-1.61864000	1.34799300
C	4.26374600	-1.73375100	-0.48264100
C	3.34713800	-2.71103100	1.97369600
C	4.94085000	-2.74938800	0.18664100
H	4.62996400	-1.37900700	-1.44055400
C	4.48362000	-3.24772200	1.41773800
H	2.97078100	-3.07603200	2.92626900
H	5.83958300	-3.16795000	-0.25548500
H	5.01907100	-4.04764700	1.91557500
H	3.28168200	-0.68018700	1.96779300
C	4.04967500	0.08175200	3.81303200
C	3.90638200	0.74206000	2.63271400
H	4.71763300	0.80748500	1.91104800
H	3.06810300	1.41648800	2.48524100
C	5.23469700	-0.71527200	4.16175900
C	6.27571200	-1.09950600	3.13569000
C	6.59889400	-0.02813100	4.10888500
H	5.09504800	-1.39578800	4.99586100
H	6.09006600	-0.82771900	2.10154700
H	6.73142300	-2.07388400	3.27042500
H	7.27938600	-0.24345500	4.92368000
H	6.59365300	0.99929500	3.76240500
C	2.96967200	0.13400200	4.86498700
H	2.65548400	-0.88869400	5.10702300
H	2.09797600	0.67451000	4.48228200
C	3.50210900	0.80063600	6.12117500
C	3.87313700	2.14782500	6.08601300
C	3.65491800	0.08358000	7.30733100
C	4.38071100	2.76893200	7.22208400
H	3.76179400	2.70930700	5.16132900
C	4.16390500	0.70429300	8.44700100
H	3.36868500	-0.96419300	7.34187100
C	4.52750200	2.04705700	8.40636700
H	4.66180500	3.81634600	7.18511600
H	4.27439000	0.13718400	9.36549300
H	4.92315200	2.53133700	9.29294500

IX

C	2.16495400	-5.94087400	3.99065100
C	2.01261000	-4.66695400	3.24993500
H	1.11863400	-4.60430900	2.63170900
H	2.05987800	-3.81752100	3.93519900
C	1.48541900	-7.11009100	3.65286900
C	-0.00385300	-6.98420800	3.12158400
C	1.01546400	-7.30731400	2.15916000
H	1.70546400	-8.01193800	4.20806100
H	-0.42544400	-5.98764500	3.15917700
H	-0.61358900	-7.77599800	3.53976400
H	1.15249400	-8.33761900	1.85349600
H	1.36318600	-6.55206300	1.46483000
C	3.11987100	-5.91851500	5.13809400
H	2.60962000	-5.31134500	5.90382500

H	3.98300300	-5.30935400	4.83962900
C	3.54110900	-7.25286500	5.69722600
C	4.58350300	-7.95942700	5.09279200
C	2.88368600	-7.80616600	6.79721900
C	4.96520100	-9.20419900	5.58411200
H	5.10054800	-7.52972600	4.23889400
C	3.26547600	-9.05231400	7.28886300
H	2.07399000	-7.25959700	7.27322800
C	4.30606700	-9.75209600	6.68300000
H	5.77923500	-9.74408700	5.11232500
H	2.75278200	-9.47287500	8.14736700
H	4.60574000	-10.72076800	7.06853800
H	2.89065000	-4.58485200	2.59312000

TS$_{IX-X}$

C	0.81536000	0.06113600	-0.33908800
C	1.45132300	1.38359100	-0.58975100
H	2.39901600	1.30822100	-1.12395700
H	0.77060600	2.05186400	-1.11874700
C	1.59682300	-1.11905500	-0.15147000
C	2.92959800	-1.33625500	-0.88544500
C	2.99820900	-0.94304200	0.51731100
H	1.05629400	-2.01586900	0.12584300
H	3.18183200	-0.61203400	-1.65208500
H	3.10472000	-2.37291000	-1.14654300
H	3.19368600	-1.69338700	1.27342000
H	3.28615000	0.06905900	0.77263200
Si	0.00126800	0.12281900	-3.54973300
C	-0.29992100	1.97109900	-3.73939000
H	-1.02001100	2.09271700	-4.55738000
H	-0.80741200	2.35408800	-2.84556000
C	0.97033700	2.77818900	-4.04236800
H	1.43595700	2.44842600	-4.97518100
H	0.74303100	3.84235800	-4.14368400
H	1.72058100	2.67650400	-3.25206300
C	1.50387000	-0.50211300	-4.48637600
H	1.39417200	-0.21753100	-5.54007300
H	2.39130700	0.03093600	-4.12308200
C	1.68625400	-2.01962100	-4.35979800
H	2.59441900	-2.35797900	-4.86488200
H	1.75426600	-2.32840900	-3.31139700
H	0.84047700	-2.55389900	-4.80100900
H	0.29558100	-0.19731300	-2.07905000
H	-1.19600100	-0.69482300	-3.86138600
C	-0.59265200	0.02544300	0.18765300
H	-0.44650200	0.24343200	1.26008500
H	-0.96887400	-0.99897900	0.13661300
C	-1.58370400	0.99754300	-0.40390400
C	-2.52037300	0.53679500	-1.33248200
C	-1.58731800	2.34787200	-0.04444000
C	-3.42187300	1.41860100	-1.92398100
H	-2.54474900	-0.51785100	-1.59339300
C	-2.48886600	3.23053500	-0.63495400
H	-0.88877700	2.71145100	0.70507200
C	-3.40209300	2.76849700	-1.58084000
H	-4.14070400	1.04886600	-2.64750700
H	-2.48171100	4.27721300	-0.35029200
H	-4.10349700	3.45680700	-2.04001800
H	1.64246600	1.83808400	0.39249700

X

C	1.68109600	0.91753900	-0.95270900
C	2.46544900	2.21461300	-1.12265200
H	2.80440600	2.35532300	-2.15146200
H	1.82897600	3.06270800	-0.86346000
C	2.47463400	-0.33289600	-1.26750600
C	3.71601000	-0.35491500	-1.96468500
C	3.86042700	-0.57612900	-0.38236500
H	1.90780400	-1.25918400	-1.25392700
H	3.75224200	-1.57772400	0.02337400
H	3.76909900	0.24012600	0.33503100
C	1.03006300	0.80283400	0.44109400
H	1.80988600	0.87501300	1.21038400
H	0.57453200	-0.18947900	0.53906900
C	-0.01298700	1.86755500	0.68334400
C	-1.26663300	1.77423900	0.07150600
C	0.25949900	2.97238100	1.49252600
C	-2.22619300	2.76341800	0.26376700
H	-1.49461300	0.91450800	-0.55438400
C	-0.69879600	3.96506100	1.68783300
H	1.23020000	3.05344500	1.97555900
C	-1.94367400	3.86278500	1.07306100
H	-3.19640600	2.67480200	-0.21422100
H	-0.47254400	4.81610900	2.32199300
H	-2.69204900	4.63338100	1.22571400
H	0.87039000	0.90656700	-1.69515700
H	3.33865600	2.25590100	-0.45862000
Si	5.89523400	-0.40837200	-0.39109400
H	6.34195800	-1.08601900	-1.62390800
C	6.37911600	-1.32817100	1.15972600
H	7.43178100	-1.07123800	1.33922000
H	5.82092500	-0.91865700	2.00991200
C	6.25519800	1.42406600	-0.34070700
H	7.32659000	1.52706400	-0.55296600
H	5.74468700	1.94276600	-1.16018100
C	6.21511200	-2.85097800	1.07751400
H	6.56144400	-3.32609200	1.99769000
H	5.16990900	-3.14290600	0.93758700
H	6.79153600	-3.26860700	0.24809700
C	5.93069900	2.07699100	1.01130400
H	4.87404000	1.97393600	1.28447900
H	6.52064000	1.63314900	1.81718600
H	6.15191200	3.14597900	0.98590200
H	4.15014500	0.57579400	-2.31715000
H	4.00432000	-1.24653400	-2.51013200

$TS_{X\text{-}4aa}$

C	1.46710100	0.66951700	-1.14135200
C	2.26252300	1.89240400	-1.58025500
H	2.49213200	1.86402600	-2.64944600
H	1.68330200	2.79622700	-1.38382400
C	2.12268700	-0.63129300	-1.44756500
C	3.50844400	-0.80224800	-1.80726200
C	3.98290600	-0.86349400	-0.30484200
H	1.58287200	-1.52878200	-1.14221200
H	3.68427700	-1.80290800	0.16529800
H	3.61102400	-0.03300900	0.30324500
C	1.01085600	0.73422500	0.34893400
H	1.88413800	0.89777900	0.98795000
H	0.56899800	-0.22648900	0.63420400
C	0.01195300	1.84689900	0.55610200

C	-1.32030500	1.67467500	0.16980400
C	0.40686700	3.07024100	1.10015100
C	-2.24019700	2.70747700	0.32114900
H	-1.64206600	0.71995700	-0.24043900
C	-0.51271600	4.10584500	1.25468100
H	1.44066600	3.21176200	1.40595800
C	-1.83680600	3.92681800	0.86365400
H	-3.27268500	2.55906700	0.02220000
H	-0.19340700	5.05090300	1.68144700
H	-2.55371600	4.73208000	0.98443300
H	0.52329000	0.62431400	-1.70454600
H	3.20707900	1.97352200	-1.02926400
Si	5.90308800	-0.66186200	-0.29777800
H	6.40310600	-1.53860400	-1.38795400
C	6.53553600	-1.21698200	1.37969200
H	7.59235100	-0.92850500	1.44157400
H	6.01908400	-0.63935300	2.15587900
C	6.25601000	1.15251600	-0.64158600
H	7.32160400	1.25386000	-0.87661100
H	5.72379000	1.46694700	-1.54838600
C	6.37847800	-2.72117300	1.63444500
H	6.77928100	-3.00089900	2.61186300
H	5.32745700	-3.02448700	1.61399600
H	6.90689800	-3.30826000	0.87814500
C	5.89432200	2.06625200	0.53867600
H	4.84486400	1.96047900	0.83891400
H	6.50495900	1.83517900	1.41578800
H	6.05570700	3.11810300	0.29036700
H	3.95005600	0.04288800	-2.33528300
H	3.73606400	-1.74920500	-2.29321600
Si	0.92164400	-0.52596300	-4.54769700
H	1.35717500	-0.98183500	-3.16122700
H	0.28290000	-1.72059100	-5.15272300
C	-0.33771400	0.83996700	-4.26823000
H	0.13398600	1.65907900	-3.71031300
H	-0.60573600	1.26504900	-5.24247500
C	2.45374500	-0.00734600	-5.50016000
H	2.90224600	0.86759500	-5.01491700
H	3.19470900	-0.81214200	-5.43765600
C	-1.59874900	0.34183500	-3.54803200
H	-1.35906800	-0.16645700	-2.60676200
H	-2.27634600	1.16607400	-3.31101900
H	-2.14290100	-0.37619100	-4.16702700
C	2.13578600	0.31154600	-6.96940600
H	3.03939900	0.59650600	-7.51387000
H	1.70103000	-0.55404000	-7.47722400
H	1.42524000	1.13872000	-7.05408200

4aa

C	1.83121000	0.97121100	-0.44457900
C	2.93996000	1.85294500	0.12863500
H	3.83384000	1.83092400	-0.50279100
H	2.61202200	2.89324400	0.20621800
C	2.24323600	-0.50206900	-0.56798200
C	3.53117400	-0.73773100	-1.37716500
H	1.40893000	-1.04465400	-1.02870900
C	0.54437400	1.08112800	0.39533300
H	0.76773800	0.76114000	1.42117900
H	-0.19495800	0.37636000	-0.00460200
C	-0.04713900	2.46923400	0.41272100
C	-0.75112100	2.94852900	-0.69697500

C	0.12050000	3.31765100	1.51000600
C	-1.27364300	4.23846100	-0.71048000
H	-0.89367000	2.29752500	-1.55656600
C	-0.39981400	4.61047400	1.50206200
H	0.66605500	2.95847300	2.37926600
C	-1.09905300	5.07482400	0.39105200
H	-1.82099600	4.59073500	-1.57928700
H	-0.26000700	5.25380100	2.36522900
H	-1.50761500	6.08018800	0.38341700
H	1.59914700	1.33626700	-1.45662500
H	3.22372500	1.51237700	1.13237400
Si	6.39597300	-0.77868500	-1.52220600
H	6.25795700	-1.66792200	-2.71517500
C	7.87106100	-1.36456700	-0.49939300
H	8.79244600	-1.13127900	-1.04731800
H	7.90885600	-0.77095400	0.42277700
C	6.66283700	1.00307100	-2.09414600
H	7.44175800	1.01980300	-2.86524200
H	5.74729200	1.36101700	-2.58180500
C	7.82236100	-2.85937600	-0.15963300
H	8.68748100	-3.17007300	0.43345900
H	6.92513300	-3.10776400	0.41587400
H	7.80702200	-3.47011900	-1.06748900
C	7.04827900	1.94237000	-0.94271100
H	6.30063300	1.92664700	-0.14200300
H	8.00509300	1.65018000	-0.49913200
H	7.14386700	2.97883200	-1.27905100
H	3.64535100	0.08202300	-2.10141500
H	3.42707200	-1.64684500	-1.97940200
H	2.36627600	-0.92539100	0.43936200
C	4.79920600	-0.87654400	-0.51849800
H	4.75728100	-1.82336300	0.03460100
H	4.83368200	-0.08922500	0.24562000

TS$_{V\text{-}VI}$

C	0.57690400	-0.13597300	-0.62892000
C	-0.25002300	1.01898600	-1.22195900
H	-0.37604100	0.91761200	-2.30401200
H	0.27113500	1.96984900	-1.04469900
C	0.32178200	-0.19842000	0.88907200
C	-1.19765800	-0.17037800	1.13738800
C	1.00732600	-1.42181100	1.49375900
H	0.76421000	0.71359300	1.32928900
H	-1.60578400	-1.18009800	0.99590700
H	-1.43933000	0.13368500	2.16023000
H	0.95518600	-1.40250800	2.58846600
H	0.46860900	-2.32309600	1.17241400
Si	-1.86951100	0.98300400	-0.22210500
C	-2.36030200	2.68136500	0.43114000
H	-1.53510100	3.06959400	1.04142400
H	-2.46251300	3.37304300	-0.41426800
C	-3.30008000	0.21636900	-1.17938900
H	-2.95799800	-0.73432000	-1.60743500
H	-4.10459400	-0.03930700	-0.47878200
C	-3.65832400	2.64967100	1.24866300
H	-3.57677800	1.96242000	2.09730100
H	-3.91391500	3.63569900	1.64660000
H	-4.50188000	2.31256600	0.63762500
C	-3.83320200	1.13654700	-2.28545900
H	-3.04286500	1.40466100	-2.99446600
H	-4.63931000	0.66495000	-2.85434000

H	-4.22637800	2.06956300	-1.86872200
C	2.07108300	-0.01566000	-0.92202900
H	0.21237200	-1.08574000	-1.05161000
H	2.27773100	-0.16445400	-1.98699500
H	2.39774300	1.01021800	-0.69435300
C	2.93463800	-0.96814600	-0.12960500
C	2.45225300	-1.57864900	1.06145900
C	4.27048000	-1.18382100	-0.50713400
C	3.30199500	-2.47224800	1.77122300
C	5.10227100	-1.99120300	0.25045300
H	4.64856300	-0.69404000	-1.39925700
C	4.61431500	-2.65215200	1.39199400
H	2.90681900	-2.96771700	2.65330200
H	6.13699900	-2.12588900	-0.04687800
H	5.26628800	-3.30115700	1.96508600
H	3.28708700	-0.39197600	1.55397700
Si	3.89627800	1.56680000	2.56737000
H	3.25138500	2.27808500	1.44339800
H	4.01638100	0.08067700	1.99355200
C	5.70695000	1.96762900	2.76394500
H	5.76012200	3.00882500	3.10593200
H	6.12240600	1.35462100	3.56991100
C	2.83682700	1.34137500	4.08475500
H	2.83191700	2.29425900	4.62706200
H	1.80267200	1.16671400	3.76721900
C	6.50299100	1.78352500	1.46533800
H	6.09848900	2.40107800	0.65865200
H	7.54763900	2.06679500	1.61018700
H	6.48160300	0.74133500	1.13359800
C	3.32840600	0.20421800	4.99211700
H	2.68650100	0.10625300	5.87000100
H	3.32038500	-0.75532300	4.46550000
H	4.34762700	0.38247800	5.34473500

TS$_{V\text{-}C7H9+}$

C	0.89889300	-0.14350000	-0.57523600
C	0.05310900	1.05934800	-1.02908200
H	-0.25055900	0.97290900	-2.07656400
H	0.64804300	1.97855800	-0.93917200
C	0.89702400	-0.19138300	0.96460700
C	-0.55586700	-0.08127200	1.46280700
C	1.60836600	-1.44791700	1.46068400
H	1.45087200	0.69881200	1.31047500
H	-1.03540200	-1.06762200	1.40617100
H	-0.60590400	0.24090400	2.50734500
H	1.73765000	-1.42966700	2.54942500
H	0.97759300	-2.31868600	1.24030700
Si	-1.38338200	1.10185600	0.21925000
C	-1.68835400	2.82128000	0.92805000
H	-0.75996000	3.17476000	1.39402500
H	-1.89621000	3.51525800	0.10418500
C	-2.98224200	0.39641100	-0.48537700
H	-2.75491500	-0.57068400	-0.95125400
H	-3.67132800	0.17948600	0.34031100
C	-2.83764000	2.84753800	1.94432200
H	-2.64943700	2.15955200	2.77528200
H	-2.98377000	3.84465100	2.36895200
H	-3.78226100	2.54574100	1.48043800
C	-3.65056800	1.33238600	-1.50088600
H	-2.97730100	1.56133000	-2.33355800
H	-4.55849300	0.89304200	-1.92343400

H	-3.93034400	2.28415500	-1.03766900
C	2.33069500	-0.11150300	-1.10836200
H	0.41540500	-1.06915000	-0.92586000
H	2.35171600	-0.21154600	-2.19827600
H	2.76312000	0.87806200	-0.89079500
C	3.23054100	-1.15923300	-0.50365000
C	2.95600100	-1.68513300	0.79725700
C	4.40059400	-1.55145300	-1.15171200
C	3.81903600	-2.68658200	1.33128100
C	5.25878600	-2.48420900	-0.57767300
H	4.63349500	-1.12655300	-2.12317700
C	4.96505600	-3.06555900	0.66213500
H	3.56994300	-3.12135700	2.29581200
H	6.16331500	-2.77165300	-1.10385200
H	5.63307100	-3.80314400	1.09269800
H	3.77342500	-0.60922900	1.33038900
C	4.40763500	-0.57888600	4.11490100
C	5.66487500	-1.13363600	3.81487800
C	6.30894800	-0.77480500	2.62101000
C	5.69689300	0.07088100	1.71415400
C	4.38603300	0.55104500	1.97115100
C	3.78992800	0.28462200	3.23189500
H	3.93076200	-0.82304300	5.05874800
H	7.29515700	-1.17540400	2.40815800
H	6.18904100	0.33592800	0.78434500
H	3.96834700	1.32776800	1.33488600
H	2.82429900	0.72037800	3.46906300
C	6.30488700	-2.11771700	4.74862800
H	7.39243400	-2.02399300	4.73517900
H	5.94565600	-1.99089900	5.77095900
H	6.05738200	-3.13667700	4.43012900

TS$_{V\text{-}IX}$

C	0.86736800	-0.07861800	-0.38427500
C	0.13509600	1.13568300	-0.98104000
H	0.02482400	1.05367900	-2.06635500
H	0.71521300	2.04693300	-0.78147600
C	0.58355000	-0.12180100	1.12862100
C	-0.93800500	-0.02119100	1.34243700
C	1.19721400	-1.36436900	1.76924000
H	1.05235000	0.77481500	1.56539800
H	-1.39545300	-1.00547400	1.17559800
H	-1.18755400	0.28272700	2.36350400
H	1.13992600	-1.31062200	2.86194800
H	0.61480100	-2.24277700	1.46570200
Si	-1.50597400	1.19057200	-0.01429600
C	-1.90054500	2.90552700	0.65920900
H	-1.06440200	3.22959100	1.29158500
H	-1.94274800	3.61711000	-0.17464200
C	-2.96115500	0.53202900	-1.01317400
H	-2.67112800	-0.43109100	-1.45162400
H	-3.79412800	0.31640800	-0.33265100
C	-3.21340900	2.94052100	1.45271000
H	-3.19279400	2.23067700	2.28634100
H	-3.41179900	3.93200800	1.86919400
H	-4.06535000	2.67300200	0.81924000
C	-3.41233400	1.50329400	-2.11193500
H	-2.59202200	1.73366600	-2.79978800
H	-4.23426800	1.09360500	-2.70557400
H	-3.75503600	2.45154400	-1.68521600
C	2.37508700	-0.06821800	-0.64600100

H	0.44524300	-0.99714700	-0.82212400
H	2.59981400	-0.09984700	-1.71628200
H	2.78688800	0.88465000	-0.27447800
C	3.10144600	-1.18366900	0.04960600
C	2.65098500	-1.61864000	1.34799300
C	4.26374600	-1.73375100	-0.48264100
C	3.34713800	-2.71103100	1.97369600
C	4.94085000	-2.74938800	0.18664100
H	4.62996400	-1.37900700	-1.44055400
C	4.48362000	-3.24772200	1.41773800
H	2.97078100	-3.07603200	2.92626900
H	5.83958300	-3.16795000	-0.25548500
H	5.01907100	-4.04764700	1.91557500
H	3.28168200	-0.68018700	1.96779300
C	4.04967500	0.08175200	3.81303200
C	3.90638200	0.74206000	2.63271400
H	4.71763300	0.80748500	1.91104800
H	3.06810300	1.41648800	2.48524100
C	5.23469700	-0.71527200	4.16175900
C	6.27571200	-1.09950600	3.13569000
C	6.59889400	-0.02813100	4.10888500
H	5.09504800	-1.39578800	4.99586100
H	6.09006600	-0.82771900	2.10154700
H	6.73142300	-2.07388400	3.27042500
H	7.27938600	-0.24345500	4.92368000
H	6.59365300	0.99929500	3.76240500
C	2.96967200	0.13400200	4.86498700
H	2.65548400	-0.88869400	5.10702300
H	2.09797600	0.67451000	4.48228200
C	3.50210900	0.80063600	6.12117500
C	3.87313700	2.14782500	6.08601300

C	3.65491800	0.08358000	7.30733100
C	4.38071100	2.76893200	7.22208400
H	3.76179400	2.70930700	5.16132900
C	4.16390500	0.70429300	8.44700100
H	3.36868500	-0.96419300	7.34187100
C	4.52750200	2.04705700	8.40636700
H	4.66180500	3.81634600	7.18511600
H	4.27439000	0.13718400	9.36549300
H	4.92315200	2.53133700	9.29294500

TS$_{V\text{-}V'}$

C	0.55406200	0.10845500	-0.55941800
C	-0.19618500	1.24661100	-1.27307500
H	-0.07670900	1.19464000	-2.35919900
H	0.21159300	2.21304100	-0.94738900
C	-0.01566700	-0.03480100	0.86539500
C	-1.55039000	-0.11421600	0.77384700
C	0.59815200	-1.24781000	1.56080200
H	0.26280300	0.87295800	1.42396000
H	-1.84825100	-1.13675200	0.50543500
H	-2.02903000	0.12159400	1.72869700
H	0.30112400	-1.30841900	2.61215700
H	0.23055200	-2.16222300	1.07706500
Si	-1.98878000	1.06817600	-0.65612000
C	-2.72239800	2.69838300	-0.06245000
H	-2.08028200	3.09705700	0.73295300
H	-2.67907400	3.42816900	-0.88032700
C	-3.11831700	0.26580900	-1.93266400
H	-2.62863500	-0.64364000	-2.30326500
H	-4.03948000	-0.06643300	-1.43814300
C	-4.16466000	2.55160500	0.44043400

H	-4.22931200	1.82435900	1.25658200
H	-4.56443900	3.49927500	0.81170900
H	-4.82846300	2.20443100	-0.35793900
C	-3.45237600	1.20353700	-3.10042700
H	-2.54420500	1.54669400	-3.60693100
H	-4.08109700	0.71327600	-3.84882300
H	-3.98726600	2.09339400	-2.75314200
C	2.06731300	0.32293400	-0.52333300
H	0.34581100	-0.83643000	-1.08546400
H	2.49903900	0.27518700	-1.52765000
H	2.26724400	1.33774200	-0.14984700
C	2.80522400	-0.63334200	0.37781000
C	2.10837300	-1.29326400	1.44839400
C	4.15924100	-0.86809000	0.22961300
C	2.86340600	-2.15565600	2.32937900
C	4.87173700	-1.72379600	1.08971100
H	4.68939900	-0.37425400	-0.57900000
C	4.24370200	-2.35734900	2.14181500
H	2.32408700	-2.64478000	3.13505400
H	5.93354500	-1.87248200	0.92756500
H	4.78324800	-3.00621300	2.81990200
H	2.65025800	-0.82954200	2.49713100

TS1

C	0.61380900	-1.37004100	0.30333600
C	-0.48802000	-0.96060500	-0.74096800
H	-0.73486400	-1.83852300	-1.34775800
H	-0.07249300	-0.21126900	-1.41915400
C	-0.06619900	-1.81105300	1.55667900
C	-2.23012600	-1.93715100	1.37322500
C	-0.97785000	-2.91854100	1.61024400
H	-0.06910100	-1.14982800	2.42491700
H	-2.96295300	-2.59923900	0.89839200
H	-2.60825300	-1.62906500	2.34896500
H	-1.07638100	-3.39880300	2.58055600
H	-0.87298700	-3.62968400	0.79188400
Si	-2.06785700	-0.44054800	0.15713600
C	-1.87567900	1.16855900	1.11091700
H	-1.06137000	1.08046300	1.84118100
H	-1.56184400	1.94808700	0.40566800
C	-3.56109800	-0.38862300	-0.97291900
H	-3.60102300	-1.32577700	-1.54073600
H	-4.47109000	-0.36125200	-0.36174200
C	-3.17039200	1.58749300	1.82325200
H	-3.50222000	0.82367900	2.53392200
H	-3.03482700	2.51738700	2.38069600
H	-3.98346600	1.74653000	1.10858000
C	-3.52343700	0.81491700	-1.92648500
H	-2.62476500	0.80154000	-2.55118800
H	-4.38826000	0.81981600	-2.59423100
H	-3.52726300	1.76039700	-1.37530700
C	1.64330500	-0.26166400	0.53766600
H	1.12059800	-2.25917000	-0.09612500
H	1.97630400	0.11120700	-0.43427000
H	1.16863600	0.57983900	1.05813300
C	2.86000800	-0.76121600	1.33977100
H	3.54158000	0.07506400	1.51642500
H	3.39817100	-1.50831200	0.74781700
C	2.40678500	-1.35940400	2.64359300
C	2.12229600	-2.73000000	2.73946500
C	2.11545100	-0.53443400	3.73943400

C	1.55880500	-3.26403500	3.90532800
H	2.37865400	-3.38951900	1.91368300
C	1.54103200	-1.06255700	4.88845200
H	2.33161500	0.52873800	3.67678000
C	1.25283300	-2.43029800	4.97158300
H	1.35756500	-4.32863300	3.96652300
H	1.31650800	-0.41200700	5.72729800
H	0.80626500	-2.83683900	5.87240700

TS2

C	0.38795200	-0.85677100	0.29707100
C	-0.35742200	0.29837300	-0.39817900
H	-0.20912500	0.27264100	-1.48106200
H	0.03239900	1.25883900	-0.03812600
C	-0.25499500	-1.02102400	1.67504800
C	-1.81554600	-1.23012500	1.52555300
C	-0.09248600	-2.26334400	2.37208300
H	-0.07971300	-0.15127600	2.31547500
H	-2.08518900	-2.20740500	1.10921500
H	-2.36238300	-1.05908300	2.45340100
H	-0.27316100	-2.32331600	3.44232500
H	-0.14287000	-3.18894200	1.80462300
Si	-2.16488300	0.09683900	0.13507300
C	-2.90666100	1.64078100	0.90255100
H	-2.26806400	1.95858700	1.73555500
H	-2.85220400	2.44564200	0.15862900
C	-3.28562500	-0.64580600	-1.17398400
H	-2.78825300	-1.52241000	-1.60629900
H	-4.20040600	-1.01468000	-0.69499800
C	-4.35401000	1.44983100	1.37425500
H	-4.42792600	0.65992800	2.12838500
H	-4.75356100	2.36550200	1.81721200
H	-5.00924900	1.17187300	0.54293900
C	-3.63194000	0.37046100	-2.27250200
H	-2.73040000	0.74858100	-2.76469800
H	-4.26542600	-0.07626300	-3.04288200
H	-4.16857100	1.23157800	-1.86214700
C	1.89741200	-0.65114500	0.41767700
H	0.20401400	-1.78665400	-0.26304800
H	2.31083100	-0.50628400	-0.58487500
H	2.08909400	0.28047500	0.96526200
C	2.63467400	-1.82640300	1.07708700
H	3.71316700	-1.64672100	1.00927900
H	2.43921400	-2.74150600	0.50689500
C	2.30584700	-2.07971100	2.53577800
C	2.07109000	-3.38766800	3.00134600
C	2.37550600	-1.04100900	3.49151400
C	1.89500300	-3.65219800	4.36638500
H	2.06050500	-4.20751700	2.28781400
C	2.18132300	-1.30124000	4.83557600
H	2.58713900	-0.02745300	3.16389000
C	1.93903500	-2.61163500	5.27923200
H	1.72330600	-4.67007100	4.69808100
H	2.23156500	-0.49013000	5.55399900
H	1.79739500	-2.80650900	6.33661900

TS3

C	0.40488800	-0.93459400	0.27488400
C	-0.36450000	0.22168200	-0.39947300
H	-0.21918600	0.21083500	-1.48332800

H	0.02744000	1.17994200	-0.03408400
C	-0.23916900	-1.18502200	1.65283000
C	-1.77598400	-1.31564900	1.47017200
C	0.12197700	-2.45796300	2.38549600
H	-0.03975900	-0.32133700	2.30045000
H	-2.02940800	-2.29992900	1.05515800
H	-2.31161000	-1.20285800	2.41632500
H	-0.29274100	-2.48180700	3.38850700
H	-0.09143600	-3.35024500	1.79973600
Si	-2.16682400	0.02046400	0.15823100
C	-2.86086300	1.58333500	0.94384000
H	-2.20223000	1.87433400	1.77183800
H	-2.80342300	2.39906200	0.21245800
C	-3.31639500	-0.62482000	-1.18347300
H	-2.84810400	-1.49870500	-1.65275200
H	-4.24140800	-0.98715900	-0.71862000
C	-4.30290100	1.41667000	1.44015100
H	-4.37984800	0.60971300	2.17618400
H	-4.67667900	2.32928000	1.91215700
H	-4.97963800	1.17139500	0.61541700
C	-3.63646000	0.44099400	-2.24072500
H	-2.72445900	0.81603900	-2.71645600
H	-4.28110300	0.04589400	-3.03040000
H	-4.14922100	1.29946200	-1.79522900
C	1.89777400	-0.64218700	0.37899500
H	0.26399300	-1.84928600	-0.32281400
H	2.31688400	-0.50525700	-0.62179000
H	2.04062800	0.30767500	0.91041700
C	2.66503700	-1.76224000	1.08362300
H	3.73707000	-1.53530400	1.09599800
H	2.54273100	-2.70210100	0.53572100
C	2.25339200	-1.96795800	2.51503600
C	1.84262700	-3.24698500	2.96168000
C	2.37842900	-0.93043000	3.47726700
C	1.70535900	-3.52128800	4.36140300
H	1.91873500	-4.08224000	2.27335800
C	2.17789300	-1.19793200	4.80850500
H	2.67287700	0.06200600	3.15289400
C	1.85241800	-2.50639200	5.26273400
H	1.47135800	-4.53070800	4.67909600
H	2.29773700	-0.40257800	5.53669000
H	1.74050000	-2.69017900	6.32491500

9 References

[S1] (*a*) J. B. Lambert, L. Lin and S. Keinan, *Org. Biomol. Chem.*, 2003, **1**, 2559–2565; (*b*) C. Wang, G. Erker, G. Kehr, K. Wedeking and R. Fröhlich, *Organometallics*, 2005, **24**, 4760–4773.

[S2] C. A. Reed, *Acc. Chem. Res.*, 2010, **43**, 121–128.

[S3] R. K. Harris, E. D. Becker, S. M. Cabral de Menezes, R. Goodfellow and P. Granger, *Pure. Appl. Chem.,* 2001, **73**, 1795–1818.

[S4] (*a*) Z. Xie, R. Bau, A. Benesi and C. A. Reed, *Organometallics*, 1995, **14**, 3933–3941; (*b*) L. Omann, B. Pudasaini, E. Irran, H. F. T. Klare, M.-H. Baik and M. Oestreich, *Chem. Sci.*, 2018, **9**, 5600–5607.

[S5] T. He, G. Wang, V. Bonetti, H. F. T. Klare and M. Oestreich, *Angew. Chem., Int. Ed.*, 2020, **59**, 12186–12191.

[S6] K. Schwärzer, A. Bellan, M. Zöschg, K. Karaghiosoff and P. Knochel, *Chem.–Eur. J.*, 2019, **25**, 9415–9418.

[S7] R. Boobalan, R. Santhoshkumar and C. Cheng, *Adv. Synth. Catal.*, 2019, **361**, 1140–1145.

[S8] R. Alexandru C, B. Emeric, A. Zsolt and G. Marie, *Rev. Roum. Chim.*, 1984, **29**, 719–725.

[S9] H. Zheng, X. Shan, J. Qu and Y. Kang, *Org. Lett.*, 2018, **20**, 3310–3313.

[S10] S. Tanpure, M. El-Mansy and P. Blakemore, *Org. Lett.*, 2020, **22**, 2999–3003.

[S11] P. Long, T. He and M. Oestreich, *Org. Lett.*, 2020, **22**, 7383–7386.

[S12] J. Liu, H. Yao, X. Li, H. Wu, A. Lin, H. Yao, J. Xu and S. Xu, *Org. Chem. Front.*, 2020, **7**, 1314–1320

[S13] A. Hoyt and P. Blakemore, *Tetrahedron Lett.*, 2015, **56**, 2980–2982.

[S14] S. Chanthamath, S. Takaki, K. Shibatomi and S. Iwasa, *Angew. Chem., Int. Ed.*, 2013, **52**, 5818–5821.

[S15] R. Larock, W. Leung and S. Stolz-Dunn. *Tetrahedron Lett.*, 1989, **30**, 6629–6632.

[S16] E. Larionov, L. Lin, L. Guénée and C. Mazet, *J. Am. Chem. Soc.*, 2014, **136**, 16882–16894.

[S17] G. Wang, J. Jiang, X. Bu, J. Dai, J. Xu, Y. Fu and H. Xu. *Org. Lett.*, 2015, **17**, 3682–3685.

[S18] N. Tsukada and J. Hartwig, *J. Am. Chem. Soc.*, 2005, **127**, 5022–5023.

[S19] B. Satpathi, L. Dutta and S. Ramasastry, *Org. Lett.*, 2019, **21**, 170–174.

[S20] X. Ning, S. Lou, Y. Mao, Z. Xu and D. Xu, *Org. Lett.*, 2018, **20**, 2445−2448.

[S21] L. Ren, M. Yang, C. Tung, L. Wu and H. Cong, *ACS Catal.*, 2017, **7**, 8134−8138.

[S22] *Agilent CrysAlis PRO*, 2012, Agilent Technologies, Yarnton, UK.

[S23] G. M. Sheldrick, *Acta Crystallogr., Sect. A.*, 1990, **46**, 467–473.

[S24] G. M. Sheldrick, *Acta Crystallogr., Sect. A.*, 2008, **64**, 112–122.

[S25] Mercury 3.9; Cambrige Crystallographic Data Center: https://www.ccdc.cam.ac.uk/solutions/csd-system/components/mercury/.

[S26] Gaussian 16, Revision A.03, M. J. Frisch, G. W. Trucks, H. B. Schlegel, G. E. Scuseria, M. A. Robb, J. R. Cheeseman, G. Scalmani, V. Barone, G. A. Petersson, H. Nakatsuji, X. Li, M. Caricato, A. V. Marenich, J. Bloino, B. G. Janesko, R. Gomperts, B. Mennucci, H. P. Hratchian, J. V. Ortiz, A. F. Izmaylov, J. L. Sonnenberg, D. Williams-Young, F. Ding, F. Lipparini, F. Egidi, J. Goings, B. Peng, A. Petrone, T. Henderson, D. Ranasinghe, V. G. Zakrzewski, J. Gao, N. Rega, G. Zheng, W. Liang, M. Hada, M. Ehara, K. Toyota, R. Fukuda, J. Hasegawa, M. Ishida, T. Nakajima, Y. Honda, O. Kitao, H. Nakai, T. Vreven, K. Throssell, J. A. Montgomery, Jr., J. E. Peralta, F. Ogliaro, M. J. Bearpark, J. J. Heyd, E. N. Brothers, K. N. Kudin, V. N. Staroverov, T. A. Keith, R. Kobayashi, J. Normand, K. Raghavachari, A. P. Rendell, J. C. Burant, S. S. Iyengar, J. Tomasi, M. Cossi, J. M. Millam, M. Klene, C. Adamo, R. Cammi, J. W. Ochterski, R. L. Martin, K. Morokuma, O. Farkas, J. B. Foresman, and D. J. Fox, Gaussian, Inc., Wallingford CT, 2016.

[S27] C. Y. Legault, CYLview, version 1.0b; Universitéde Sherbrooke: Quebec, Canada, 2009; http://www.cylview.org.

[S28] (*a*) Y. Zhao and D. G. Truhlar, *Theor. Chem. Acc.*, 2008, **120**, 215–241; (*b*) Y. Zhao and D. G. Truhlar, *Acc. Chem. Res.*, 2008, **41**, 157–167.

[S29] J. Tomasi and M. Persico, *Chem. Rev.*, 1994, **94**, 2027–2094.
